# Highly selective catalytic *trans*-hydroboration of alkynes mediated by borenium cations and B(C_6_F_5_)_3_†
†Electronic supplementary information (ESI) available: Complete experimental details, NMR spectra and crystallographic data in .cif format. CCDC 1429287–1429289 and 1440939. For ESI and crystallographic data in CIF or other electronic format see DOI: 10.1039/c5sc04798f


**DOI:** 10.1039/c5sc04798f

**Published:** 2016-02-12

**Authors:** John S. McGough, Samuel M. Butler, Ian A. Cade, Michael J. Ingleson

**Affiliations:** a University of Manchester , Oxford Road , Manchester M13 9PL. , UK . Email: michael.ingleson@manchester.ac.uk

## Abstract

The *trans*-hydroboration of terminal alkynes mediated by borenium cations [NHC(9-BBN)]^+^ (NHC = N-heterocyclic carbene, 9-BBN = 9-borabicyclo(3.3.1)nonane) exclusively affords *Z*-vinylboranes. NHCs and chelating dialkyl substituents on the borenium cation and “non”-basic anions were essential to preclude alternative reactions including dehydroboration. Deuterium labelling studies indicate the mechanism involves addition of the boron electrophile to the alkyne and transfer of hydride to the opposite face of the activated alkyne. *trans*-Hydroboration proceeds with only catalytic amounts of B(C_6_F_5_)_3_ or [Ph_3_C][B(C_6_F_5_)_4_] to activate the (NHC)9-BBN(H) precursor with the borenium regenerated in the hydride transfer step. The NHC can be removed from the *trans*-hydroborated products by the addition of Et_2_O–BF_3_ providing access to vinylBBN species effective for Suzuki–Miyaura couplings to generate *Z*-alkenes. Combinations of catalytic B(C_6_F_5_)_3_ and stoichiometric [HB(C_6_F_5_)_3_]^–^ also lead to *trans*-hydroboration of terminal alkynes to form *Z*-isomers of [arylCH=CHB(C_6_F_5_)_3_]^–^.

## Abstract 2

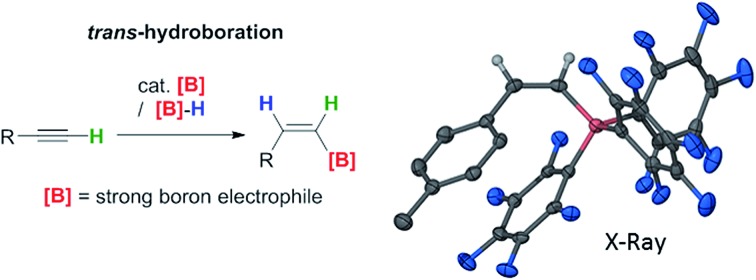
Transition metal free alkyne *trans*-hydroboration is achieved using strong boron electrophiles in the presence of a B–H moiety.

## Introduction

Vinylboranes and boronate esters are ubiquitous reagents in synthesis widely used in C–C, C–N and C–O bond formation.^1^ The hydroboration reaction is a powerful transformation for generating vinylboranes which with terminal alkynes yields *E*-vinylboranes *via syn*-addition of a B–H moiety.^2^ In contrast, the *trans*-hydroboration of terminal alkynes to form *Z*-vinylboranes is rare with the first example only reported in 2000 using Rh or Ir catalysts.^3^ Subsequent breakthroughs have been limited excluding four notable exceptions using Ru, Pd and Co based catalysts.^4^–^6^ A transition metal free catalytic methodology for *trans*-hydroboration of alkynes to form *Z*-vinyl boranes using strong boron electrophiles represents a conceptually new approach not realised to date. In contrast, the transition metal free *trans*-hydrosilylation of alkynes has been reported using AlCl_3_ as activator.^7^ This was subsequently applied to alkene and alkyne *trans*-hydrosilylation using other main group Lewis acids as catalysts.^8^,^9^ In these systems the unusual selectivity is achieved by a stepwise mechanism, with activation of the alkyne by a silicon electrophile followed by subsequent transfer of a hydride to the opposite face in a steric controlled step. An analogous stepwise approach has not been reported for boron electrophiles. This is partly due to the challenge of generating a sufficiently electrophilic borane to activate the alkyne that does not then engage in *syn*-1,2- or 1,1-elementoboration (eqn (1) and (2)) of alkynes.

**Fig. 1:**
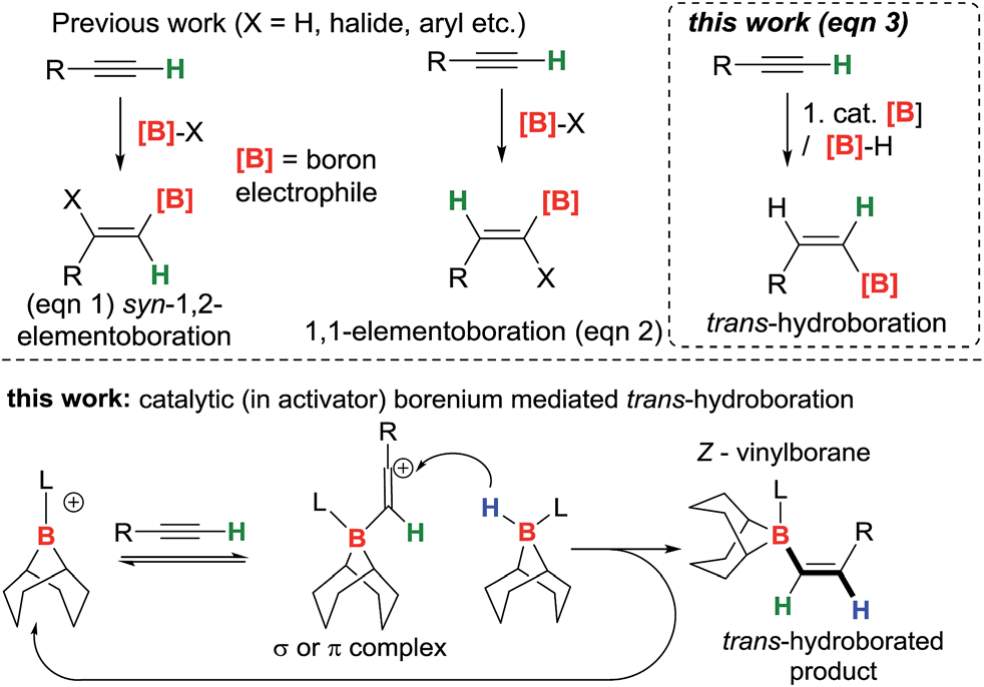
Previous work on alkyne elemento-boration (eqn (1) and (2)), and the work described herein (eqn (3)). Bottom, intermolecular hydride transfer for *trans*-hydroboration (intermediate shown as a vinyl cation a π complex between borenium and alkyne is also feasible).

The *trans*-hydroboration of alkynes could be achieved by using an appropriate borenium cation^10^ to activate the alkyne followed by intermolecular hydride transfer from a borane to the less hindered face of the intermediate. Borenium cations have been used in the elementoboration of alkynes but to date only *via syn*-1,2 or 1,1 addition to the alkyne.^11^–^14^ Quenching the borenium activated alkyne with an external donor (essential for *trans*-selectivity) requires strongly bound “non-migratory” groups on boron to preclude *syn*-1,2- or 1,1-elementoboration; thus chelating dianionic substituents and a strong Lewis base are essential. The transfer of a hydride intermolecularly from a borane-Lewis adduct would generate further equivalents of the borenium ion and thus render *trans*-hydroboration catalytic in the activator initially used to form the borenium cation (Fig. 1, bottom). A related catalytic cycle has been reported, albeit for imine hydroboration, using [PinB(DABCO)][HB(C_6_F_5_)_3_] as catalyst.^15^ Herein we report the *trans*-hydroboration of terminal alkynes using catalytic B(C_6_F_5_)_3_ as an activator. The reaction exclusively generates *Z*-vinylboranes in excellent yields that can be used for subsequent Suzuki–Miyaura couplings to form *Z*-alkenes.

## Results and discussion

**Scheme 1:**
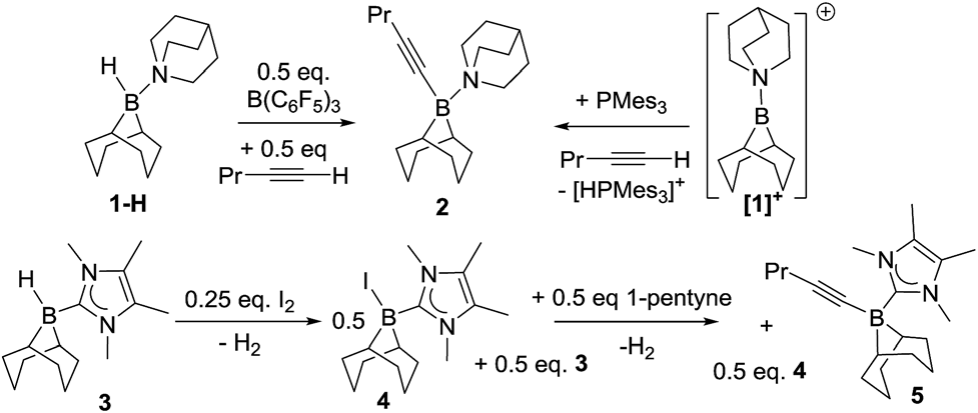
Alkyne dehydroboration with boreniums (or functional equivalents).

Initial studies used 9-BBN(H)(amine) (9-BBN = 9-borabicyclo[3.3.1]nonane) ligated with strongly nucleophilic amines in an attempt to disfavour amine dissociation and alkyne *cis*-hydroboration.^2^ However, using 9-BBN(H)(quinuclidine), **1-H**, in 1,2-C_6_H_4_Cl_2_ with 50 mol% B(C_6_F_5_)_3_ as activator dehydroboration of 1-pentyne to form 9-BBN(1-pentynyl)(quinuclidine), **2**, and *cis*-hydroboration (from the reaction of free BBN and 1-pentyne) were the major outcomes (the identity of **2** was confirmed by independent synthesis using the frustrated Lewis pair (FLP) of [**1**][B(C_6_F_5_)_4_]/P(mesityl)_3_, Scheme 1, top right).^16^ Presumably dissociation of quinuclidine from boron is occurring at some point during the reaction enabling deprotonation of the borenium activated alkyne and formation of **2**.

N-Heterocyclic carbenes (NHCs) are stronger sigma donors than amines and 1,3,4,5-tetramethylimidazolylidene (IMe_4_) was selected as its low steric bulk (%*V*_bur_ = 26.1%)^17^ maximises nucleophilicity favouring irreversible binding to boron whilst minimising the kinetic barrier to alkyne and borenium cation interaction. Curran and co-workers have previously used (NHC)BH_3_ compounds activated with I_2_ to form (NHC)BH_2_I (a functional equivalent of a borenium) in alkene *syn*-hydroboration.12*b* 9-BBN(H)(IMe_4_), **3** was readily synthesised and activated with I_2_ (0.25 equiv.) forming a 1 : 1 mixture of 9-BBN(I)(IMe_4_) (**4**) and **3***in situ*. Subsequent addition of 1-pentyne to this mixture led to dehydroboration to form the alkynyl borane, **5**, instead of *trans*-hydroboration. Addition of further equivalents of **3** and 1-pentyne to this reaction demonstrated that it is catalytic (in I_2_) with further **5** produced along with H_2_ (presumably from reaction of the HI by-product from dehydroboration with **3** which regenerates **4**). Iodide is therefore sufficiently Brønsted basic to deprotonate the borenium activated alkyne; thus “non”-basic anions and NHCs are both required to realise *trans*-hydroboration. Whilst not-productive for *trans*-hydroboration the formation of **5** does represent a catalytic (in activator) electrophilic C–H borylation with H_2_ the only by-product and this topical catalytic conversion^18^ is being explored in a separate study.

**Scheme 2:**
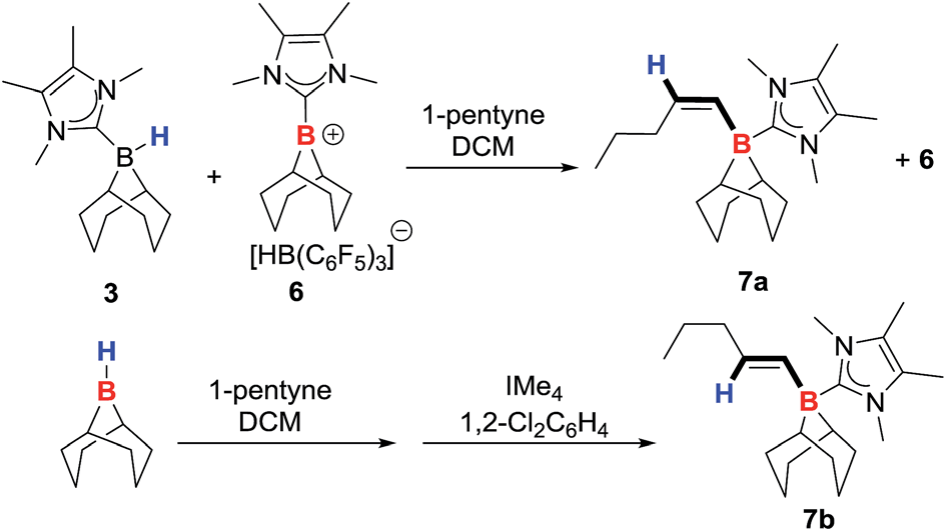
Hydroboration of 1-pentyne to give **7a** and **7b**.

The reaction of **3** with 1 equivalent of B(C_6_F_5_)_3_ affords the borenium salt [9-BBN(IMe_4_)][HB(C_6_F_5_)_3_] (**6**) quantitatively by NMR spectroscopy. 1 : 1 mixtures of **3** and **6** only display a single broad ^11^B NMR signal (instead of discrete signals for the adduct **3** and the borenium **6**) that shifts depending on the ratio of **3** : **6**. This is attributed to rapid hydride transfer between the boron centres *via* a hydride bridged intermediate.^19^,^20^ Low temperature NMR studies on mixtures of **3** and **6** did not reach the slow exchange regime at –80 °C in CD_2_Cl_2_. Nevertheless, a 50 : 50 mixture of **3** and **6** in DCM reacts with 1-pentyne to generate a new signal in the ^11^B NMR spectrum at –16.1 ppm, along with 2 vinyl proton signals in a 1 : 1 ratio at 6.03 ppm (d, ^3^*J*_HH_ = 13.3 Hz) and 5.54 ppm (dt, ^3^*J*_HH_ = 13.2 and 6.1 Hz). These two signals correspond to the alkene protons of the *trans*-hydroborated product 9-BBN(IMe_4_)-1-pentene (**7a**, Scheme 2) based on 2D NMR experiments, and by comparison with the *cis*-hydroborated *E*-alkyne generated by the hydroboration of 1-pentyne by 9-BBN followed by the addition of IMe_4_. The *E*-vinylborane (**7b**) showed vinyl signals at 6.01 ppm (d, ^3^*J*_HH_ = 17.4 Hz) and 4.74 ppm (dt, ^3^*J*_HH_ = 17.5 and 6.4 Hz). The large ^3^*J*_HH_ coupling constant observed between the *cis*-vinyl protons in **7a** is comparable with the ^3^*J*_HH_ coupling constants observed for other *Z*-vinylboranes (which are generally in the range of 13–15 Hz),^3^,4a,^21^ further supporting isomer identity. Whilst this provided proof of principle for alkyne *trans*-hydroboration using boreniums the reaction takes days for significant conversion even with 50 mol% of B(C_6_F_5_)_3_ (relative to **3**).

Enhancing electrophilicity at boron would potentially increase the rate of reaction. However, attempts using catecholborane/NHC derived species led to rapid ligand scrambling, therefore modification of the NHC was explored. Stephan and co-workers previously used the chlorinated NHC 1,3-dimethyl-4,5-dichloroimidazolylidene (I-DCDM) to increase the rate of hydrogenation of imines catalysed by [9-BBN(NHC)][B(C_6_F_5_)_4_], presumably due to the enhanced electrophilicity at boron.19*b* The neutral adduct 9-BBN(H)(I-DCDM) (**8**) was reacted with B(C_6_F_5_)_3_ to generate the borenium species [9-BBN(I-DCDM)][HB(C_6_F_5_)_3_] (**9**). In DCM a 1 : 1 mixture of **8** and **9** shows similar ^11^B NMR spectra to that of **3** and **6** indicating a fast exchange. A 1 : 1 : 1 mixture of **8**, **9** and 1-pentyne resulted in rapid consumption of the alkyne to produce a number of species with alkene proton signals. The major new product had a ^11^B NMR signal at –15.9 ppm, and two vinyl protons, a doublet at 5.96 ppm (^3^*J*_HH_ = 13.20 Hz), and a pseudo quintet (overlapped d of t, ^3^*J*_HH_ = 6.60, 13.20 Hz) at 5.59 ppm consistent with *trans*-hydroboration of the alkyne (confirmed by 2D and NOE NMR spectroscopy). The minor products that increase with time are attributed to borocation induced isomerisation reactions (analogous to that observed by Curran, Vedejs, Lacôte and co-workers during borenium mediated *syn*-hydroboration).12*a* Reducing the loading of B(C_6_F_5_)_3_ reduces the rate of vinylborane-isomerisation; however, neither low loadings of B(C_6_F_5_)_3_ or lower temperatures prevented the formation of the minor products. Furthermore, using **8**/10 mol% B(C_6_F_5_)_3_*trans*-hydroboration does not go to completion, with *ca.* 30% conversion observed (by ^1^H NMR spectroscopy). Nevertheless, the observation of *ca.* 3 turnovers confirmed that catalytic in B(C_6_F_5_)_3_*trans*-hydroboration is indeed viable.

**Scheme 3:**
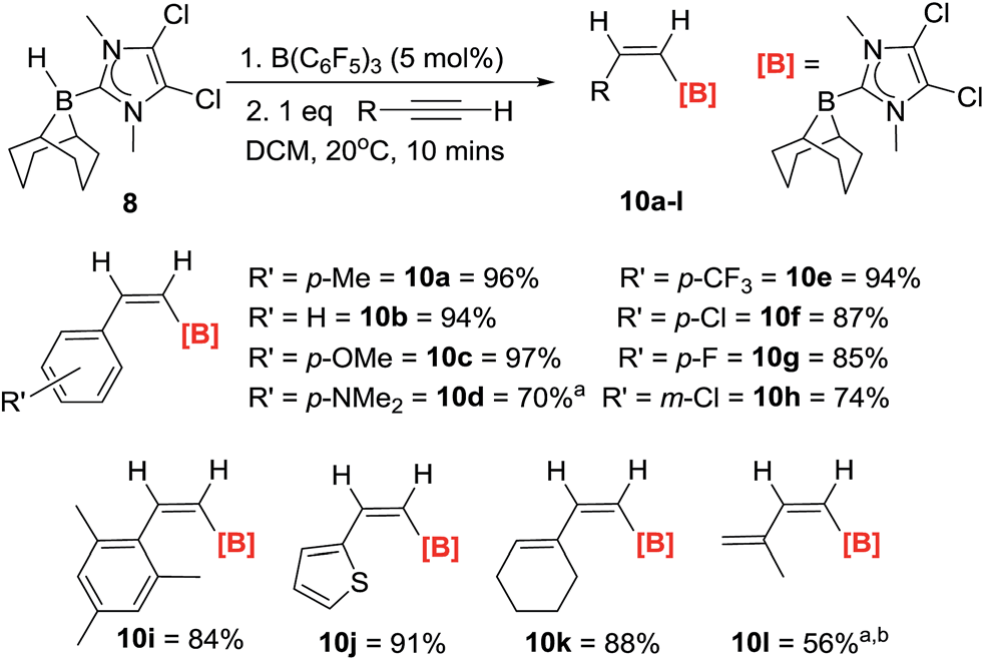
Reaction scope and isolated yields (unless otherwise stated) for the *trans*-hydroboration of terminal alkynes. *a* = yield by NMR spectroscopy *versus* mesitylene as internal standard. *b* = 10 mol% B(C_6_F_5_)_3_.

The hydroboration of a range of arylacetylenes was subsequently investigated as these substrates are not amenable to extensive hydride/borane migration derived isomerisation during or post *trans*-hydroboration. The reaction of **8**/10 mol% B(C_6_F_5_)_3_ and 4-ethynyltoluene proceeds rapidly to give a single product with an ^11^B NMR signal at –14.9 ppm. The *in situ*^1^H NMR spectrum in DCM shows two doublets (6.79 ppm, ^3^*J*_HH_ = 14.4 Hz and 6.62 ppm, ^3^*J*_HH_ = 14.4 Hz) for the *cis*-vinyl protons. The magnitude of this coupling constant is again fully consistent with other *Z*-vinyl boranes,^3^,4a,^21^ and the stereochemistry was unambiguously assigned *via* 2D-NOE NMR experiments. It was found that 5 mol% B(C_6_F_5_)_3_ was optimal for complete *trans*-hydroboration, with the reaction being finished within 5 minutes at room temperature in CH_2_Cl_2_. The products are then isolated without column chromatography simply by addition of pentane, filtration (removing minor quantities of ionic by-products) and drying. *trans*-Hydroboration is tolerant of a range of functional groups (Scheme 3) and in all cases only the *Z*-vinylborane isomer was formed with no *E*-isomer or over reduction products (diborylated alkanes) observed. Electron donating groups such as *para*-methoxy (**10c**) and *para*-NMe_2_ (**10d**) are compatible as are electron withdrawing groups including *p*-chloro- (**10e**), *p*-fluoro- (**10f**) *p*-trifluoromethyl- (**10g**) and *m*-chloro (**10h**) substituted phenylacetylenes. The substrate scope was extended to a hindered alkyne with mesityl acetylene forming **10i** in good yield (84%). The compatibility of hetero-aromatic substrates was demonstrated using 2-ethynylthiophene with the formation of **10j** in an excellent yield (91%). The reaction is not limited to heteroaryl and arylacetylenes with conjugated enynes also amenable with hydroboration exclusively occurring at the alkyne, affording the borylated dienes **10k** and **10l**. The *trans*-hydroboration of alkyl alkynes such as 1-octyne is problematic in terms of isolating the *trans*-hydroborated products cleanly due to intractable minor products from isomerisation reactions.^12^ However, the *trans*-hydroboration products are the major products formed.

**Scheme 4:**
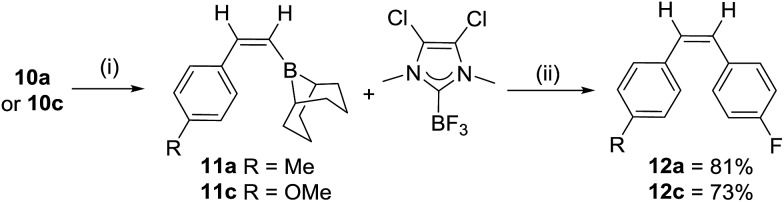
Suzuki Miyaura cross coupling of **10x**. (i) 1 equiv. BF_3_(OEt_2_), toluene, 60 °C, 1 h. (ii) Dried *in vacuo*, then Pd(P^*t*^Bu_3_)_2_ (10 mol%), 4-iodo-fluorobenzene (1.1 eq.), THF, KO^*t*^Bu (2.5 eq.) 20 °C, 18 h.

The cross coupling of **10a** with 4-iodo-fluorobenzene using a range of Pd catalysts/bases principally generated *para*-methylstyrene from proto-deborylation. It was hypothesized that either NHC dissociation at raised temperatures was poisoning the Pd catalyst or strong NHC binding to boron prevented transmetallation, thus prior removal of the carbene was explored. Heating **10a** and one equiv. of BF_3_:OEt_2_ in toluene at 60 °C for 1 h led quantitatively to the formation of **11a** and the NHC–BF_3_ adduct (Scheme 4). Importantly, there was no loss of alkene *Z*-stereochemistry (by NOE spectroscopy). After removal of toluene, the mixture of **11a** (or **11c**) and NHC–BF_3_ can be used in Suzuki–Miyaura couplings without any purification. Cross coupling proceeds to give exclusively the *cis*-stilbenes **12a** and **12c** in good isolated yields (81 and 73%, respectively), with the NMR spectra of **12c** consistent with that previously reported,^22^ further confirming the *trans* selectivity of the hydroboration. The NHC adducts **10a–l** therefore represent air stable precursors that are readily unmasked for use in Suzuki–Miyaura reactions (and other transformations previously reported using vinylBBN species),^23^ analogous to recent elegant work using NHCBH_2_(aryl) compounds as boronic acid precursors.^24^

### Mechanistic studies

**Scheme 5:**
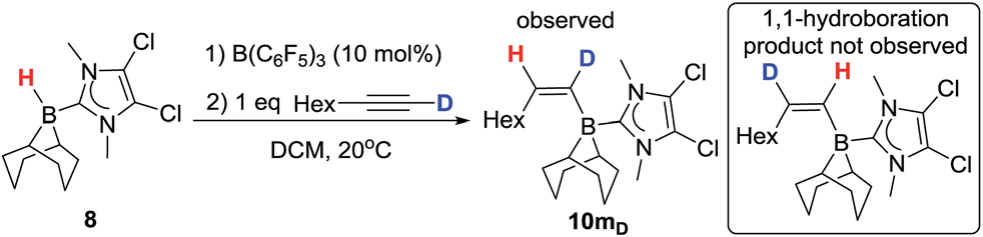
Deuterium labelling experiment with **8**.

The mechanism for formation of **10a–l** could proceed by 1,2-hydroboration or 1,1-hydroboration. 1,1-Hydroboration would involve a 1,2-hydride shift in the borenium activated intermediate prior to intermolecular hydride transfer; related 1,2-hydride shifts have been reported on combination of B(C_6_F_5_)_3_ with terminal alkynes which ultimately results in 1,1-carboboration of the alkyne.^25^ The hydroboration of 1-octyne with **8** proceeds to give a single major hydroborated product **10m** (by ^1^H NMR spectroscopy, using 10 mol% B(C_6_F_5_)_3_), with signals at 5.95 ppm (d, ^3^*J*_HH_ = 13.2 Hz for C1 bound proton) and 5.61 (dt, ^3^*J*_HH_ = 13.2 and 6.5 Hz) for the C2 proton. In contrast, when 1-octyne-d_1_ is used, **10m_D_** forms with the signal at 5.95 ppm not observed whilst a vinylic signal at 5.62 ppm is observed although now as a triplet (^3^*J*_HH_ = 5.6 Hz); furthermore a vinyl deuteron signal (5.37 ppm) is seen in the ^2^H NMR spectrum (see ESI†). Based on these results, we conclude that the reaction is a 1,2-*trans*-hydroboration and not 1,1-hydroboration (Scheme 5).

**Scheme 6:**
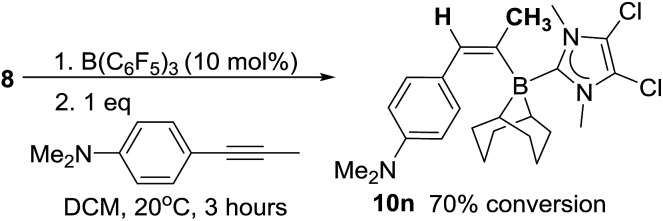
*trans*-Hydroboration of an internal alkyne.

With deuterium labelling studies indicating a 1,2-*trans*-hydroboration mechanism the *trans*-hydroboration of internal alkynes is feasible. The reaction with a range of internal alkynes was not successful under a range of catalytic loadings and conditions with minimal activation of the alkyne observed (<5%).^26^ However, using the nucleophilic internal alkyne 4-(1-propynyl)-*N*,*N*-dimethylaniline *trans*-hydroboration was effective, albeit at a slower rate to that observed with terminal alkynes. After stirring for 3 hours the ^11^B NMR spectra shows one major new signal at –12.6 ppm, along with a new singlet in the ^1^H NMR spectrum at 6.68 ppm indicating a single new vinyl borane product is formed in 70% conversion and confirmed as the *E* isomer by NMR spectroscopy (**10n**, Scheme 6) thus is formed by *trans*-1,2-hydroboration. This represents the first highly selective *trans*-hydroboration of an unsymmetric internal aryl-alkyne, as previous work produced varying ratios of isomeric products.4*b*

**Fig. 2:**
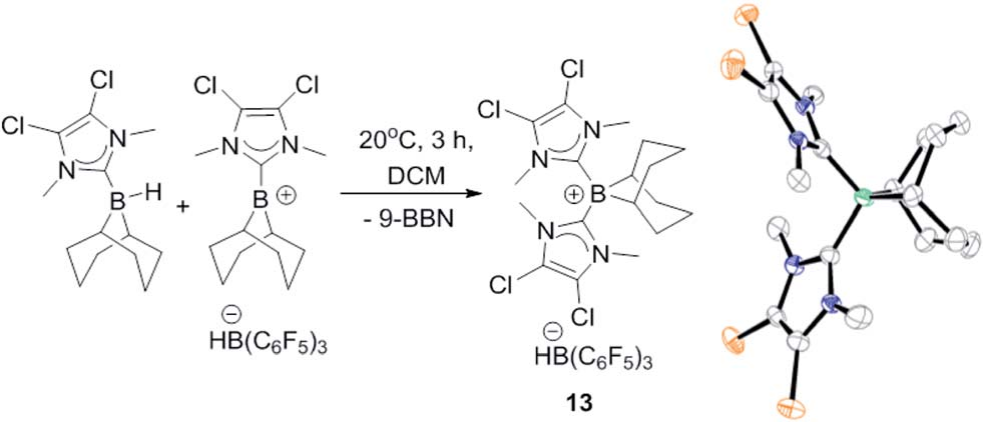
Formation of **13** and structure of **13** (thermal ellipsoids at 50% probability). Hydrogens and [HB(C_6_F_5_)_3_]^–^ omitted for clarity.

The failure to *trans*-hydroborate the majority of internal alkynes investigated is in part due to decomposition of the mixtures of **8** and **9** over the longer reaction times required (relative to terminal alkynes which react within minutes). A freshly prepared solution of equimolar **8** and **9** shows only a single broadened signal (due to fast exchange) in the ^11^B NMR spectrum but on standing for 3 h at 20 °C in DCM a new ^11^B NMR signal grows in at –12.8 ppm, concurrent with the signal for **8** (–17 ppm, ^1^*J*_BH_ = 79 Hz). The signal at –12.8 ppm in the ^11^B NMR spectrum also is observed in the reactions with internal alkynes that do not undergo *trans*-hydroboration indicating that borenium cation decomposition out-competes the slower *trans*-hydroboration of most internal alkynes. The new boron species was isolated by layering a DCM solution with hexanes to produce single crystals suitable for X-ray diffraction analysis. This revealed the compound to be the boronium salt [9-BBN(I-DCDM)_2_][HB(C_6_F_5_)_3_] (**13**, Fig. 2). Borenium decomposition therefore proceeds by NHC transfer which may occur *via* the hydride bridged intermediate, with NHC transfer coming within the solvent shell *via* a concerted process. No activation of the DCM solvent by the NHC is observed, disfavouring an intermolecular pathway (**8** is stable in DCM for days at 20 °C, precluding NHC → B dissociation as NHCs react rapidly with DCM).^27^ The by-product from formation of **13** is 9-BBN and low quantities of alkyne *cis*-hydroboration products also are observed during attempted catalytic *trans*-hydroboration of internal alkynes. Attempts to preclude this decomposition pathway by using larger *N*-substituents on the NHC (*e.g.*, mesityl) was successful in preventing boronium formation, but these more sterically hindered borenium/BBN(H)(NHC) mixtures did not react even with terminal alkynes, presumably due to a larger steric barrier.

*trans*-Hydroboration is proposed to proceed by activation of the alkyne by **9** to generate a vinyl cation (or π complex), which is then quenched by transfer of a hydride to the activated alkyne to generate **10x**; however, this last step could involve hydride transfer from **8** or from [HB(C_6_F_5_)_3_]^–^ (with the latter finding precedence in olefin reduction).^28^ The former will regenerate an equivalent of **9** directly whilst if hydride transfer is from [HB(C_6_F_5_)_3_]^–^ the subsequent reaction of B(C_6_F_5_)_3_ with **8** will then reform **9**. Hydride transfer is feasible directly from **8** as the use of 5 mol% of [Ph_3_C][B(C_6_F_5_)_4_] as an activator in place of B(C_6_F_5_)_3_ in the *trans*-hydroboration of ethynyltoluene proceeded with no loss of activity (complete in <5 minutes at 20 °C) to generate **10a** in an excellent yield (97%). The reaction of stoichiometric **9** with 4-ethynyltoluene also led to *trans*-hydroboration and formation of **10a** and B(C_6_F_5_)_3_, (as observed by *in situ* NMR spectroscopy), indicating that [HB(C_6_F_5_)_3_]^–^ is able to transfer hydride to the activated alkyne as in the absence of **8** the anion is the only hydride source. The extremely rapid reaction at 5 mol% loading precludes rate comparison between [B(C_6_F_5_)_4_]^–^ and [HB(C_6_F_5_)_3_]^–^, whilst attempts with lower activator loadings led to irreproducible results due to the sensitivity of borenium cations to varying quantities of trace protic impurities.

**Fig. 3:**
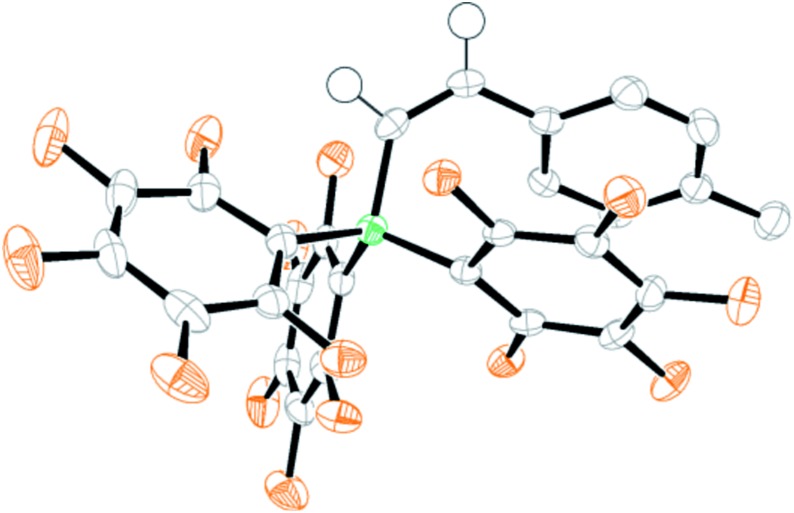
ORTEP depiction of [**14a**]^–^ (thermal ellipsoids at 50%).

Analysis of the low quantity of ionic by-products formed in the catalytic *trans*-hydroboration provided further insight into the minor processes operating alongside borenium mediated *trans*-hydroboration. Along with boronium salt **13** an additional C_6_F_5_ containing species was observed (by ^19^F NMR spectroscopy) in the pentane insoluble fraction post *trans*-hydroboration of terminal alkynes. The second ionic by-product displayed ^19^F signals at –132.5, –164.5 and –167.3 ppm consistent with a [(C_6_F_5_)_3_B-vinyl]^–^ formulation.^29^ Recrystallization of the ionic by-products from borenium mediated *trans*-hydroboration of 4-ethynyltoluene unambiguously identified this species as [(C_6_F_5_)_3_B-*Z*-(4-methylstyrene)]^–^ [**14a**]^–^ (Fig. 3). In this structure the anion is partnered with [1,3-dimethyl-4,5-dichloro imidazolium]^+^ presumably formed from trace adventitious moisture. Examination (by ^11^B and ^19^F NMR spectroscopy) of the crude *trans*-hydroboration reaction mixtures pre-workup revealed anion [**14a**]^–^ was present as a minor product during the borenium mediated *trans*-hydroboration of **10a–10d** and **10h–10k**.

**Scheme 7:**
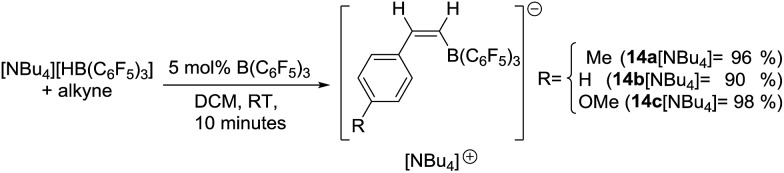
Alkyne *trans*-hydroboration with B(C_6_F_5_)_3_/[HB(C_6_F_5_)_3_]^–^.

It was hypothesised that [**14a**]^–^ forms during borenium mediated *trans*-hydroboration when B(C_6_F_5_)_3_ (present after transfer of hydride from [HB(C_6_F_5_)_3_]^–^) activates the alkyne instead of reacting with **8**. The B(C_6_F_5_)_3_ activated alkyne is then quenched by subsequent hydride transfer from **8** or [HB(C_6_F_5_)_3_]^–^ to generate [**14a**]^–^. This is related to the reactivity of B(C_6_F_5_)_3_/Lewis base FLPs which undergo 1,2-addition reactions with alkynes;^16^,^30^ in this case the nucleophile is [HB(C_6_F_5_)_3_]^–^ or **8**. 1,1-Carboboration products can be formed from the reaction of B(C_6_F_5_)_3_ and terminal alkynes but no resonances consistent with these products are observed.^31^ This indicates quenching the B(C_6_F_5_)_3_ activated alkyne by intermolecular hydride transfer is more rapid than intramolecular hydride migration and 1,1-carboboration. To confirm this hypothesis [NBu_4_][HB(C_6_F_5_)_3_] was reacted with 4-ethynyltoluene in the presence of 5 mol% B(C_6_F_5_)_3_ to give [**14a**][NBu_4_] which could be isolated in 96% yield. The product shows a single ^11^B signal at –16.8 ppm in the NMR spectrum, along with 2 new vinyl resonances at 6.70 (d, ^3^*J*_HH_ = 14.55 Hz) and 6.65 (d, ^3^*J*_HH_ = 14.92 Hz) in the ^1^H NMR spectrum fully consistent with a *cis*-vinylborane. The scope of B(C_6_F_5_)_3_ catalysed *trans*-hydroboration was explored with terminal alkynes such as phenylacetylene and 4-ethynylanisole reacting rapidly to give [**14b**]^–^ and [**14c**]^–^, respectively (Scheme 7). However, less nucleophilic terminal alkynes such as 4-(trifluoromethyl)-phenylacetylene and internal alkynes such as 3-hexyne did not undergo *trans*-hydroboration (even after heating to 100 °C for extended periods). Nevertheless, the use of an inexpensive borane/borohydride combination to achieve transition metal free *trans*-hydroboration is unprecedented to the best of our knowledge.

## Conclusions

In conclusion, highly Lewis acidic boranes containing low migratory aptitude substituents (chelated alkyls or C_6_F_5_ groups) enable the transition metal-free *trans*-hydroboration of alkynes in the presence of an appropriate hydroborane or borohydride. Using a borenium cation *trans*-hydroboration is applicable to a range of aryl (containing electron donating and withdrawing groups), heteroaryl and vinyl substituted terminal alkynes, exclusively generating *Z*-vinylborane isomers in excellent yields. Mechanistically the reaction does not involve intramolecular hydride transfer steps and is thus applicable to internal alkynes (albeit with limited scope currently). In both borenium cation and B(C_6_F_5_)_3_ mediated *trans*-hydroboration the *Z*-vinylborane products are readily isolated as air stable solids and for the former the utility of these products in cross coupling has been demonstrated. Work is ongoing to expand the scope of the *trans*-elementoboration methodology by rational borocation modification, as well as to probe the range of neutral boranes capable of alkyne activation and *trans*-elementoboration.

## Supplementary Material

Supplementary information

Crystal structure data
