# Supplementary material for: Highly selective catalytic trans-hydroboration of alkynes mediated by borenium cations and B(C6F5)3
Source: Chem Sci. 2016 Feb 12;7(5):3384–9. doi: 10.1039/c5sc04798f (PMC6006955; doi:10.1039/c5sc04798f)
Supplement: Supplementary file 1 [file SC-007-C5SC04798F-s001.pdf]

Electronic Supplementary Material (ESI) for Chemical Science.
This journal is © The Royal Society of Chemistry 2016

Supporting information

Highly Selective Catalytic Trans-Hydroboration of Alkynes mediated by Borenium Cations.

John S. McGough, Samuel M. Butler, Ian A. Cade, Michael J. Ingleson*

Contents

General Considerations ... 4

Synthesis of 9-BBN(H)(Quinuclidine) (**1**). ... 7

Synthesis of 9-BBN(1-pentyne)(Quinuclidine) (**2**). ... 8

Synthesis of [9-BBN(Quinuclidine)][B(C_6F_5)$_4$] ... 9

Synthesis of **2** *via* a frustrated Lewis pair. ... 10

Synthesis of 9-BBN(H)(IMe$_4$) (**3**) ... 11

Synthesis of 9-BBN(I)(IMe4) (**4**). ... 12

Synthesis of 9-BBN(1-pentyne)(IMe$_4$) (**5**). ... 13

Synthesis of [9-BBN(IMe$_4$)][HB(C_6F_5)$_3$] (**6**) ... 14

Hydroboration of 1-pentyne to generate 9-BBN(IMe$_4$)(*Z*-1-pentene) **7a.** ... 15

Hydroboration of 1-pentyne and addition of IMe$_4$ to give 9-BBN(IMe$_4$)(*E*-1-pentene) **7b.** ... 16

Synthesis of [9-BBN(I-DCDM)][HB(C_6F_5)$_3$] (**9**). ... 17

General procedure A, for the *trans*-hydroboration of alkynes with a borenium cation. ... 18

Spectral data for hydroborated acetylenes **10a-10n.** ... 19

Comparision of the hydroboration of per-protio and d$_1$-octyne 36

Carbene removal from **10a/c** to generate *Z*-styrenes **11a/c.** ... 38

Suzuki-Miyaura cross coupling of **11a/c** to generate *Z*-stilbenes **12a/c.** ... 41

Generation of the [9-BBN(I-DCDM)$_2$][HB(C_6F_5)$_3$] boronium (**13**). ... 43

Synthesis of [NBu$_4$][HB(C_6F_5)$_3$] ... 44

General procedure B, for the *trans*-hydroboration of alkynes using B(C_6F_5)$_3$. ... 45

Synthesis of [9-BBN(IMes)][HB(C_6F_5)$_3$] ... 49

References ... 50

Crystallographic data ... 51

Crystal structure of 9-BBN(H)(IMe$_4$) (**3**) (CCDC Number = 1429288) ... 51

Crystal structure of [9-BBN(I-Me$_4$)][HBCF] (**6**) (CCDC Number = 1429287) ... 52

Crystal structure of [9-BBN(I-DCDM)$_2$][HBCF] (**13**) CCDC Number = 1429289) ... 53

Crystal structure of 1,3-dimethyl-4,5-dichloroimidazolium [(*Z*)-(2-*para*-tolyl)ethenyl]tris(pentafluorophenyl)borate ([**14a**][**Me$_2$Cl$_2$Imid**]) (CCDC number = 1440939). ... 54

NMR Spectra: 55

9-BBN(H)(Quinuclidine) (**1**) 55

In-situ NMR spectra from the reaction of **1-H**/B(C_6F_5)$_3$ with 1-pentyne: of 9-BBN(1-pentyne)(Quinucidine) (**2**) 57

[9-BBN(Quinuclidine)][B(C_6F_5)$_4$] (**[1][B(C_6F_5)$_4$]**) 58

Synthesis of **2** *via* an FLP approach 60

9-BBN(IMe$_4$)(I) (**4**) 61

9-BBN(1-pentyne)(IMe$_4$) (**5**) / (**4**) mixture 63

Turnover of **4** after the addition of **3** and 1-pentyne 64

[9-BBN(IMe$_4$)][HB(C_6F_5)$_3$] (**6**) 65

NMR spectra of **3** and **6**. 67

NMR spectra of **8** and **9**. 68

NMR spectra of 9-BBN(H)(IMes) and [9-BBN(IMes)][HB(C_6F_5)$_3$]. 69

9-BBN(IMe$_4$)(Z-pent-1-ene) **7a** 70

9-BBN(IMe4)(E-1-pentene) **7b** 73

[9-BBN(I-DCDM)][HB(C_6F_5)$_3$] **9** 75

9-BBN(I-DCDM)(2-(*para*-tolyl)-*Z*-ethene) (**10a**) 77

9-BBN(I-DCDM)(2-(phenyl)-*Z*-ethene) (**10b**) 80

9-BBN(I-DCDM)(2-(*para*-anisole)-*Z*-ethene) (**10c**) 83

In-situ NMR spectra (with added mesitylene as internal standard) for 9-BBN(I-DCDM)(2-(*para*-(*N,N*-dimethyl)aniline)-*Z*-ethene) (**10d**) 86

9-BBN(I-DCDM)(2-(*para*-(*α,α,α*-trifluoromethyl)phenyl)-*Z*-ethene) (**10e**) 88

9-BBN(I-DCDM)(2-(*para*-chlorophenyl)-*Z*-ethene) (**10f**) 92

9-BBN(I-DCDM)(2-(*para*-fluorophenyl)-*Z*-ethene) (**10g**) 95

9-BBN(I-DCDM)(2-(*meta*-chlorophenyl)-*Z*-ethene) (**10h**) 98

9-BBN(I-DCDM)(2-(2,4,6-trimethylphenyl)-*Z*-ethene) (**10i**) 101

9-BBN(I-DCDM)(2-(2-thiophenyl)-*Z*-ethene) (**10j**) 103

9-BBN(I-DCDM)(Z-ethynyl-cyclohex-1-ene) (**10k**) 106

In-situ NMR spectra (with mesitylene as internal standard) for 1-[9-BBN(I-DCDM)]-(*Z)*-4-methylbuta-1,3-diene (**10l**) 109

Crude mixture containing 9-BBN(I-DCDM)(1-(*Z*-pent-1-ene)) as the major product 111

Crude mixture containing 9-BBN(I-DCDM)(1-(*Z*-oct-1-ene)) (**10m**$_H$) as the major new product 113

Crude mixture containing 9-BBN(I-DCDM)(1-(*Z*-oct-1-ene-d$_1$)) (**10m**$_D$) as the major new product ... 114

NMR spectra for 2-(9-BBN(I-DCDM))-3-(*para*-(*N,N*-dimethyl)aniline)-*E*-prop-2-ene) (**10n**) 115

In-Situ ^{19}F and ^{11}B NMR spectra of the hydroboration of alkynes with **8** and 5 mol% $B(C_6F_5)_3$ to give **10x** and minor ionic by-products 119

NMR spectra for the hydroboration of 4-ethynyltoluene with 1 eq **9**. 120

In-situ NMR spectra for Carbene Removal from **10a** to form **11a** and BF_3(I-DCDM) 124

In-situ NMR spectra for Carbene Removal from **10c** to form **11c** and BF_3(I-DCDM) 127

4-Methyl-4'-Fluoro-*Z*-Stilbene (**12a**) 130

4-Methoxy-4'-Fluoro-*Z*-Stilbene (**12c**) 132

[9-BBN(I-DCDM)$_2$][HB(C_6F_5)$_3$] (**13**) 134

[NBu_4][HB(C_6F_5)$_3$] 136

Tetrabutylammonium [(*Z*)-(2-*para*-tolyl)ethenyl]tris(pentafluorophenyl)borate (**14a**) 139

Tetrabutylammonium [(*Z*)-2-phenylethenyl]tris(pentafluorophenyl)borate (**14b**) 142

Tetrabutylammonium [(*Z*)-(2-*para*-methoxyphenyl)ethenyl]tris(pentafluorophenyl)borate (**14c**) ... 145

General Considerations

Unless stated, all reactions were performed under an atmosphere of argon or nitrogen using standard Schlenk and glovebox techniques. Glassware was dried in a hot oven overnight. Dichloromethane was dried by refluxing over CaH_2 followed by distillation, *ortho*-dichlorobenzene and d_2-dichloromethane were dried by stirring over calcium hydride and distilled under vacuum. Hexane was dried by refluxing over NaK alloy followed by distillation, THF was dried by refluxing over potassium metal followed by distillation, toluene and toluene-d_8 were both dried by refluxing over K metal followed by distillation, and pentane was dried by passing through an alumina drying column incorporated into an MBraun SPS800 solvent purification system. All solvents were degassed and stored over dried molecular sieves (3Å) under inert atmosphere in ampoules equipped with J. Young's taps, or in a dry argon atmosphere glovebox. Alkynes with the exception of 4-(1-propynyl)-*N.N*-dimethylaniline and 1-octyne-d_1 where purchased from commercial vendors and used as received. $B(C_6F_5)_3$ was purchased from commercial sources, and dried by stirring with Et_3SiH in pentanes or hexanes for 3 days, filtered, dried under high vacuum and purified by short path sublimation (60 ^{o}C, <9 x10^{-2} mBar, 18 hours). NMR spectra were recorded with a Bruker Avance III Cryoprobe (400 MHz ^{1}H; 100 MHz ^{13}C; 128 MHz ^{11}B; 376.50 MHz ^{19}F); a Bruker Avance II+ (500 MHz ^{1}H; 160 MHz ^{11}B; 125 MHz ^{11}C); or a Bruker Avance III (400 MHz ^{1}H; 100 MHz ^{13}C; 128 MHz ^{11}B; 376.50 MHz ^{19}F; 64.7 MHz ^{2}H). ^{1}H NMR chemical shifts are reported in ppm relative to the solvent resonance or protio impurities in the deuterated solvents and ^{13}C NMR using the solvent resonances unless otherwise stated. ^{2}H NMR are reported in ppm relative to the major deuterated solvent peak, typically DMSO-d_6. ^{11}B NMR spectra and ^{19}F NMR spectra were referenced to external BF_3:Et_2O and $CFCl_3$, respectively. Resonances for the carbon directly bonded to boron are significantly broadened in the ^{13}C{^{1}H} NMR spectra due to quadrupolar effects. In some cases we do observe clear resonances for the C directly bonded to B, though these are still extremely broad, however in other cases we do not clearly observe all directly bonded C-B resonances (despite using concentrated samples, large number of scans and a cryoprobe NMR spectrometer (to enhance signal to noise)). Water present in the DMSO-d_6 capillary as a sharp singlet a 3.94 ppm in the ^{1}H NMR spectra.

Despite repeated attempts to analyse **10a-n** by mass spectrometry under ESI, GCMS (chemical ionisation) and GCMS (electronic ionisation) conditions, no molecular ion mass peaks were seen and accurate mass analysis could not be performed. In the absence of mass spectra all relevant NMR spectra are included in this supporting information for compounds **10a-n.**

1,3,4,5-Tetramethylimidazol-2-thione,[1] IMe_4,[1] 9-BBN(H)(IMes)[2] 9-BBN(H)(I-DCDM) (**8**),[2] and IMes[3] were synthesised as per the appropriate literature reference. For reactions in J Young's valve equipped NMR tubes, stirring of the solution was achieved by mechanical rotational inversion.

Crystallographic data for **3**, **9, 11** and [**14a**][imidazolium] was recorded on an Agilent Supernova diffractometer Mo K/α radiation at λ = 0.7017 Å. CrysAlisPro software was used for data collection, cell refinement, data reduction and empirical absorption correction.[4] The structures were solved using the Superflip software,[5] and refined using Crystals,[6] and graphics generated using Ortep 3.[7] Non hydrogen atoms were refined with anisotropic displacement parameters, and hydrogens where included in calculated positions, refined using a riding model and shown with a sphere of arbitrary radius. The data for **2** showed significant disorder on the $[HB(C_6F_5)_3]^-$ anion. This was modelled as two distinct superimposed conformations with partial occupancies of 81% and 19%. This disorder is the source of some anomalously close contacts present between the minor component of the anion and the cation.

Several attempts were made to obtain accurate elemental analysis of the hydroborated compounds **10a-o**, however despite the NMR spectra indicating only the desired compound is present the percentage of C found in CHN was lower than expected (shown below for both **10a** and **10b**), presumably due to the formation of boron-carbide, and this was the case even with use of additional oxidising agent V_2O_5. Representative results of the analysis run with and without oxidiser are given below.

	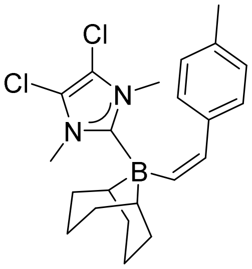 **10a**			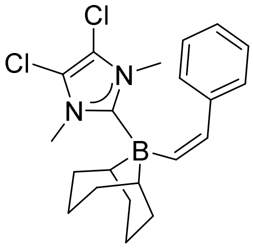 **10b**		
Element	Calculated	Found	Found with V_2O_5 added	Calculated	Found	Found with V_2O_5 added
C	65.37	61.39	61.01	64.81	62.46	62.30
H	7.48	7.75	7.66	6.99	6.91	7.09
N	6.93	6.40	6.35	7.20	7.05	7.01

Synthesis of 9-BBN(H)(Quinuclidine) (**1**).

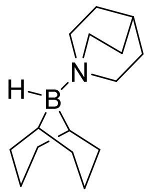

In an ampoule, 9-BBN dimer (522 mg, 2.26 mmol) and quinuclidine (503 mg, 4.52 mmol) were added. To this was added pentane (10 mL) and stirred to form a suspension. The ampoule was sealed and the reaction heated to 60 °C for 1 hour. The reaction was allowed to cool, and pentane was then removed under vacuum to give a white solid, yield 1.021g, 97%.

^{1}H NMR (400.7 MHz, *o*-DCB with a DMSO-d_6 capillary): 3.07 (t, 6H, $^{3}J_{HH}$ = 7.70 Hz), 2.32-1.84 (br, m, 14 H), 1.66 (m, 6H) 1.12 (br, s, 2H).

^{11}B NMR (128.38 MHz, *o*-DCB with a DMSO-d_6 capillary): 1.32 (br)

^{11}B{^{1}H} NMR (128.38 MHz, *o*-DCB with a DMSO-d_6 capillary): 1.20 (br)

^{13}C{^{1}H} NMR (100.61 MHz, *o*-DCB with a DMSO-d_6 capillary): 49.8, 36.9, 28.9, 25.0, 23.4, 23.3, 20.5 (br), 19.0.

Synthesis of 9-BBN(1-pentyne)(Quinuclidine) (**2**).

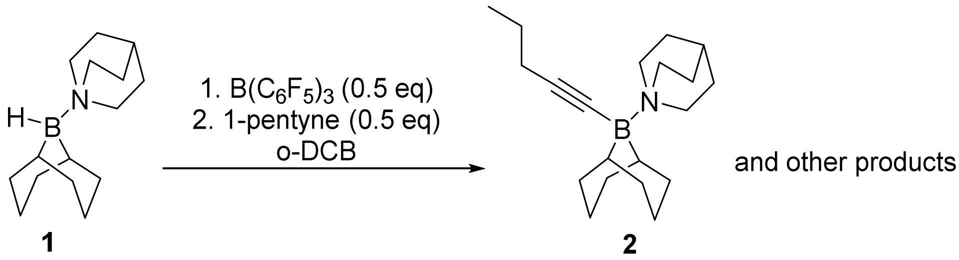

To a J Young's valve equipped NMR tube with a DMSO-d_6 capillary was added *ortho*-dichlorobenzene, (0.7 mL). To this was added 9-BBN(H)(quinuclidine) (48 mg, 0.206 mmol) and $B(C_6F_5)_3$ (52 mg, 0.103 mmol), and the reaction stirred to dissolve the solids. To this solution was added 1-pentyne (10 µL, 0.103 mmol) and the reaction was stirred for 24 hours, and monitored by NMR spectroscopy. The broad peak at 85.9 ppm in the ^{11}B NMR spectrum is due to *cis*-hydroboration of 1-pentyne by 9-BBN(H), which is slowly released during the reaction duration (peak at 57 ppm in the ^{11}B NMR spectrum see NMR spectra section).

Synthesis of [9-BBN(Quinuclidine)][$B(C_6F_5)_4$]

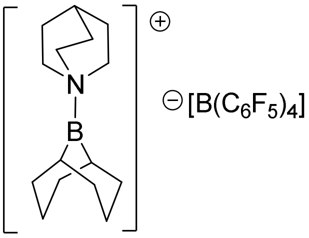

In a J Young's Valve equipped NMR tube with a DMSO-d_6 capillary, was added 9-BBN(H)(quinuclidine) (20 mg, 0.086 mmol) and *ortho*-dichlorobenzene (0.5 mL) and then stirred to dissolve. To this solution was added [Ph_3C][$B(C_6F_5)_3$] (79 mg, 0.086 mmol), and the reaction was stirred for 5 minutes, and the NMR spectra recorded. The *in-situ* NMR spectra show resonances for Ph_3CH alongside the borenium product.

^{1}H NMR (400.7 MHz, *o*-DCB with a DMSO-d_6 capillary): 3.07 (t, 6H, $^3J_{HH}$ = 7.82 Hz), 2.13-1.21 (br, overlapping multiplets, 21H).

^{11}B NMR (128.38 MHz, *o*-DCB with a DMSO-d_6 capillary): 77.4 (br, s), -17.2 (s).

^{11}B{^{1}H} NMR (128.38 MHz, *o*-DCB with a DMSO-d_6 capillary): 76.9(br, s), -17.22 (s).

^{19}F NMR (376.44 MHz, *o*-DCB with a DMSO-d_6 capillary): -131.82 (d, 8F, $^3J_{FF}$ = 21.46 Hz), -163.10 (t, 4F, $^3J_{HH}$ = 20.44 Hz), -167.05 (dt, 8F, J_{FF} = 6.8 Hz, 19.75 Hz).

Synthesis of **2** *via* a frustrated Lewis pair.

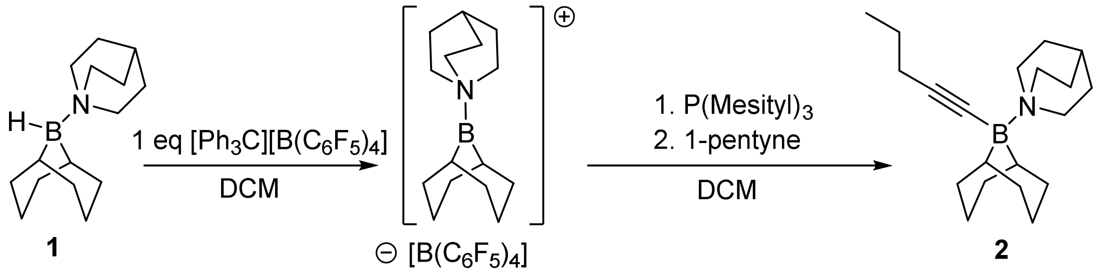

1 **2**

To a J Young's valve equipped NMR tube with a DMSO-d_6 capillary was added *ortho*-dichlorobenzene, (0.7 mL). To this was added 9-BBN(quinuclidine) (20 mg, 0.086 mmol) and $[Ph_3C][B(C_6F_5)_4]$ (79 mg, 0.086 mmol) and the solution stirred to dissolve the solids, with the formation of [9-BBN(quinuclidine)]$[B(C_6F_5)_4]$ cleanly observed by NMR spectroscopy. To this solution was added P(Mesityl)$_3$ (31 mg, 0.086 mmol), and stirred until it dissolved. No P-B bond formation is observed in the ^{11}B or ^{31}P NMR spectra at room temperature. To this solution was added 1-pentyne (8.5 µL, 0.086 mmol), and the reaction stirred and monitored by NMR spectroscopy confirming formation of **2**.

Synthesis of 9-BBN(H)(IMe_4) (**3**)

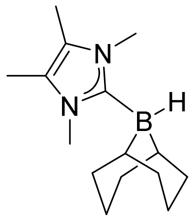

In a Schlenk equipped with a Teflon coated magnetic stirrer bar, was added 9-BBN dimer (991 mg, 4.06 mmol) and IMe_4 (1.01 g, 8.12 mmol). To this, THF (20 mL) was added and stirred to dissolve the solids. The solution was stirred for 20 hours, and then THF was removed under vacuum. The resulting solids were washed with dry pentane (2x 20 mL) and filtered to give an off-white solid, which was dried under high vacuum. Yield 1.804 g, 90.15 %. The NMR data matched well with that previously reported[2].

Synthesis of 9-BBN(I)(IMe4) (**4**).

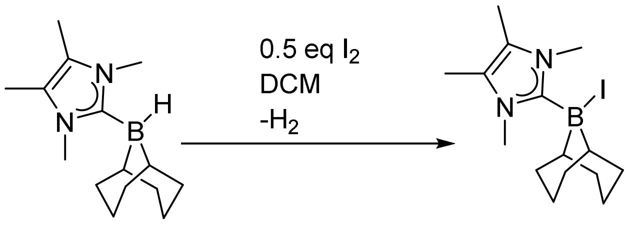

In a J Youngs valve topped NMR tube equipped with a DMSO-d_6 capillary was added DCM (0.5 mL). To this was added I_2 (16 mg, 0.063 mmol), dissolved to give a purple solution. To this was added 9-BBN(H)(IMe$_4$) (31 mg, 0.125 mmol) and stirred to dissolve, with the evolution of gas to give a clear colourless solution, and the reaction monitored by NMR spectroscopy.

^{1}H NMR (400.7 MHz, DCM, DMSO-d_6): 3.67 (s, 6H), 2.14 (s, 6H), 1.97 (m, 6H), 1.80 (m, 4H), 1.61 (m, 2H), 1.45 (m, 2H).

^{11}B NMR (128.38 MHz, DMSO-d_6): 5.47 (br, s)

^{13}C{^{1}H} NMR (100.61 MHz, DMSO-d_6): 126.2, 34.3, 32.4, 25.3 (br), 23.9, 9.3.

Synthesis of 9-BBN(1-pentyne)(IMe_4) (**5**).

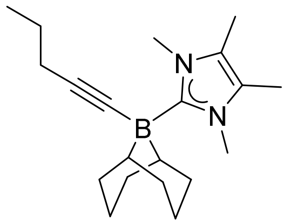

To a J Youngs valve topped NMR tube equipped with a DMSO-d_6 capillary was added DCM (0.5 mL). To this was added **3** (25 mg, 0.101 mmol) and stirred to dissolve. To this was added I_2 (0.5 eq in DCM) with the evolution of gas to give a clear solution. *In-situ* NMR spectroscopy showed complete consumption of **3** to give **4** as the only product. To this solution was added **3** (25 mg, 0.101 mmol), with ^{11}B NMR spectroscopy showing **3** and **4** as separate species in solution. To this was added 1-pentyne (10 µL, 0.101 mmol) and the mixture was stirred, with the reaction monitored by NMR spectroscopy.

After complete consumption of the alkyne (as determined by *in-situ* NMR spectroscopy), **4** was observed (ca. 50 % of NHC containing products), making an accurate integration of the aliphatic BBN region of the proton NMR unfeasible for **5**. Addition of further equivalent of 3 and 1-pentyne resulted in an increase in the resonances assigned to **5**.

^{1}H NMR (400.7 MHz, DCM, DMSO-d_6): 3.70 (s, 6H), 2.13 (s, 6H), 1.91-1.22 (br, m, 9-BBN overlapping resonances), 0.93 (t, 3H, $^3J_{HH}$ = 7.31 Hz).

^{11}B NMR (128.38 MHz, DMSO-d_6): -19.4 (s).

This compound was only produced *in-situ* and all relevant NMR spectra are included later in this SI.

Synthesis of [9-BBN(IMe$_4$)][HB(C$_6$F$_5$)$_3$] (**6**)

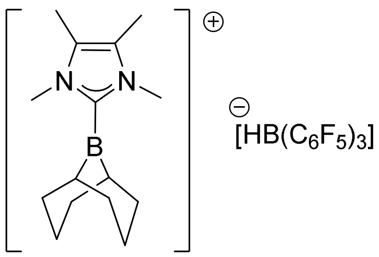

In a J Young's valve topped NMR tube equipped with a DMSO-d_6 capillary was added DCM (0.7 mL). To this was added **1** (20 mg, 0.054 mmol) which was stirred to dissolve. To this solution was added B(C$_6$F$_5$)$_3$ (33 mg, 0.054 mmol) and stirred for 30 minutes, and the reaction monitored by NMR spectroscopy. NMR data for the cation closely matches that reported for [9-BBN(IMe$_4$)][B(C$_6$F$_5$)$_4$].[2]

^{1}H NMR (400.7 MHz, DCM with a DMSO-d_6 capillary): 3.79 (s, 6H), 2.28 (s, 6H) 2.18-1.45 (br, m, 14H)

^{11}B NMR (128.38 MHz, DCM with a DMSO-d_6 capillary): 81.1 (s), -26.1 (d, $^{1}J_{BH}$ = 84.1 Hz).

^{11}B{^{1}H} NMR (128.38 MHz, DCM with a DMSO-d_6 capillary): 80.9 (s), -26.1 (s).

^{19}F NMR (376.44 MHz, DCM with a DMSO-d_6 capillary): -134.7 (d, $^{3}J_{FF}$ = 21.5 Hz), -165.1 (t, $^{3}J_{FF}$ = 20.4 Hz), -168.2 (tm, $^{3}J_{FF}$ = 23.8 Hz, 6.8 Hz, major).

^{13}C{^{1}H} NMR (100.61 MHz, DCM with a DMSO-d_6 capillary): 148.5 (br, d, $^{1}J_{CF}$ = 237.2Hz), 138.4 (vbr, d, $^{1}J_{CF}$ = 249.2 Hz), 136.7 (br, d, $^{1}J_{CF}$ = 245.5 Hz), 131.9, 34.2, 34.0, 32.8, 22.7, 8.7

Hydroboration of 1-pentyne to generate 9-BBN(IMe_4)(*Z*-1-pentene) **7a.**

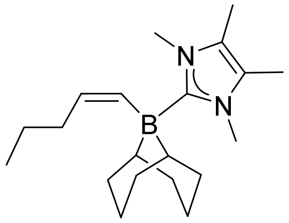

In J Young's valve equipped NMR tube was added DCM-d_2 (0.7 mL). To this was added **3** (32 mg, 0.13 mmol) followed by $B(C_6F_5)_3$ (22 mg 0.065 mmol), and the reaction stirred for 1 hour to dissolve all the solids. To the solution was added 1-pentyne (6.5 µL, 0.065 mmol), and the reaction stirred at room temperature for 6 days, and the reaction progress monitored by NMR spectroscopy. Traces of **3**, 9-BBN(H), [9-BBN(IMe_4)$_2$][HBCF] and cis-hydroborated 1-pentyne are seen in the NMR spectra alongside the major resonances attributable to **7a**. The proton resonances for the 9-BBN backbone of **7a** could not be accurately integrated due to the overlap of these signals with those from9-BBN(H), **3**, and [9-BBN(IMe)$_4$][HBCF].

^{1}H NMR (400.7 MHz, CD_2Cl_2): 6.03 (d, 1H, $^3J_{HH}$ = 13.33 Hz), 5.54 (pseudo dt, 1H, $^3J_{HH}$ = 6.11 Hz, 13.21 Hz), 3.64 (s, 6H), 2.09 (s, 6H), 1.87 – 1.23 (br, m, 9-BBN backbone), 1.03 (sextet, 2H, $^3J_{HH}$ = 7.40 Hz), 0.68 (t, 3H, $^3J_{HH}$ = 7.34 Hz).

^{11}B NMR (128.38 MHz, CD_2Cl_2): -16.05 (s).

The cis configuration of the product is confirmed by the magnitude of the coupling constants and NOE spectroscopy (see section 2).

Hydroboration of 1-pentyne and addition of IMe_4 to give 9-BBN(IMe_4)(*E*-1-pentene) **7b.**

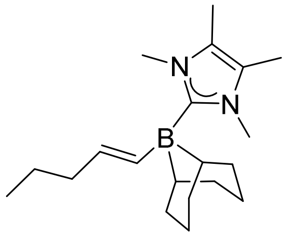

In J Young's valve equipped NMR tube with a DMSO-d_6 capillary was added DCM (0.7 mL). To this was added 9-BBN (50 mg, 0.123 mmol) and the reaction mixture was stirred to dissolve the solids. To this solution was added 1-pentyne (40 µL, 0.246 mmol) and the reaction was stirred at room temperature for 2 days, and the reaction progress monitored by NMR spectroscopy. The solution was cannula transferred to an ampoule, and the solvent was removed *in vacuo*. The solids were taken up in *o*-DCB (3 mL), and to this solution free IMe_4 (51 mg, 0.246 mmol) was then added, and the reaction allowed to stir overnight. The solvent was then removed under vacuum , and the solids were re-dissolved in DCM (0.7 mL) and cannula transferred to a J Young's valve equipped NMR tube with a DMSO-d_6 capillary, and the NMR spectra of the products recorded. The species 1,1-(9-BBN(IMe_4))$_2$-pentane from over reduction of the alkyne (alkenes are reported to hydroborate more rapidly than alkynes with BBN) can also be seen in the NMR spectra (and proved intractable in our hands), making accurate integration of the 9-BBN backbone of **7b** unfeasible.

^{1}H NMR (400.7 MHz, DCM with a DMSO-d_6 capillary): 6.01 (d, 1H, $^3J_{HH}$ = 17.36 Hz), 4.74 (doublet of triplets, 1H, $^3J_{HH}$ = 17.48 Hz, 6.36 Hz), 3.58 (s, 6H), 2.04 (s, 6H), 1.93-1.19 (br, m), 0.82 (t, 3H, $^3J_{HH}$ = 6.90 Hz).

^{11}B NMR (128.38 MHz, DCM with a DMSO-d_6 capillary): -14.9 (s).

Synthesis of [9-BBN(I-DCDM)][HB(C_6F_5)$_3$] (**9**).

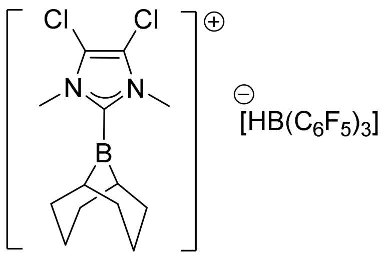

In a J Young's valve equipped NMR tube with a DMSO d_6 capillary was added DCM (0.5 mL). To this was added **8** (35 mg, 0.122 mmol) and B(C_6F_5)$_3$ (62 mg, 0.122 mmol). This was stirred at ambient temperature for 5 minutes, and the reaction monitored by NMR spectroscopy. Resonances for the cation are closely comparable to that previously reported for the B(C_6F_5)$_4$ salt.[2]

^{1}H NMR (400.7 MHz, DCM with a DMSO-d_6 capillary): 3.95 (s, 6H), 2.23-1.87 (m, 12H), 1.45 (m, 2H).

^{11}B NMR (128.38 MHz, DCM with a DMSO-d_6 capillary): 80.9 (br, s), -26.5 (br, s).

^{11}B{^{1}H} NMR (128.38 MHz, DCM with a DMSO-d_6 capillary): 81.0 (br, s), -26.2 (s).

^{19}F NMR (376.44 MHz, DCM with a DMSO-d_6 capillary): -134.8 (d, 2F, $^3J_{FF}$ = 22.1 Hz), -164.9 (t, 1F, $^3J_{FF}$ = 20.4 Hz), -168.1 (dt, 2F, $^3J_{FF}$ = 20.1 Hz, 23.5 Hz).

^{13}C{^{1}H} NMR (100.61 MHz, DCM with a DMSO-d_6 capillary): 148.3 (br, d, $^1J_{CF}$ = 236.2 Hz), 138.2 (br, d, $^1J_{CF}$ = 241.4 Hz), 136.7 (br, d, $^1J_{CF}$ = 243.6 Hz), 124.1, 35.8, 34.3, 33.3 (br), 22.6.

General procedure A, for the *trans*-hydroboration of alkynes with a borenium cation.

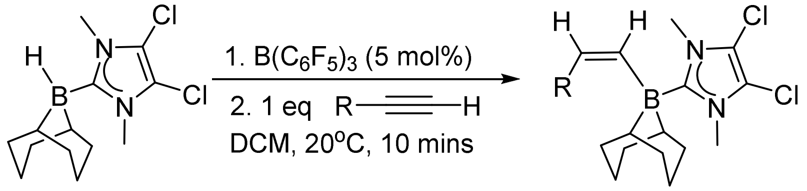

8 **10a-l**

To a J Young's valve equipped NMR tube with a DMSO-d_6 capillary was added DCM (0.5 mL). To this was added **8** (68 mg, 0.237 mmol) and $B(C_6F_5)_3$ (6 mg, 0.012 mmol), before stirring to dissolve the solids. To this solution **alkyne** was added in 1 portion, and the NMR spectra rapidly taken. The reaction progress was followed by NMR spectroscopy, and after the completion of the reaction, the solution was decanted into a conical flask open to air. The reaction vessel was washed with bench grade pentane (3 x 3 ml) and combined with the DCM solution, before the further addition of pentane (to a total of 75 mL), with the formation of a white precipitate containing minor quantities of ionic by-products. The solution was filtered and the solvents removed *in-vacuo* to give an oil, and further drying *in-vacuo* gave the hydroborated compound as a solid. NMR spectroscopy was performed on either $CDCl_3$ or C_6D_6 solutions of the product, dependant on the clarity of resonances in the aryl and vinyl regions.

Spectral data for hydroborated acetylenes **10a-10n.**

9-BBN(I-DCDM)(2-(*para*-tolyl)-*Z*-ethene) (**10a**)

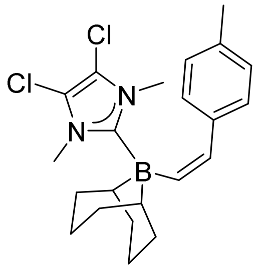

General procedure A, where 4-ethynylytoluene (27 µL, 0.226 mmol) was added as the alkyne. Yield 87 mg, 96%.

^{1}H NMR (400.7 MHz, $CDCl_3$): 6.90 (d, 2H, $^{3}J_{HH}$ = 7.58 Hz), 6.79 (d, 1H, $^{3}J_{HH}$ = 14.43 Hz), 6.69 (d, 2H, $^{3}J_{HH}$ = 7.58 Hz), 6.62 (d, 1H, $^{3}J_{HH}$ = 14.43 Hz), 3.51 (s, 6H), 2.27 (s, 3H), 2.20 (m, 1H), 1.98-1.55 (br, m, 8H), 1.40 (m, 2H), 1.28-1.17 (br, m, 3H).

^{11}B NMR (128.38 MHz, $CDCl_3$): -14.9 (s)

^{11}B{^{1}H} NMR (128.38 MHz, $CDCl_3$): -14.9 (s)

^{13}C{^{1}H} NMR (100.61 MHz, $CDCl_3$): 153.1 (br), 140.6, 134.4, 131.7, 127.6, 127.0, 116.4, 34.7, 32.4, 25.2 (br), 24.3, 23.3, 21.0.

9-BBN(I-DCDM)(2-(phenyl)-*Z*-ethene) (**10b**)

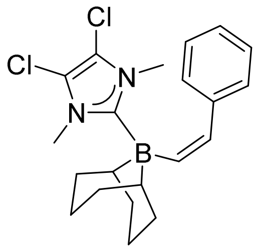

General procedure A, where phenylacetylene (25 µL, 0.228 mmol) was added as the alkyne. Yield 82 mg, 94%.

^{1}H NMR (400.7 MHz, C_6D_6): 7.05 (d, 1H, $^{3}J_{HH}$ = 14.31 Hz), 6.96 (d, 1H, $^{3}J_{HH}$ = 14.43 Hz), 6.82 (m, 3H), 6.73 (m, 2H), 2.97 (s, 6H), 2.69 (m, 2H), 2.47-2.00 (m, 6H), 1.56 (br, m, 3H), 1.35 (br, s, 3H).

^{11}B NMR (128.38 MHz, C_6D_6): -14.8 (s)

^{11}B{^{1}H} NMR (128.38 MHz, C_6D_6): -14.8 (s)

^{13}C{^{1}H} NMR (100.61 MHz, C_6D_6): 154.3 (br), 144.6, 132.7, 128.0, 125.3, 116.7, 34.8, 33.4, 32.8, 26.4 (br), 25.5, 24.6.

9-BBN(I-DCDM)(2-(*para*-anisole)-*Z*-ethene) (**10c**)

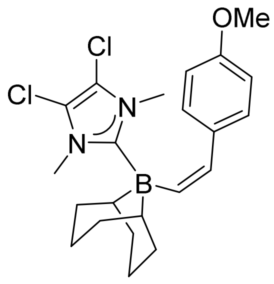

General procedure A, where 4-ethynylanisole (31 µL, 0.239 mmol) was added as the alkyne. Yield 93 mg, 97%. The ^{13}C NMR spectrum was recorded in $CDCl_3$ to avoid signal overlap between the solvent and aromatic resonances.

^{1}H NMR (400.7 MHz, C_6D_6): 7.03 (d, 1H, $^3J_{HH}$ = 14.31 Hz), 6.91 (d, 1H, $^3J_{HH}$ = 14.43 Hz), 6.65 (d, 2H, $^3J_{HH}$ = 8.07 Hz), 6.47 (d, 2H, $^3J_{HH}$ = 8.56 Hz), 3.33 (s, 3H), 3.04 (s, 6H), 2.69 (m, 2H), 2.45-2.00 (br, m, 7H), 1.61-1.21 (m, 5H).

^{11}B NMR (128.38 MHz, C_6D_6): -14.7 (s)

^{11}B{^{1}H} NMR (128.38 MHz, C_6D_6): -14.7 (s)

^{13}C{^{1}H} NMR (100.61 MHz, $CDCl_3$): 175.2 (br, Carbene C), 157.2, 153.4 (br), 136.2, 131.4, 128.2, 116.3, 112.4, 55.2, 34.7, 32.4, 31.8, 24.3, 23.3.

9-BBN(I-DCDM)(2-(*para*-(*N,N*-dimethyl)aniline)-*Z*-ethene) (**10d**)

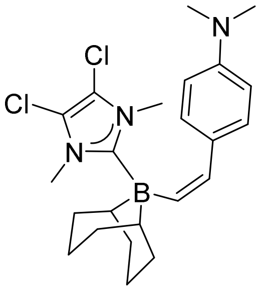

General procedure A, where 4-ethynyl-*N,N*-dimethylaniline (33 mg, 0.227 mmol) was used as the alkyne. After work up, traces of alkyne, **8**, 4-(dimethylamino)-styrene (from protodeborylation), and 9-BBN(OR) (identity of R is not known) could also be seen, making accurate integration of the 9-BBN backbone of **10d** unfeasible. Conversion (by ^{1}H NMR integration against mesitylene (10 µL) as an internal standard) 70%. Proton and 2D-noesy NMR were re-recorded in deuterated solvent to improve the signal to noise ration and to better clarify the aromatic and vinyl resonances.

^{1}H NMR (400.7 MHz, DCM, DMSO-d_6 capillary): 6.71 (d, 1H, $^{3}J_{HH}$ = 14.31 Hz), 6.65 (d, 2H, $^{3}J_{HH}$ = 8.31 Hz), 6.52 (d, 1H, $^{3}J_{HH}$ = 14.31 Hz), 6.26 (d, 2H, $^{3}J_{HH}$ = 8.44 Hz), 3.53 (s, 6H), 2.86 (s, 6H), 2.20-1.16 (br, m, 9-BBN backbone).

^{11}B NMR (128.38 MHz, DCM, DMSO-d_6 capillary): -15.6 (s)

^{11}B{^{1}H} NMR (128.38 MHz DCM, DMSO-d_6 capillary): -15.6 (s)

9-BBN(I-DCDM)(2-(*para*-(*α,α,α*-trifluoromethyl)phenyl)-*Z*-ethene) (**10e**)

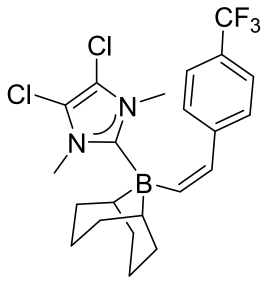

General procedure A, where 4-ethynyl-1-(α,α,α-trifluoromethyl)benzene (39 μL, 0.239 mmol) was added as the alkyne. Yield 98 mg, 94%. ^{1}H NMR spectra were -recorded in C_6D_6 to resolve the aryl and vinyl proton coupling, and to reduce second order effects.

^{1}H NMR (400.7 MHz, C_6D_6): 7.05 (d, 2H, $^{3}J_{HH}$ = 7.95 Hz), 6.98 (d, 1H, $^{3}J_{HH}$ = 14.79 Hz), 6.83 (d, 1H, $^{3}J_{HH}$ = 14.79 Hz), 6.59 (d, 2H, $^{3}J_{HH}$ = 7.95 Hz), 2.89 (s, 6H), 2.62 (m, 2H), 2.41-1.92 (m, 7 H), 1.78-1.20 (m, 5H).

^{11}B NMR (128.38 MHz, $CDCl_3$): -14.9 (s)

^{11}B{^{1}H} NMR (128.38 MHz, $CDCl_3$): -14.9 (s)

^{19}F NMR (376.44 MHz, $CDCl_3$): -62.02(s)

^{13}C{^{1}H} NMR (100.61 MHz, $CDCl_3$):155.52 (br), 147.7, 130.4, 127.5, 127.3 (q, $^{2}J_{CF}$ = 32.3 Hz), 124.4 (q, $^{1}J_{CF}$ = 272.2 Hz) 123.9 (q, $^{3}J_{CF}$ = 7.3 Hz), 116.5, 34.8, 32.2, 31.7, 25.7 (br) 24.2, 23.2.

9-BBN(I-DCDM)(2-(*para*-chlorophenyl)-*Z*-ethene) (**10f**)

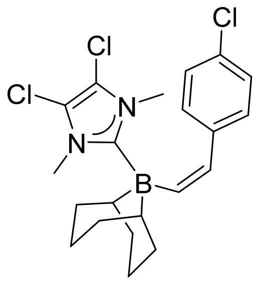

General procedure A, where *para*-chlorophenylacetylene (33 mg, 0.242 mmol) was used as the alkyne. Yield 85 mg, 87 %. NMR spectra were initially recorded in $CDCl_3$, however aryl and vinyl resonances were found to be coincident with each other. The effect was lessened in C_6D_6 and the signals could be resolved, with partial overlap of 1 vinyl doublet with an aryl proton resonance.

^{1}H NMR (400.7 MHz, C_6D_6): 6.94 (d, 1H, $^3J_{HH}$ = 14.56 Hz), 6.84 (two overlapped doublets, 3H), 6.47 (d, 2H, $^3J_{HH}$ = 8.03 Hz), 2.91 (s, 6H), 2.64 (m, 2H), 2.38 (m, 1H), 2.27-1.96 (br, m, 6H), 1.52 (br, m, 3H), 1.27 (br, s, 2H).

^{11}B NMR (128.38 MHz, C_6D_6): -14.8 (s)

^{11}B{^{1}H} NMR (128.38 MHz, C_6D_6): -14.8 (s)

^{13}C{^{1}H} NMR (100.61 MHz, C_6D_6): 155.6 (br), 142.8, 131.3, 129.3, 127.6, 116.7, 34.8, 33.3, 26.4 (br), 25.4, 24.41.

9-BBN(I-DCDM)(2-(*para*-fluorophenyl)-*Z*-ethene) (**10g**)

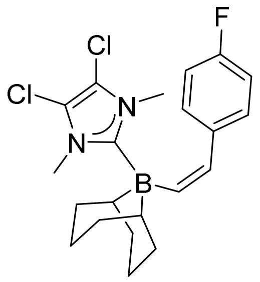

General procedure A, where *para*-fluoroethynylbenzene (29 mg, 0.241 mmol) was used as the alkyne. Yield 79 mg, 85%.

^{1}H NMR (400.7 MHz, C_6D_6): 6.93 (d, 1H, $^{3}J_{HH}$ = 14.56 Hz), 6.88 (d, 1H, $^{3}J_{HH}$ = 14.56 Hz) 6.53 (d, 4H, $^{3}J_{HH}$ = 7.78 Hz), 2.95 (s, 6H), 2.61 (m, 2H), 2.33 (m, 1 H), 2.18 (m, 1H), 2.06-1.96 (m, 5H), 1.52 (m, 3H), 1.28 br, s, 2H).

^{11}B NMR (128.38 MHz, C_6D_6): -14.8 (s).

^{11}B{^{1}H} NMR (128.38 MHz, C_6D_6): -14.9 (s).

^{19}F NMR (376.44 MHz, C_6D_6): 118.10 (s).

^{13}C{^{1}H} NMR (100.61 MHz, C_6D_6): 160.8 (d, $^{1}J_{CF}$ = 243.6 Hz), 139.6, 130.8, 128.7 (d, $^{3}J_{CF}$ = 7.3 Hz), 116.1, 113.6 (d, $^{2}J_{CF}$ = 20.5 Hz), 34.1, 32.6, 32.1, 29.9, 25.6 (br), 24.7, 23.7.

Due to the close proximity of the vinyl proton resonances, NOE cross peaks could not be clearly identified, and stereochemical assignment is made on the basis of coupling constants and by analogy to other hydroborated compounds.

9-BBN(I-DCDM)(2-(*meta*-chlorophenyl)-*Z*-ethene) (**10h**)

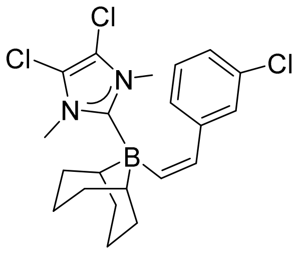

General procedure A, where 3-chloro-1-ethynylbenzene (28 µL, 0.227 mmol) was used as the alkyne. Yield 75 mg, 74 %. Overlap of the 1 vinyl doublet with an aromatic multiplet is observed, but the vinyl coupling constant could still be measured.

^{1}H NMR (400.7 MHz, C_6D_6): 6.94 (d, 1H, $^{3}J_{HH}$ = 14.67 Hz), 6.82 (overlapped aromatic multiplet and vinyl doublet, 2H, $^{3}J_{HH}$ = 14.79 Hz), 6.72 (s, 1H), 6.55 (m, 2H), 3.01 (s, 6H), 2.60 (m, 2H), 2.43-1.94 (br, m, 7H), 1.55-1.25 (m, 5H).

^{11}B NMR (128.38 MHz, C_6D_6): -14.8 (s)

^{11}B{^{1}H} NMR (128.38 MHz, C_6D_6): -14.8 (s)

^{13}C{^{1}H} NMR (100.61 MHz, C_6D_6): 156.0 (br), 146.7, 133.7, 131.1, 128.9, 127.9, 126.3, 125.4, 117.0, 34.9, 33.3, 32.8, 26.4, 25.4, 24.4.

9-BBN(I-DCDM)(2-(2,4,6-trimethylphenyl)-*Z*-ethene) (**10i**)

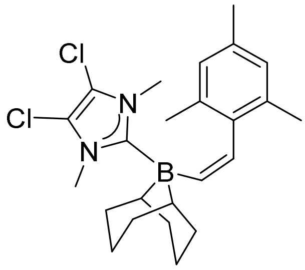

General procedure A, where 2-ethynyl,-1,3,5,-trimethylbenzene (37 µL, 0.236 mmol) was used as the alkyne. Yield 81 mg, 84 %. Mesityl -CH_3 proton resonances overlap with the 9-BBN back bone and are integrated alongside each other. Due to the close proximity of the vinyl proton resonances, cross peaks in the NOESY spectrum could not be adequately resolved, and stereochemistry is assigned on the basis of the magnitude of the coupling constants and by reference to the other *trans*-hydroborated compounds herein.

^{1}H NMR (400.7 MHz, C_6D_6): 6.84 (d, 1H, $^3J_{HH}$ = 14.79 Hz), 6.76 (d, 1H, $^3J_{HH}$ = 14.92 Hz), 6.54 (s, 2H), 2.87 (s, 6H), 2.63 (m, 2H, 9-BBN back bone), 2.41-1.89 (m, 18 H, 9-BBN back bone and mesityl CH_3 resonances), 1.58-1.31 (m, 4H, 9-BBN backbone).

^{11}B NMR (128.38 MHz, C_6D_6): -14.9 (s)

^{11}B{^{1}H} NMR (128.38 MHz, C_6D_6): -14.9 (s)

^{13}C{^{1}H} NMR (100.61 MHz, C_6D_6): 139.8, 136.4, 134.8, 132.5, 127.9, 116.8, 34.4, 33.4, 32.6, 25.6, 25.1, 21.2, 20.5.

9-BBN(I-DCDM)(2-(2-thiophenyl)-*Z*-ethene) (**10j**)

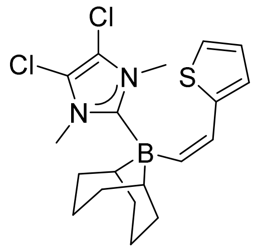

General procedure A, where 2-ethynylthiophene (26 mg, 0.241 mmol) was used as the alkyne. Yield 73 mg, 91%.

^{1}H NMR (400.7 MHz, $CDCl_3$): 6.98 (d, 1H, $^{3}J_{HH}$ = 5.01 Hz), 6.80 (d, 1H, $^{3}J_{HH}$ = 14.31 Hz), 6.77 (m, 1H), 6.60 (d, 1H, $^{3}J_{HH}$ = 14.06 Hz), 6.41 (m, 1H), 3.55 (s, 6H), 2.15 (m, 2H), 1.98-1.77 (br, m, 2H), 1.67-1.14 (br, m, 10H).

^{11}B NMR (128.38 MHz, $CDCl_3$): -14.9 (s)

^{11}B{^{1}H} NMR (128.38 MHz, $CDCl_3$): -14.9 (s)

^{13}C{^{1}H} NMR (100.61 MHz, $CDCl_3$): 175.2 (v br), 159.1 (br), 145.9, 126.0, 123.3, 123.2, 123.0, 116.6, 34.9, 32.3, 31.8, 25.7 (br), 24.3, 23.3.

9-BBN(I-DCDM)(Z-ethynyl-cyclohex-1-ene) (**10k**)

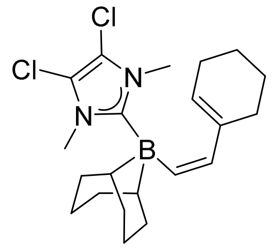

General procedure A, where 1-ethynyl-cyclohexene (27 µL, 0.242 mmol) was used as the alkyne. After standard work up, the product was dissolved in pentane (30 mL) and filtered through a short (<1cm) plug of silica, before being dried under vacuum. Yield 78 mg, 88 %.

^{1}H NMR (400.7 MHz, $CDCl_3$): 6.25 (d, 1H, $^{3}J_{HH}$ = 14.43 Hz), 5.99 (d, 1H, $^{3}J_{HH}$ = 14.31 Hz), 5.10 (m, 1H), 3.76 (s, 6H), 2.13 (m, 2H), 1.94-1.81 (m, 4H), 1.68-1.15 (m, 16H).

^{11}B NMR (128.38 MHz, $CDCl_3$): -15.0 (s)

^{11}B{^{1}H} NMR (128.38 MHz, $CDCl_3$): -14.9 (s)

^{13}C{^{1}H} NMR (100.61 MHz, $CDCl_3$): 139.9, 134.9, 119.4, 116.5, 35.1, 32.5, 31.8, 28.9, 25.4, 24.4, 23.2, 22.8, 22.2.

1-[9-BBN(I-DCDM)]-(*Z)*-4-methylbuta-1,3-diene (**10l**)

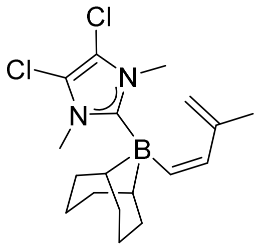

To a J Young's valve equipped NMR tube with a DMSO-d_6 capillary was added DCM (0.5 mL). To this was added **8** (68 mg, 0.237 mmol) and $B(C_6F_5)_3$ (12 mg, 0.023 mmol), before stirring to dissolve the solids. To this solution 2-methylbuten-3-yne (21 µL, 0.220 mmol) was added in 1 portion, and the NMR spectra taken after 5 minutes which showed conversion to the desired product. Yield (vs. mesitylene as an internal ^{1}H NMR standard) = 56%.

The product is unstable in solution with new products being observed on standing and rapid work up under ambient conditions also leads to the growth of other by-product, thus any future conversion would be need to be performed in-situ.. Resonances for the unknown by products / subsequent reactions are seen in the ^{1}H NMR spectrum, making integration of the 9-BBN rings unfeasible. These minor products at short reaction time also precluded the listing of an accurate ^{13}C{^{1}H} NMR spectrum.

^{1}H NMR (400.7 MHz, DCM, DMSO-d_6 capillary): 6.25 (d, 2H, $^3J_{HH}$ = 14.43 Hz), 6.06 (d, 2H, $^3J_{HH}$ = 14.06 Hz), 4.40 (br, s, 1H), 4.31 (br, s, 1H), 3.72 (s, 6H), 2.08-1.15 (br, m, overlapping 9-BBN and methyl protons).

^{11}B NMR (128.38 MHz, DCM, DMSO-d_6 capillary): -15.6 (s)

^{11}B{^{1}H} NMR (128.38 MHz, DCM, DMSO-d_6 capillary): -15.6 (s).

9-BBN(I-DCDM)(1-(*Z*-pent-1-ene))

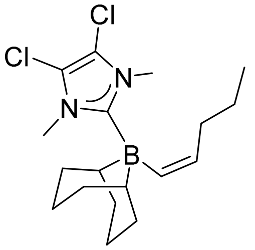

To a J Young's valve equipped NMR tube was added DCM-d_2 (0.7 mL). To this was added **8** (50 mg, 0.17 mmol) and $B(C_6F_5)_3$ (9 mg, 0.018 mmol), before stirring to dissolve the solids. To this solution 1-pentyne (15 µL, 0.152 mmol) was added, and the reaction progress monitored by NMR spectroscopy. The spectrum shows signals for the desired product, as well as **8** and **13**.

^{1}H NMR (400.7 MHz, CD_2Cl_2): 5.96 (d, 1H, $^3J_{HH}$ = 13.33 Hz), 5.59 (pseudo quintet, d of t, 1H, $^3J_{HH}$ = 6.60, 13.20 Hz), 3.81 (s, 6H), 2.04-1.23 (br, m), 0.73 (t, 3H, $^3J_{HH}$ = 7.34 Hz).

^{11}B NMR (128.38 MHz, CD_2Cl_2): -15.9 (s)

^{11}B{^{1}H} NMR (128.38 MHz, CD_2Cl_2): -15.9 (s)

9-BBN(I-DCDM)(1-(*Z*-oct-1-ene)) (**10m$_H$**)

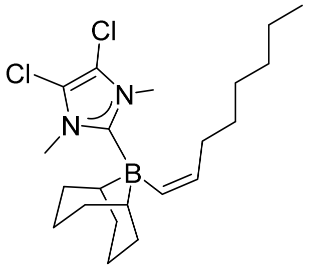

To a J Young's valve equipped NMR tube DCM-d_2 (0.7 mL). To this was added **8** (50 mg, 0.17 mmol) and $B(C_6F_5)_3$ (9 mg, 0.018 mmol), before stirring to dissolve the solids. To this solution 1-octyne (15 µL, 0.152 mmol) was added, and the reaction progress monitored by NMR spectroscopy. On completion the solution was cannula transferred to a Schlenk, and the volatiles removed *in vacuo*, to give a white solid. This was dried under vacuum, then pentane (5 mL) was added, and stirred. This suspension was then filtered, and the pentane soluble fraction was dried under vacuum to give a pale oil. This was dissolved in dry $CDCl_3$, cannula transferred to a J Young's valve topped NMR tube and the NMR spectrum recorded. Signals for 9-BBN(H), 9BBN(OR) (where the identity of R is not known), **8** and imidazolium species (presumably from the decomposition of **8** by trace water) are also seen in the NMR spectrum, and due to this accurate integration of the 9-BBN backbone of **10m$_H$** could not be achieved.

^{1}H NMR (400.7 MHz, $CDCl_3$): 5.95 (d, 1H, $^3J_{HH}$ = 13.20 Hz), 5.61 (d of t, 1H, $^3J_{HH}$ = 6.48, 13.20 Hz), 3.81 (s, 6H), 2.40 (m), 2.02-1.07 (br, m), 0.85 (m).

^{11}B NMR (128.38 MHz, $CDCl_3$): -14.7 (s)

^{11}B{^{1}H} NMR (128.38 MHz, $CDCl_3$): -14.8(s)

1H NMR between 5.40 and 6.40 ppm: the minor peak at 5.99 ppm is taken to be one of the alkene protons from isomerisation reactions, which overlaps with the resonance for **$10m_H$**.

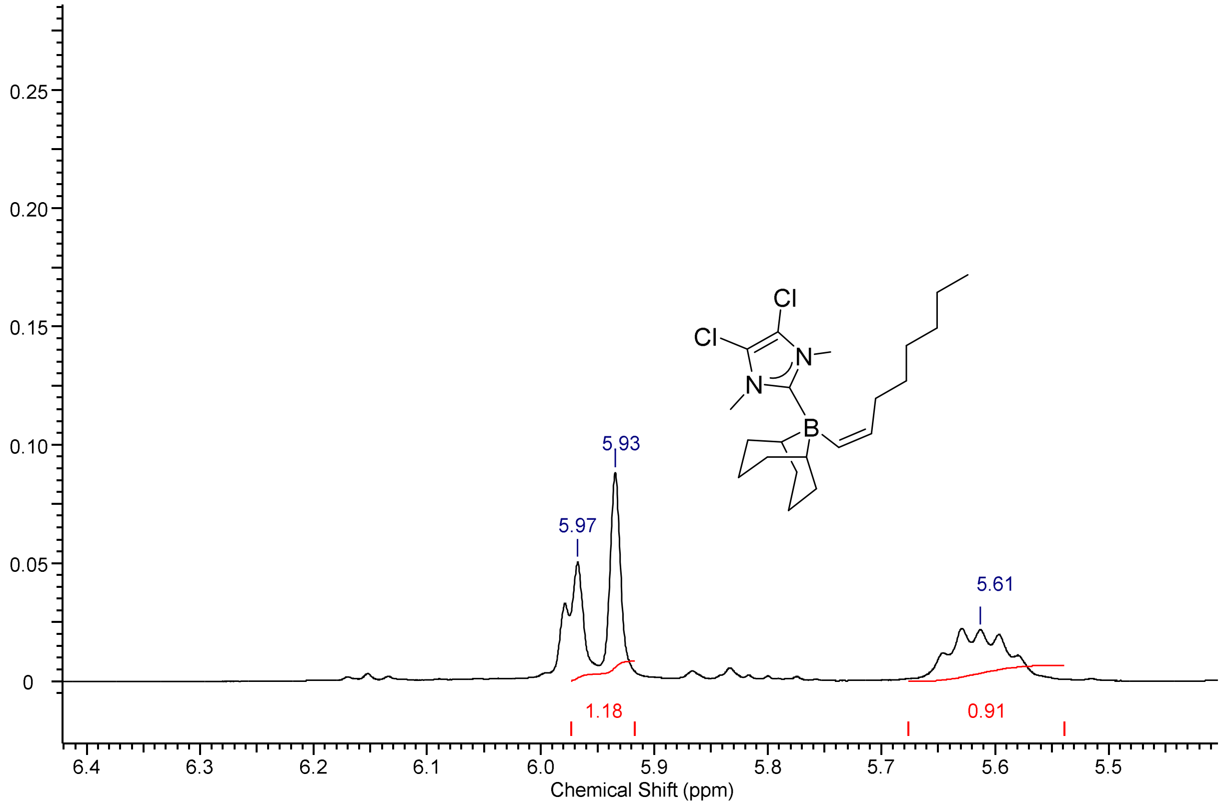

9-BBN(I-DCDM)(1-(*Z*-oct-1-ene-d_1)) (**10m**$_{\boldsymbol{D}}$)

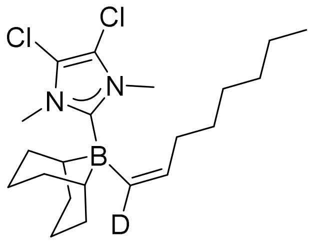

To a J Young's valve equipped NMR tube was added DCM-d_2 (0.7 mL). To this was added **8** (50 mg, 0.17 mmol) and $B(C_6F_5)_3$ (9 mg, 0.018 mmol), before stirring to dissolve the solids. To this solution 1-octyne-d_1 (15 µL, 0.152 mmol) was added, and the reaction progress monitored by NMR spectroscopy. On completion the solution was cannula transferred to a Schlenk, and the volatiles removed *in vacuo*, to give a white solid. This was dried under vacuum, then pentane (5 mL) was added, and stirred. This suspension was then filtered, and the pentane soluble fraction was dried under vacuum to give a pale oil. This was dissolved in dry $CDCl_3$, cannula transferred to a J Young's valve topped NMR tube and the NMR spectrum recorded. The ^{2}H NMR spectrum were recorded in *protio*-DCM with a DMSO-d_6 capillary, to allow to best resolution of the signals. Signals for 9-BBN(H), **8** and imidazolium species from the decomposition of **8** by trace water are also seen in the NMR spectrum. Accurate integration of the 9-BBN backbone of **10m**$_{\mathbf{D}}$ was not feasible due to the presence of multiple 9-BBN containing species.

When the reaction was repeated in *protio*-DCM, signals for the formation of **10m**$_{\mathbf{D}}$ are seen *in-situ* including a ^{2}H NMR signal at 5.38 ppm. Upon work up and redissolving in $CHCl_3$, the ^{2}H NMR signal shifts to 5.44 ppm (vs. DMSO), precluding it from being CD_2Cl_2 (either residual or natural abundance).

^{1}H NMR (400.7 MHz, $CDCl_3$): 5.62 (t, 1H, $^3J_{HH}$ = 5.62 Hz), 3.81 (s, 6H), 2.02-1.07 (br, m), 0.85 (m).

^{2}H NMR (61.41 MHz, DCM with a DMSO-d_6 capillary): 5.37 (br, s).

^{11}B NMR (128.38 MHz, $CDCl_3$): -14.8 (s)

^{11}B{^{1}H} NMR (128.38 MHz, $CDCl_3$): -14.9 (s)

1H NMR between 5.40 and 6.40 ppm, ($CDCl_3$): The peak at 5.7 ppm is an unknown impurity.

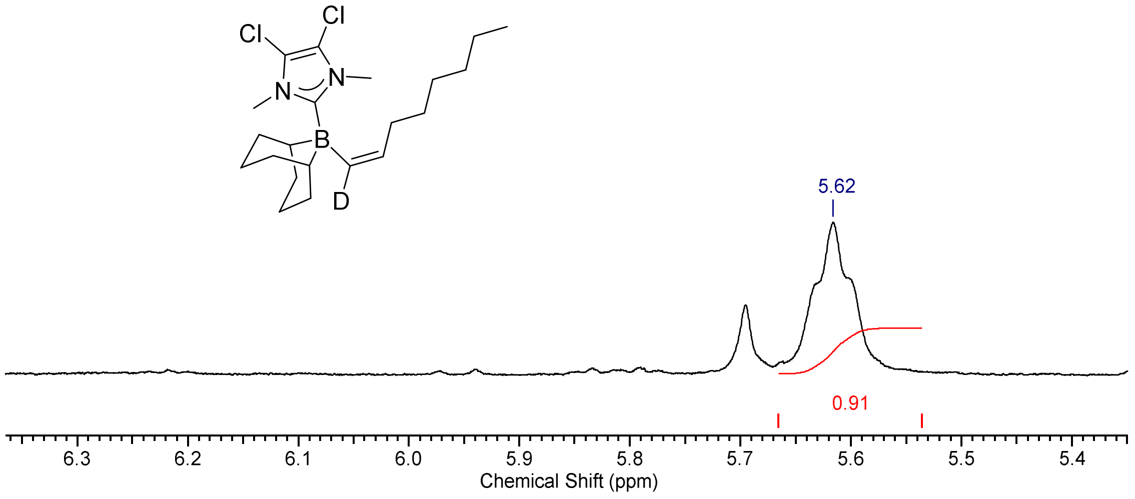

^{2}H NMR between 0 and 8 ppm, (DCM, DMSO-d_6 capillary):

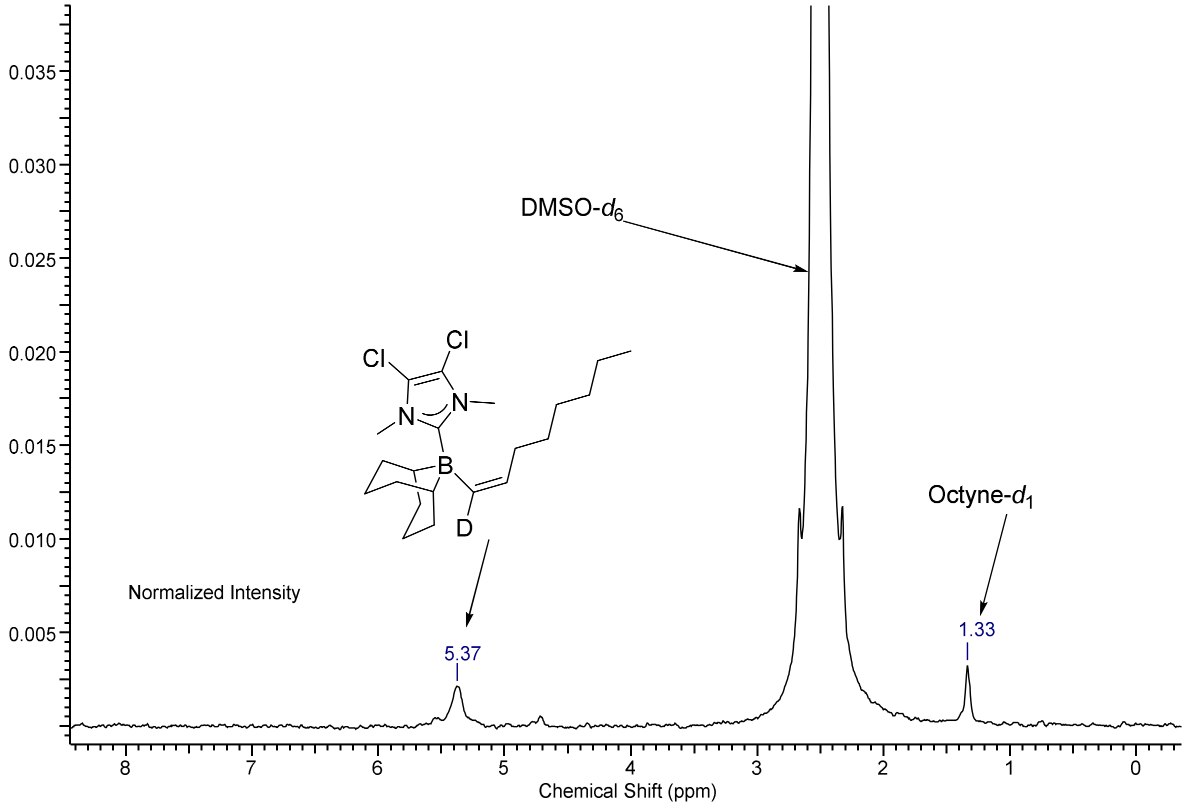

^{2}H NMR between 0 and 8 ppm, (CHCl$_3$, DMSO-d_6 capillary):

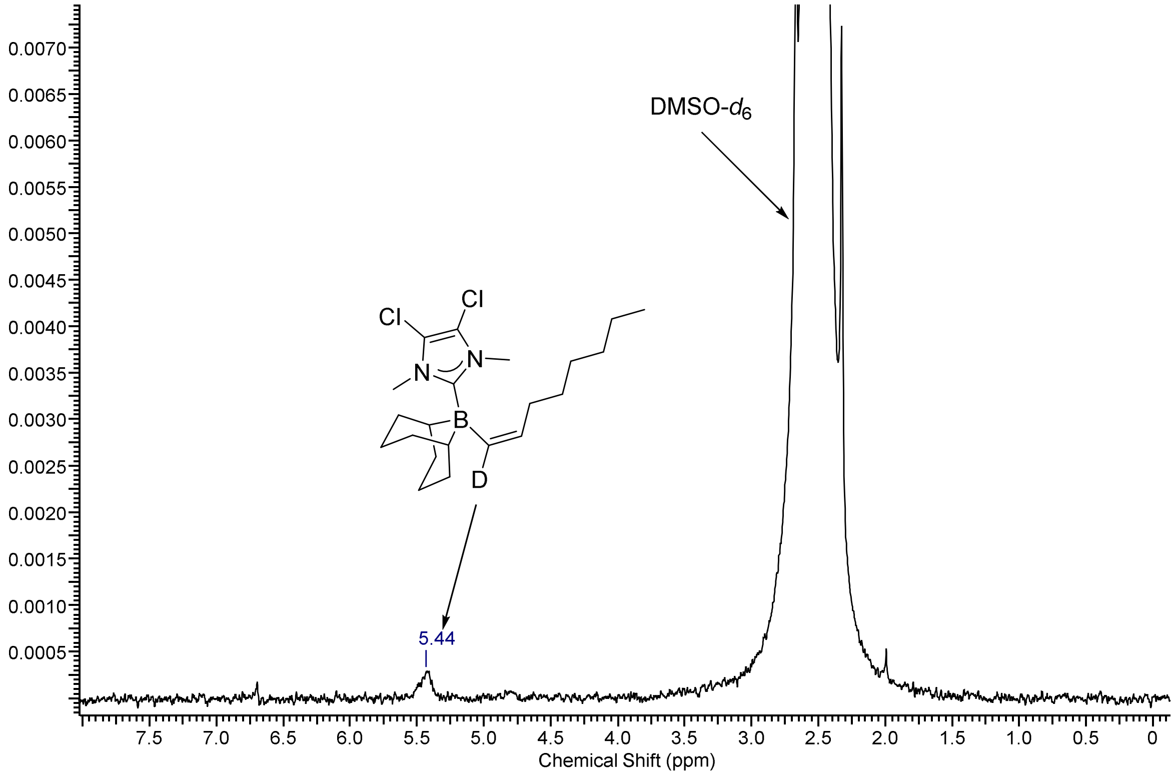

10m$_H$ and **10m$_D$** comparison stack plot in CDCl$_3$, between 5.1 ppm and 7.3 ppm.

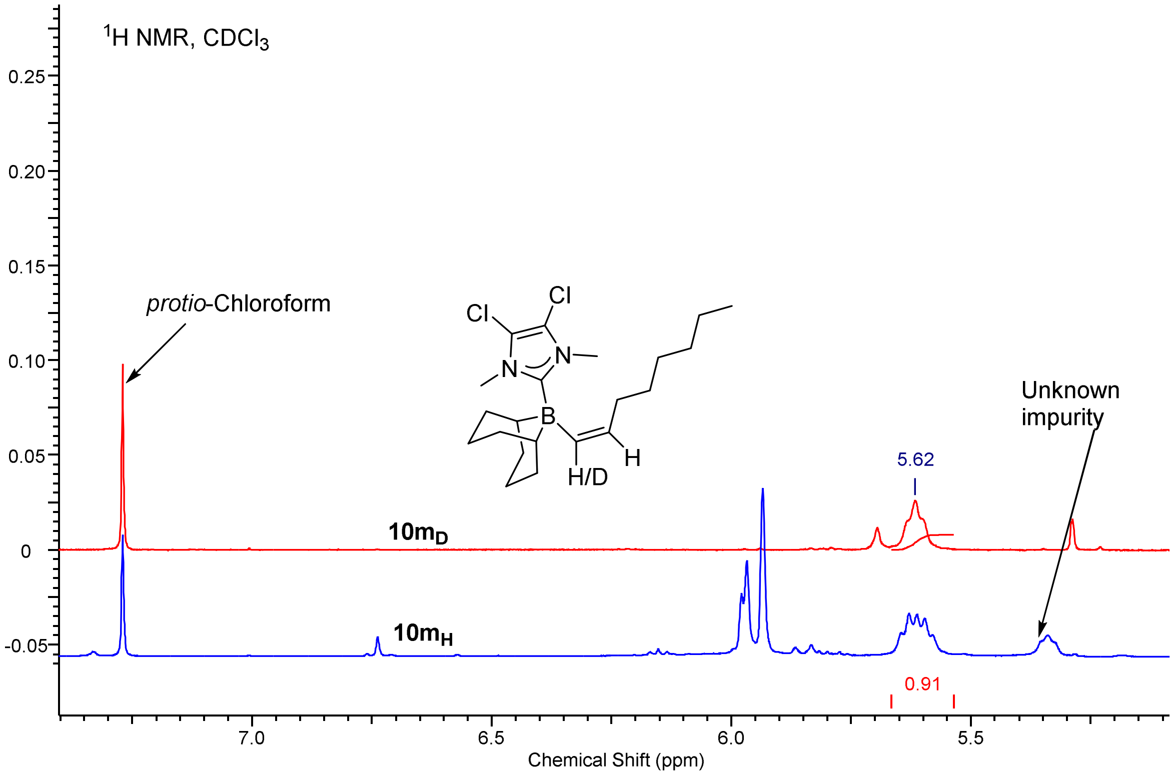

2-(9-BBN(I-DCDM))-3-(*para*-(*N,N*-dimethyl)aniline)-*E*-prop-2-ene) (**10n**)

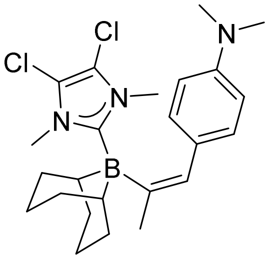

To a J Young's valve equipped NMR tube with a DMSO-d_6 capillary was added DCM (0.5 mL). To this was added **8** (68 mg, 0.237 mmol) and $B(C_6F_5)_3$ (12 mg, 0.024 mmol), before stirring to dissolve the solids. To this 4-(1-propynyl)-*N,N*-dimethylaniline (37 mg, 0.232 mmol) was added, and the NMR spectra rapidly taken after brief stirring. After 3 hours the solution was decanted into a conical flask open to air. The reaction vessel was washed with bench grade pentane (3x 3 ml) and combined with the DCM solution, before the further addition of pentane (75 mL), with the formation of a white precipitate. The solution was filtered and the solvents removed *in vacuo* to give an oil, and the NMR spectra recorded in C_6D_6. Conversion as measured by ^{1}H NMR integration 70%. Signals for 4-(1-propynyl)-*N,N*-dimethylaniline, **8** and 9-BBN(OR) (where the identity of R is unknown) are also observed in the NMR spectra, and due to this, accurate integration of the 9-BBN backbone of **10n** in the 1H NMR spectrum was not carried out.

^{1}H NMR (400.7 MHz, C_6D_6): 6.68 (br s, 1H), 6.64 (d, 2H, $^3J_{HH}$ = 8.68 Hz), 6.32 (d, 2H, $^3J_{HH}$ =8.68 Hz), 2.91 (s, 6H), 2.50 (s, 6H), 2.47 (d, 3H, $^4J_{HH}$ = 1.47 Hz), 2.16-1.88 (br, m), 1.78-1.29 (br, m).

^{11}B NMR (128.38 MHz, C_6D_6): -12.6 (s)

^{11}B{^{1}H} NMR (128.38 MHz, C_6D_6): -12.6 (s)

Hydroboration of 4-ethynyltoluene with stoichiometric **9**:

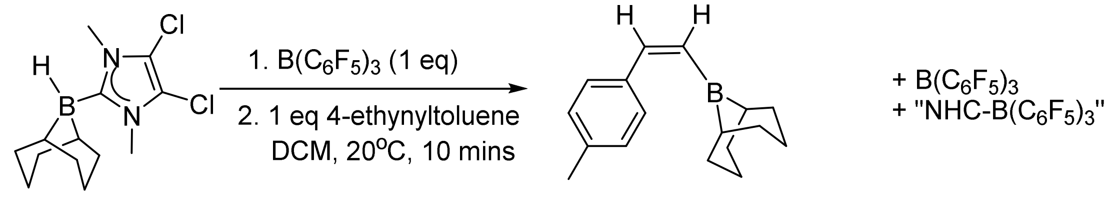

8

To a J Youngs valve topped NMR tube equipped with a DMSO-d_6 capillary was added DCM (0.5 mL). To this was added **8** (23 mg, 0.08 mmol) and $B(C_6F_5)_3$ (41 mg, 0.08 mmol), and the reaction mixture was stirred to dissolve the solids, and the formation of **9** confirmed by NMR spectroscopy. Following this, 4-ethynyltoluene (10.5 µL, 0.08 mmol) was added, and the reaction monitored by NMR spectroscopy. Major products from the reaction are Z-(vinyl)-9BBN complex formed from trans-hydroboration and then NHC dissociation (by comparison with other cis-vinylBR$_2$ complexes particularly based upon the 15 Hz $^3J_{HH}$ coupling constant observed for the vinylic protons) and a complex tentatively assigned as (NHC)$B(C_6F_5)_3$ from transfer of a carbene during hydroboration.[12] $B(C_6F_5)_3$ is observed as the other fluorine containing species.

Carbene removal from **10a/c** to generate *Z*-styrenes **11a/c.**

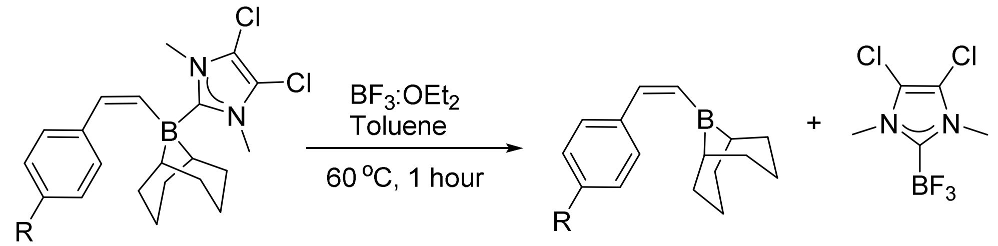

10a R = CH$_3$
10c R = OMe

11a R = CH$_3$
11c R = OMe

General procedure:

In a J Young's valve equipped NMR tube, **10a/c** was dissolved in toluene-d_8 (0.7 mL). To this solution BF_3:OEt_2 was added. The tube was sealed and heated at 60 °C in an oil bath for 1 hour. The reaction was monitored by NMR spectroscopy, and showed quantitative conversion of **10a/c** after 1 h. Signals for Et_2O are seen in the ^{1}H NMR spectrum, as well as traces of residual BF_3:OEt_2 in the ^{11}B and ^{19}F NMR spectra. ^{13}C NMR spectra for **11a,c** and I-DCDM:BF_3 were recorded *in-situ* as a mix of products, and signals are assigned based on comparison between the two.

Z-9-*(*4-methylstyryl)-9-BBN (**11a**):

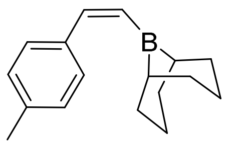

As per the general procedure, using **10a** (100 mg, 0.248 mmol) and BF_3:OEt_2 (35 µL, 0.276 mmol).

^{1}**H NMR** (400.7 MHz, C_7D_8): 7.20 (d, 1H, $^{3}J_{HH}$ = 13.94 Hz), 6.96 (d, 2H, $^{3}J_{HH}$ = 8.07), 6.92 (d, 2H, $^{3}J_{HH}$ = 7.82 Hz), 6.45 (d, 1H, $^{3}J_{HH}$ = 13.94 Hz), 2.09 (s, *p*-Me coincident with residual *protio*- solvent resonance for toluene isotopomers), 1.96-1.76 (br, m, 12 H), 1.40 (m, 2H).

^{11}**B NMR** (128.38 MHz, C_7D_8): 83.5 (br, s)

^{11}**B{^{1}H} NMR** (128.38 MHz, C_7D_8): 83.3 (br, s)

^{13}**C{^{1}H} NMR** (100.61 MHz, C_7D_8): 143.9, 130.2, 129.4, 126.7, 24.2, 21.5, 15.5

Z-9-(4-methoxystyryl)-9-BBN (**11c**):

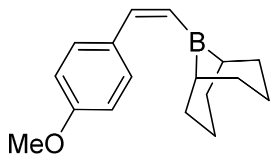

As per the general procedure, using **10c** (104 mg, 0.248 mmol) and BF_3:OEt_2 (35 µL, 0.276 mmol).

^{1}H NMR (400.7 MHz, C_7D_8): 7.09 (d, 1H, $^{3}J_{HH}$ = 13.96 Hz), 6.87 (d, 2H $^{3}J_{HH}$ = 8.44 Hz), 6.58 (d, 2H, $^{3}J_{HH}$ = 8.56 Hz), 6.26 (d, 1H, $^{3}J_{HH}$ = 13.82 Hz), 3.19 (s, 3H), 1.83-1.58 (m, 12H), 1.25 (m, 2H)

^{11}B NMR (128.38 MHz, C_7D_8): 82.6 (br, s)

^{11}B{^{1}H} NMR (128.38 MHz, C_7D_8): 83.2 (br, s)

^{13}C{^{1}H} NMR (100.61 MHz, C_7D_8): 160.7, 144.4, 133.1, 130.7, 114.0, 55.1, 24.2, 14.7.

B-(1,3-dimethyl-4,5-dichloroimidazolylidiene)trifluoroborate :

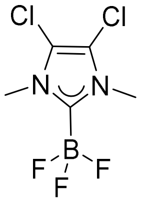

^{1}H NMR (400.7 MHz, C_7D_8): 3.16 (s, 6H)

^{11}B NMR (128.38 MHz, C_7D_8): -0.26 (quartet, $^{1}J_{BF}$ = 32.7 Hz)

^{11}B{^{1}H} NMR (128.38 MHz, C_7D_8): -0.26 (quartet, $^{1}J_{BF}$ = 32.7 Hz)

^{19}F NMR (376.44 MHz, C_7D_8): -138.3 (quartet, $^{1}J_{BF}$ = 33.0 Hz)

^{13}C{^{1}H} NMR (100.61 MHz, C_7D_8): 35.1, 117.8

Suzuki-Miyaura cross coupling of **11a/c** to generate *Z*-stilbenes **12a/c.**

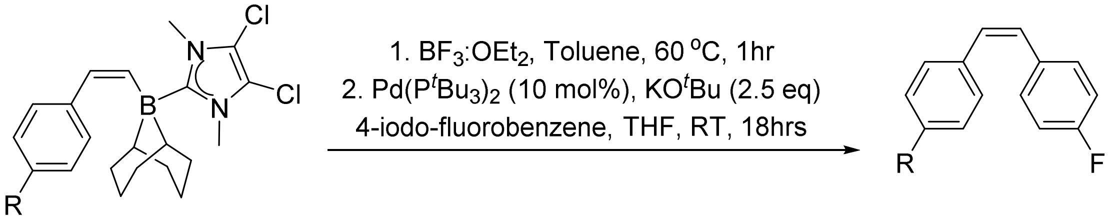

10a R = Me
10c R = OMe

12a R = Me, 81%
12c R = OMe, 73%

In an ampoule, **10a** or **10c** (0.248 mmol) was dissolved in toluene (1 mL), and to this solution BF_3:OEt_2 (35 µL, 0.276 mmol) was added. The ampoule was sealed, and the solution stirred and heated in an oil bath at 60 ^{o}C for 1 hour. The solution was allowed to cool, and the volatiles removed under vacuum. To the residue was added THF (3 mL), and the mixture stirred to dissolve all solids. This solution was then cannula transferred to a second ampoule containing KO^{t}Bu (76 mg, 0.624 mmol) and Pd(P*t*Bu$_3$)$_2$ (27 mg, 0.025 mmol) and stirred until homogeneous. To this solution, 4-iodo-fluorobenzene (26 µL, 0.225 mmol) was added before the ampoule was sealed and stirred overnight (18 hours). The reaction was quenched with the addition of saturated NH_4Cl solution (aq) (20 mL), and the products were extracted with DCM (3x 20 mL). The organic fractions were combined and dried over anhydrous magnesium sulphate, filtered and solvent removed under vacuum. The products **12a/c** was isolated by column chromatography on silica gel.

4-Methyl-4'-Fluoro-Z-Stilbene (**12a**)

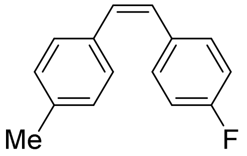

Product was isolated by column chromatography with Pentane: DCM (9:1) as the eluent. Yield 42.8 mg, 81%

^{1}H NMR: (400.7 MHz, $CDCl_3$): 7.14 (dd, 2H, $^3J_{HH}$ = 8.68 Hz, $^3J_{HF}$ = 5.50 Hz), 7.04 (d, 2H, $^3J_{HH}$ = 8.07 Hz), 6.96 (d, 2H, $^3J_{HH}$ = 7.95 Hz), 6.84 (t, 2H, $^3J_{HH}$ = 8.80 Hz), 6.48 (d, 1H, $^3J_{HH}$ = 12.23 Hz), 6.42 (d, 1H $^3J_{HH}$ = 12.23 Hz), 2.24 (s, 3H).

^{19}F NMR: (376.44 MHz, $CDCl_3$): -114.90 (m)

^{13}C{^{1}H} NMR (100.61 MHz, $CDCl_3$): 161.8 (d, $^1J_{CF}$ = 246.6 Hz), 137.0, 134.0, 133.3 (d, $^4J_{CF}$ = 3.7 Hz), 130.5 (d, $^3J_{CF}$ = 8.1 Hz), 130.2, 128.9 ($^2J_{CF}$ = 27.1 Hz), 128.4, 115.2, 115.0, 21.2.

Accurate Mass - Found: 213.1074 (M+H, APCI positive). Calculated for $C_{15}H_{14}F$ = 213.1074.

Importantly, the NMR spectra are considerably different to that reported previously for the trans isomer.[9]

4-Methoxy-4'-Fluoro-Z-Stilbene (**12c**).

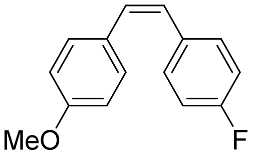

Product was isolated by column chromatography using Pentane: Ethyl Acetate (9:1) as the eluent. Yield 41.5 mg, 73%. The spectrum is fully consistent with the previously published data,[10] and is distinct from the reported values for the *E*- isomer.[11]

^{1}H NMR (400.7 MHz, $CDCl_3$): 7.48-7.44 (m, 4H), 7.04 (m, 2H), 6.98 (d, 2H, $^3J_{HH}$ = 5.77 Hz), 6.90 (d, 2H, $^3J_{HH}$ = 8.78 Hz), 3.84 (s, 3H).

^{19}F NMR (376.44 MHz, $CDCl_3$): -114.90 (m).

^{13}C{^{1}H} NMR (100.61 MHz, $CDCl_3$): 162.0 (d, $^3J_{CF}$ = 246.5 Hz), 159.3, 133.7, 129.9, 127.9 (d, $^4J_{CF}$ = 2.2 Hz), 127.7 (d, $^3J_{CF}$ = 8.1 Hz), 127.6, 125.3, 115.5 (d, $^2J_{CF}$ = 22.0 Hz), 114.1, 55.31.

Accurate Mass - Found: 229.1023 (M+H, APCI positive). Calculated for $C_{15}H_{14}OF$ = 229.1023.

Generation of the [9-BBN(I-DCDM)$_2$][HB(C_6F_5)$_3$] boronium (**13**).

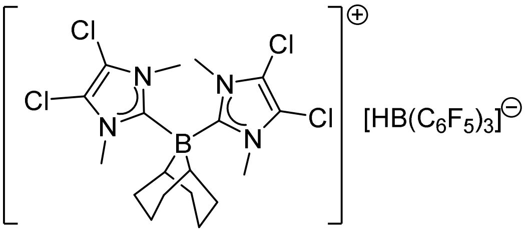

To a J.Youngs valve topped NMR tube equipped with a DMSO-d_6 capillary was added DCM (0.5 mL). To this was added **8** (35 mg, 0.122 mmol) and B(C_6F_5)$_3$ (31 mg, 0.061 mmol) before being stirred to form a solution. After stirring for several hours, *in-situ* NMR spectroscopy showed the formation of a new species in the ^{11}B spectrum at -13 ppm, along with the generation of 9-BBN(H). The solution was transferred to an ampoule and layered with hexanes to produce crystals suitable for X-Ray diffraction analysis. Subsequent to this, the solution was filtered and the crystalline material was dissolved in DCM and transferred to a J Youngs valve topped NMR tube with a DMSO-d_6 capillary, and the NMR spectra recorded. Due to the presence of imidazolium species from partial decomposition, ^{13}C{^{1}H} NMR data are not reported.

^{1}H NMR (400.7 MHz, DCM DMSO-d_6): 3.90 (s, 12H), 1.91-1.31 (m, 16H)

^{11}B NMR (128.38 MHz, DCM DMSO-d_6): -12.9 (s)

^{11}B{^{1}H} NMR (128.38 MHz, DCM DMSO-d_6): -12.9 (s)

^{19}F NMR (376.44 MHz, DCM, DMSO-d_6): 134.7 (d, 2F, $^3J_{FF}$ = 21.45 Hz), 165.8 (t, 1F, $^3J_{FF}$ = 20.10 Hz) 168.14 (dt, 2F, $^3J_{FF}$ = 20.90 Hz, $^2J_{FF}$ = 6.81 Hz)

Synthesis of $[NBu_4][HB(C_6F_5)_3]$

$$B(C_6F_5)_3 + [NBu_4][BH_4] \xrightarrow[-\ 0.5\ B_2H_6]{DCM} [NBu_4][HB(C_6F_5)_3]$$

In a Schlenk was added $[NBu_4][BH_4]$ (300 mg, 1.166 mmol), followed by $B(C_6F_5)_3$ (597 mg, 1.166 mmol). To this, DCM (20 mL) was added and the solids dissolved to give a clear colourless solution which was stirred for 30 minutes. The volatiles where then removed *in-vacuo* to give a white solid which was dried under vacuum to give the product (867 mg, 90 %).

^{1}H NMR (400.7 MHz, DCM, DMSO-d_6): 3.08 (m, 8H), 1.56 (quintet, 8H, $^3J_{HH}$ = 7.46 Hz, 8.23 Hz), 1.36 (septet, 8H, $^3J_{HH}$ = 7.34 Hz), 0.96 (t, 12H, $^3J_{HH}$ = 7.34 Hz).

^{1}H{^{11}B} NMR (400.7 MHz, DCM, DMSO-d_6): 3.57 (br, s, 1H), 3.05 (br, s, 8H), 1.57 (br, s, 8H), 1.36 (br, s, 8H), 0.96 (br, s, 12H).

^{11}B NMR (128.38 MHz, DCM, DMSO-d_6): -26.1 (d, $^1J_{BH}$ = 93.4 Hz).

^{11}B{^{1}H} NMR (128.38 MHz, DCM, DMSO-d_6):-26.1 (s).

^{19}F NMR (376.44 MHz, DCM, DMSO-d_6): -134.50 (br, d, 6F, $^3J_{FF}$ = 21.2 Hz), -165.08 (t, 3F, $^3J_{FF}$ = 20.1 Hz), -168.04 (td, 6F, $^3J_{FF}$ = 20.1 Hz, 7.2 Hz)

^{13}C{^{1}H} NMR (100.61 MHz, DCM, DMSO-d_6): 148.2 (d, $^1J_{CF}$ = 235.5 Hz), 137.9 (d, $^1J_{CF}$ = 242.8 Hz), 136.5 (d, $^1J_{CF}$ = 243.5 Hz), 59.0, 23.8, 19.7, 13.3.

General procedure B, for the *trans*-hydroboration of alkynes using $B(C_6F_5)_3$.

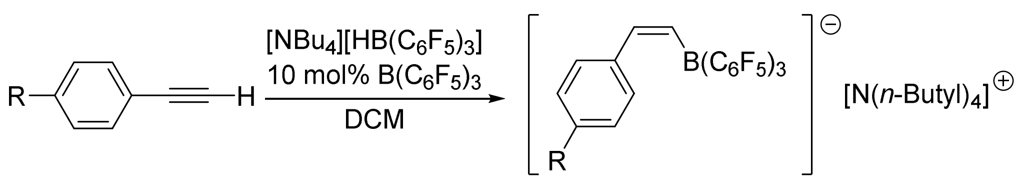

In a J Youngs valve topped NMR tube equipped with a DMSO-d_6 capillary was added DCM (0.5 mL). To this was added $[NBu_4][HB(C_6F_5)_3]$ (75 mg, 0.10 mmol) and $B(C_6F_5)_3$ (5 mg, 0.01 mmol) and the mixture stirred to give a clear colourless solution. To this solution was added *alkyne* (1.1 eq) in 1 portion, and the reaction stirred and monitored by NMR spectroscopy. After the reaction had gone to completion (typically <10 minutes), the solution was decanted into a round bottom flask and the tube washed with DCM (3x3 mL). The volatiles were removed *in vacuo*, and the solids washed with pentane (3x5 mL) before being dried under vacuum to give the pure product.

In the samples [**14a**]$^-$ - [**14c**]$^-$, the vinyl protons are highly coupled and in some cases coincident to the point where a single broad signal is observed instead of two distinct doublets. Despite attempts to resolve these by changing the polarity of the NMR solvents (spectra run in $CDCl_3$, MeCN-d_4, DMSO-d_6 and Acetone-d_6, poor solubility observed in benzene-d_6 and toluene), a distinct cross peak could not be observed in 2D NOE experiments. The stereochemistry of **14a-14c** is therefore assigned based on the crystal structure of **14a** (as the 1,3-dimethyl-4,5-dichloro imidazolium salt), and by comparison of the coupling constants with known *Z*-vinyl boranes (*vide supra*, and literature, J = 13.9-15.1 Hz).

Tetrabutylammonium [(*Z*)-(2-*para*-tolyl)ethenyl]tris(pentafluorophenyl)borate ([**14a**][**NBu$_4$**])

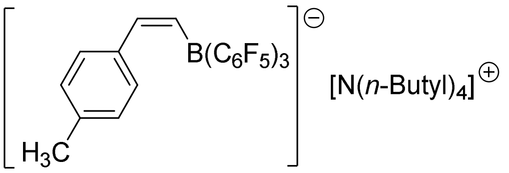

General procedure B, where 4-ethynyltoluene (14 µL, 0.107 mmol) was added as the alkyne, yield 86 mg, 96%).

^{1}H NMR (400.7 MHz, $CDCl_3$): 6.88 (d, 2H, $^{3}J_{HH}$ = 7.95 Hz), 6.78 (d, 2H, $^{3}J_{HH}$ = 7.82 Hz), 6.72-6.63 (overlapping doublets, 2H, $^{3}J_{HH}$ = 14.55 Hz, 14.92 Hz), 2.98 (m, 8H), 2.19 (s, 3H), 1.52 (quintet, 8H, $^{3}J_{HH}$ = 7.70 Hz, 8.19 Hz), 1.34 (septet, 8H, $^{3}J_{HH}$ = 7.34 Hz), 0.97 (t, 12H, $^{3}J_{HH}$ = 7.34 Hz).

^{11}B NMR (128.38 MHz, $CDCl_3$): -16.1 (s)

^{19}F NMR (376.44 MHz $CDCl_3$): -131.79 (br d, 6F, $^{3}J_{FF}$ = 19.4 Hz), -163.86 (t, 3F, $^{3}J_{FF}$ =20.4 Hz), -167.29 (td, 6F, $^{3}J_{FF}$ = 20.1 Hz, 6.1 Hz).

^{13}C{^{1}H} NMR (100.61 MHz, $CDCl_3$): 148.1 (d, $^{1}J_{CF}$ = 234.8 Hz), 138.7, 138.1 (d, $^{1}J_{CF}$ =239.9 Hz), 136.2 (d, $^{1}J_{CF}$ = 258.2 Hz), 134.1, 132.8, 127.9, 127.3, 58.6, 23.6, 20.9, 19.5, 13.4.

Accurate Mass: Found = 629.0599 (HESI negative mode), calculated for $C_{27}H_9F_{15}B$ = 629.0563.

Tetrabutylammonium [(*Z*)-2-phenylethenyl]tris(pentafluorophenyl)borate ([**14b][NBu$_4$]**)

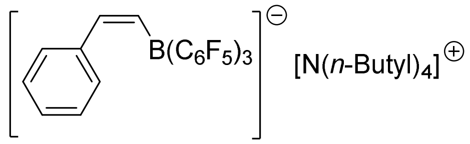

General procedure B, where phenylacetylene (12 µL, 0.109 mmol) was added as the alkyne, yield 78 mg, 90%).

^{1}H NMR (400.7 MHz, $CDCl_3$): 7.00-6.90 (m, 5H), 6.76 (d, 1H, $^{3}J_{HH}$ = 14.67 Hz), 6.67 (d, 1H, $^{3}J_{HH}$ = 14.92 Hz), 2.97 (m, 8H), 1.52 (quintet, 8H, $^{3}J_{HH}$ = 7.70 Hz, 8.07 Hz), 1.34 (septet, 8H, $^{3}J_{HH}$ = 7.34 Hz), 0.96 (t, 12H, $^{3}J_{HH}$ = 7.34 Hz).

^{11}B NMR (128.38 MHz, $CDCl_3$): -16.2 (s)

^{19}F NMR (376.44 MHz $CDCl_3$): -131.84 (br d, 6F, $^{3}J_{FF}$ = 19.4 Hz), -163.71 (t, 3F, $^{3}J_{FF}$ =20.4 Hz), -167.29 (td, 6F, $^{3}J_{FF}$ = 19.4 Hz, 5.4 Hz).

^{13}C{^{1}H} NMR (100.61 MHz, $CDCl_3$): 147.8 (d, $^{1}J_{CF}$ = 246.5 Hz), 141.3, 136.8 (d, $^{1}J_{CF}$ =240.6 Hz), 135.2 (d, $^{1}J_{CF}$ =249.4 Hz), 132.6, 127.6, 124.3, 58.3, 23.2, 19.1, 13.0.

Accurate Mass: Found = 615.0385 (HESI negative mode), calculated for $C_{26}H_7F_{15}B$ = 615.0396.

Tetrabutylammonium [(*Z*)-(2-*para*-methoxyphenyl)ethenyl]tris(pentafluorophenyl)borate ([**14c**][**NBu$_4$**])

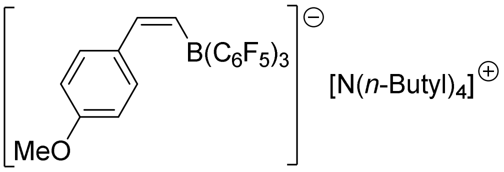

General procedure B, where 4-ethynylanisole (14 µL, 0.108 mmol) was added as the alkyne, yield 86 mg, 98%).

^{1}H NMR (400.7 MHz, $CDCl_3$): 6.93 (d, 2H, $^3J_{HH}$ = 8.68 Hz), 6.63 (br, s, 2H), 6.54 (d, 2H, $^3J_{HH}$ = 8.68 Hz), 3.70(s, 3H), 2.98 (m, 8H), 1.52 (quintet, 8H, $^3J_{HH}$ = 7.21 Hz, 8.56 Hz), 1.34 (septet, 8H, $^3J_{HH}$ = 7.34 Hz), 0.96 (t, 12H, $^3J_{HH}$ = 7.34 Hz).

^{11}B NMR (128.38 MHz, $CDCl_3$): -16.2 (s)

^{19}F NMR (376.44 MHz $CDCl_3$): -131.79 (br d, 6F, $^3J_{FF}$ = 18.7 Hz), -163.72 (t, 3F, $^3J_{FF}$ =20.8 Hz), -167.23 (td, 6F, $^3J_{FF}$ = 18.7 Hz, 6.1 Hz).

^{13}C{^{1}H} NMR (100.61 MHz, $CDCl_3$): 148.2 (d, $^1J_{CF}$ = 254.6 Hz), 138.7, 137.5 (d, $^1J_{CF}$ = 234.8 Hz), 136.0 (d, $^1J_{CF}$ =253.82 Hz), 134.1, 132.8, 127.9, 127.3, 58.6, 23.6, 20.9, 19.5, 13.4.

Accurate Mass: Found = 645.0493 (HESI negative mode), calculated for $C_{27}H_9F_{15}BO$ = 645.0512.

Synthesis of [9-BBN(IMes)][$HB(C_6F_5)_3$]

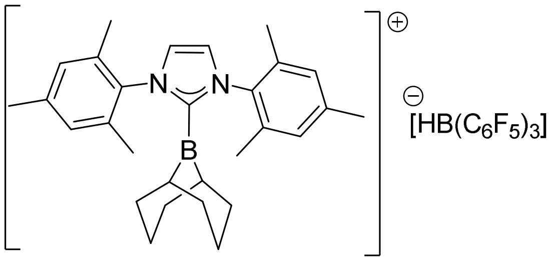

In a J Young's NMR tube valve equipped NMR tube with a DMSO d_6 capillary was added DCM (0.5 mL). To this was added 9-BBN(H)(IMes) (38 mg, 0.07 mmol), followed by $B(C_6F_5)_3$ (38 mg, 0.07 mmol), and this solution was stirred for 18 hours. Some residual 9-BBN(H)(IMes) could be seen in the NMR spectrum, making the accurate integration of the 9-BBN backbone resonances unfeasible. The spectral data for the cation is a close match with that reported for [9-BBN(IMes)][$B(C_6F_5)_4$].[2]

^{1}H NMR (400.7 MHz, DCM with a DMSO-d_6 capillary): 7.63 (s, 2H), 7.09 (s, 4H), 2.36 (s, 6H), 2.08 (s, 12H), 1.78-0.83 (br, m, 14H 9-BBN backbone).

^{11}B NMR (128.38 MHz, DCM with a DMSO-d_6 capillary): 84.4 (s), -26.1 (d, $^{1}J_{BH}$ = 88.8 Hz).

^{11}B{^{1}H} NMR (128.38 MHz, DCM with a DMSO-d_6 capillary): 84.8 (s), -26.0 (s).

^{19}F NMR (376.44 MHz, DCM with a DMSO-d_6 capillary): -134.5 (d, $^{3}J_{FF}$ = 22.1 Hz), -165.2 (t, $^{3}J_{FF}$ = 20.4 Hz), -168.2 (tm, $^{3}J_{FF}$ = 23.8 Hz, 6.8 Hz).

^{13}C{^{1}H} NMR (100.61 MHz, DCM with a DMSO-d_6 capillary): 147.2 (d, br, $^{1}J_{CF}$ = 236 Hz), 141.5, 136.4 (d, br, $^{1}J_{CF}$ = 243 Hz), 135.5 (d, br, $^{1}J_{CF}$ = 240 Hz), 133.0, 129.0, 126.4, 33.7, 21.0, 19.8, 16.0

References

(1) Kuhn, N., Kratz, T. *Synthesis* **1993**, *6*, 561.

(2) Farrell, J. M., Posaratnanathan, R.T., Stephan, D.W., *Chem. Sci.* **2015**, ***6***, 2010.

(3) Arduengo, A. J. I., Dias, H.V.R., Dias, Harlow, R.L., Kilne, M., *J. Am. Chem. Soc.* **1992**, *114*, 5530.

(4) CrysAilisPro, Aglient Technologies, 1.171.36.24, 03-12-2012

(5) Palatinus, L., Chapuis, G., *J. Appl. Cryst.* **2007**, *40*, 786.

(6) Betteridge, P. W., Carruther, J.R., Cooper, R.I., Prout, K., Watkin, D.J., *J. Appl. Cryst.* **2003**, *36*.

(7) Farrugia, L. J. *J. Appl. Cryst.* **2012**, *45*.

(8) Zhang, W., Kraft, S., Moore, J.S., *J. Am. Chem. Soc.* **2004**, *126*, 329.

(9) Yasuda, S, Yorimitsu, H., Oshima, K., *Organometallics* **2010**, *29*, 2634-2636.

(10) Shi, M., Xu, B., *J. Org. Chem.,* **2002**, *67*, 294.

(11) Tanaka, S., Mori, A., *Eur. J. Org. Chem.* **2014**, *6*, 1167.

(12) Chase P.A., Stephan, D.W., *Angew. Chem. Int. Ed.*, **2008**, *47* (39), 7433.

Crystallographic data

Crystal structure of 9-BBN(H)(IMe_4) (**3**) (CCDC Number = 1429288)

Ellipsoids shown at 50% probability, and H atoms except those bound to boron are omitted.

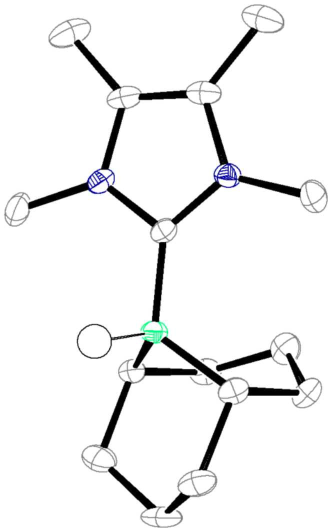

Empirical Formula.	$C_{15}HBN_{2.}$
FW g/mol.	246.20.
Crystal System and Space Group.	Monoclinic, P1 21/c 1.
Temperature (K).	150.01.
a, b, c (Å).	7.3696(5), 11.5512(8), 17.0873(9).
α, β, γ (degrees).	90, 93.641(5), 90.
Volume ($Å^3$).	1451.65(16).
Z.	4.
Radiation.	Mo *K*\α, λ = 0.7107 Å.
Absorption coefficient.	0.065.
F(000).	544.
Θ range (degrees)	2.969 – 25.340.
Number of reflections collected.	5165.
Number of unique reflections.	2652.
Number of data/ restraints/ parameters	2642, 0, 163.
R1 (data with [I^2> 2σ(I^2)]).	0.0516
wR2 (all data).	0.1277
Goodness of Fit.	0.9866
Δρ maximum and minimum (e. $Å^{-3}$).	0.50, -0.44

Crystal structure of [9-BBN(I-Me_4)][HBCF] (**6**) (CCDC Number = 1429287)

Thermal ellipsoids are shown at 50% probability, and only 1 conformation of the disordered HBCF anion is shown. Hydrogens not bound to boron are omitted.

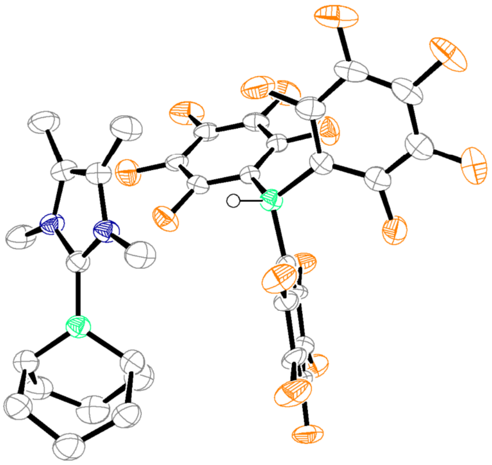

Empirical Formula	$C_{33}H_{27}B_2F_{15}N_2$
FW g/mol	758.19
Crystal System and Space Group	Monoclinic, P 21/n
Temperature (K)	151
a, b, c (Å)	10.3052(10), 33.5364(19), 10.5825(11)
α, β, γ (degrees)	90, 117.953(13), 90
Volume (Å^3)	3230.6(6)
Z	4
Radiation	Mo *K*\α, λ = 0.71073 Å.
Absorption coefficient	0.151
F(000)	1536.008
Θ range (degrees)	3.264 – 28.516
Number of reflections collected	13188
Number of unique reflections	7156
Number of data/ restraints/ parameters	7143, 228, 771
R1 (data with [I^2> 2σ(I^2)])	0.0717
wR2 (all data)	0.1613
Goodness of fit	1.0269
Δρ maximum and minimum (e. Å^{-3})	0.56, -0.45

Crystal structure of [9-BBN(I-DCDM)$_2$][HBCF] (**13**) CCDC Number = 1429289)

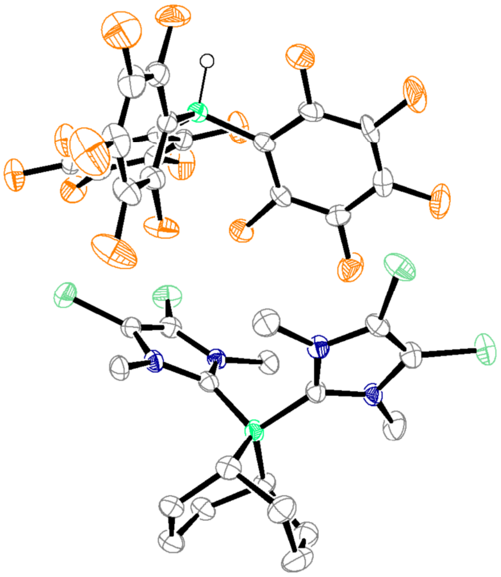

Empirical Formula	$C_{36}H_{27}B_2Cl_4F_{15}N_4$
FW g/mol	964.04
Crystal System and Space Group	Monoclinic, P 1 21 1
Temperature (K)	150.0
a, b, c (Å)	8.8757(6), 20.3411(11), 10.7976 (7)
α, β, γ (degrees)	90, 101.728(7), 90
Volume (Å^3)	1908.7(2)
Z	2
Radiation	Mo *K*\α, λ = 0.71073 Å.
Absorption coefficient	0.420
F(000)	968
Θ range (degrees)	3.374-29.127
Number of reflections collected	8992
Number of unique reflections	7031
Number of data/ restraints/ parameters	7014/ 1/ 551
R1 (data with [I^2> 2σ(I^2)])	0.0725
wR2 (all data)	0.0879
Goodness of fit	0.9775
Δρ maximum and minimum (e. Å^{-3})	0.54, -0.55

Crystal structure of 1,3-dimethyl-4,5-dichloroimidazolium [(*Z*)-(2-*para*-tolyl)ethenyl]tris(pentafluorophenyl)borate ([**14a**][**Me_2Cl_2Imid**]) (CCDC number = 1440939).

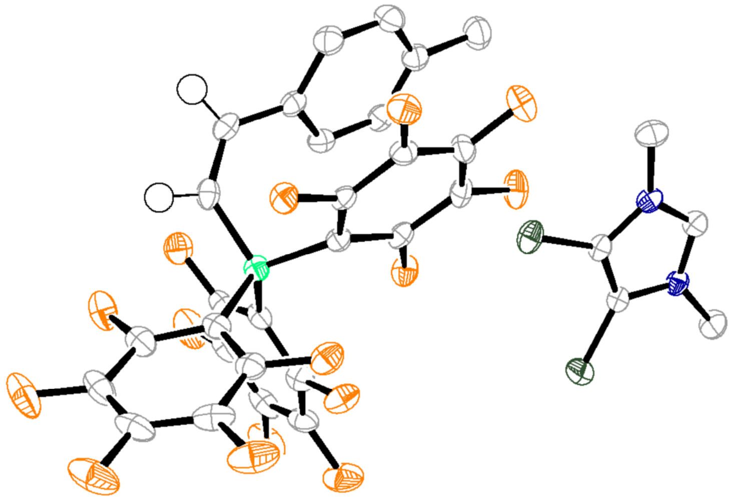

Empirical Formula	$C_{32}H_{16}F_{15}N_2Cl_2B$
FW g/mol	795.18
Crystal System and Space Group	Monoclinic, P 1 21/c 1
Temperature (K)	150.0
a, b, c (Å)	15.2641(4), 10.3890(3), 19.5887(6)
α, β, γ (degrees)	90, 92.115(3), 90
Volume ($Å^3$)	3104.23
Z	4
Radiation	Mo *K*\α, λ = 0.71073 Å.
Absorption coefficient	0.329
F(000)	1584
Θ range (degrees)	3.8530 - 28.2190
Number of reflections collected	7251
Number of unique reflections	7238
Number of data/ restraints/ parameters	7238/ 0/ 469
R1 (data with [I^2> 2σ(I^2)])	0.0487
wR2 (all data)	0.1035
Goodness of fit	0.9732
Δρ maximum and minimum (e. $Å^{-3}$)	0.60, -0.70

NMR Spectra:9-BBN(H)(Quinuclidine) (**1**)

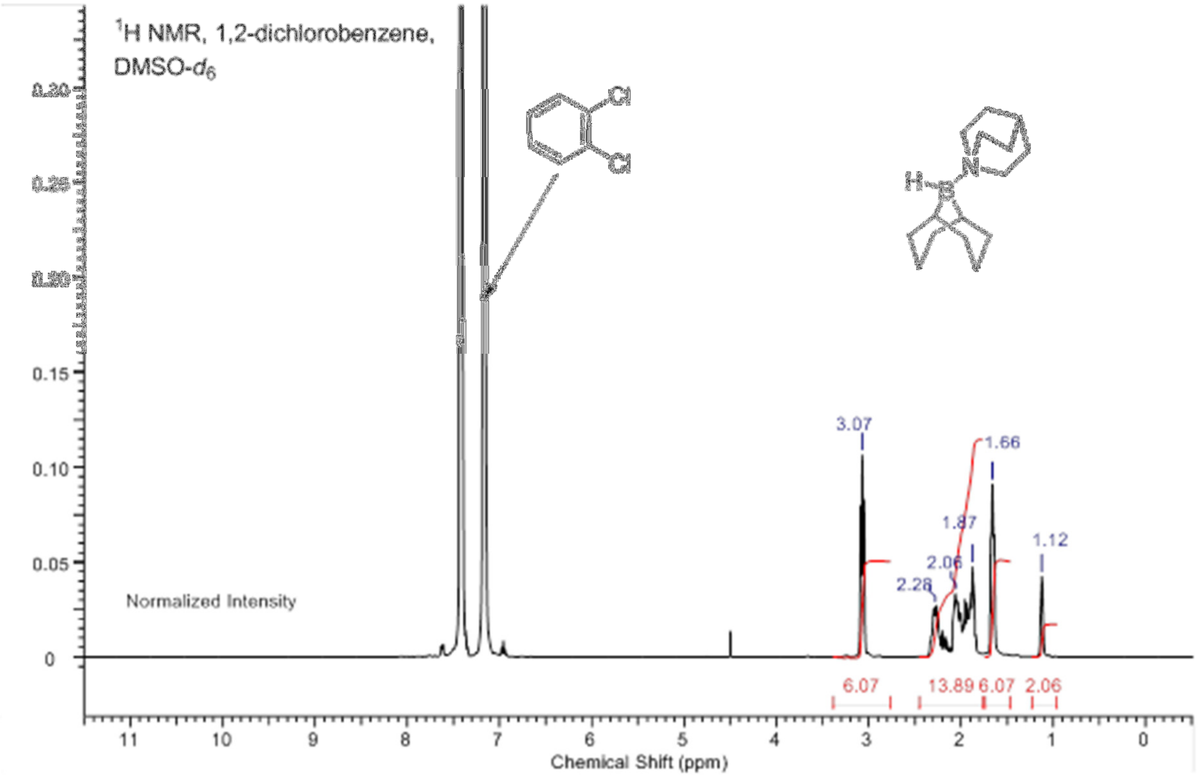

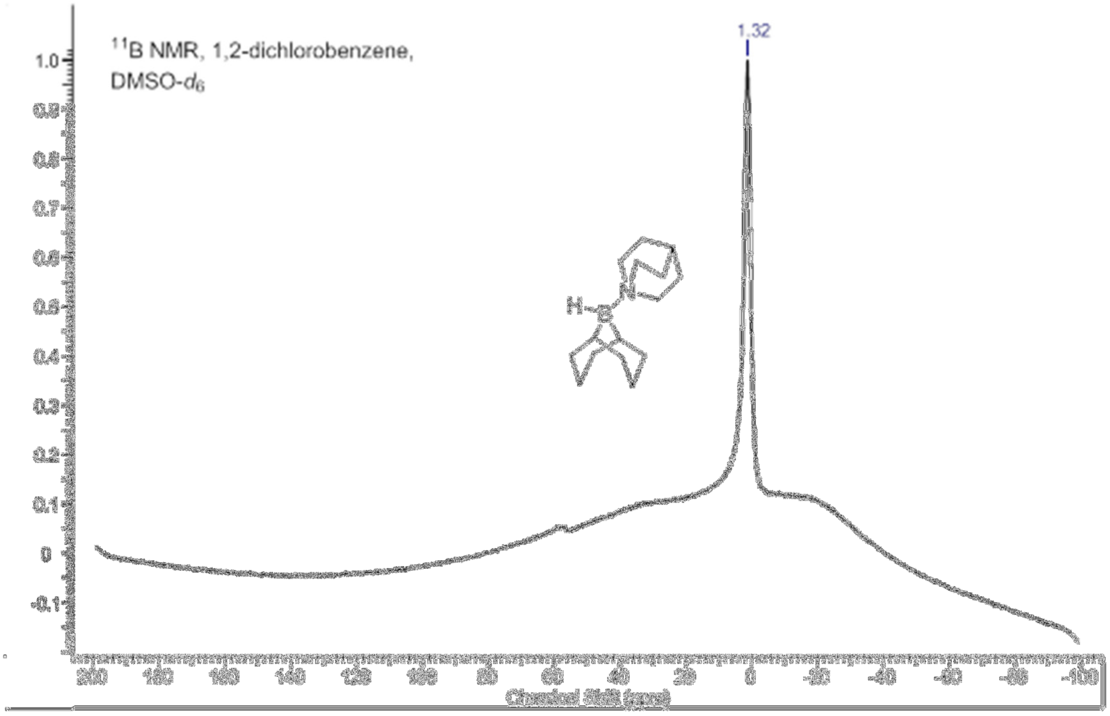

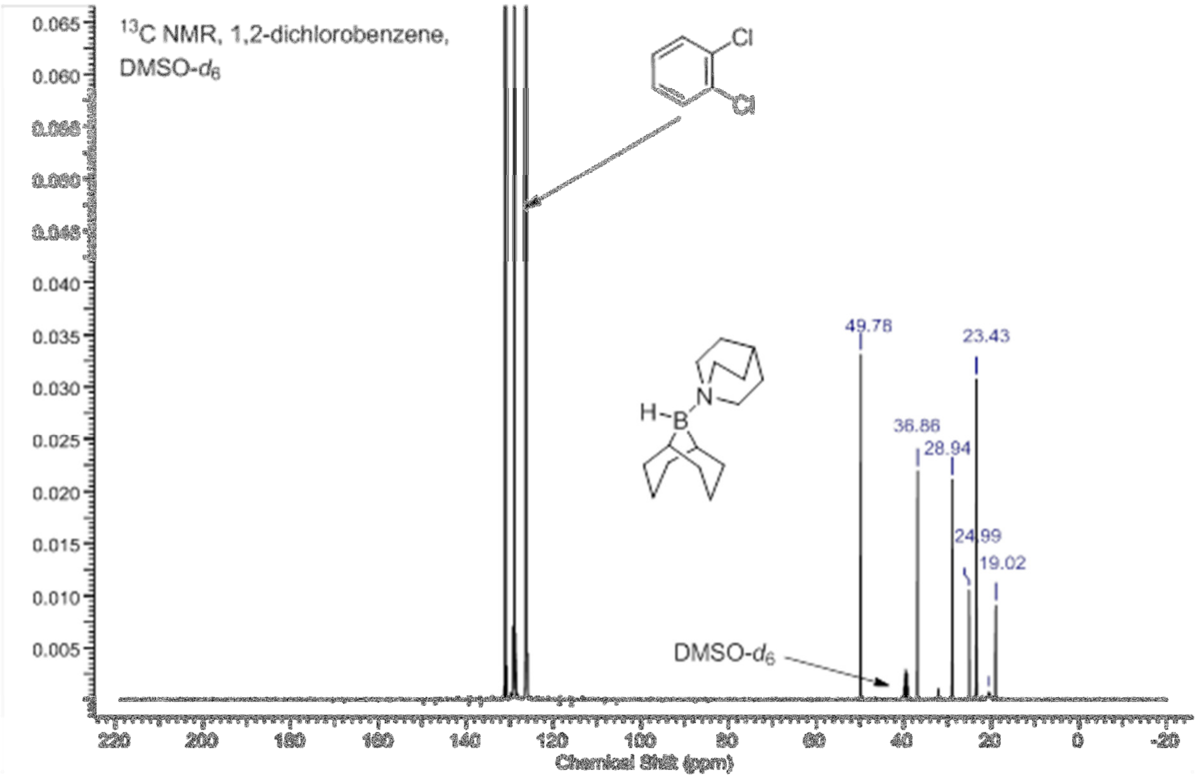

^{13}C NMR, 1,2-dichlorobenzene, DMSO-d_6
Cl
Cl
H
B
N
49.78
23.43
36.86
28.94
24.99
19.02
DMSO-d_6
0.065
0.060
0.055
0.050
0.045
0.040
0.035
0.030
0.025
0.020
0.015
0.010
0.005
220
200
180
160
140
120
100
80
60
40
20
0
-20
Chemical Shift (ppm)

In-situ NMR spectra from the reaction of **1-H**/B(C_6F_5)$_3$ with 1-pentyne: of 9-BBN(1-pentyne)(Quinucidine) (**2**).

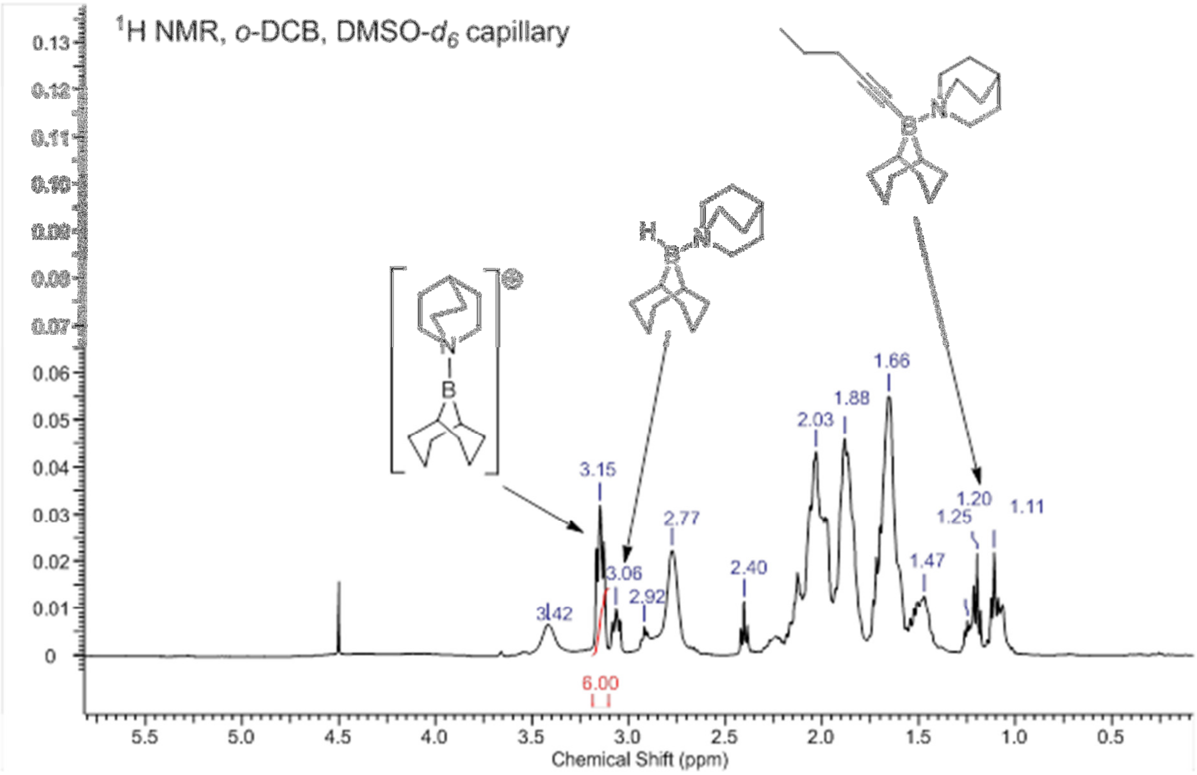

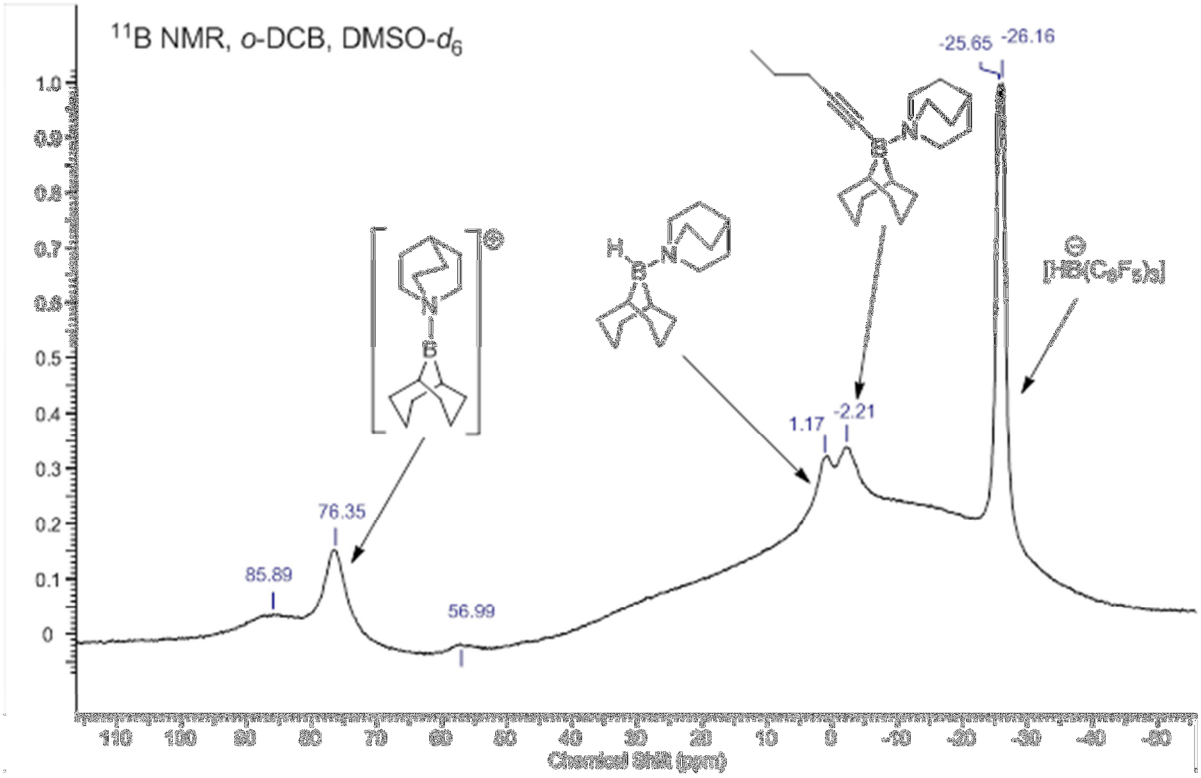

[9-BBN(Quinuclidine)][$B(C_6F_5)_4$] (**[1][$B(C_6F_5)_4$]**)

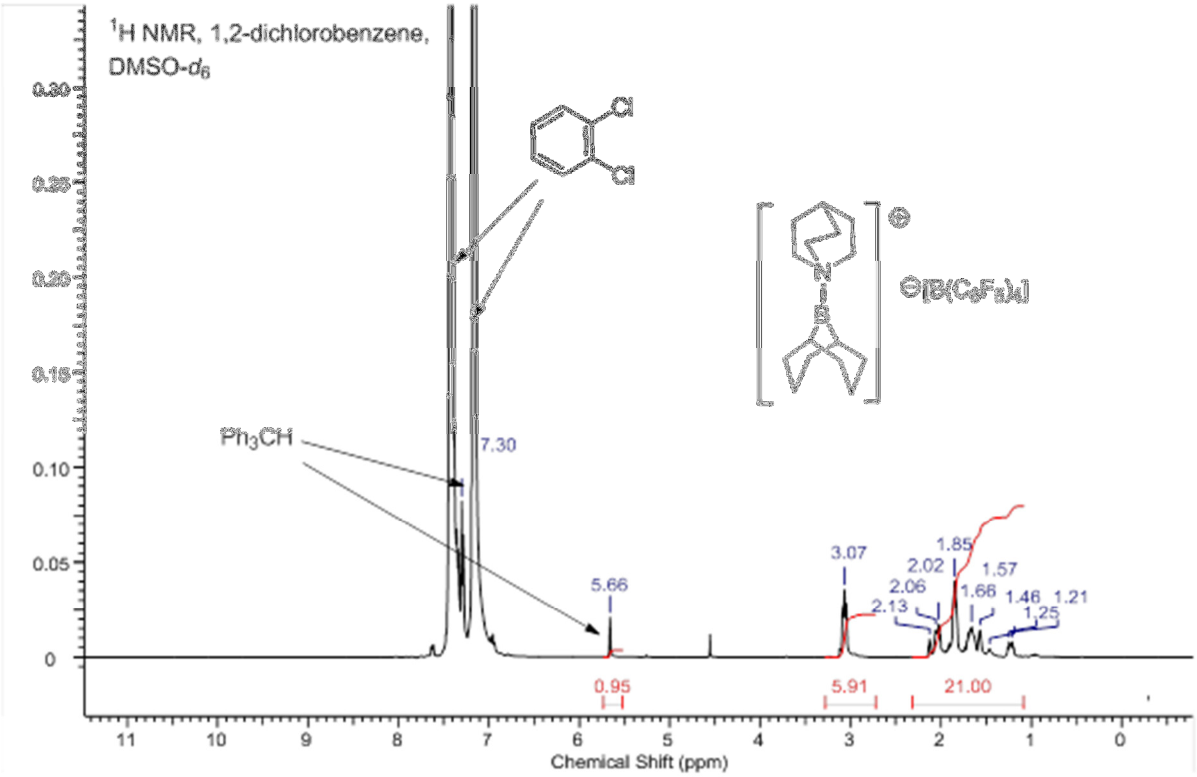

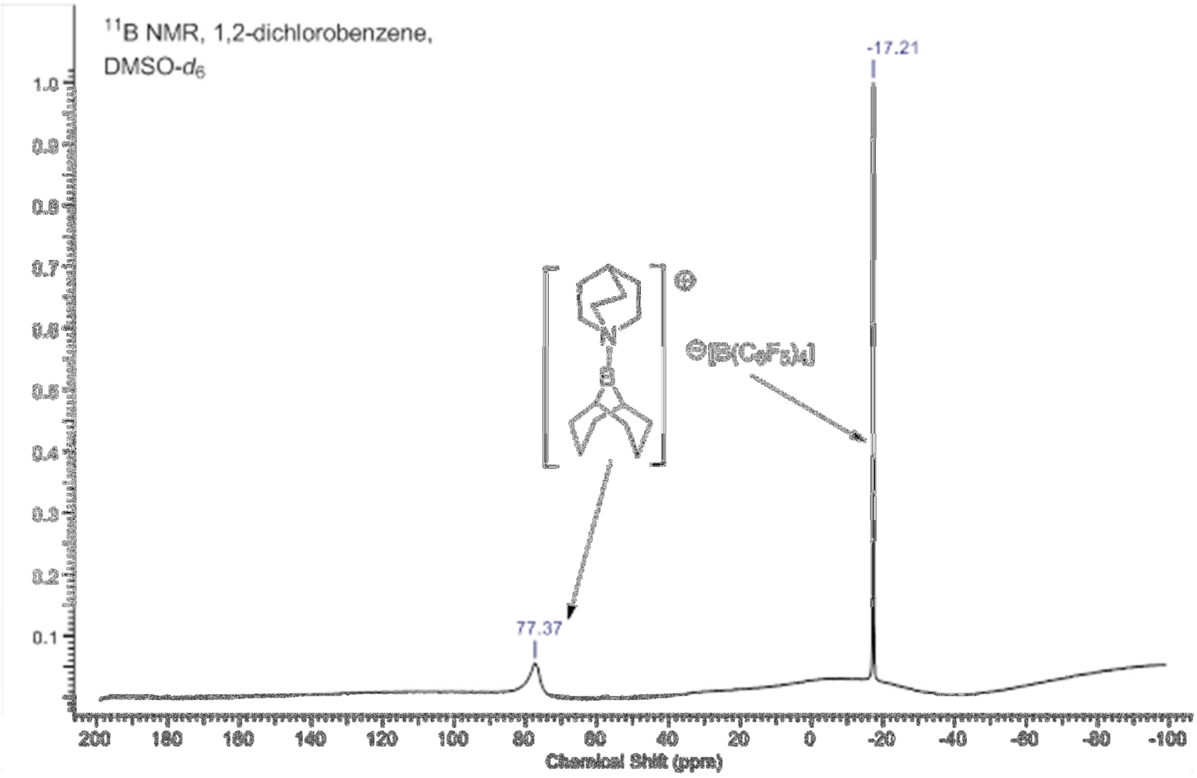

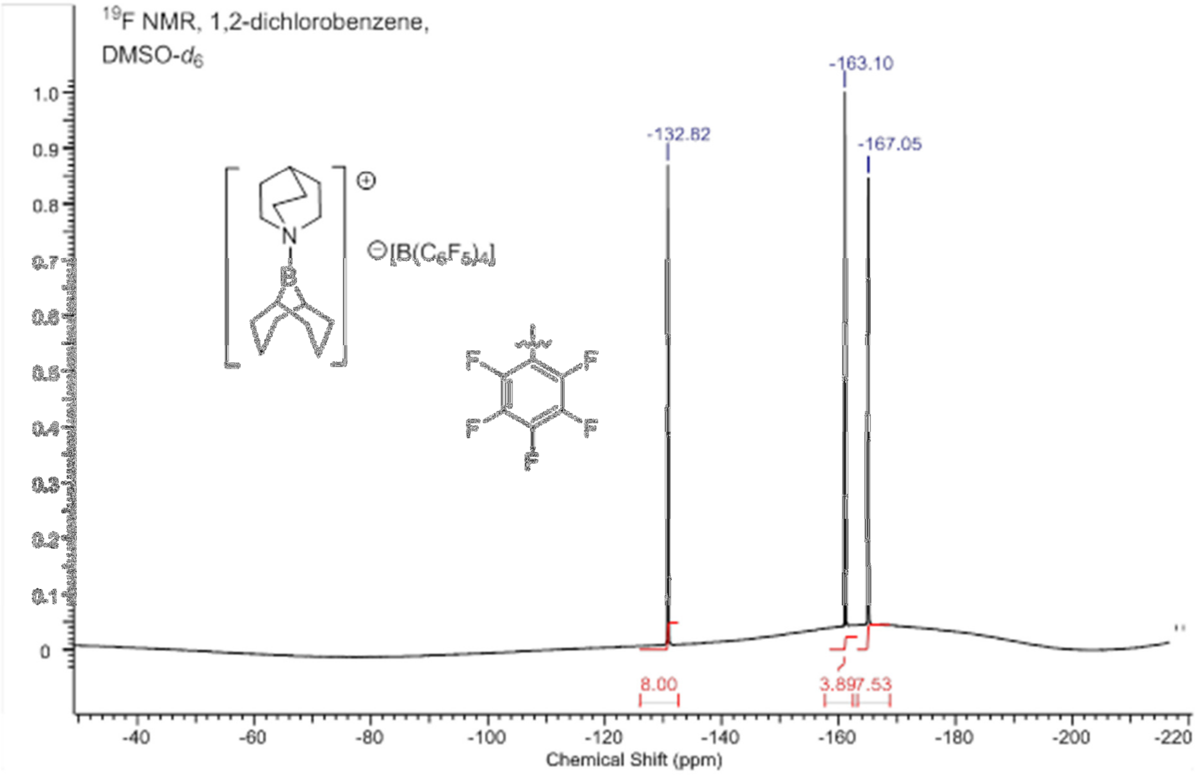

^{19}F NMR, 1,2-dichlorobenzene,
DMSO-d_6
-132.82
-163.10
-167.05
[B(C$_6$F$_5$)$_4$]
F
N
B
8.00
3.89
7.53
Chemical Shift (ppm)

Synthesis of **2** *via* an FLP approach

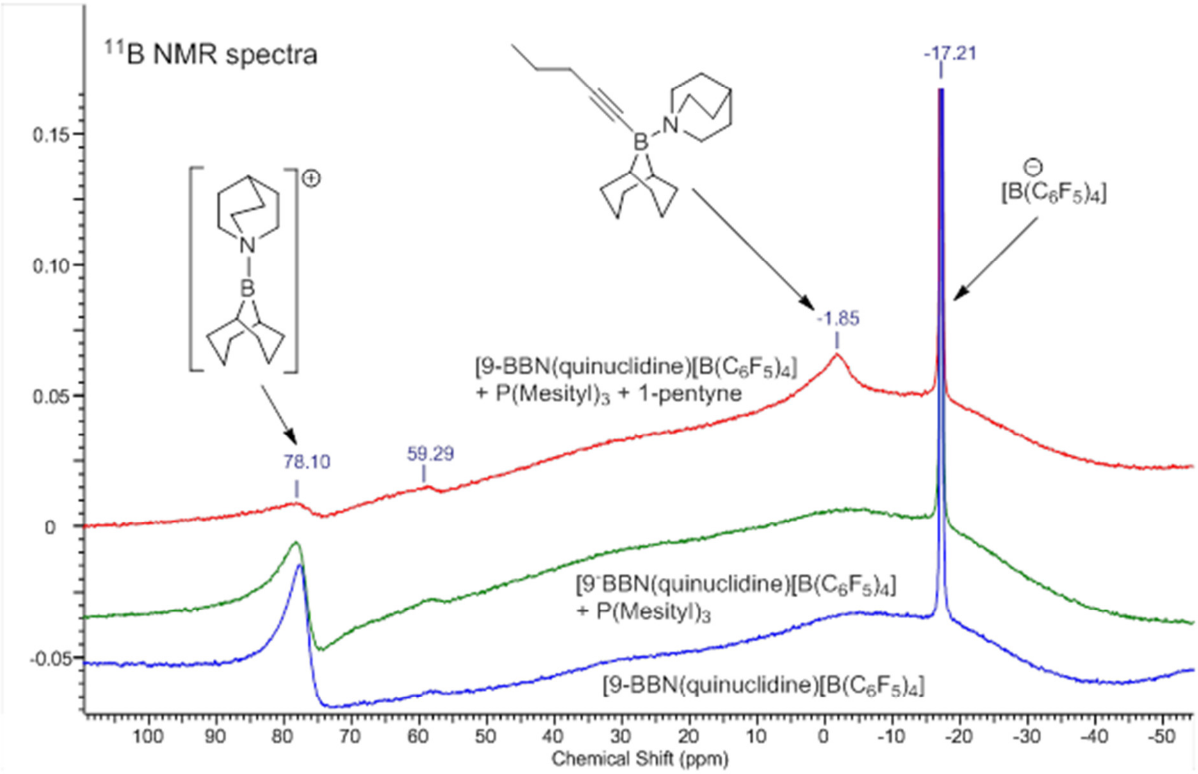

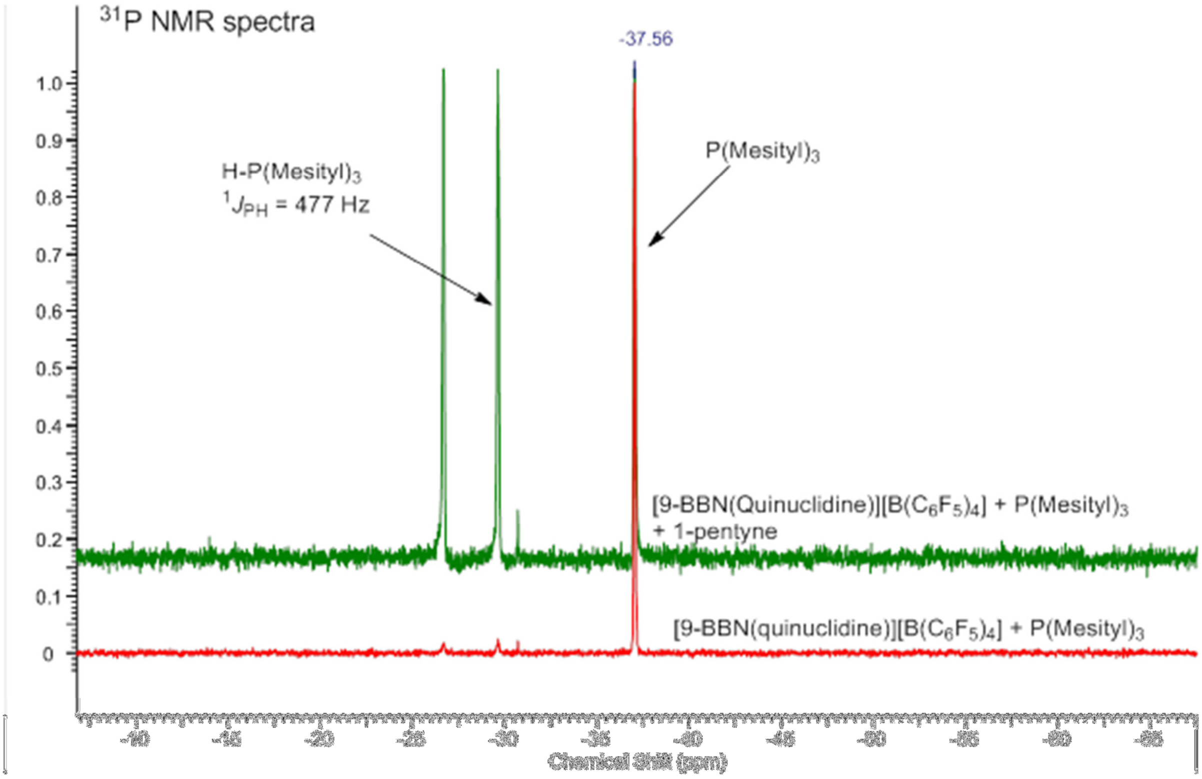

9-BBN(IMe_4)(I) (**4**)

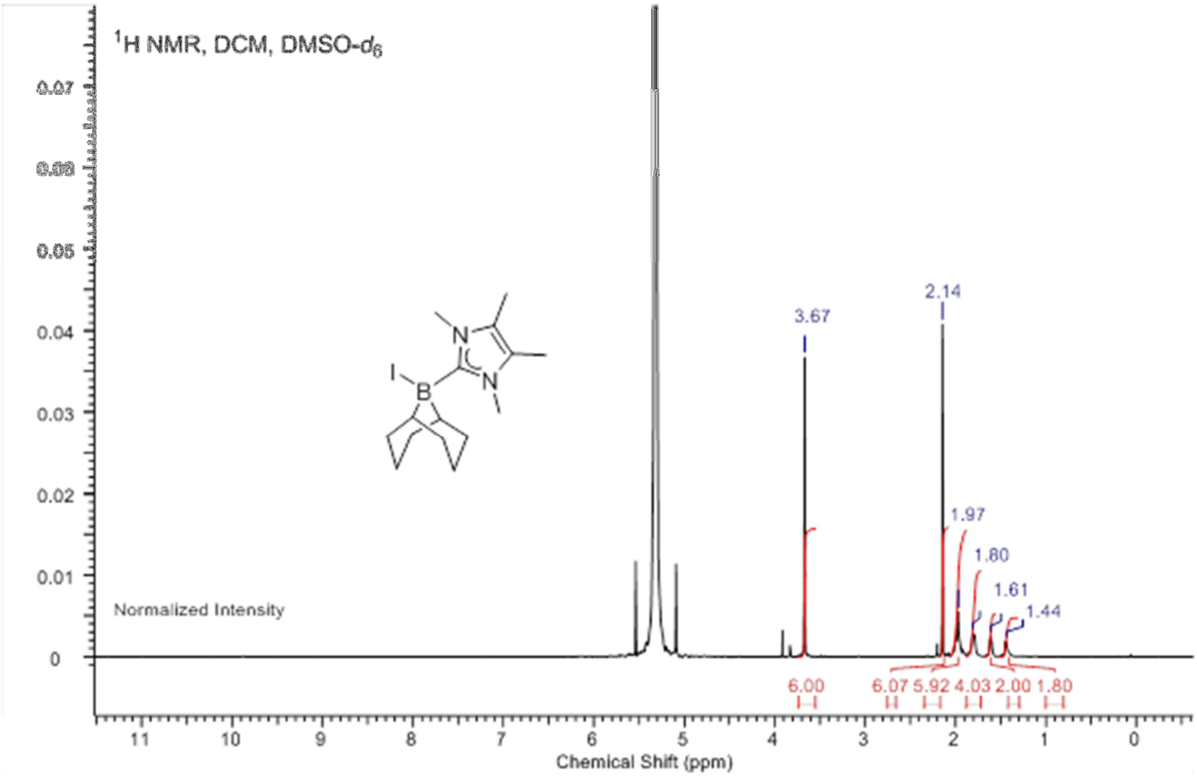

^{1}H NMR, DCM, DMSO-d6
Normalized Intensity
3.67
2.14
1.97
1.80
1.61
1.44
6.00
6.07 5.92 4.03 2.00 1.80
Chemical Shift (ppm)

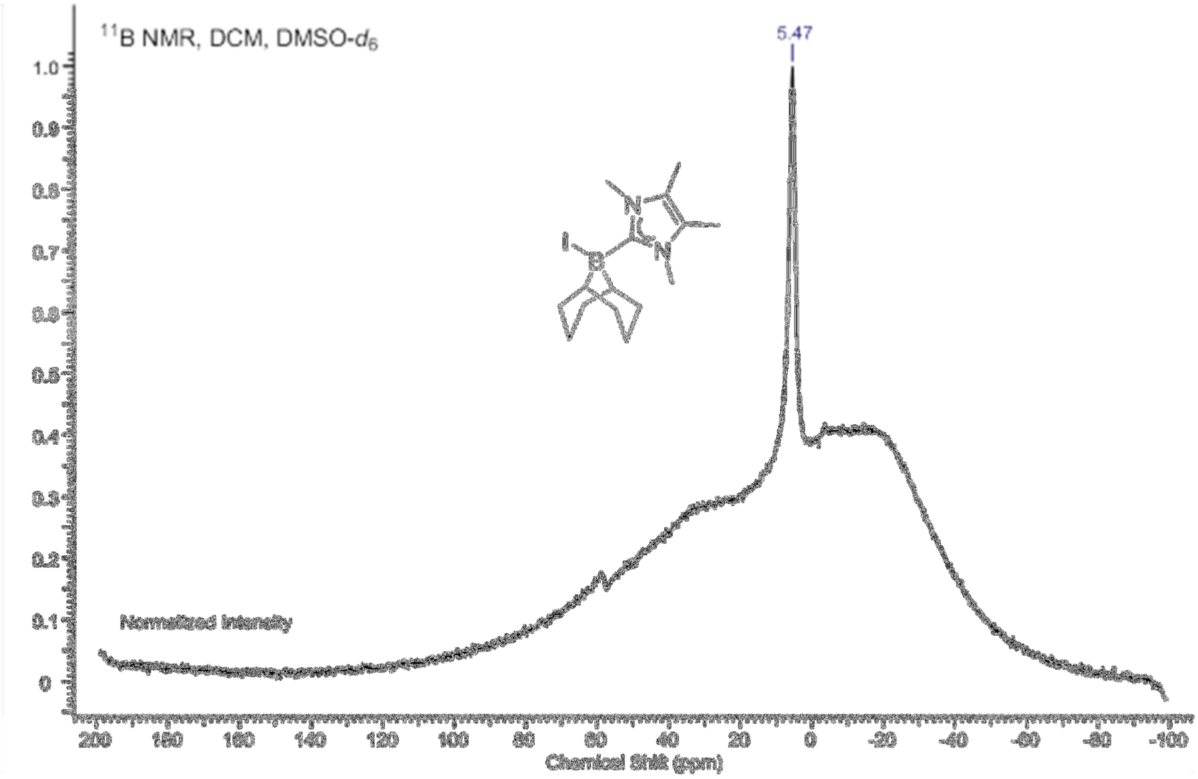

^{11}B NMR, DCM, DMSO-d6
5.47
Normalized Intensity
Chemical Shift (ppm)

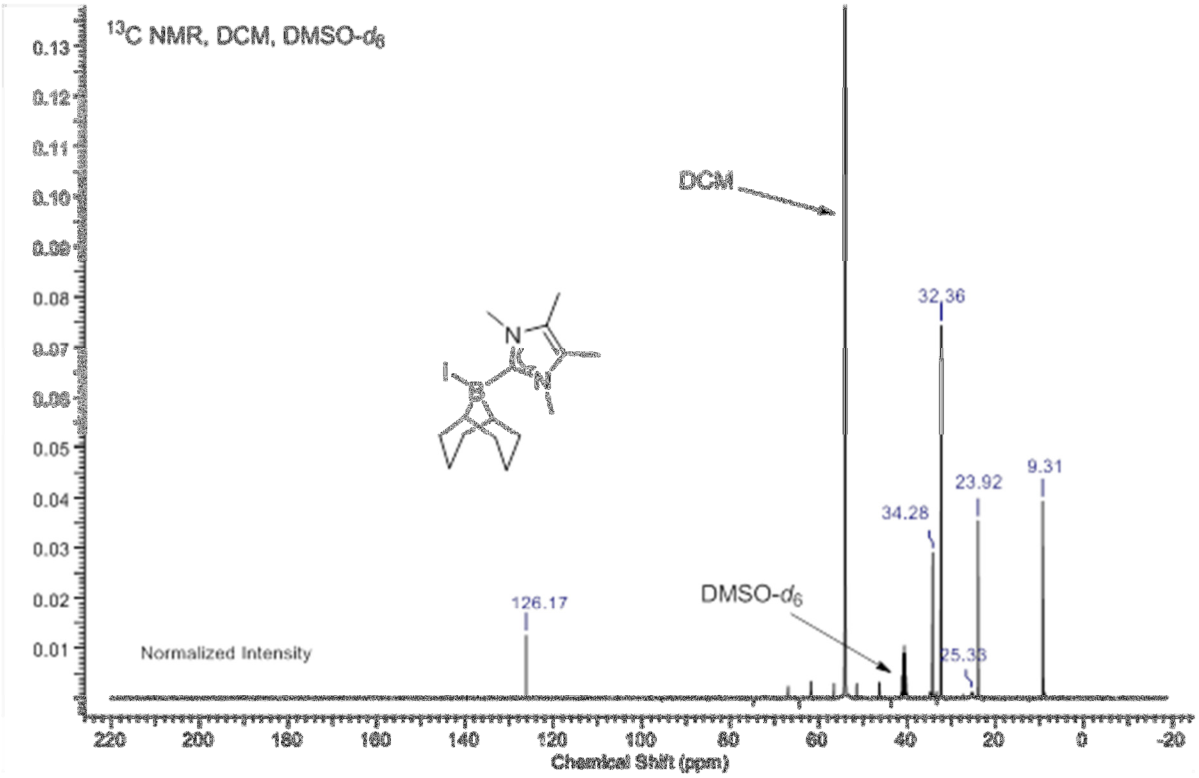

^{13}C NMR, DCM, DMSO-d_6
DCM
DMSO-d_6
126.17
34.28
32.36
25.33
23.92
9.31
Normalized Intensity
Chemical Shift (ppm)

9-BBN(1-pentyne)(IMe$_4$) (**5**) / (**4**) mixture

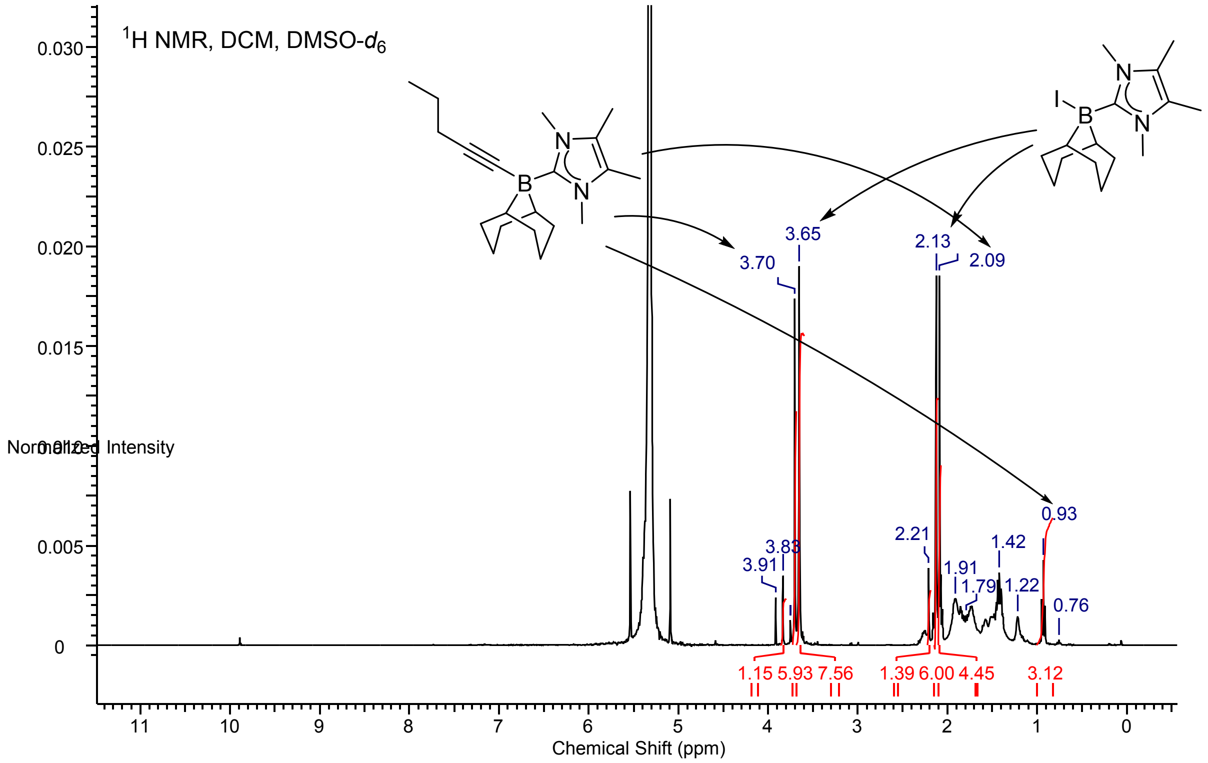

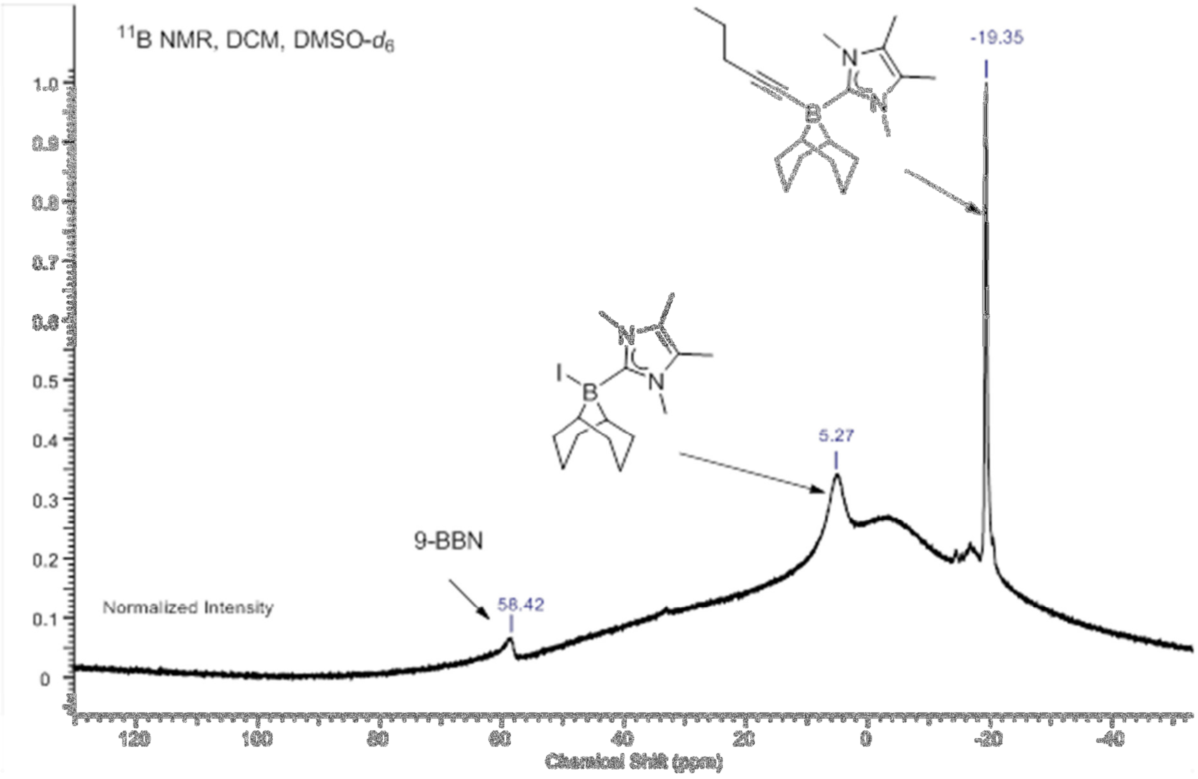

Turnover of **4** after the addition of **3** and 1-pentyne

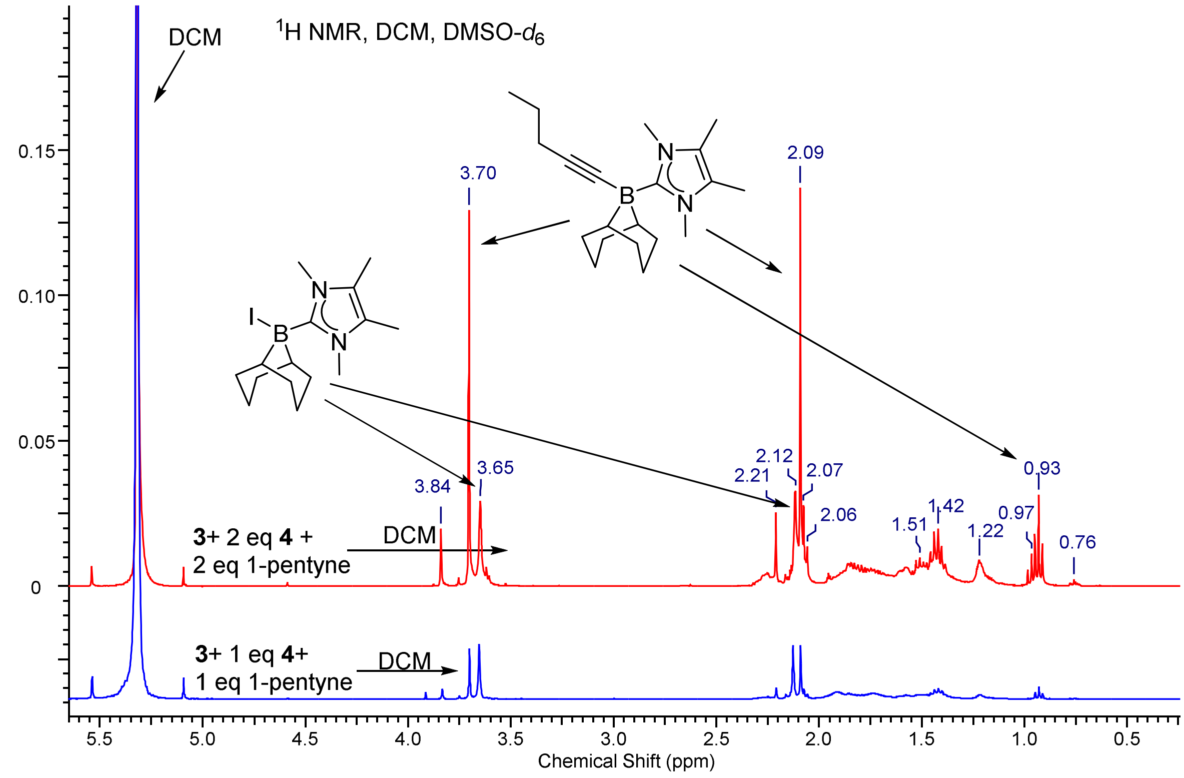

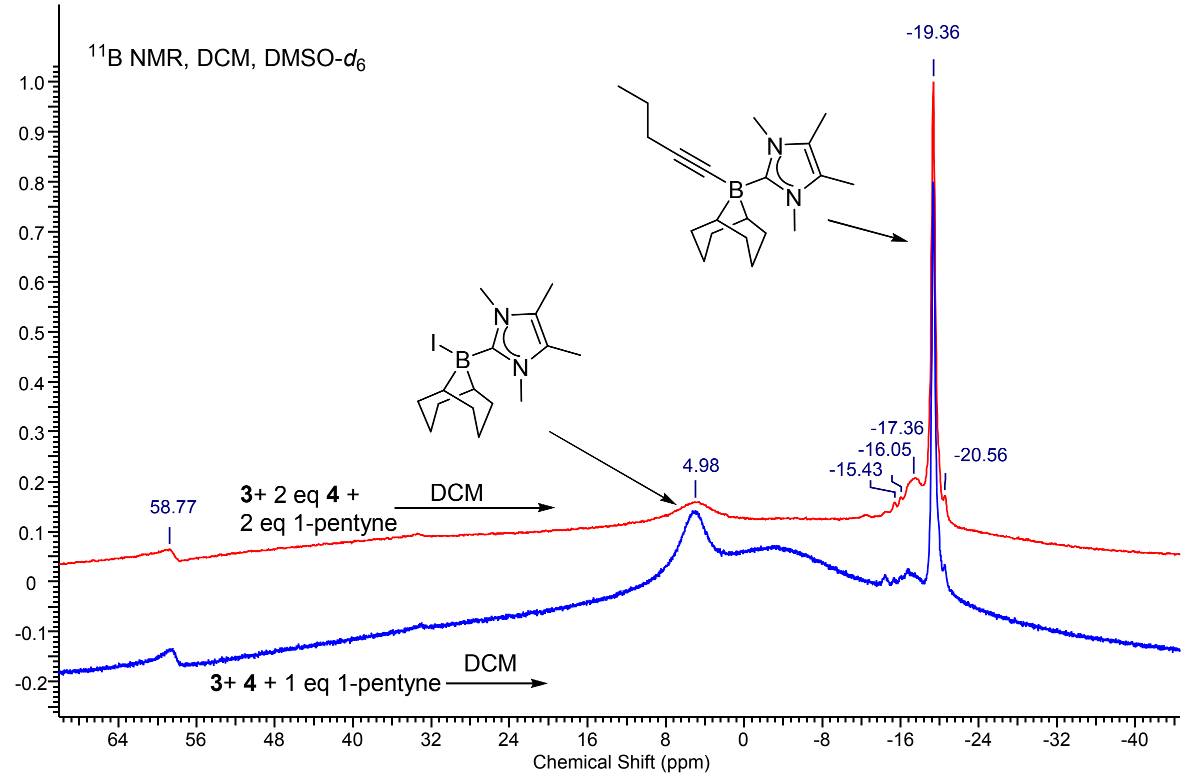

[9-BBN(IMe$_4$)][HB(C$_6$F$_5$)$_3$] (**6**)

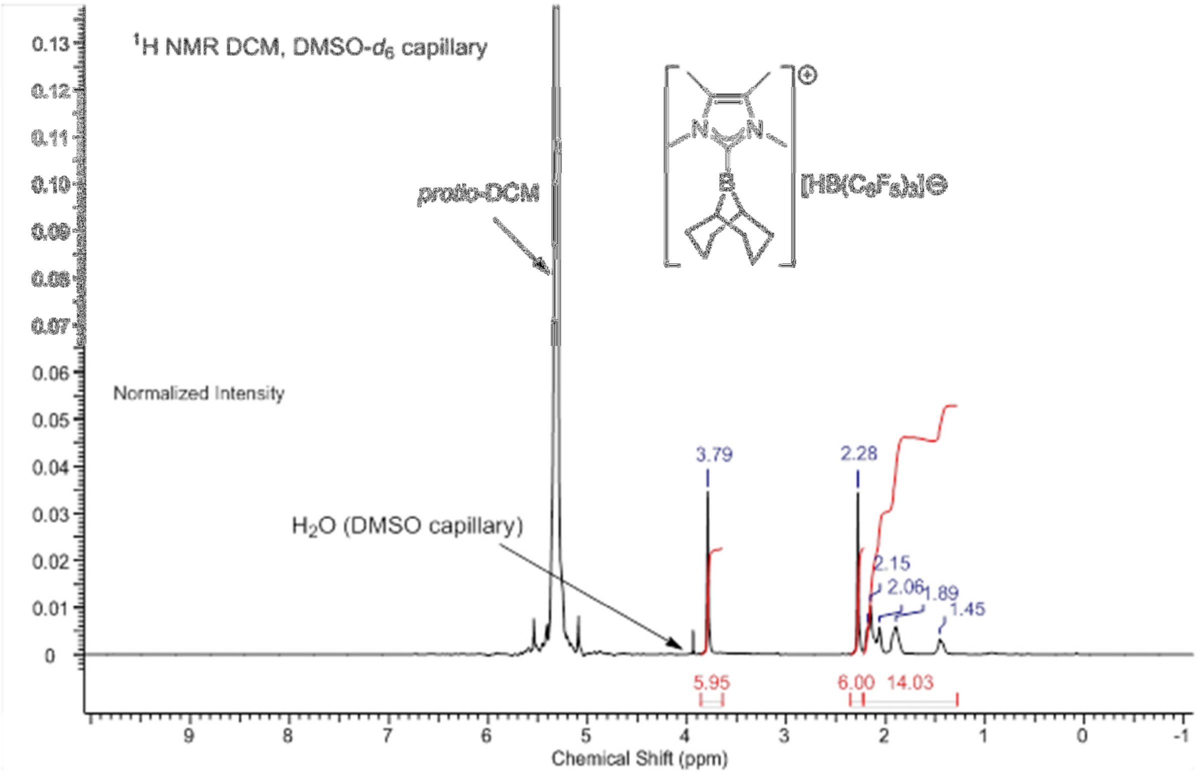

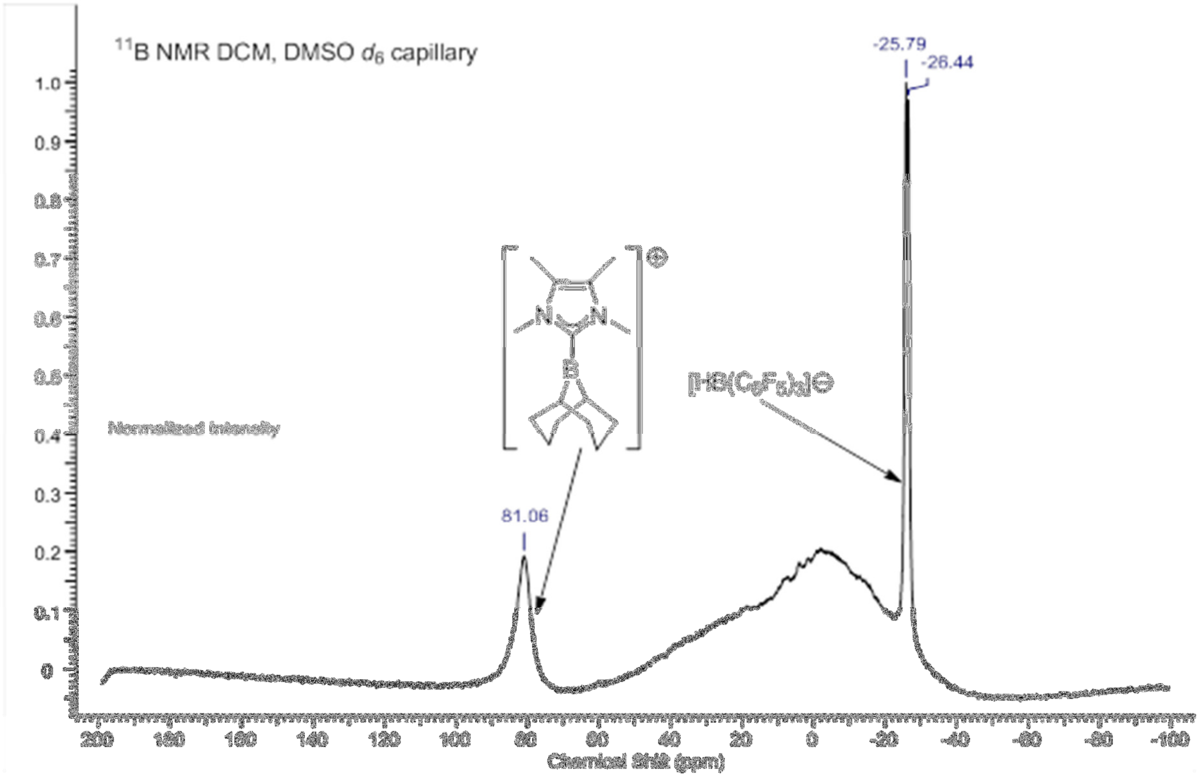

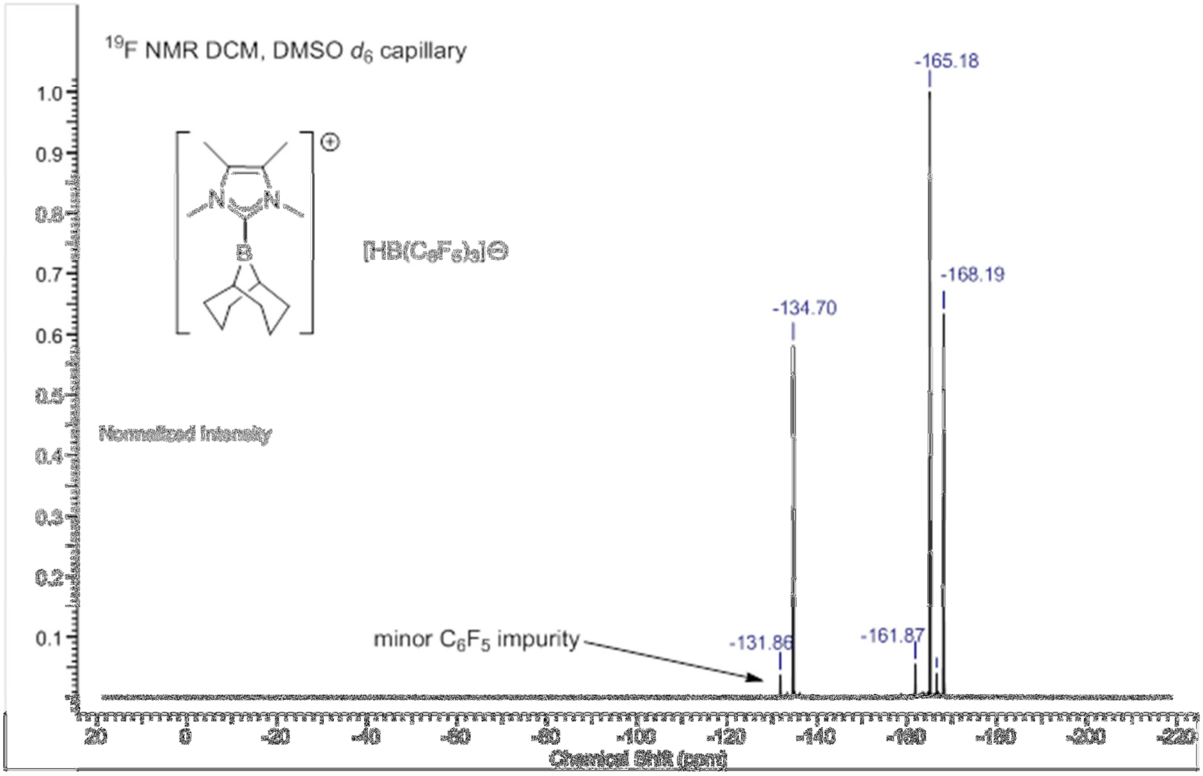
19F NMR DCM, DMSO d_6 capillary
[HB(C6F5)3]⊖
-165.18
-168.19
-134.70
Normalized Intensity
minor C6F5 impurity
-131.86
-161.87
Chemical Shift (ppm)

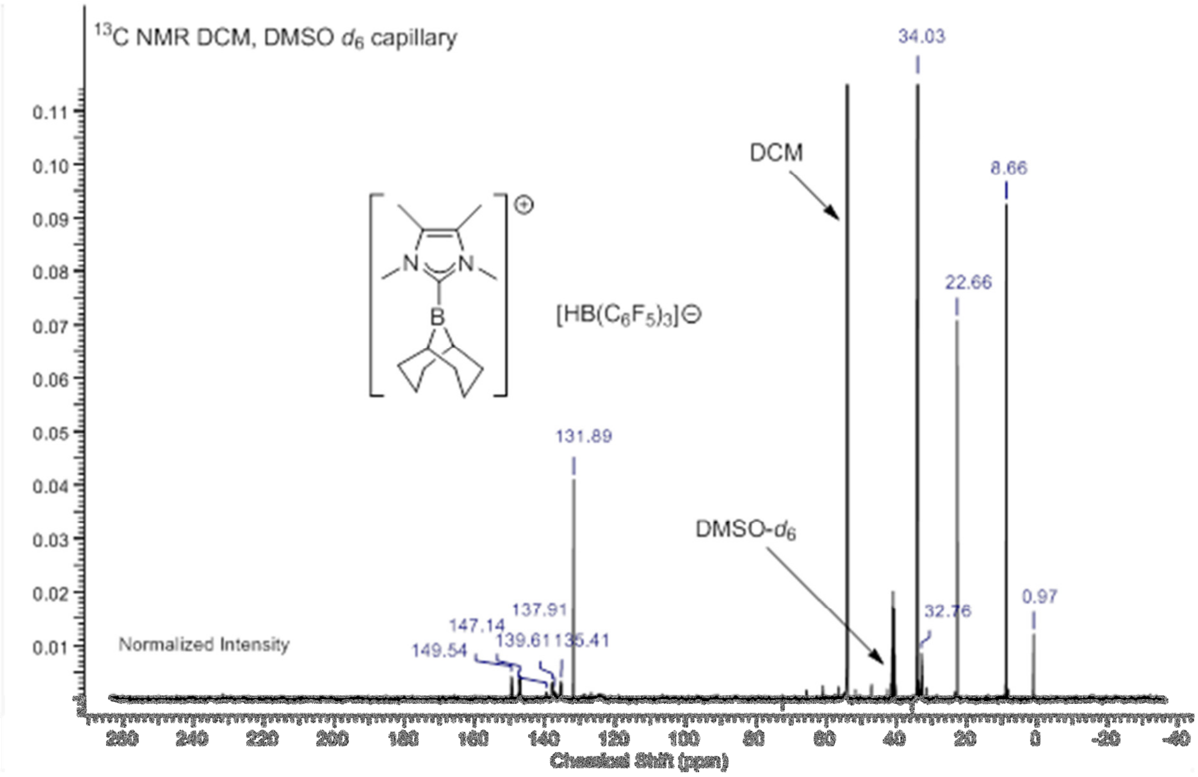
13C NMR DCM, DMSO d_6 capillary
34.03
DCM
8.66
22.66
[HB(C6F5)3]⊖
131.89
DMSO-d_6
137.91
147.14
139.61
135.41
149.54
32.76
0.97
Normalized Intensity
Chemical Shift (ppm)

NMR spectra of **3** and **6**.

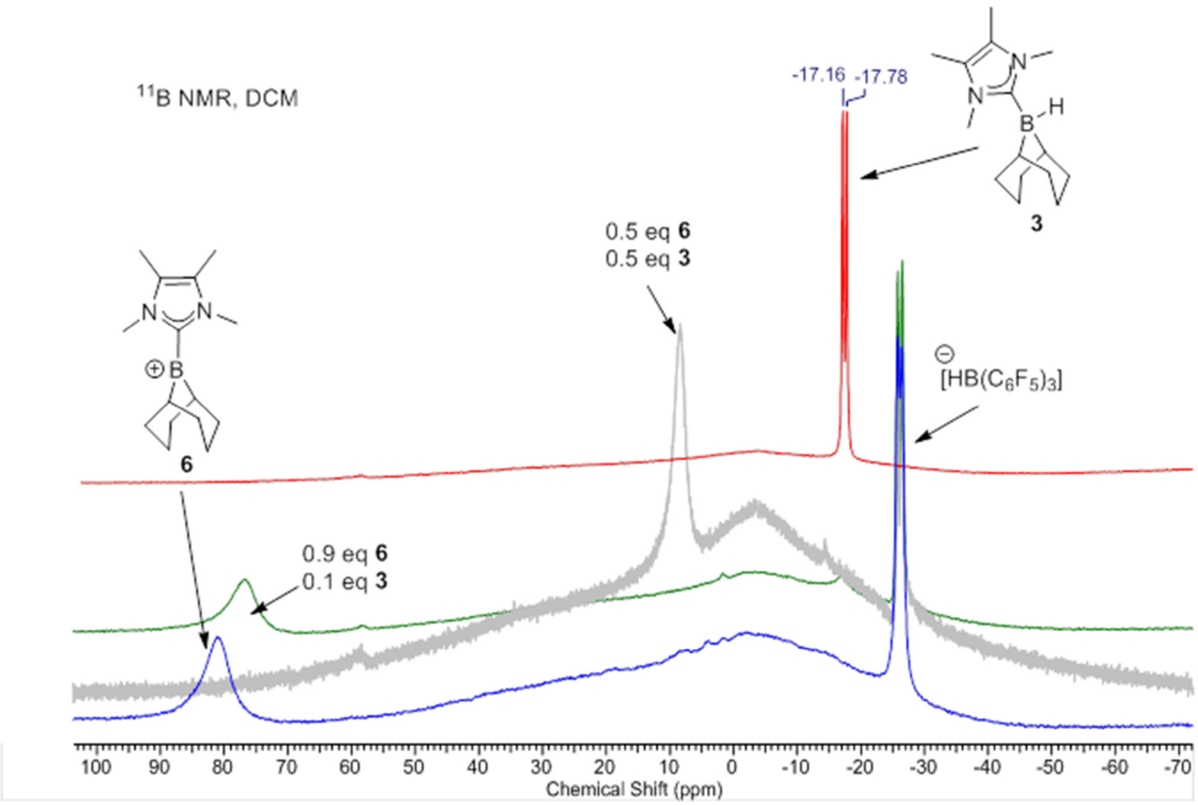

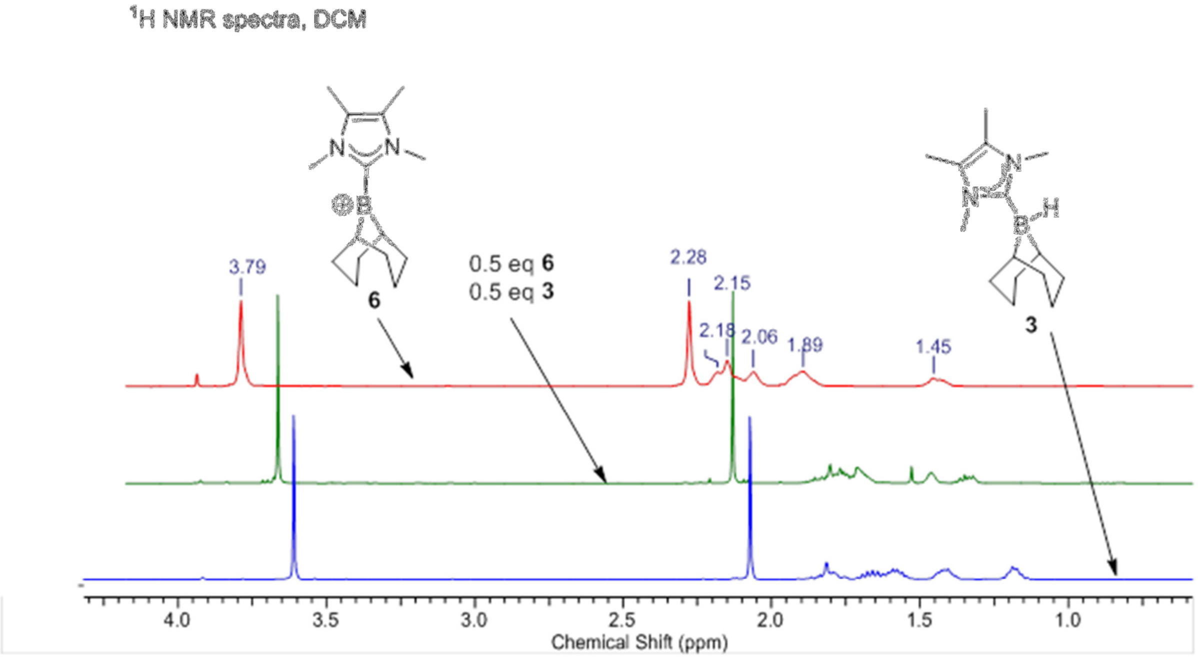

NMR spectra of **8** and **9**.

In this stack plot, the NMR of [9-BBN(I-DCDM)][$B(C_6F_5)_4$] is used as the reference for the **9**, to increase the clarity of the stack plot. The cationic components of **9** and [9-BBN(I-DCDM)][$B(C_6F_5)_4$] give almost identical resonances in the ^{11}B NMR spectrum.

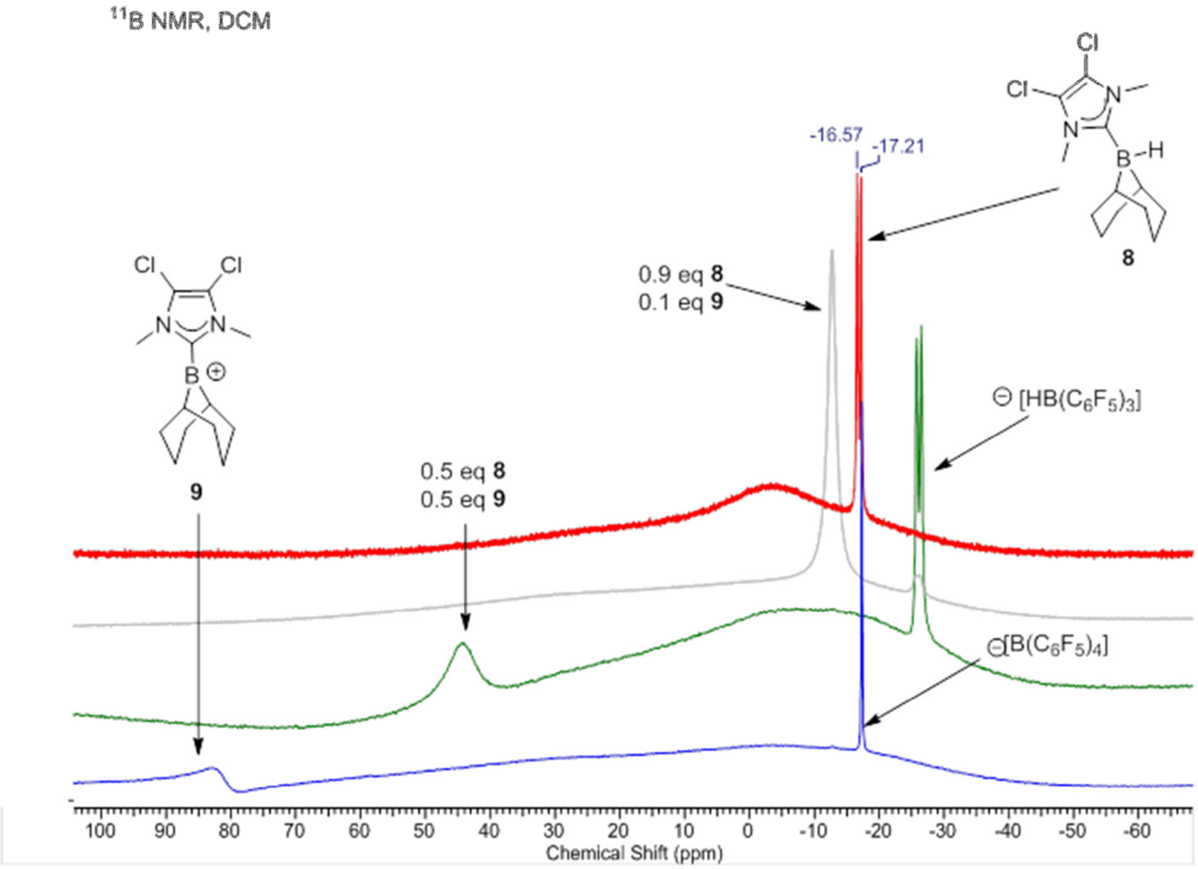

NMR spectra of 9-BBN(H)(IMes) and [9-BBN(IMes)][$HB(C_6F_5)_3$].

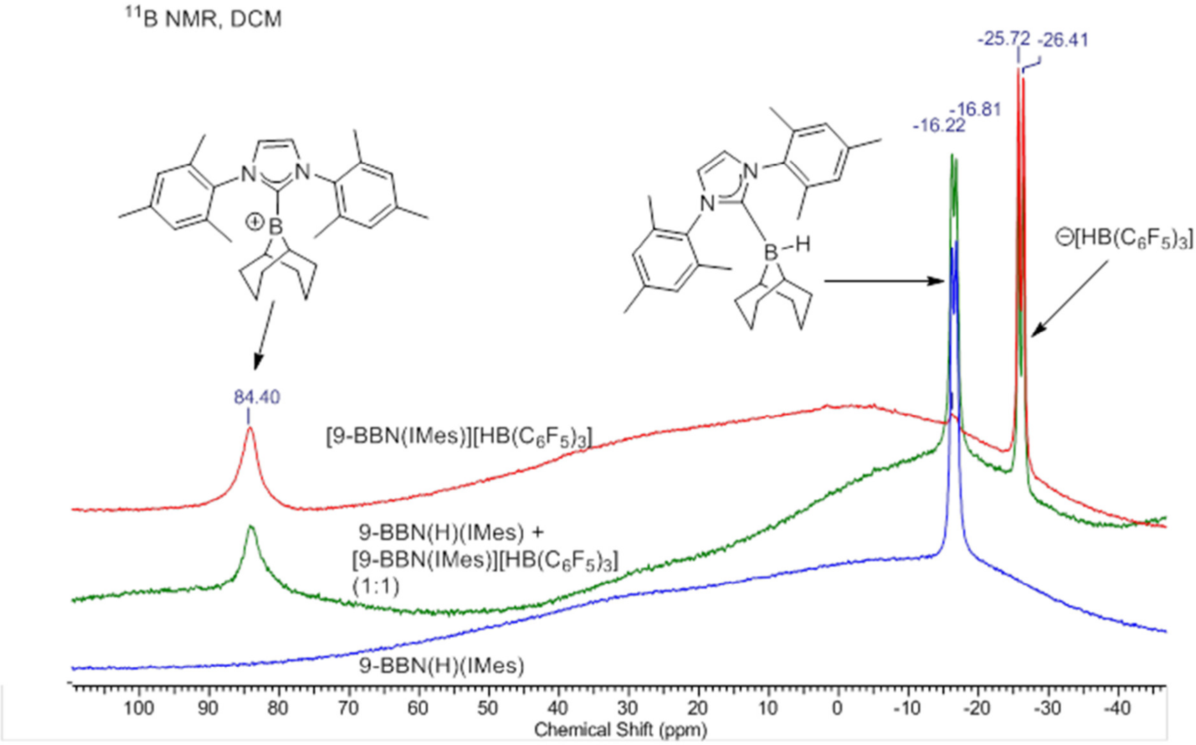

9-BBN(IMe$_4$)(Z-pent-1-ene) **7a**

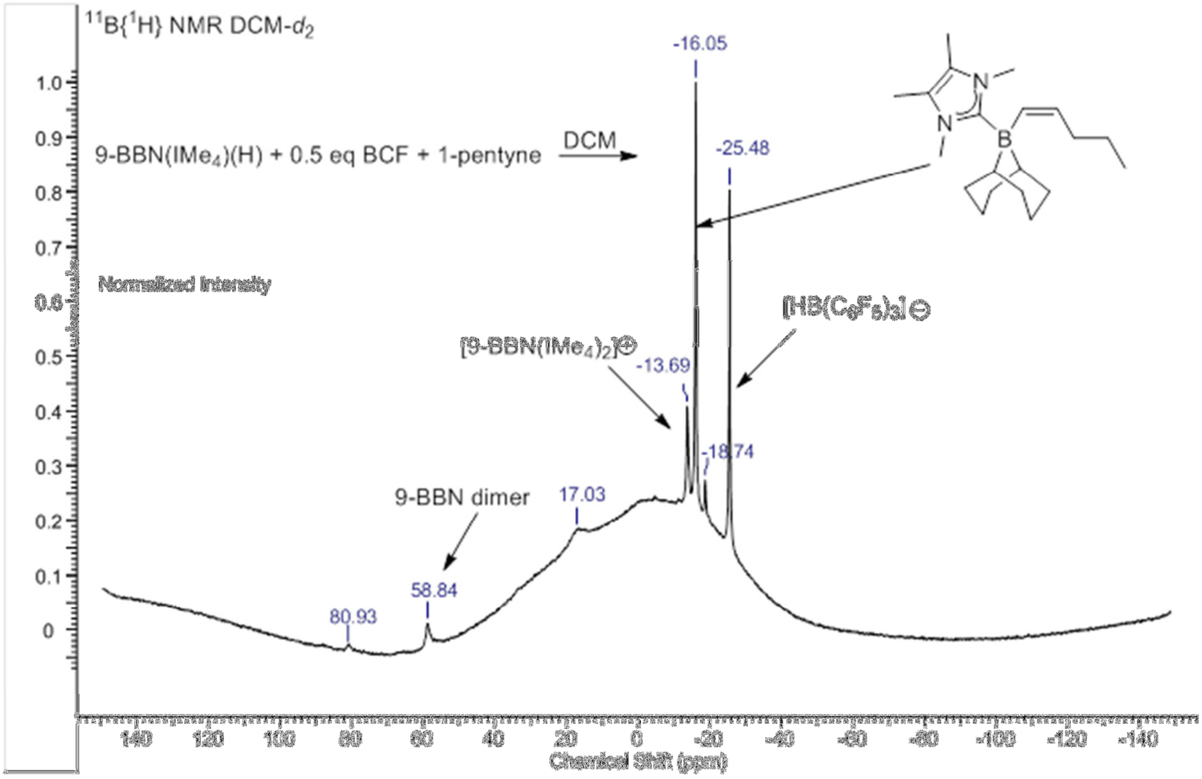

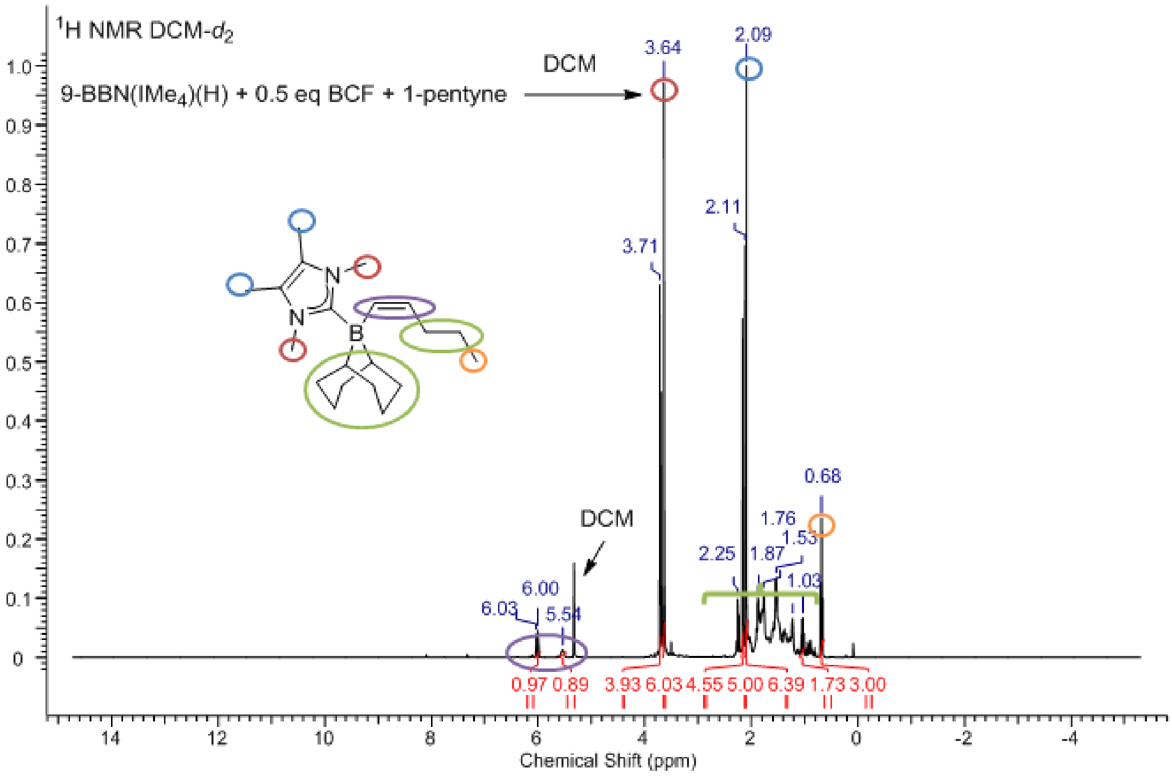

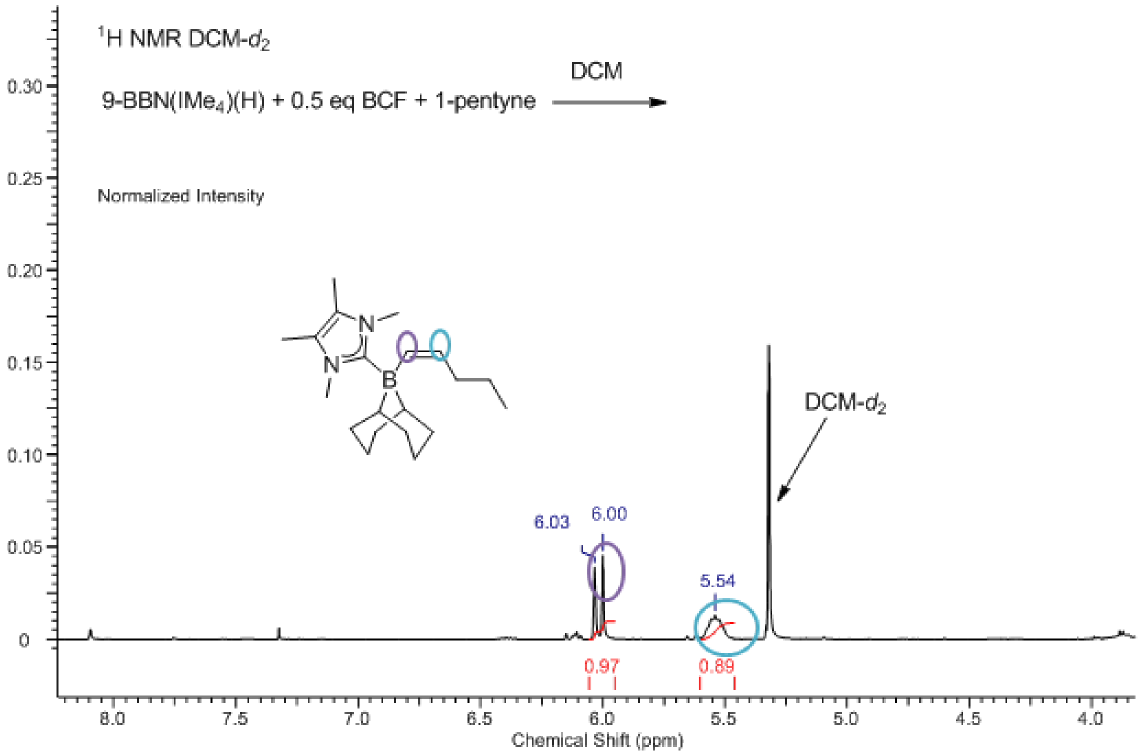

^{1}H NMR DCM-d_2
9-BBN(IMe$_4$)(H) + 0.5 eq BCF + 1-pentyne
DCM
Normalized Intensity
DCM-d_2
6.03
6.00
5.54
0.97
0.89
Chemical Shift (ppm)

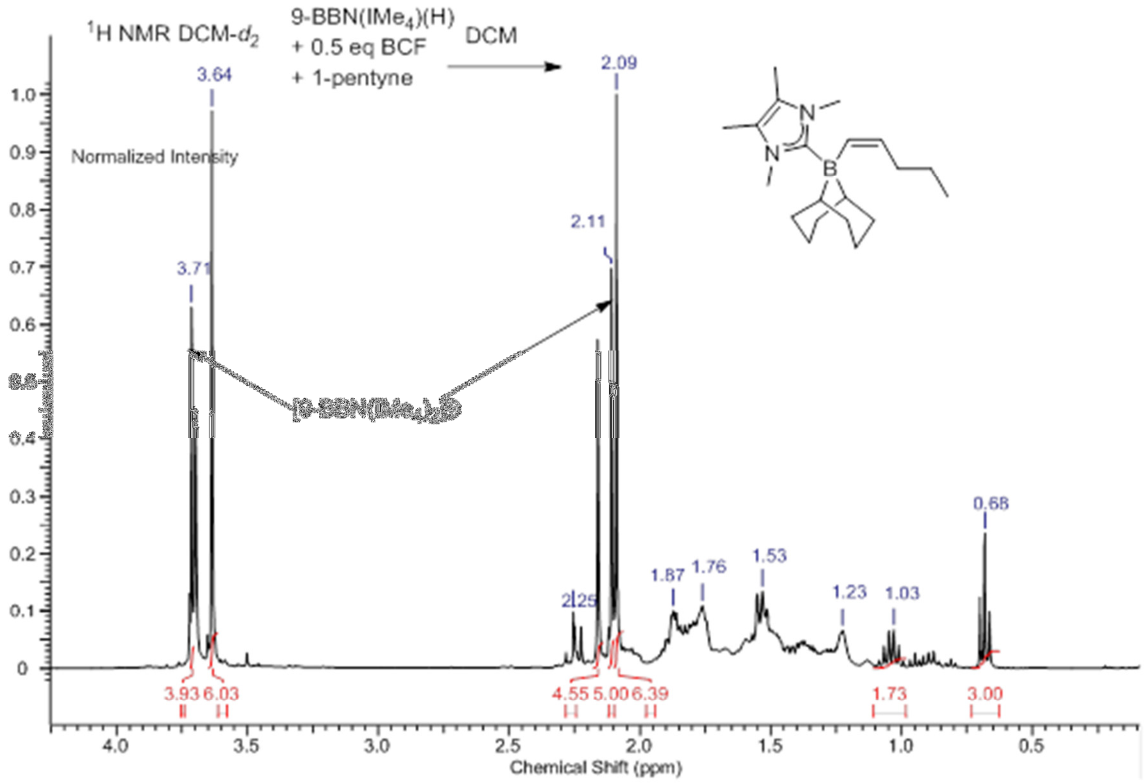

^{1}H NMR DCM-d_2
9-BBN(IMe$_4$)(H)
+ 0.5 eq BCF
+ 1-pentyne
DCM
Normalized Intensity
3.64
3.71
2.09
2.11
2.25
1.87
1.76
1.53
1.23
1.03
0.68
3.93
6.03
4.55
5.00
6.39
1.73
3.00
Chemical Shift (ppm)

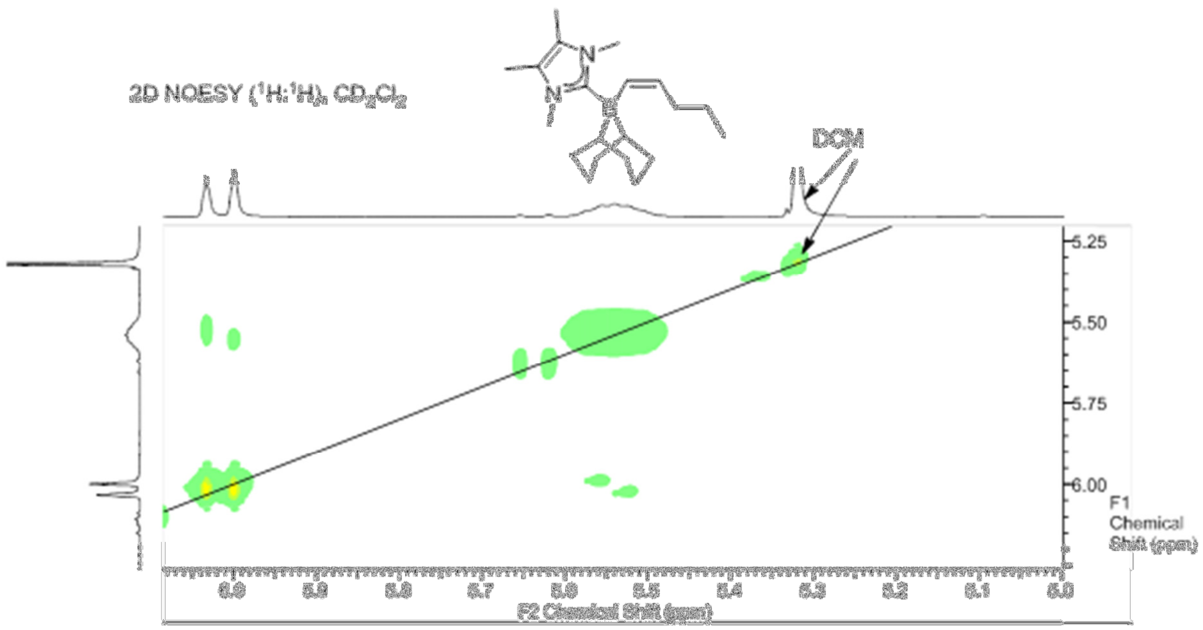

2D NOESY (1H:1H), CD2Cl2
DCM
5.25
5.50
5.75
6.00
F1 Chemical Shift (ppm)
6.0
5.9
5.8
5.7
5.6
5.5
5.4
5.3
5.2
5.1
5.0
F2 Chemical Shift (ppm)

9-BBN(IMe4)(E-1-pentene) **7b**

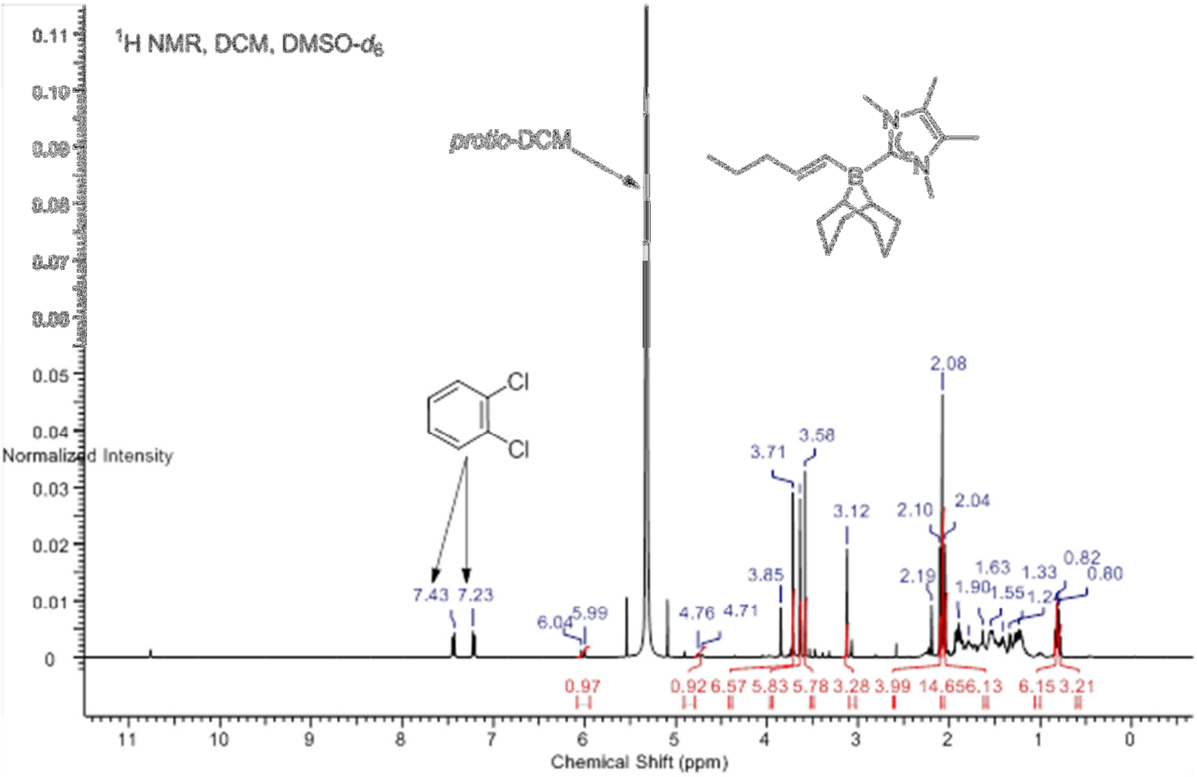

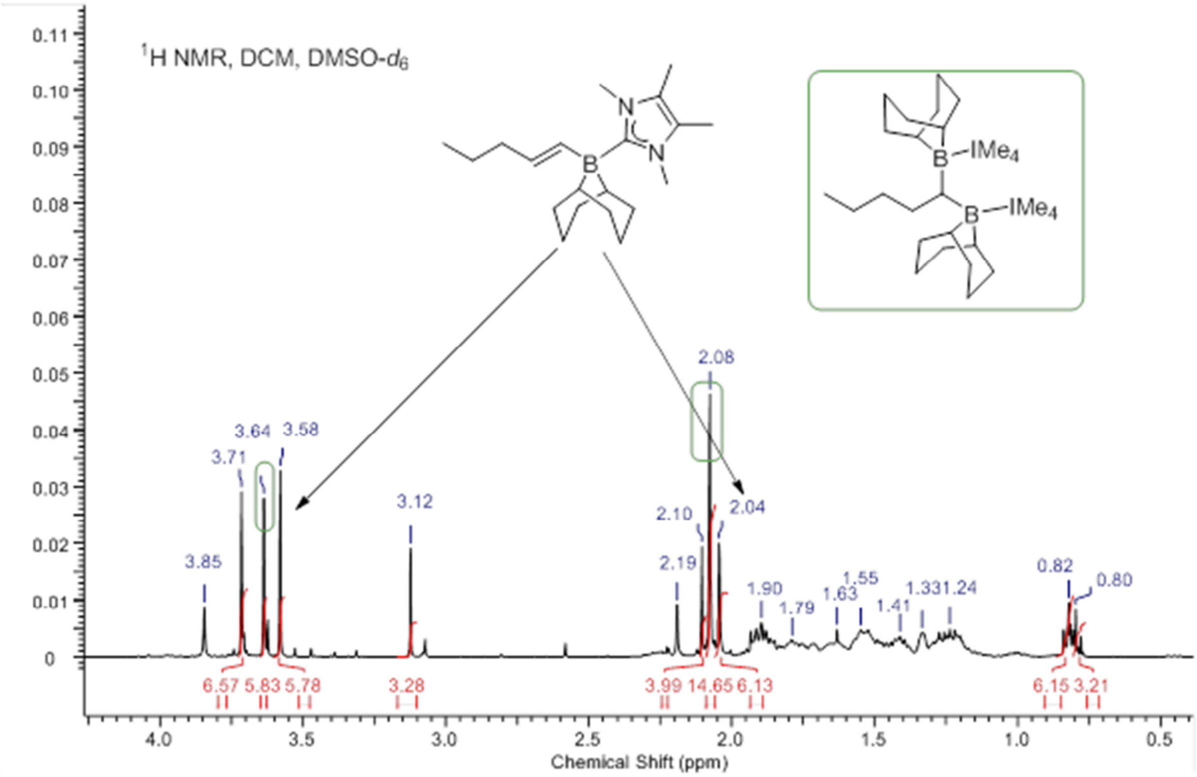

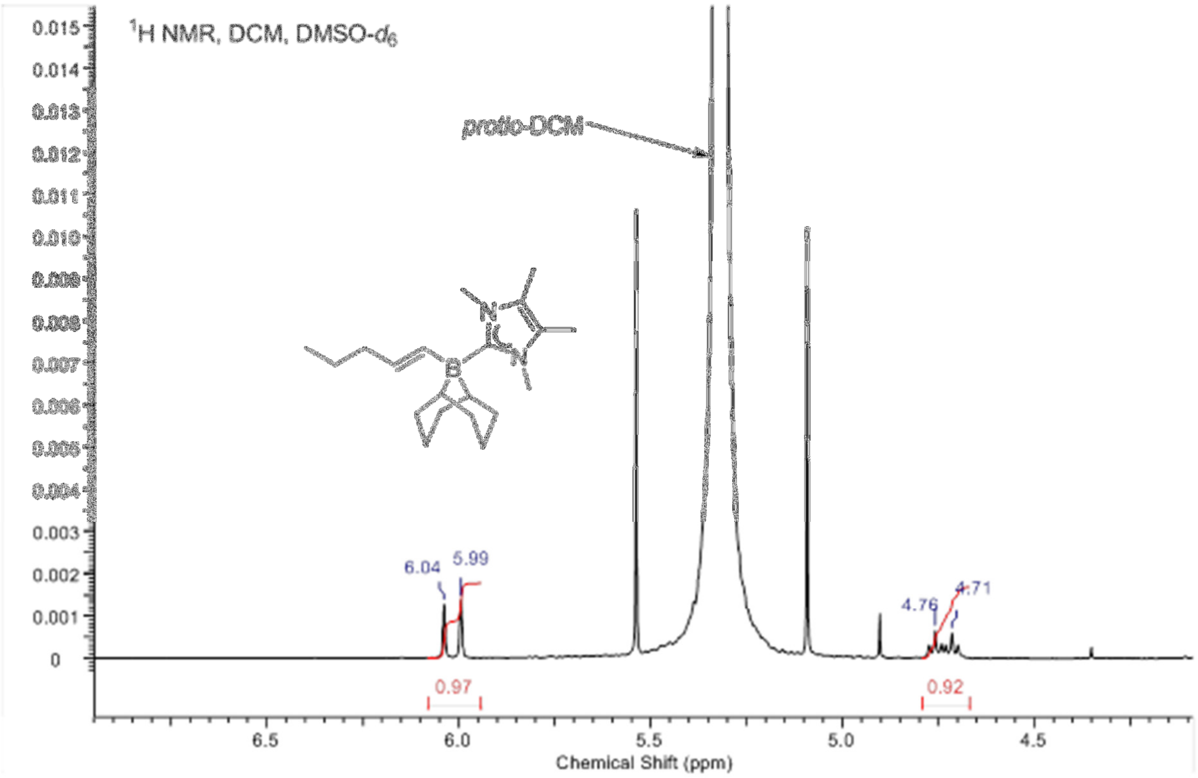

1H NMR, DCM, DMSO-d6
protio-DCM
6.04
5.99
4.76
4.71
0.97
0.92
Chemical Shift (ppm)

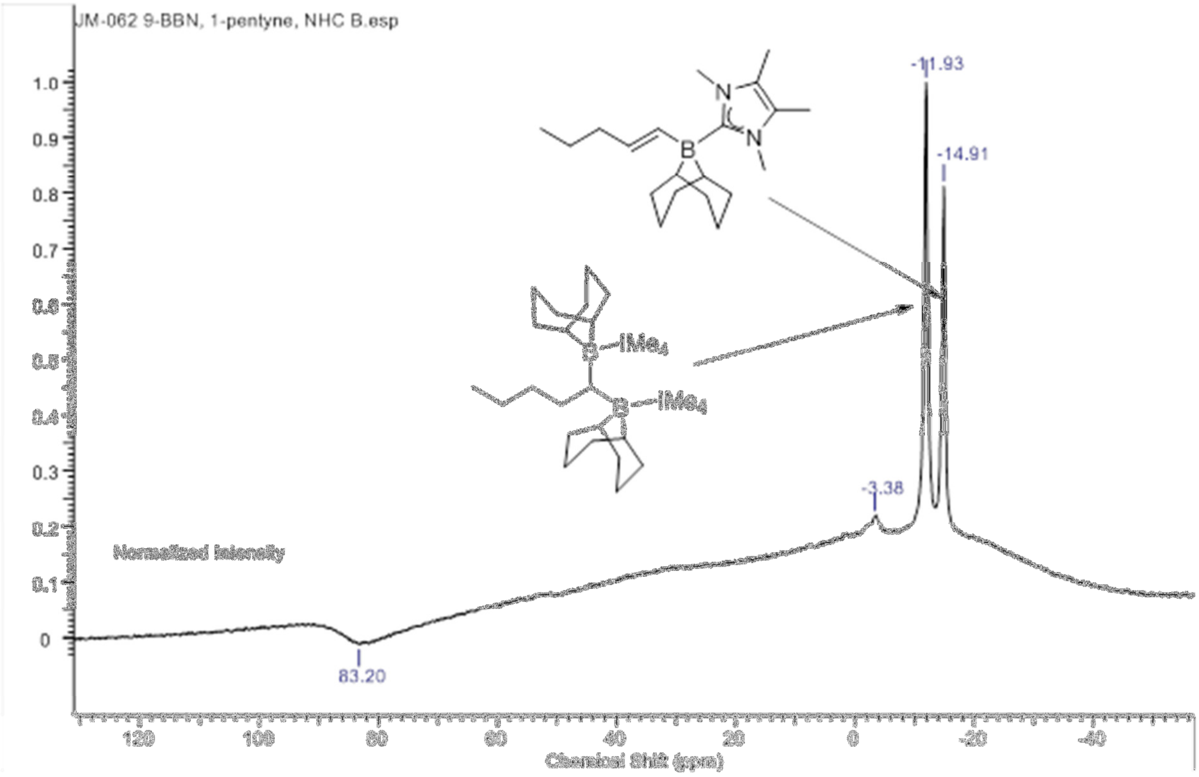

JM-062 9-BBN, 1-pentyne, NHC B.esp
-11.93
-14.91
-3.38
83.20
IMe4
IMe4
Normalized Intensity
Chemical Shift (ppm)

[9-BBN(I-DCDM)][HB(C_6F_5)$_3$] **9**

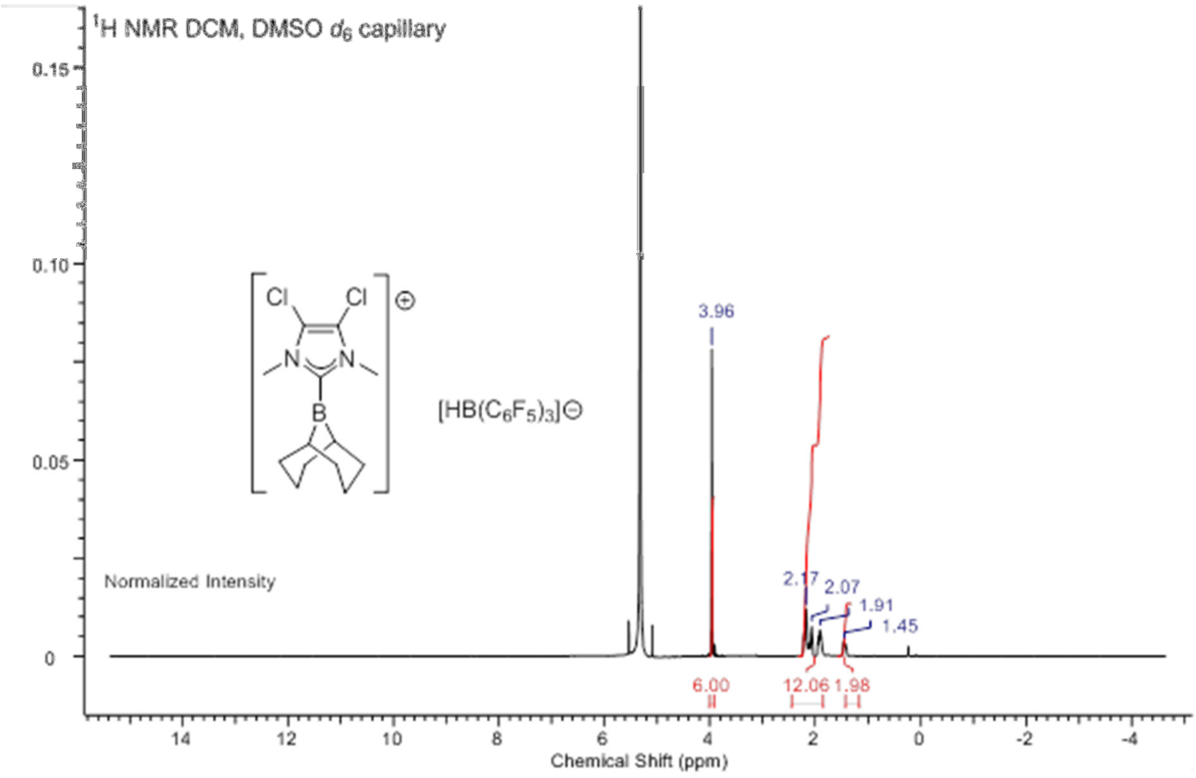

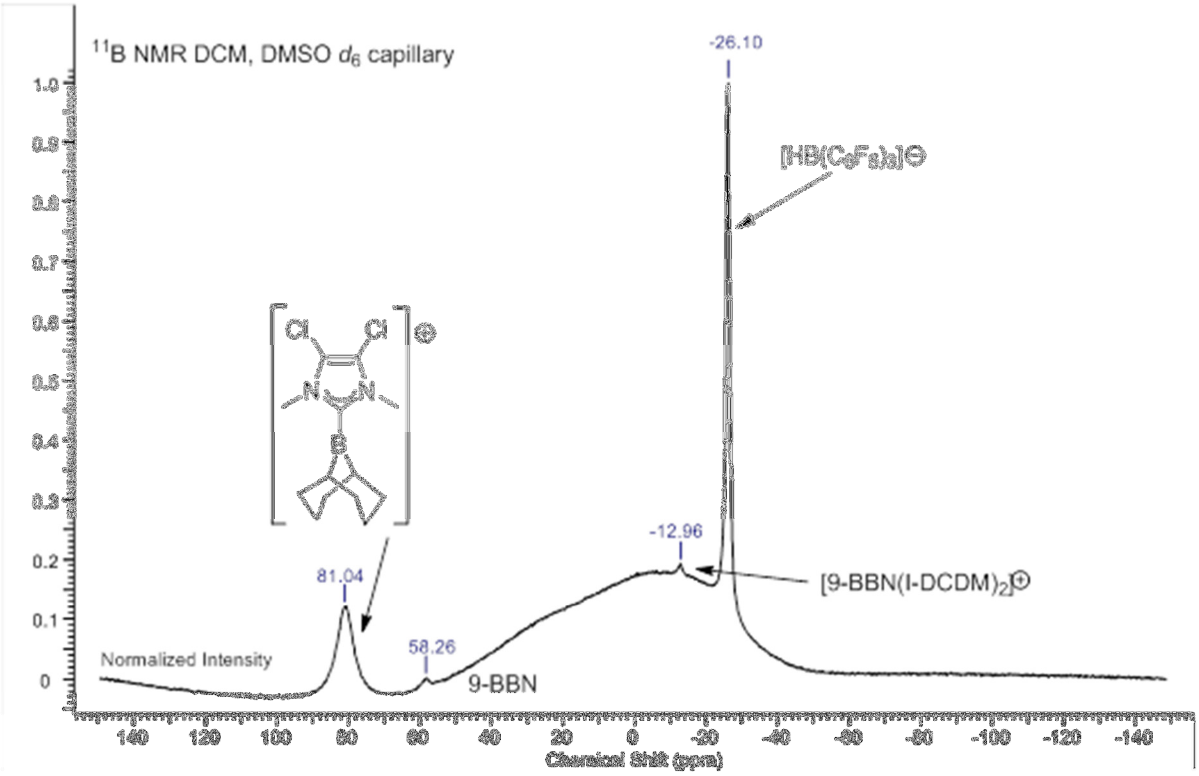

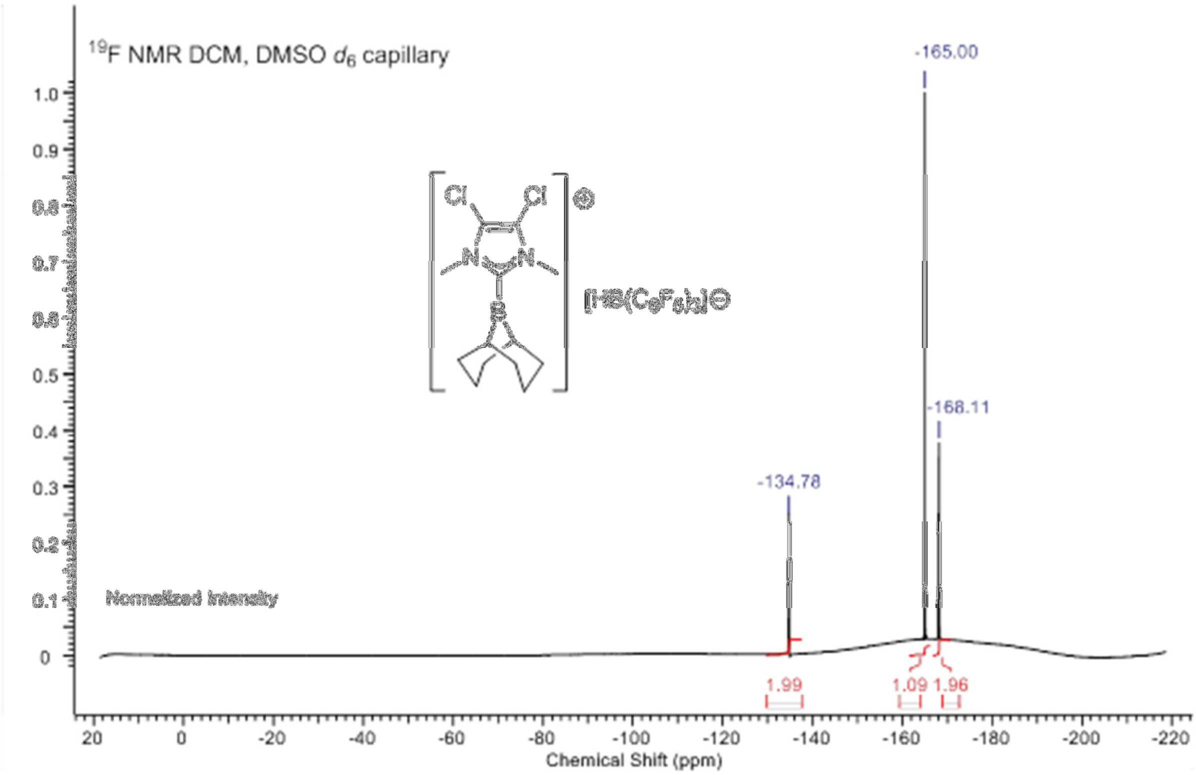

^{19}F NMR DCM, DMSO d_6 capillary
-165.00
-168.11
-134.78
Normalized Intensity
1.99
1.09
1.96
Chemical Shift (ppm)

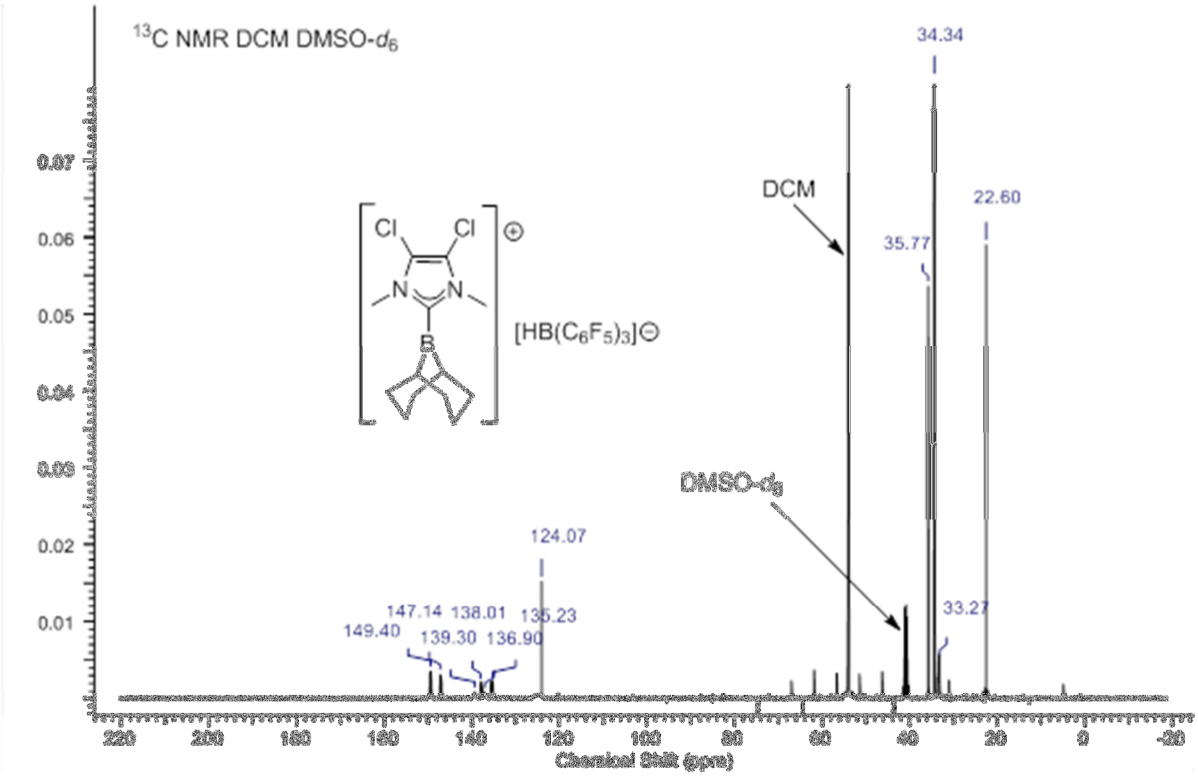

^{13}C NMR DCM DMSO-d_6
34.34
DCM
22.60
35.77
$[HB(C_6F_5)_3]^{\ominus}$
DMSO-d_6
124.07
147.14
138.01
135.23
149.40
139.30
136.90
33.27
Chemical Shift (ppm)

For the products **10a-n**, assignment of the vinyl protons is based on HSQC spectra, or due to the multiplicity of the resonance.

9-BBN(I-DCDM)(2-(*para*-tolyl)-*Z*-ethene) (**10a**)

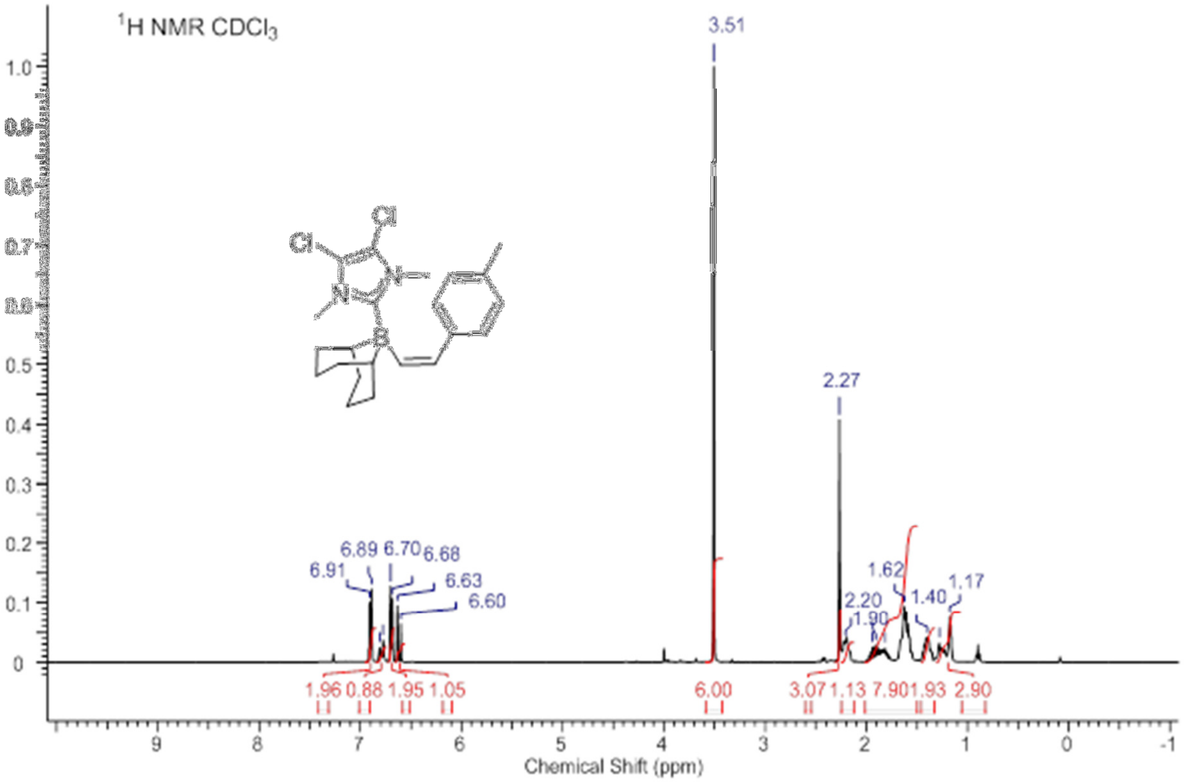

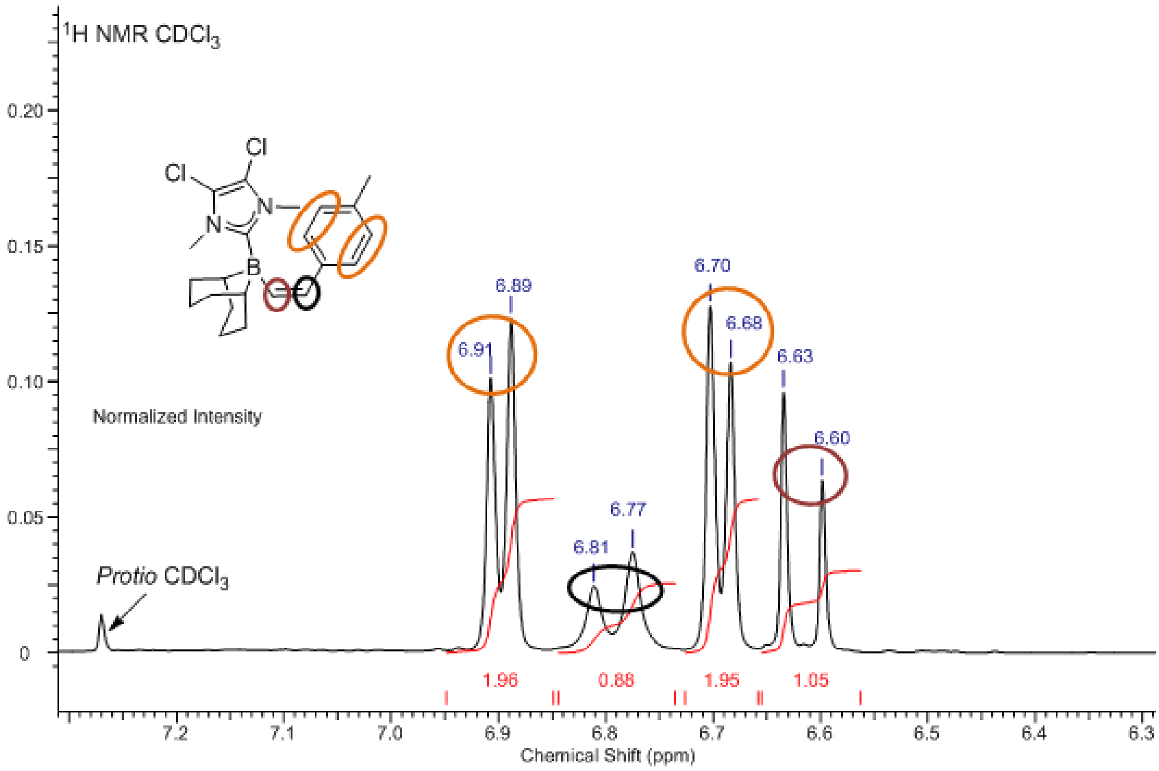

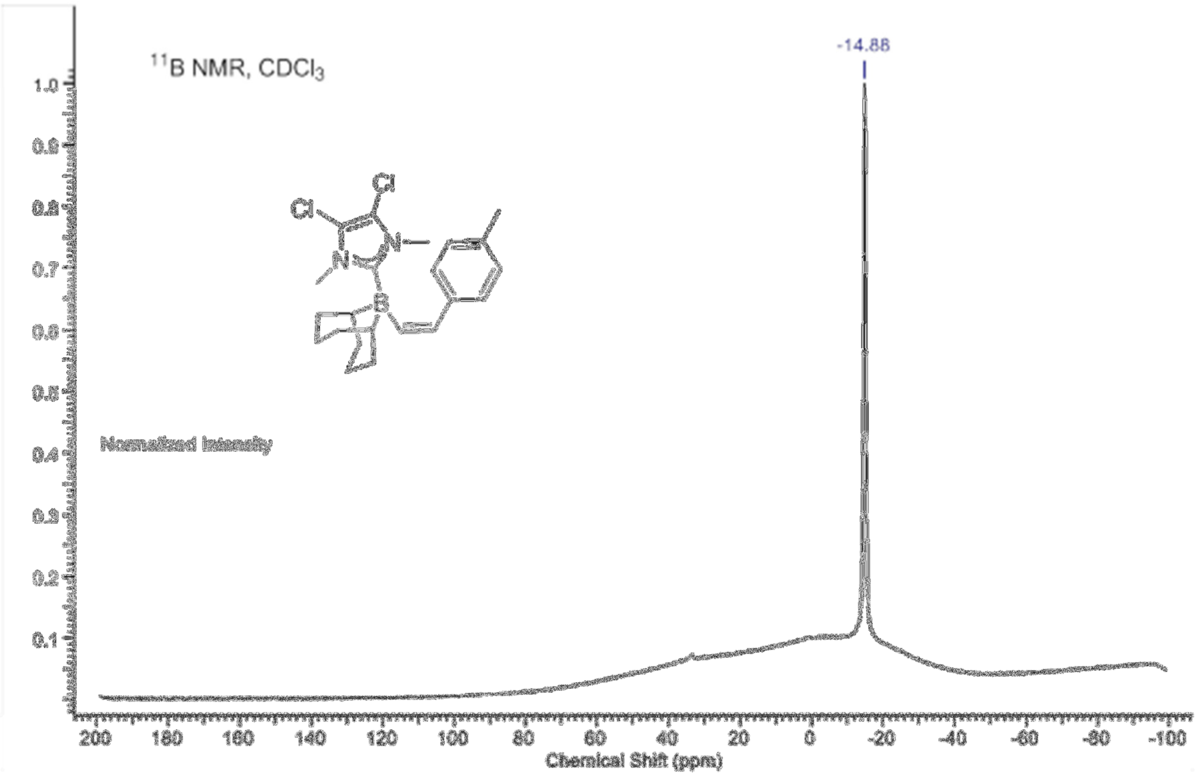

-14.88
11B NMR, CDCl3
Normalized Intensity
Chemical Shift (ppm)

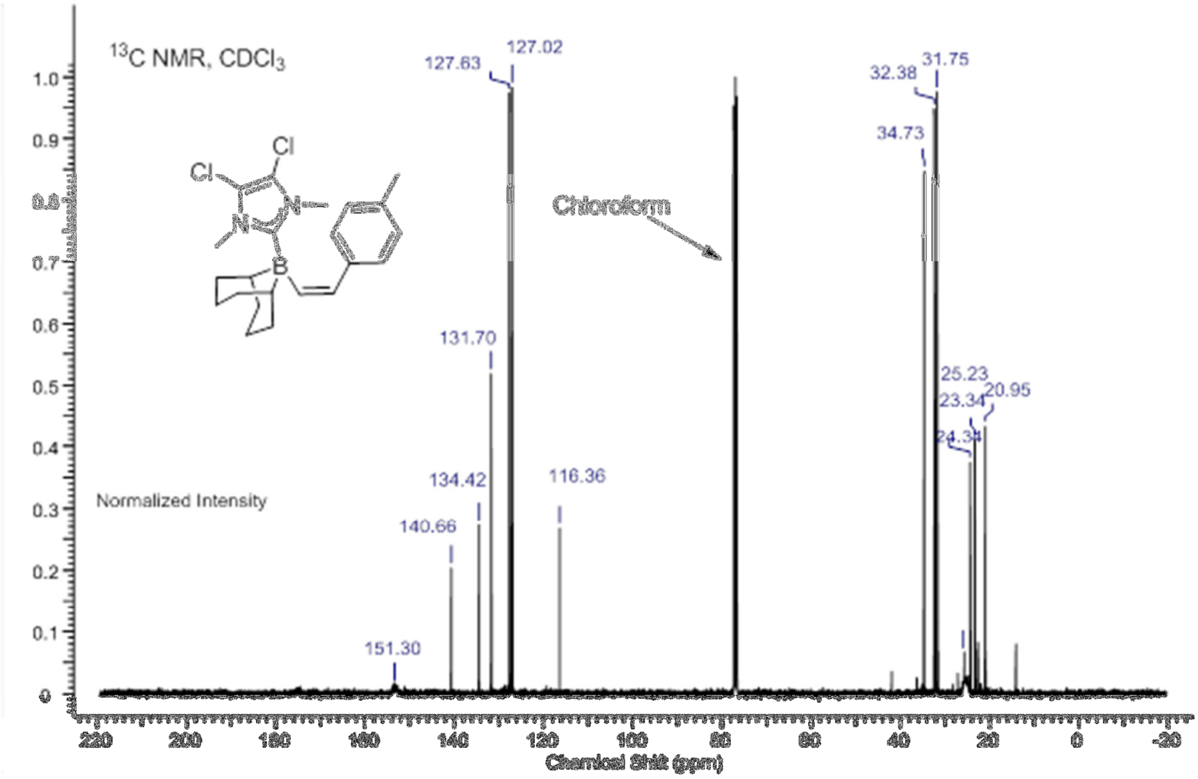

13C NMR, CDCl3
127.63
127.02
32.38
31.75
34.73
Chloroform
131.70
25.23
23.34
20.95
24.34
134.42
116.36
140.66
151.30
Normalized Intensity
Chemical Shift (ppm)

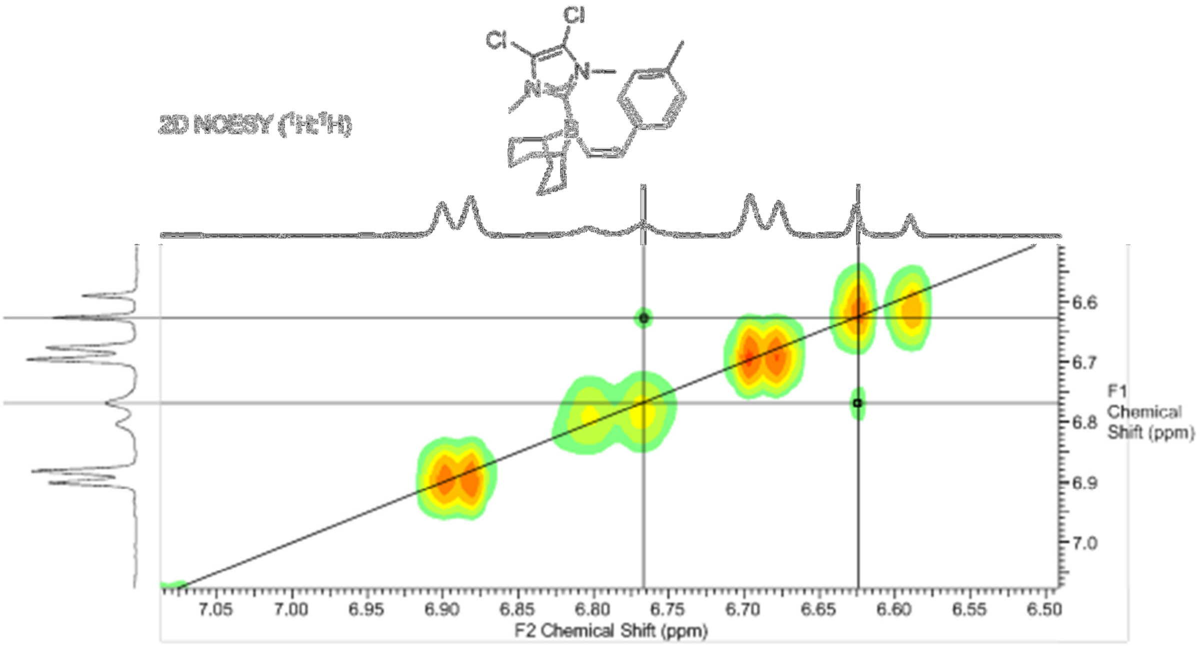
2D NOESY (1H,1H)
Cl
Cl
N
N
B
F1 Chemical Shift (ppm)
F2 Chemical Shift (ppm)
6.6
6.7
6.8
6.9
7.0
7.05
7.00
6.95
6.90
6.85
6.80
6.75
6.70
6.65
6.60
6.55
6.50

9-BBN(I-DCDM)(2-(phenyl)-*Z*-ethene) (**10b**)

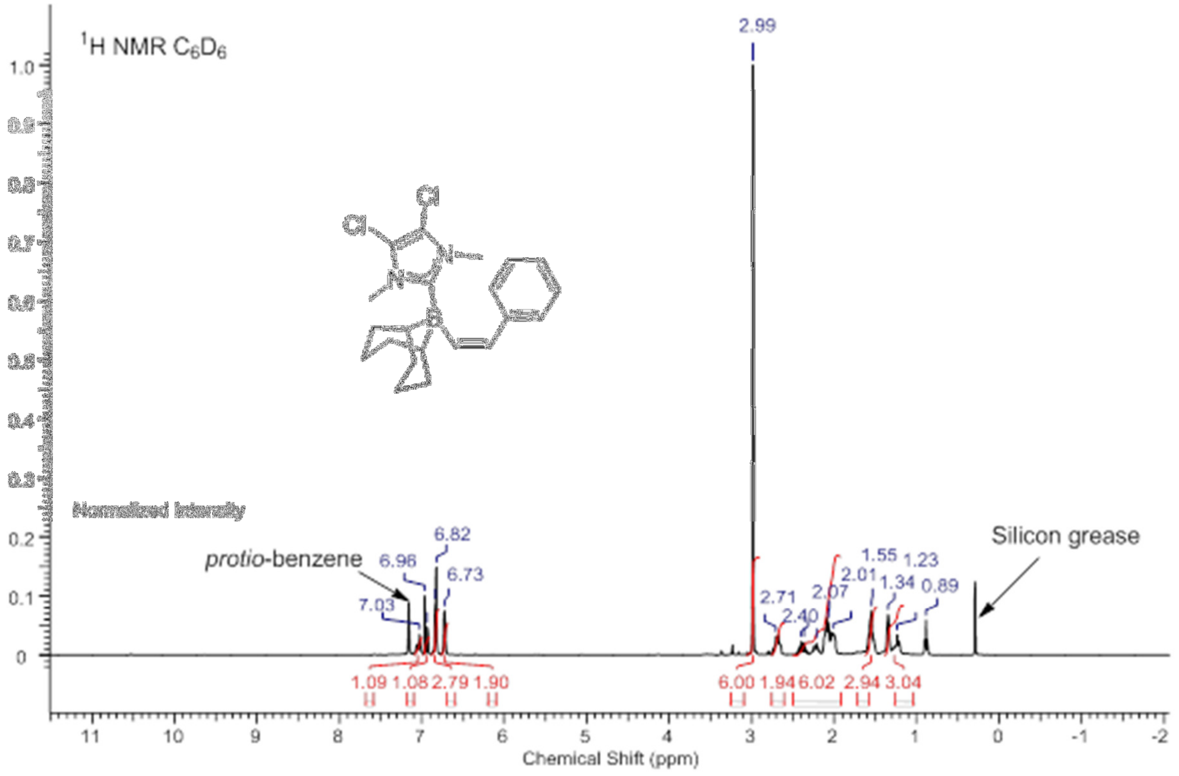

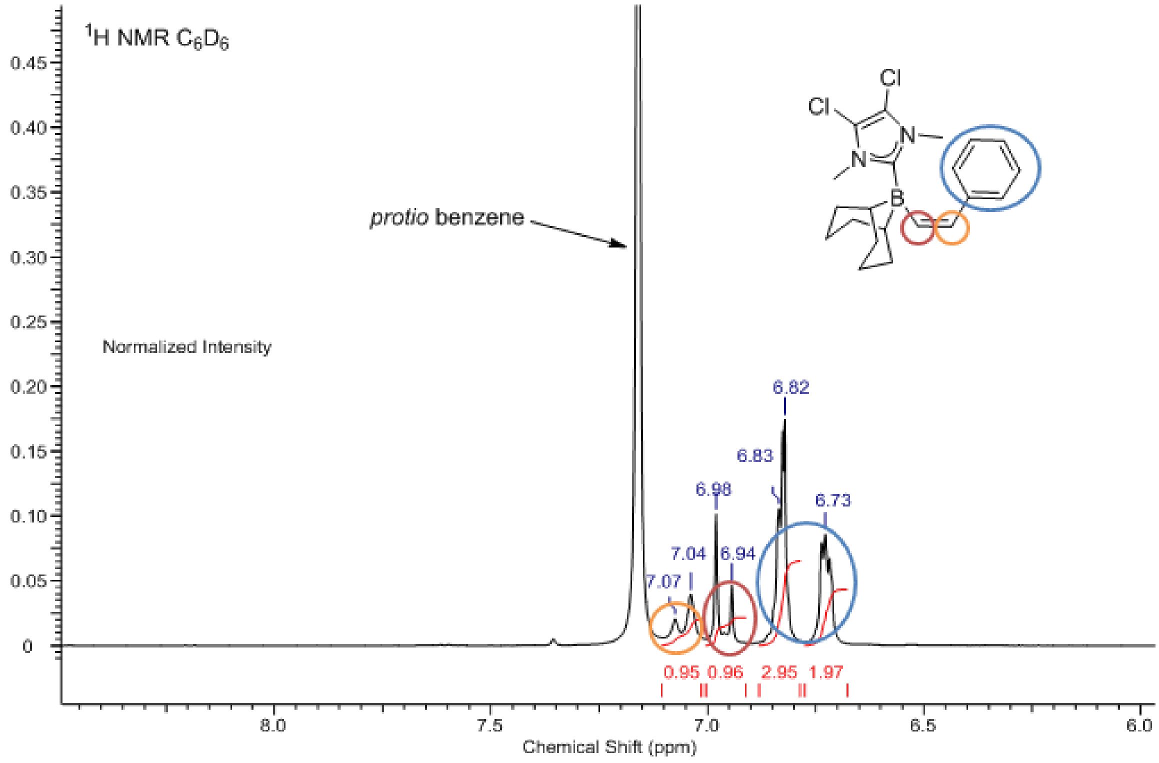

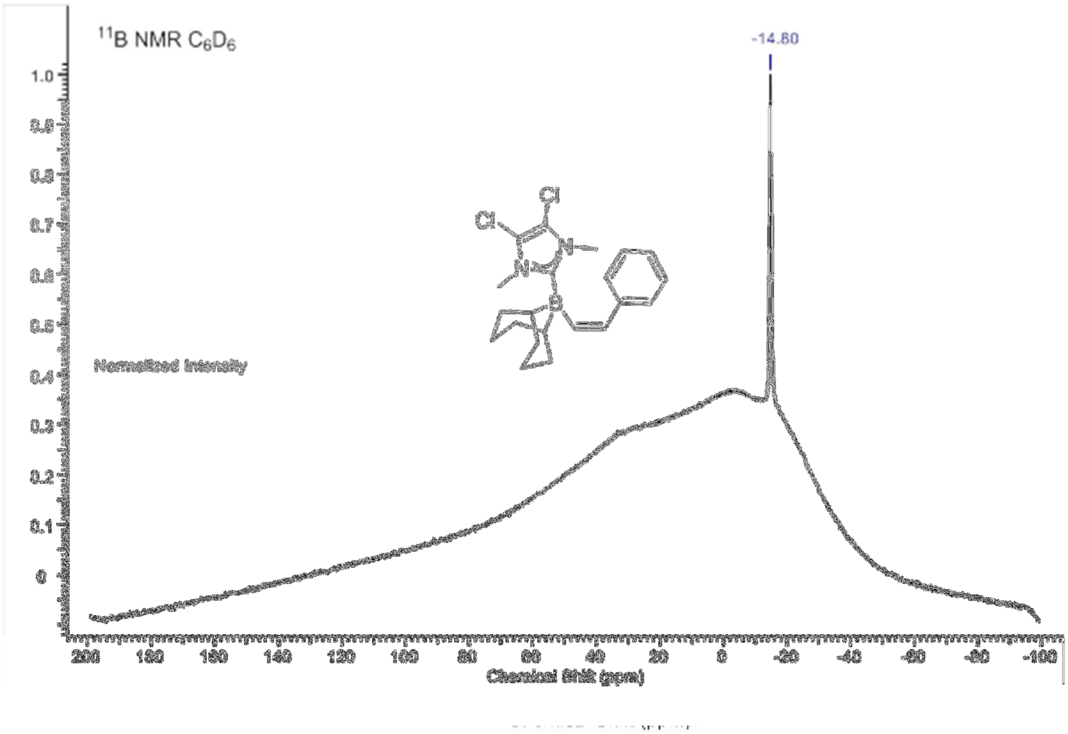

11B NMR C6D6
-14.80
Normalized Intensity
Chemical Shift (ppm)

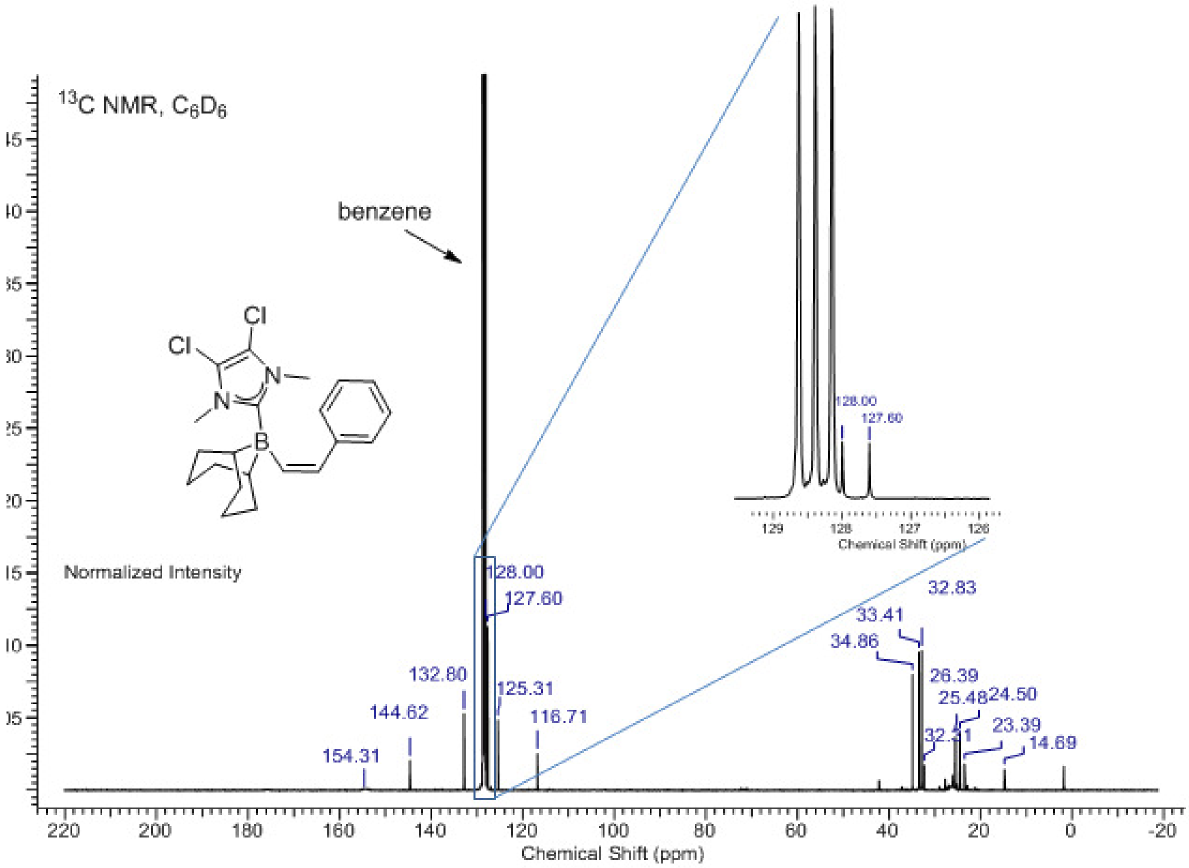

13C NMR, C6D6
benzene
Normalized Intensity
154.31
144.62
132.80
128.00
127.60
125.31
116.71
34.86
33.41
32.83
32.31
26.39
25.48
24.50
23.39
14.69
Chemical Shift (ppm)

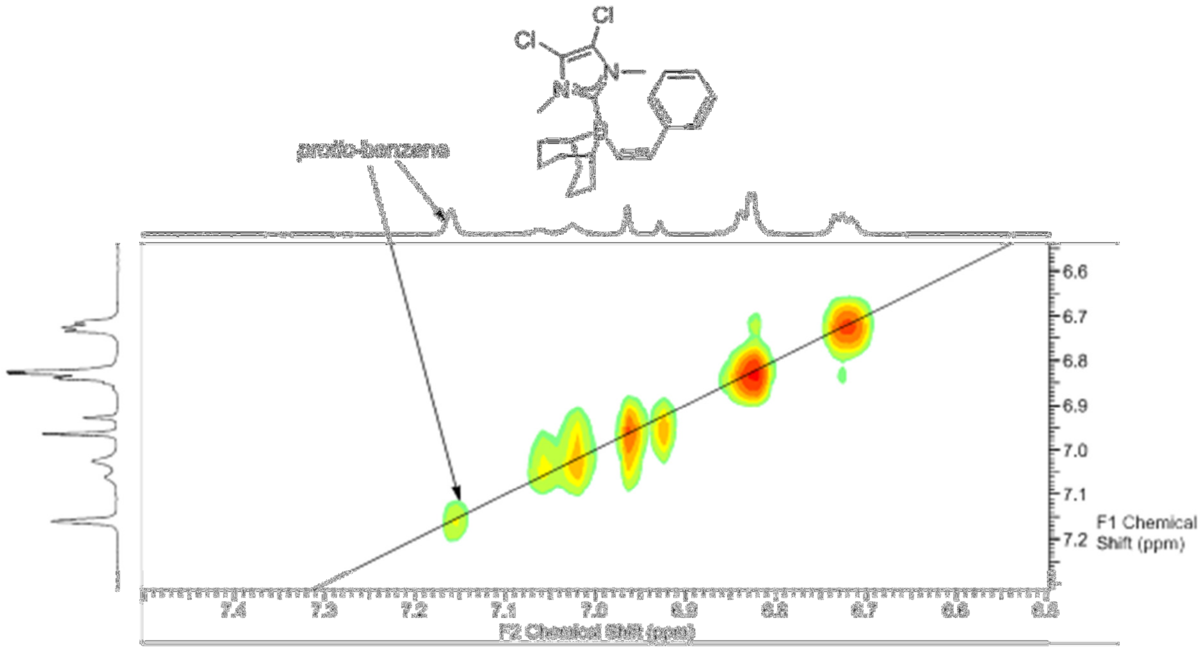

Cl
Cl
N
N
P
protio-benzene
6.6
6.7
6.8
6.9
7.0
7.1
7.2
F1 Chemical Shift (ppm)
7.4
7.3
7.2
7.1
7.0
6.9
6.8
6.7
6.6
6.5
F2 Chemical Shift (ppm)

9-BBN(I-DCDM)(2-(*para*-anisole)-*Z*-ethene) (**10c**)

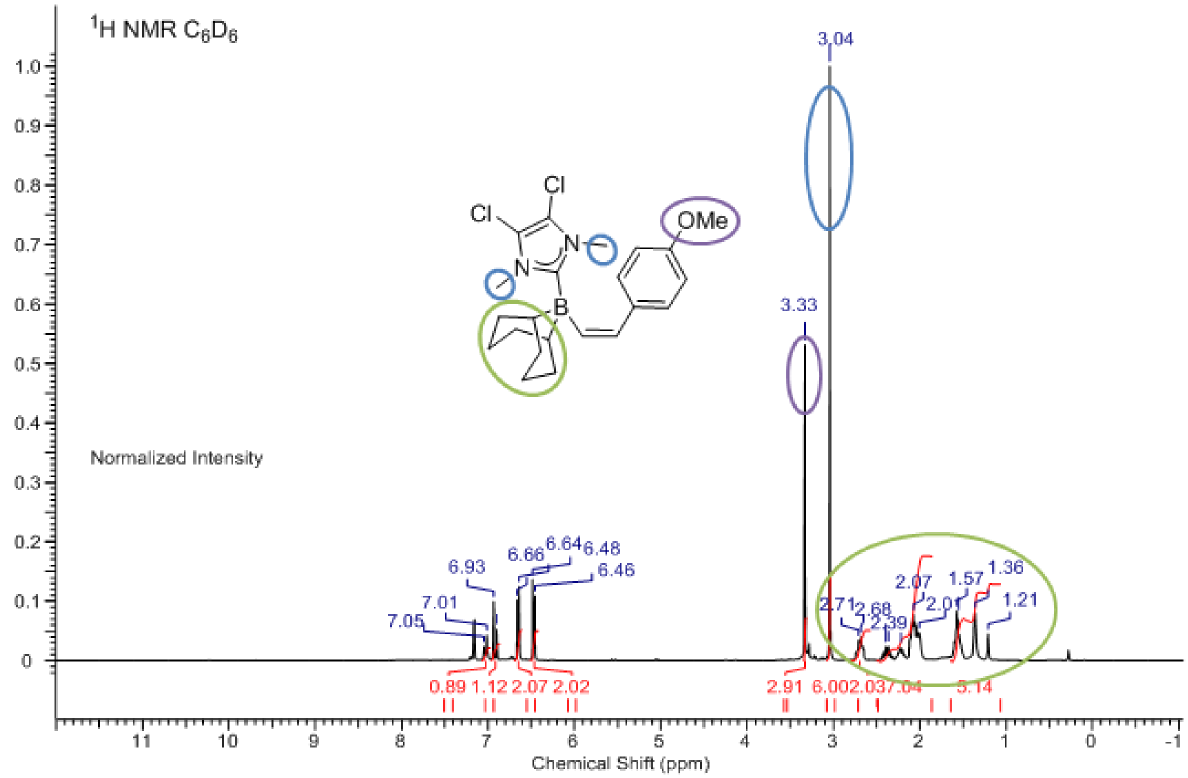

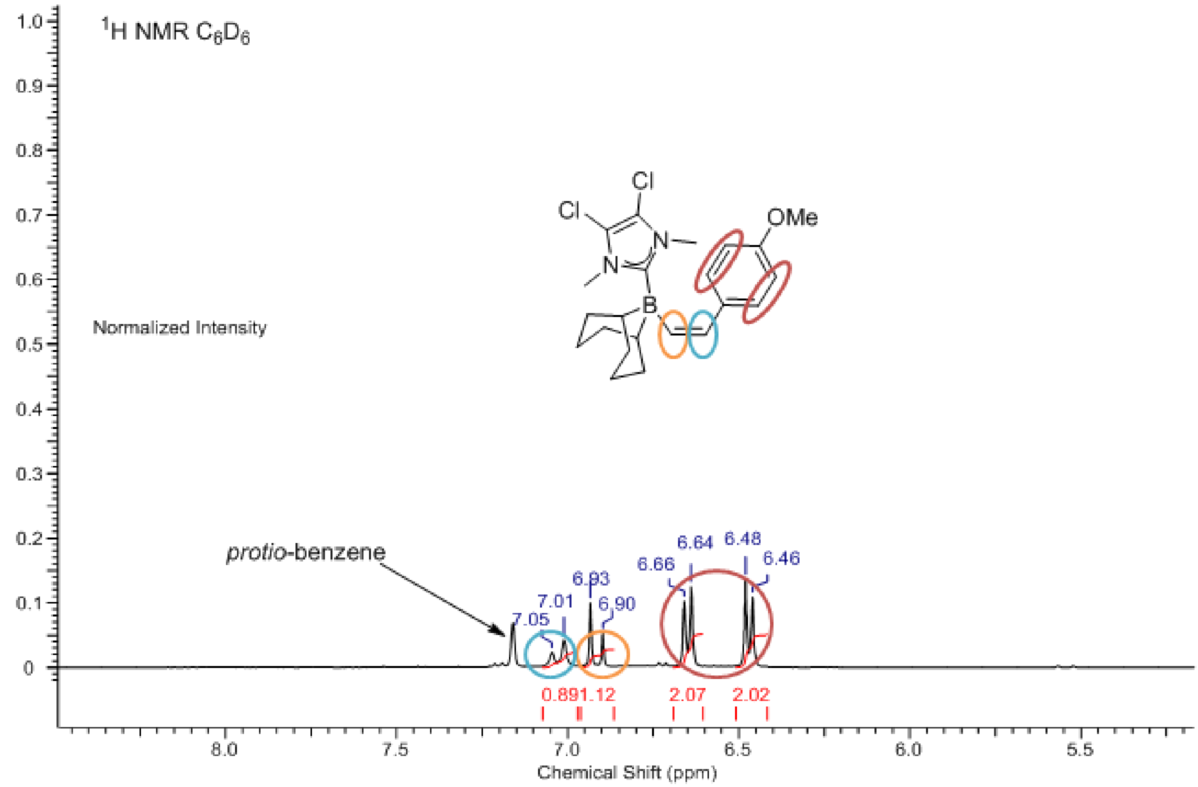

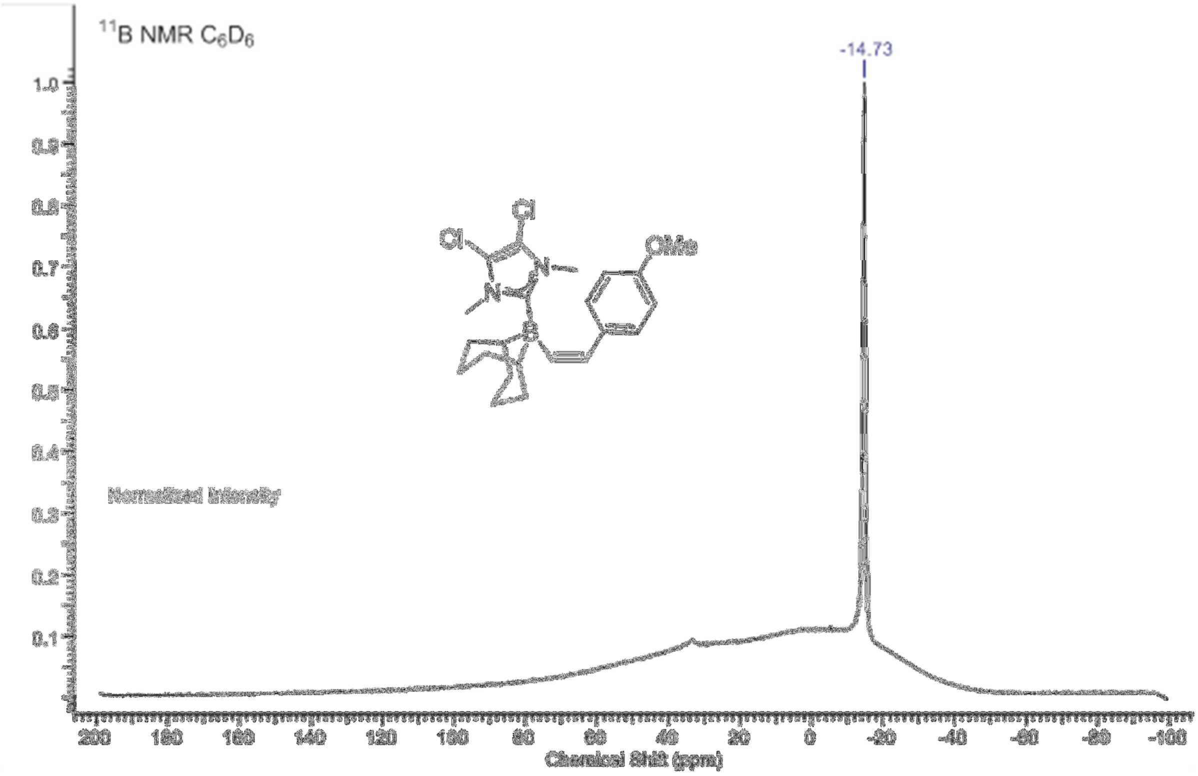
11B NMR C6D6
-14.73
Cl
Cl
N
N
B
OMe
Normalized Intensity
Chemical Shift (ppm)

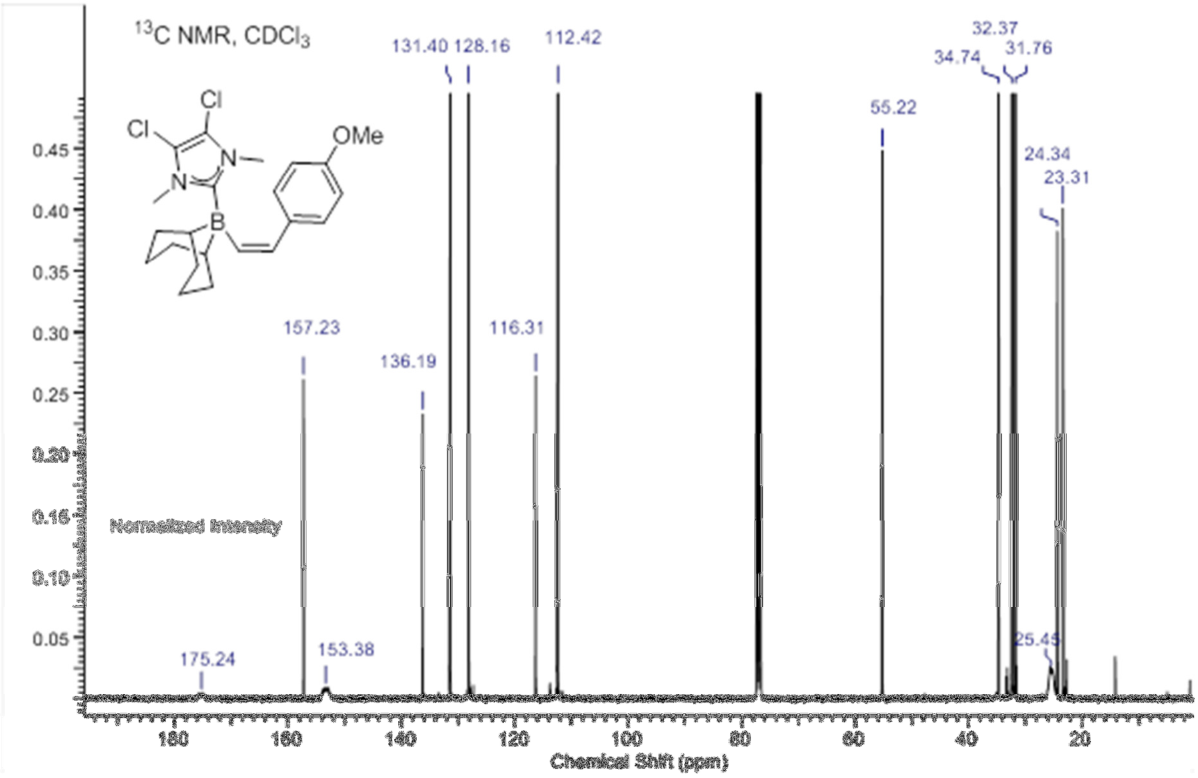
13C NMR, CDCl3
131.40 128.16
112.42
32.37
34.74
31.76
55.22
24.34
23.31
157.23
136.19
116.31
175.24
153.38
25.45
Cl
Cl
N
N
B
OMe
Normalized Intensity
Chemical Shift (ppm)

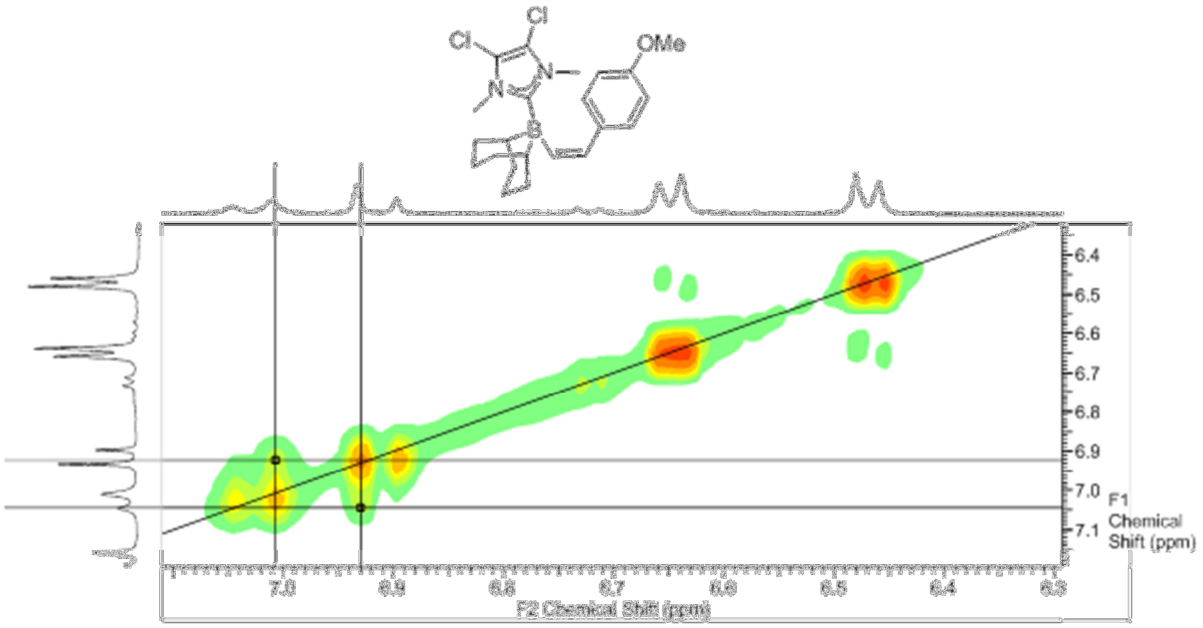
Cl
Cl
OMe
N
N
S
6.4
6.5
6.6
6.7
6.8
6.9
7.0
7.1
F1 Chemical Shift (ppm)
7.0
6.9
6.8
6.7
6.6
6.5
6.4
6.3
F2 Chemical Shift (ppm)

In-situ NMR spectra (with added mesitylene as internal standard) for 9-BBN(I-DCDM)(2-(*para*-(*N,N*-dimethyl)aniline)-*Z*-ethene) (**10d**)

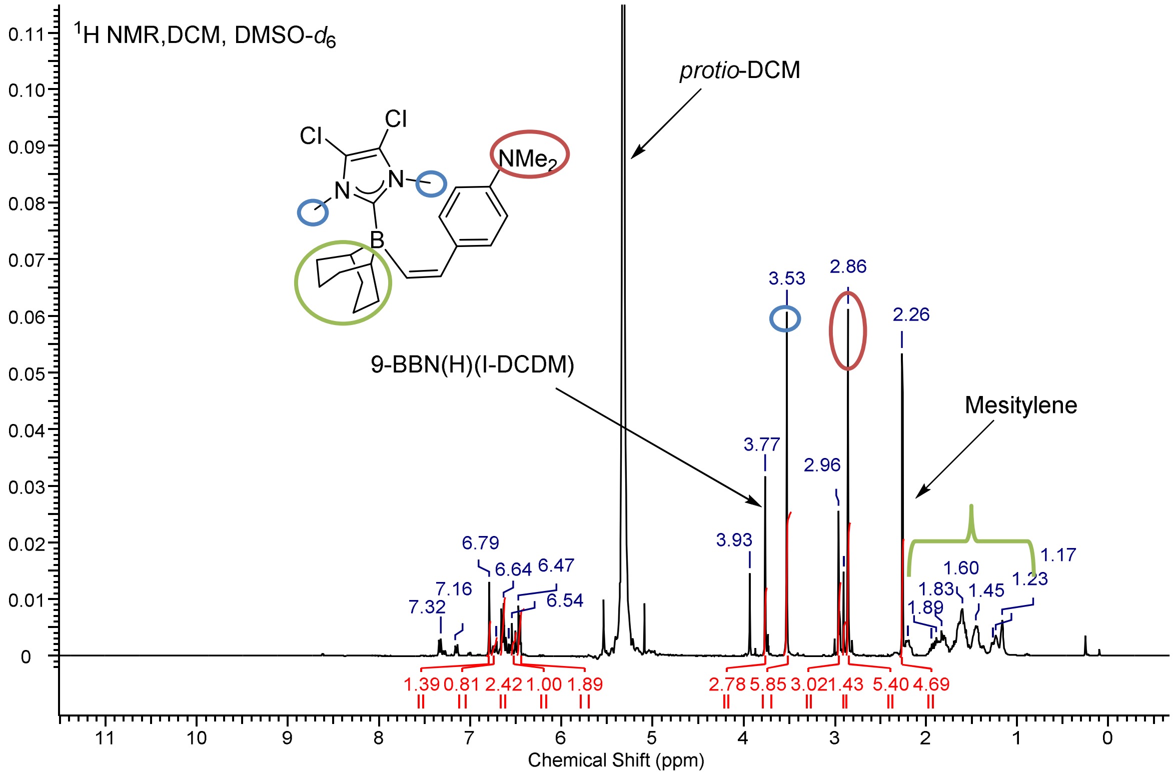

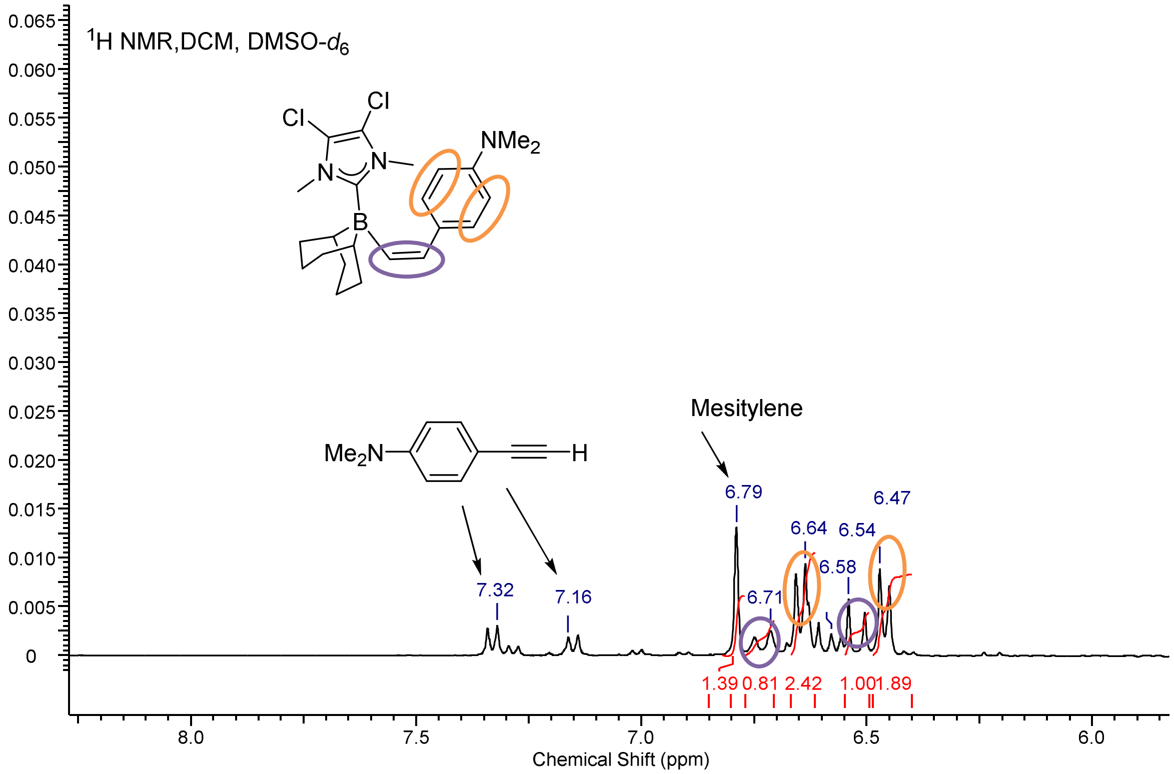

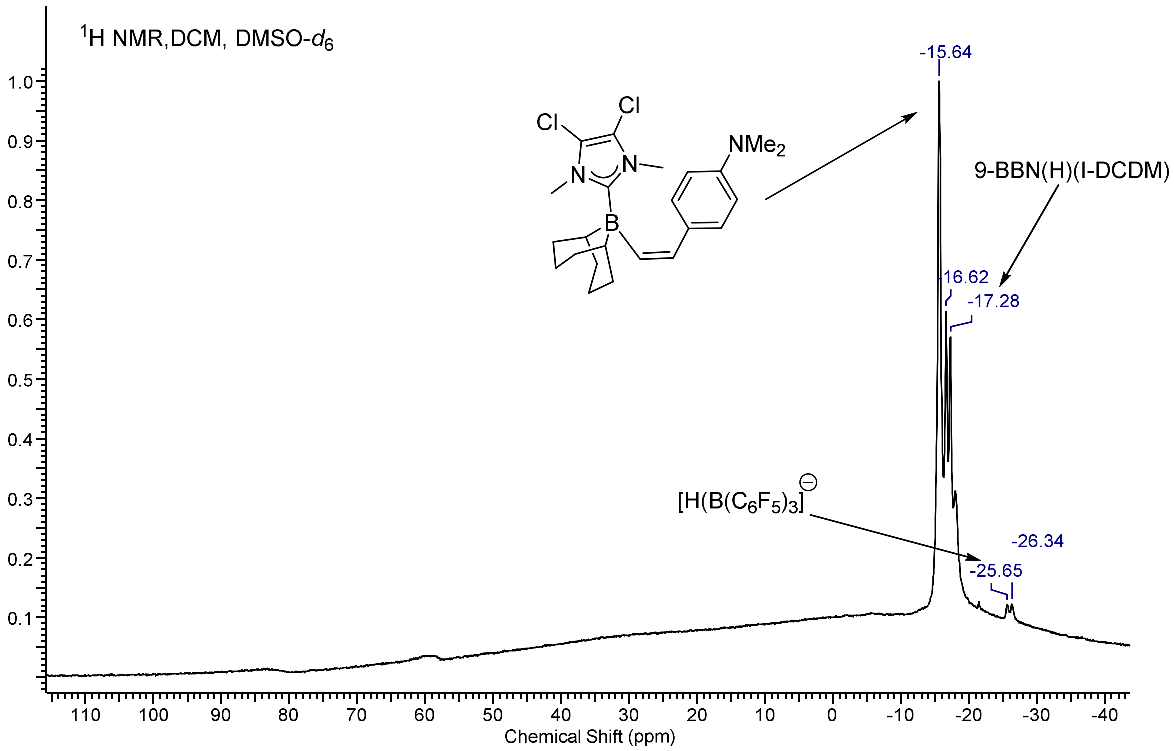

The sample proved extremely sensitive to protodeboronation on attempted isolation leading to formation of significant *p*-NMe_2-styrene. The NOESY was recorded in C_6D_6 for better separation of relevant vinyl resonances.

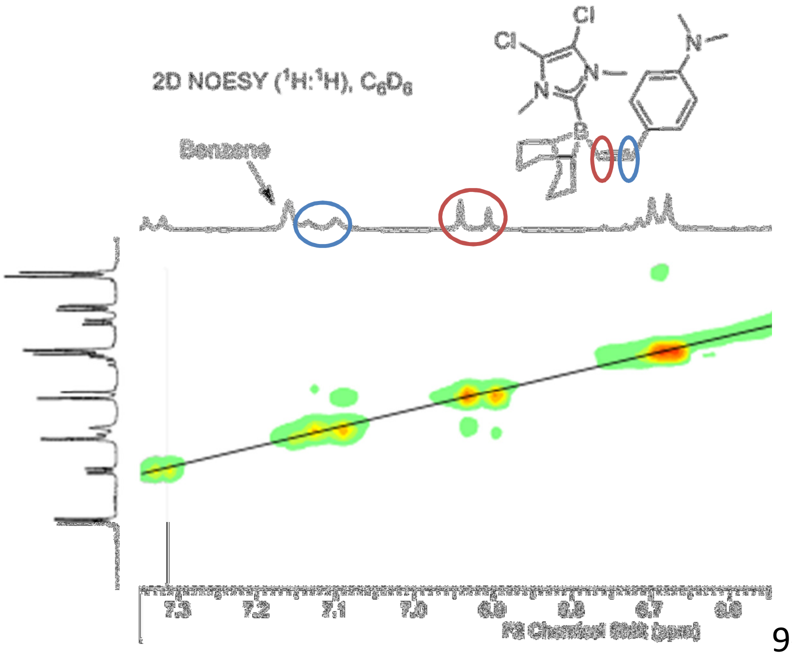

9-BBN(I-DCDM)(2-(*para*-(*α,α,α*-trifluoromethyl)phenyl)-*Z*-ethene) (**10e**)

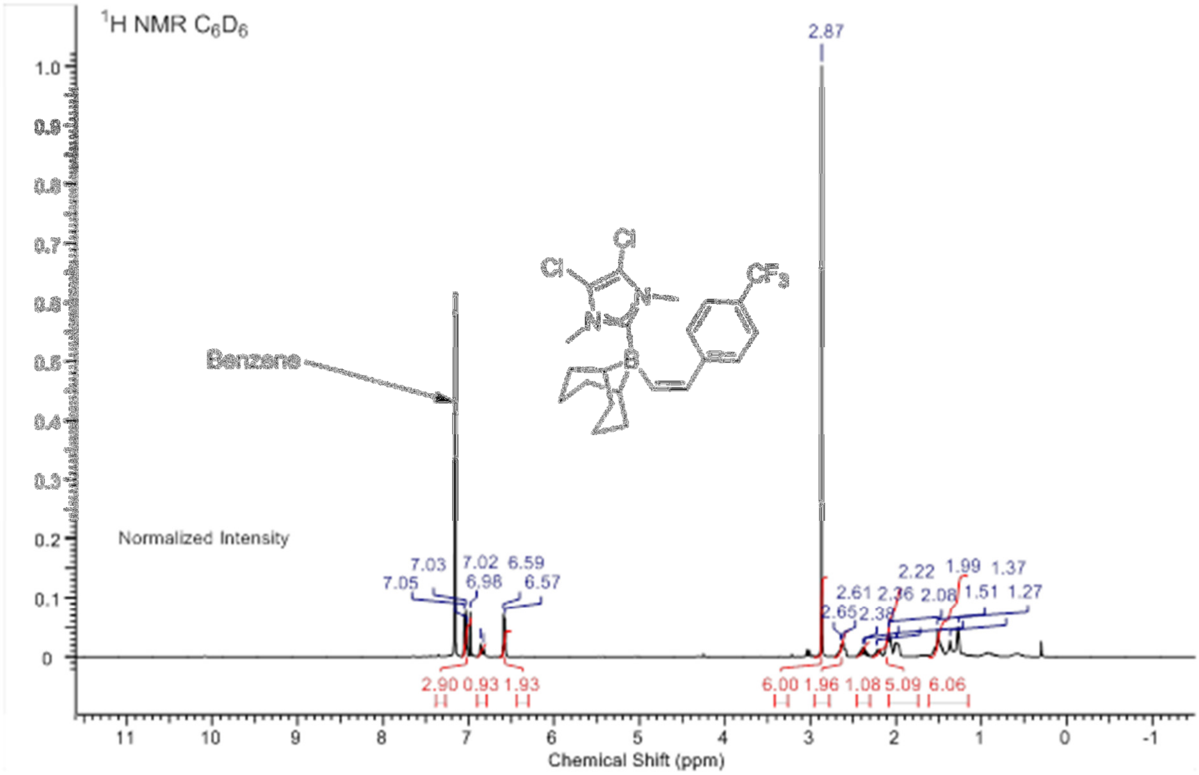

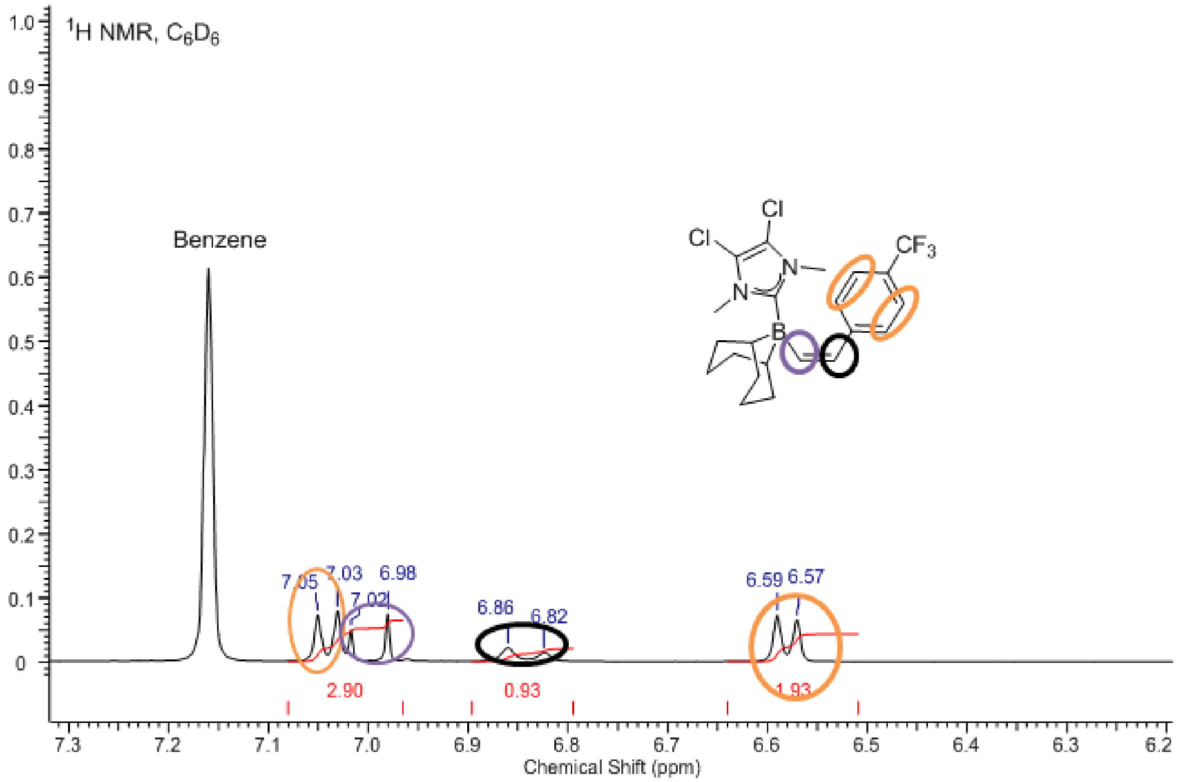

^{1}H NMR, C_6D_6
Benzene
Cl
Cl
N
N
B
CF_3
7.05
7.03
7.02
6.98
6.86
6.82
6.59
6.57
2.90
0.93
1.93
Chemical Shift (ppm)

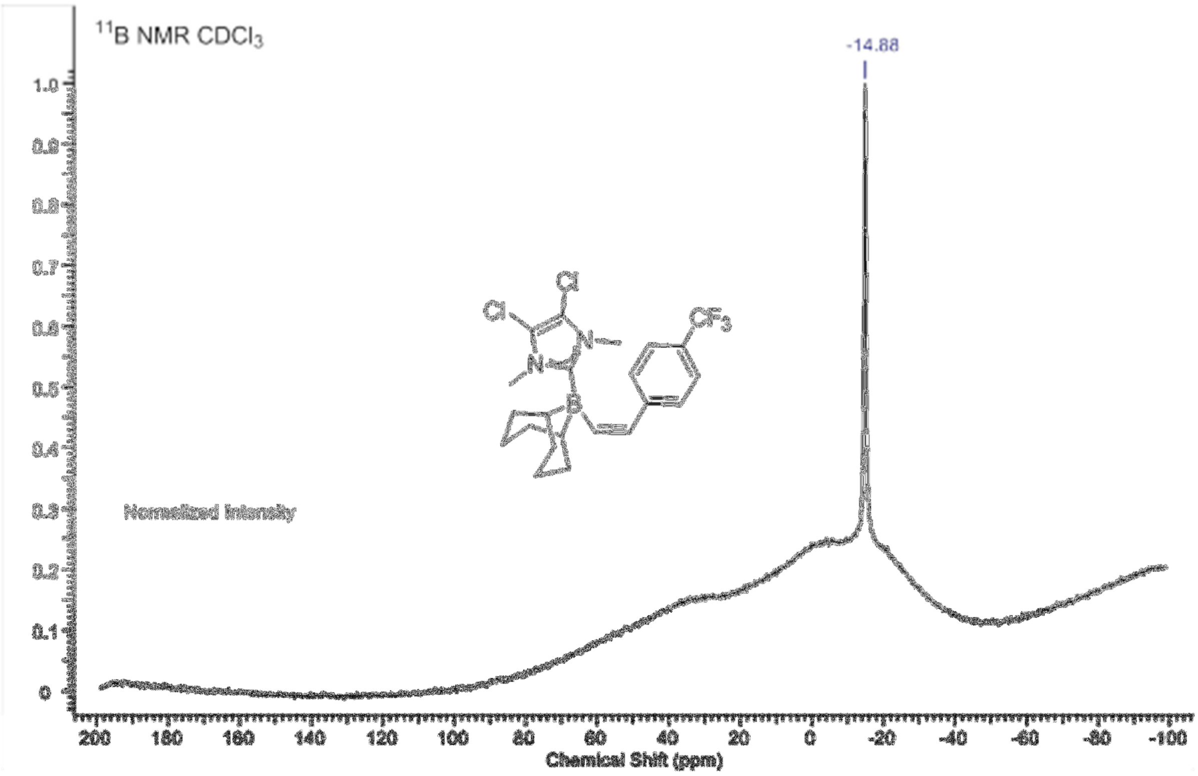
11B NMR CDCl3
-14.88
Normalized Intensity
Chemical Shift (ppm)
Cl
Cl
N
N
B
CF3

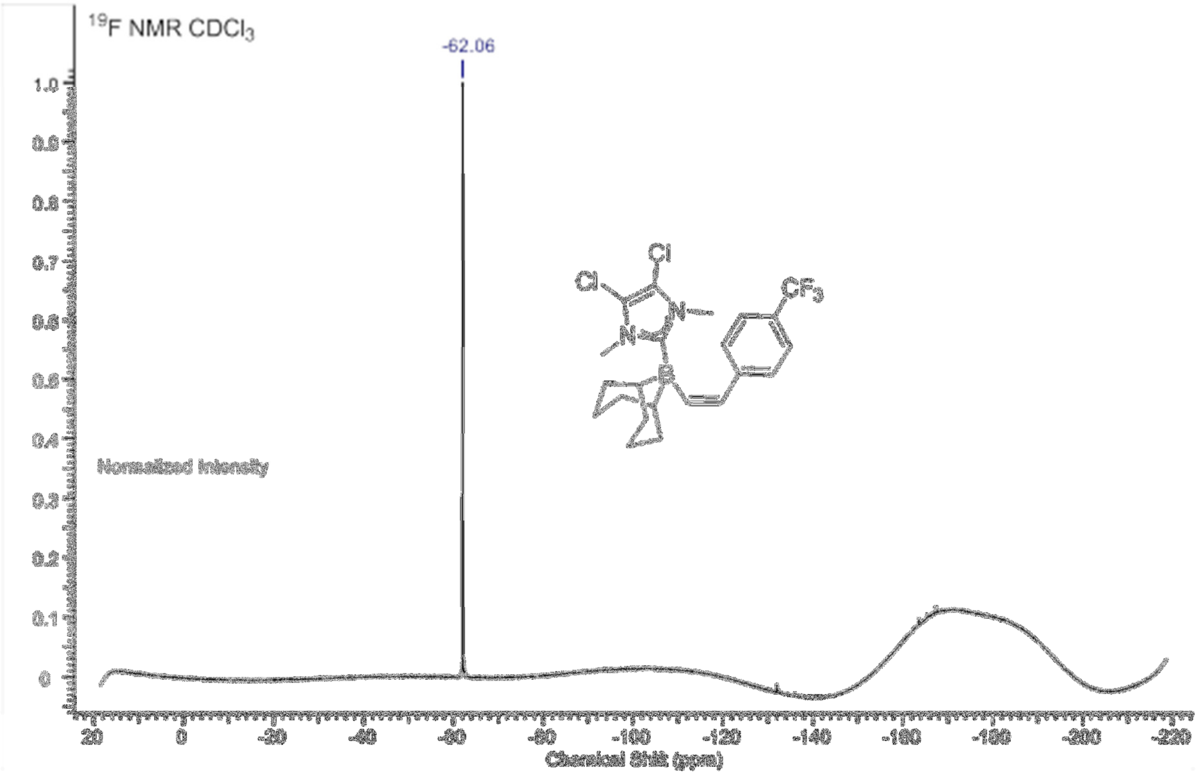
19F NMR CDCl3
-62.06
Normalized Intensity
Chemical Shift (ppm)
Cl
Cl
N
N
B
CF3

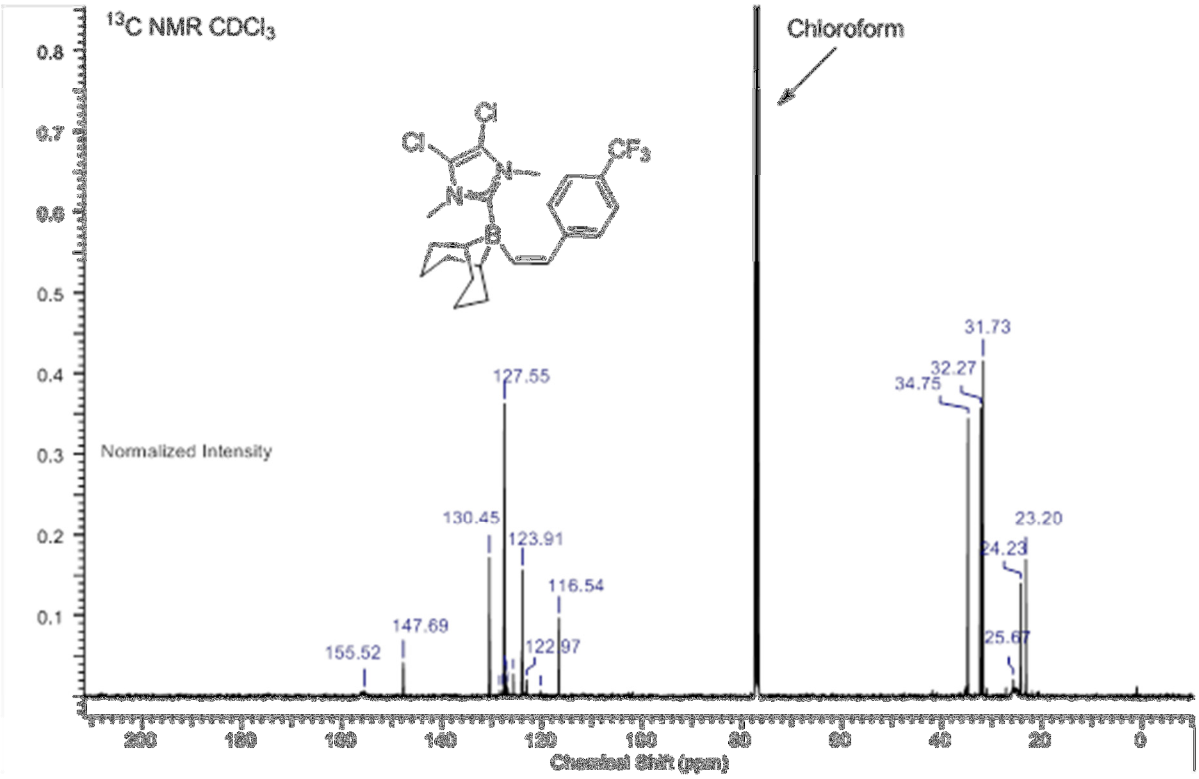

13C NMR CDCl3
Chloroform
Normalized Intensity
Chemical Shift (ppm)
155.52
147.69
130.45
127.55
123.91
122.97
116.54
34.75
32.27
31.73
25.67
24.23
23.20
CF3
Cl
N
B

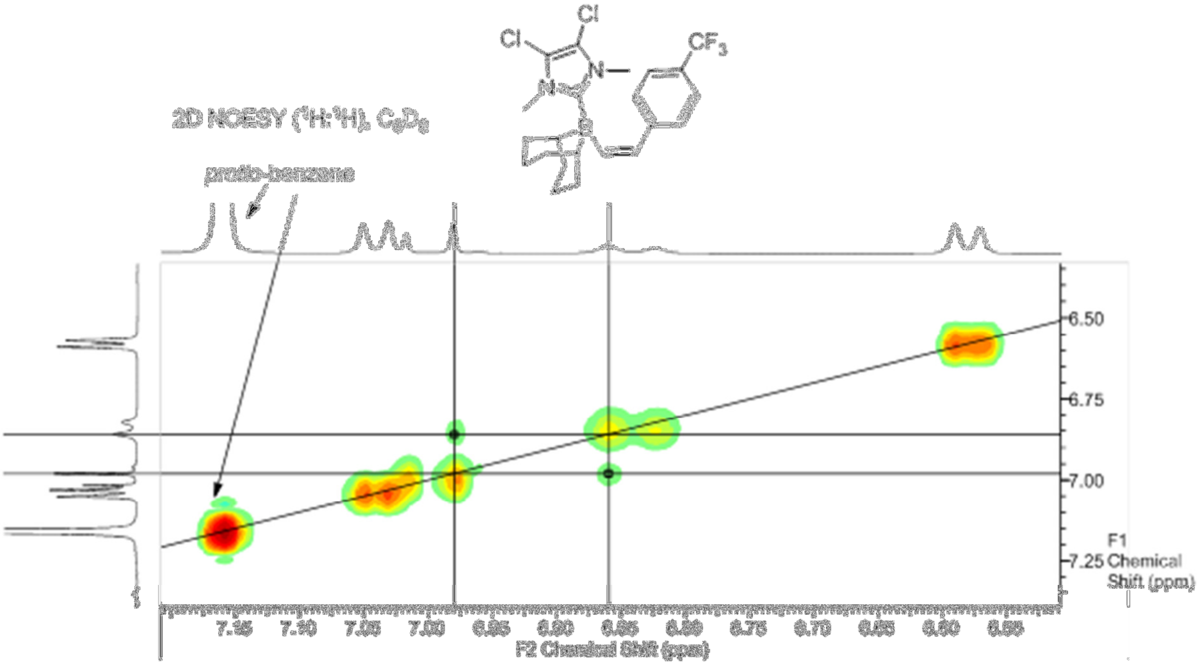

2D NOESY (1H-1H), C6D6
protio-benzene
F2 Chemical Shift (ppm)
F1 Chemical Shift (ppm)
CF3
Cl
N
B

9-BBN(I-DCDM)(2-(*para*-chlorophenyl)-*Z*-ethene) (**10f**)

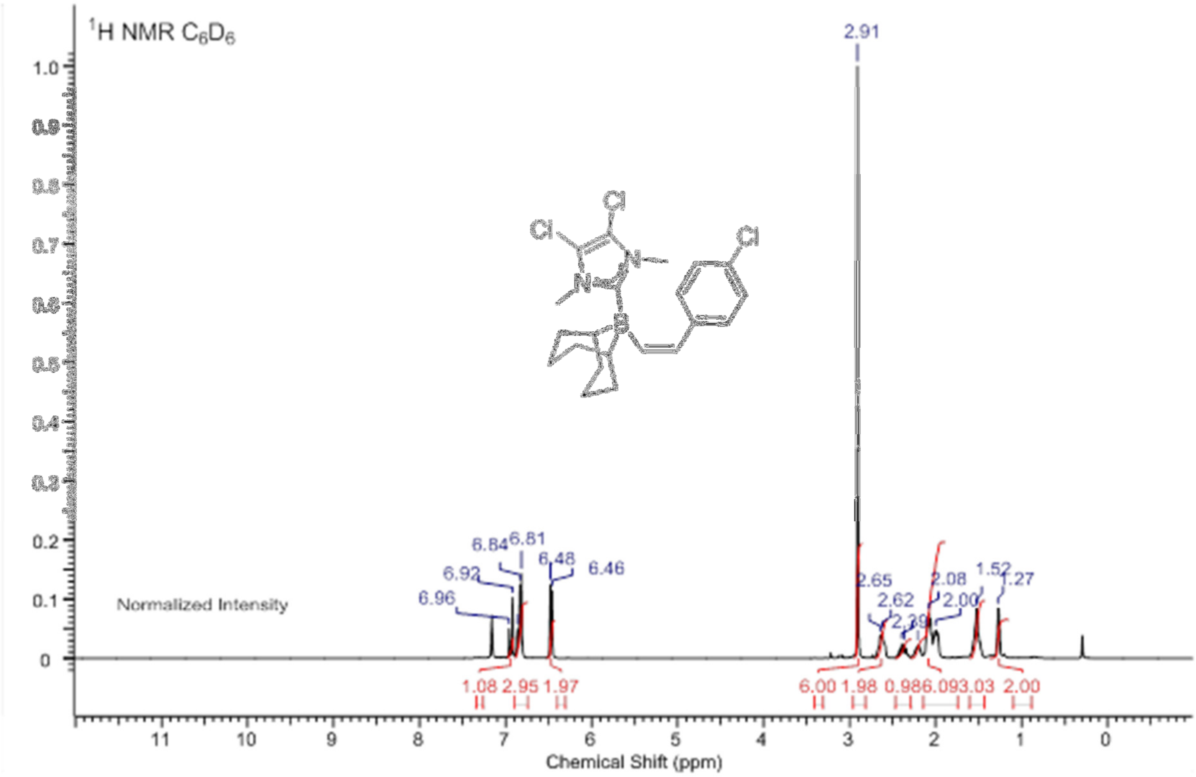

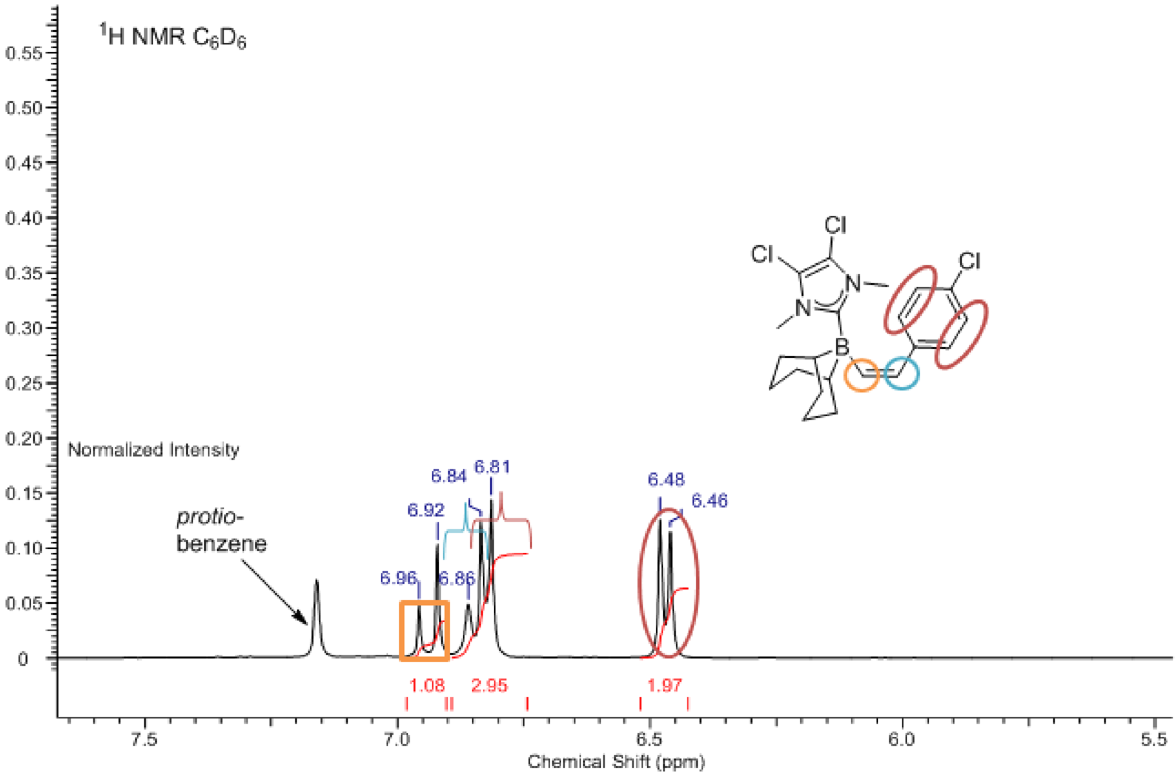

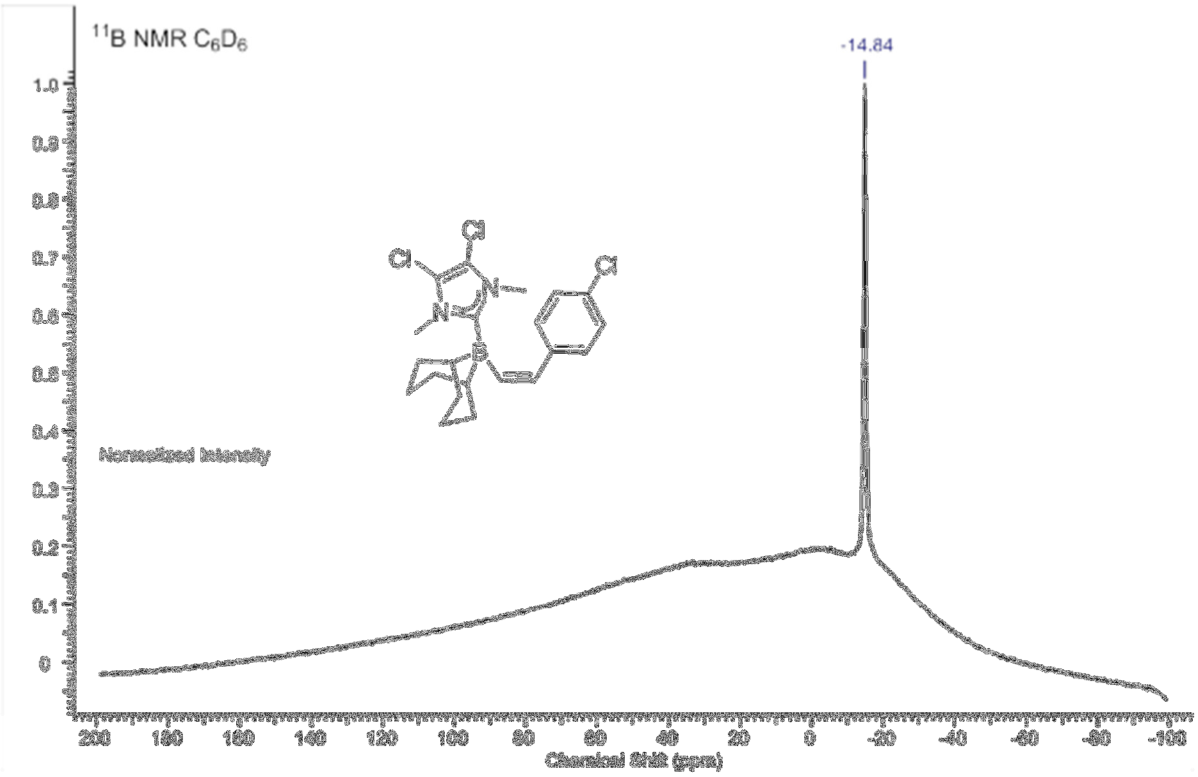

^{11}B NMR C_6D_6
-14.84
Normalized Intensity
Chemical Shift (ppm)

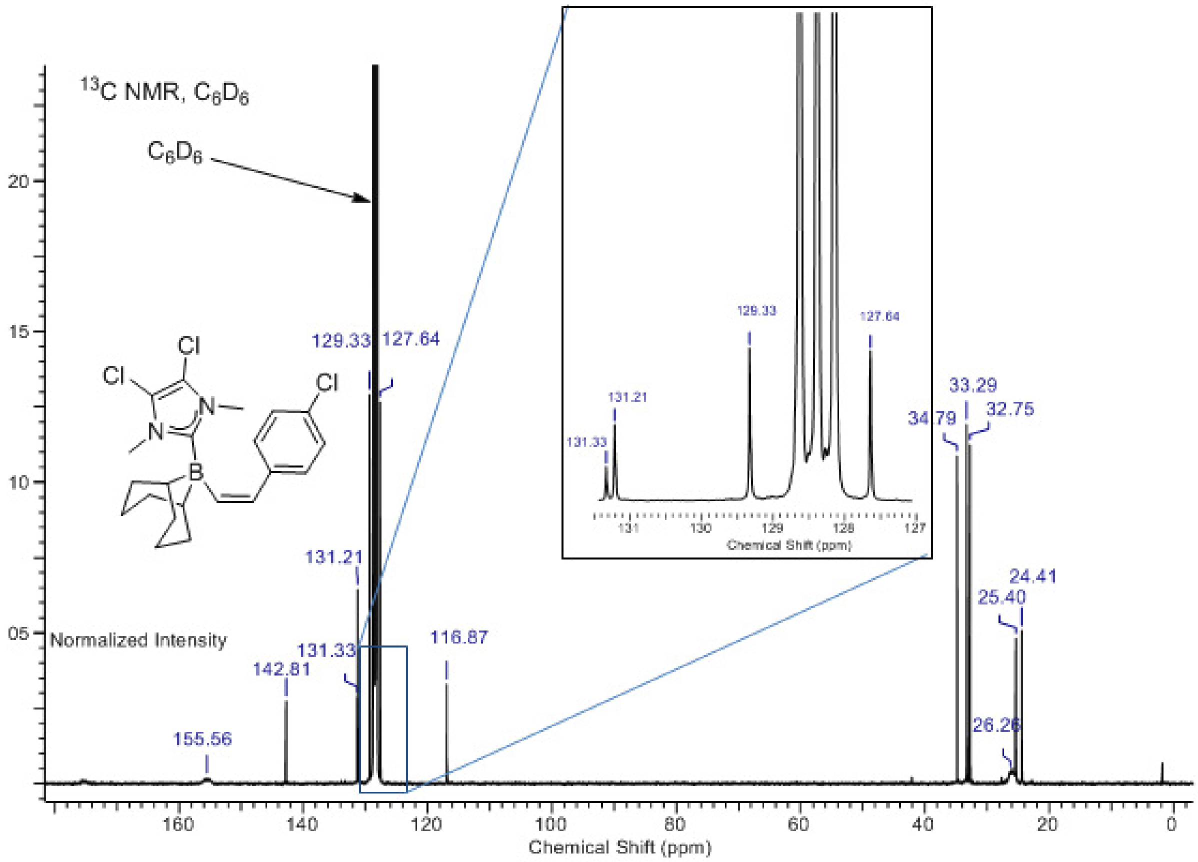

^{13}C NMR, C_6D_6
C_6D_6
Normalized Intensity
155.56
142.81
131.33
131.21
129.33
127.64
116.87
34.79
33.29
32.75
26.26
25.40
24.41
Chemical Shift (ppm)

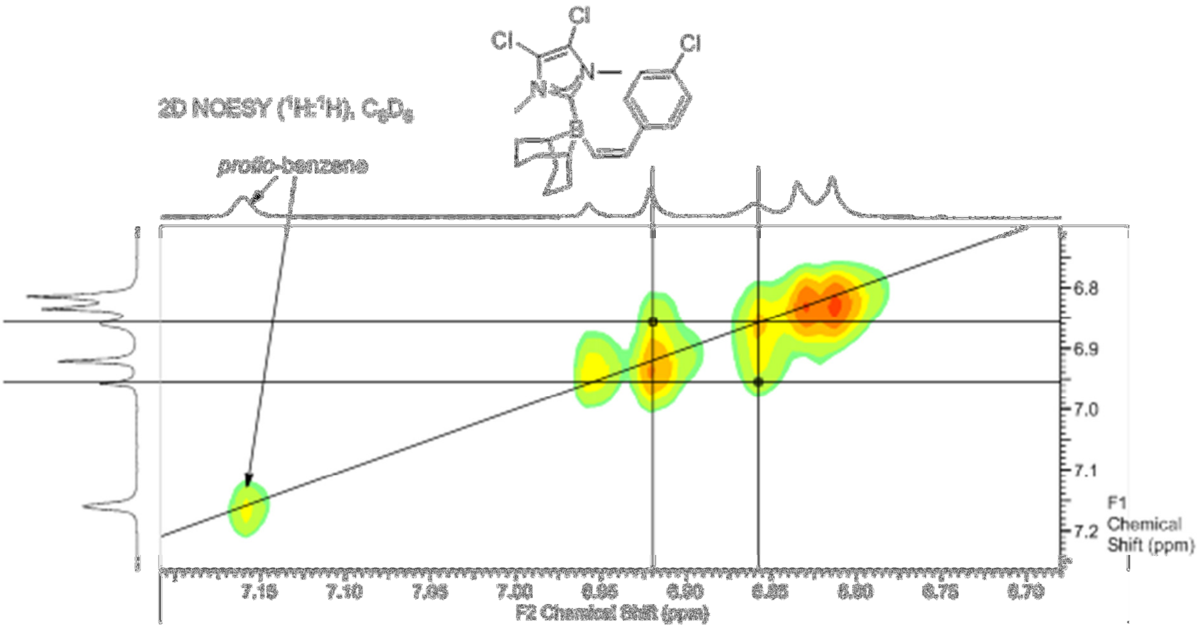
2D NOESY (^1H:^1H), C_6D_6
protio-benzene
Cl
Cl
Cl
N
N
B
7.15
7.10
7.05
7.00
6.95
6.90
6.85
6.80
6.75
6.70
F2 Chemical Shift (ppm)
6.8
6.9
7.0
7.1
7.2
F1 Chemical Shift (ppm)

9-BBN(I-DCDM)(2-(*para*-fluorophenyl)-*Z*-ethene) (**10g**)

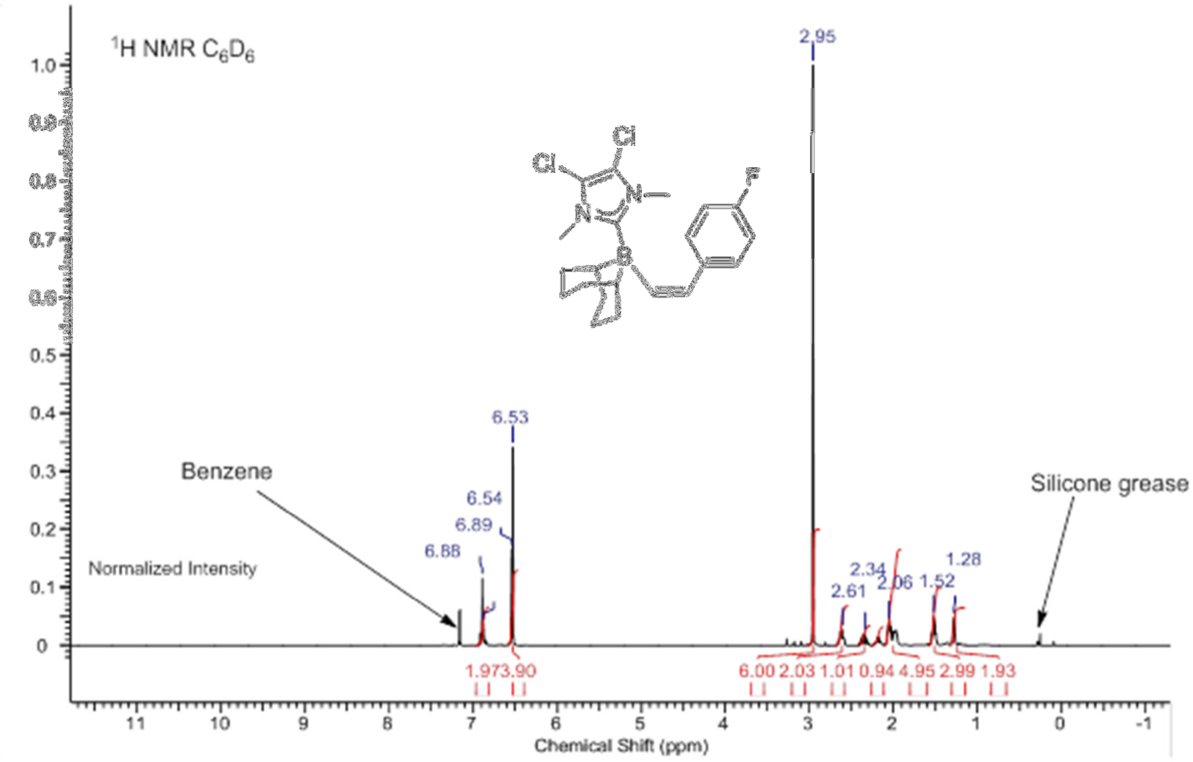

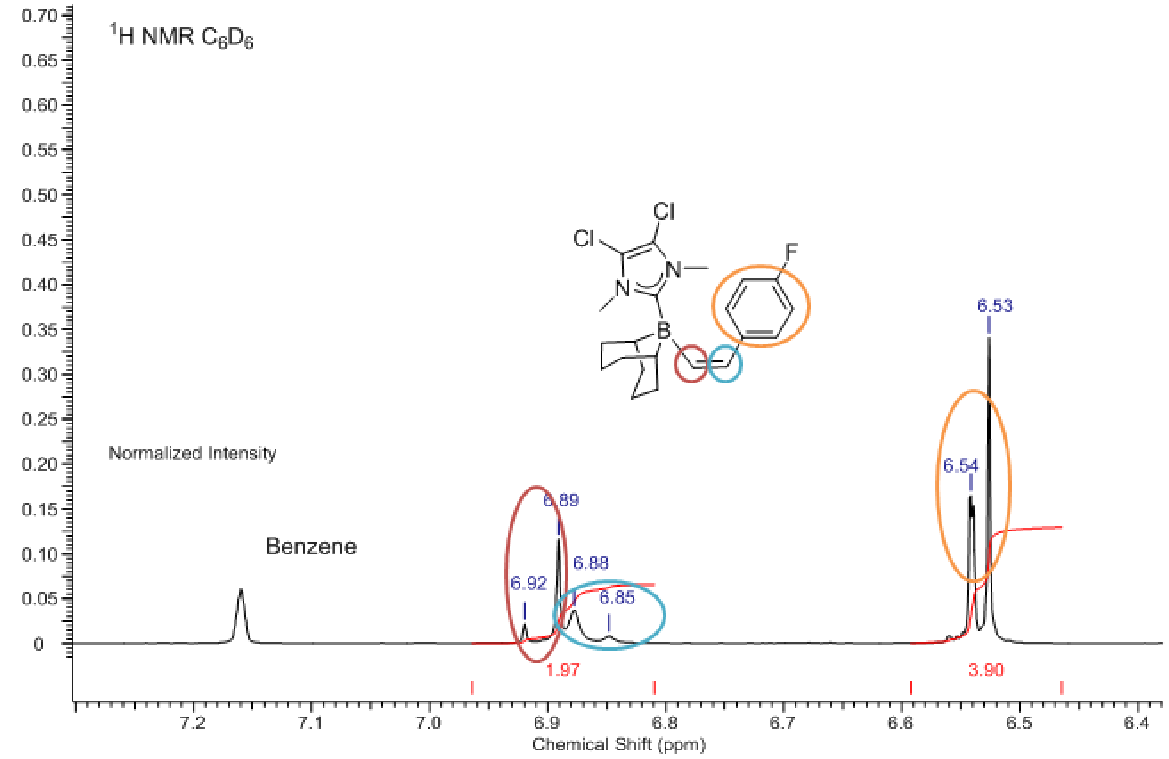

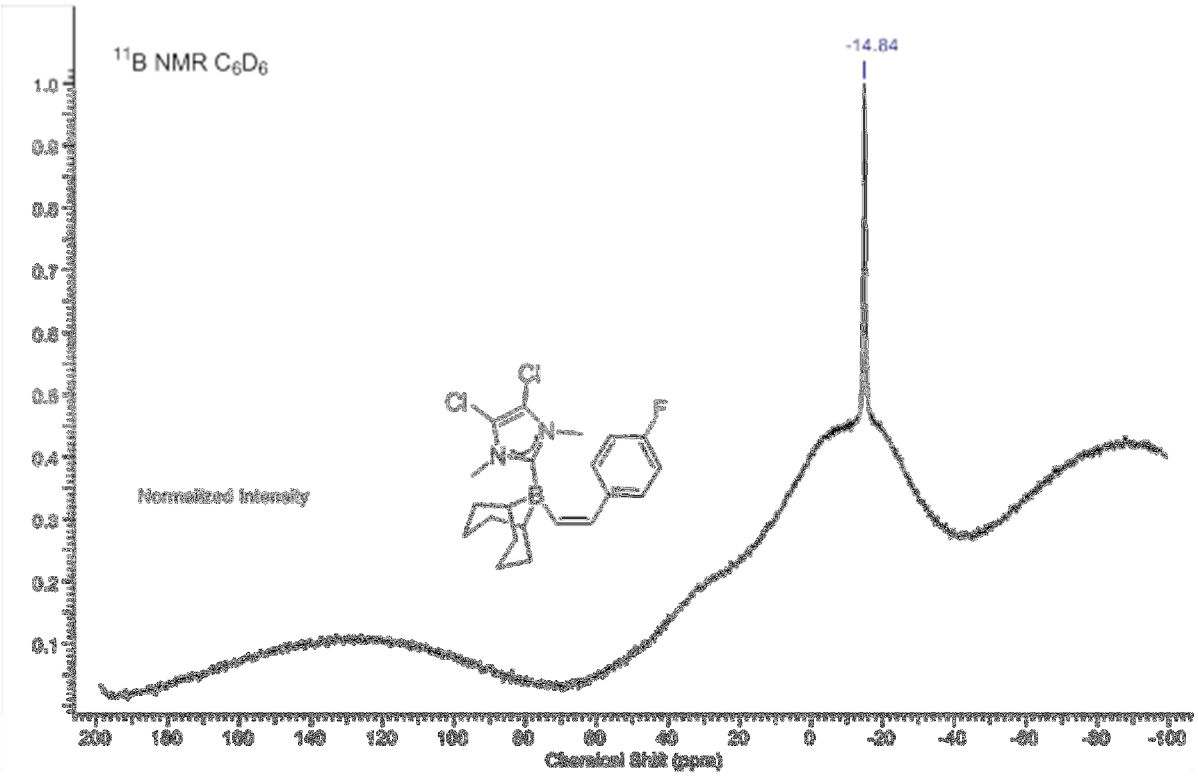

11B NMR C6D6
-14.84
Normalized Intensity
Chemical Shift (ppm)

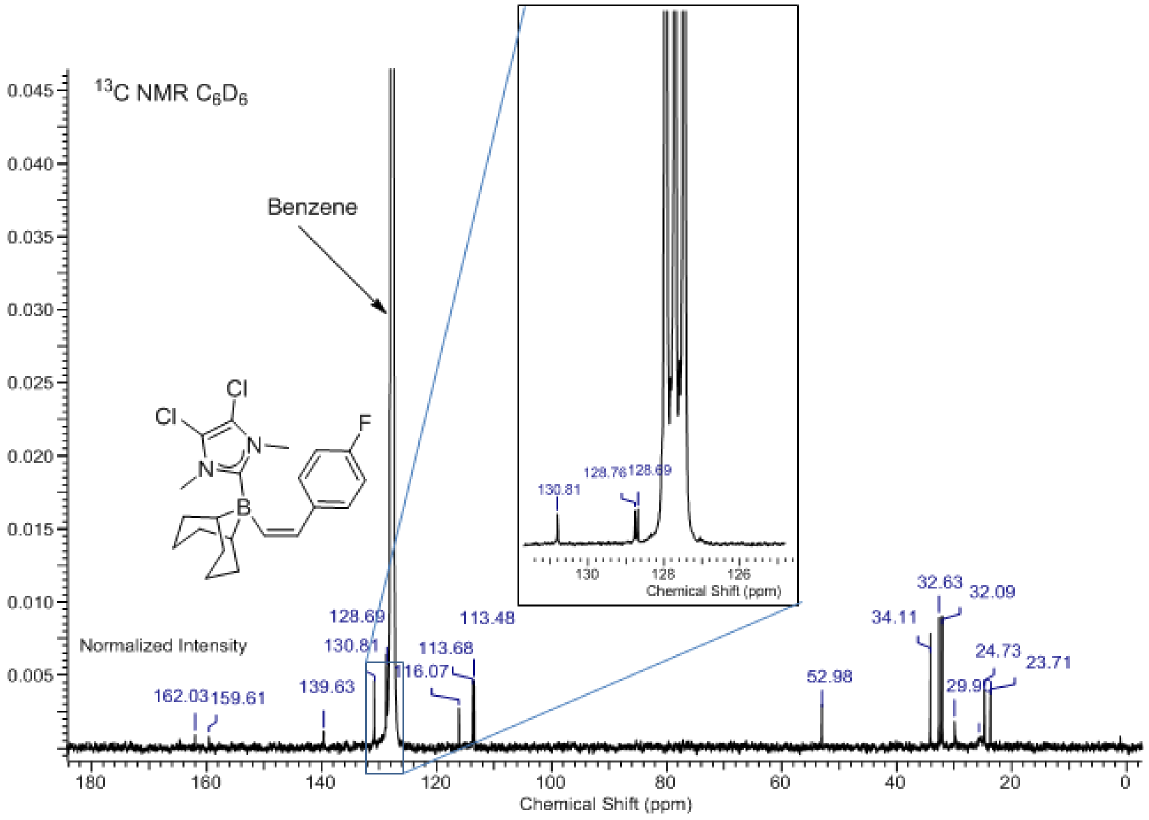

13C NMR C6D6
Benzene
Normalized Intensity
162.03
159.61
139.63
130.81
128.69
116.07
113.68
113.48
52.98
34.11
32.63
32.09
29.91
24.73
23.71
128.76
Chemical Shift (ppm)

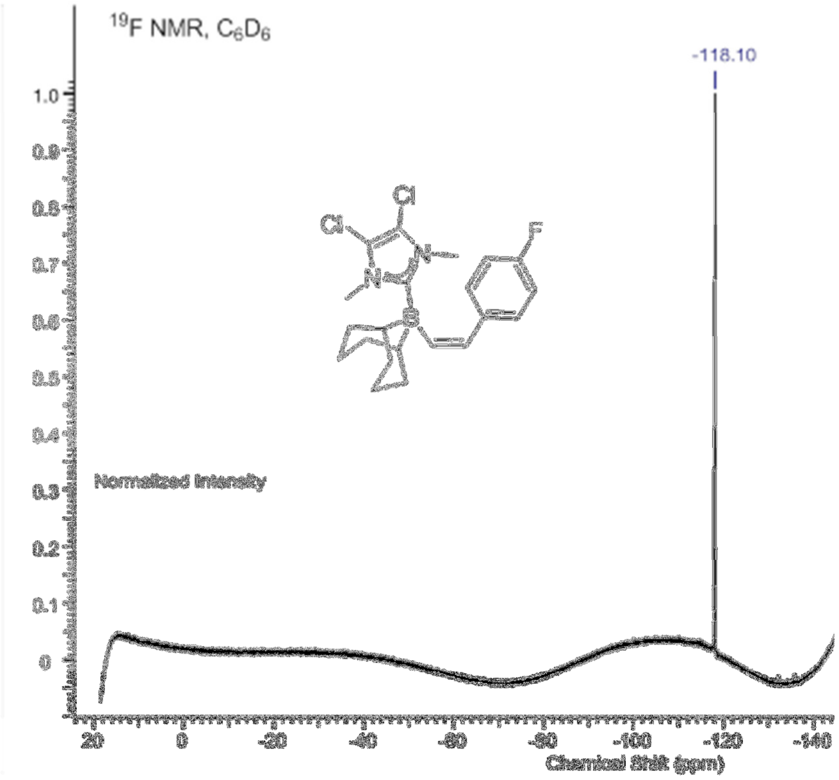

^{19}F NMR, C_6D_6
-118.10
Normalized Intensity
Chemical Shift (ppm)

9-BBN(I-DCDM)(2-(*meta*-chlorophenyl)-*Z*-ethene) (**10h**)

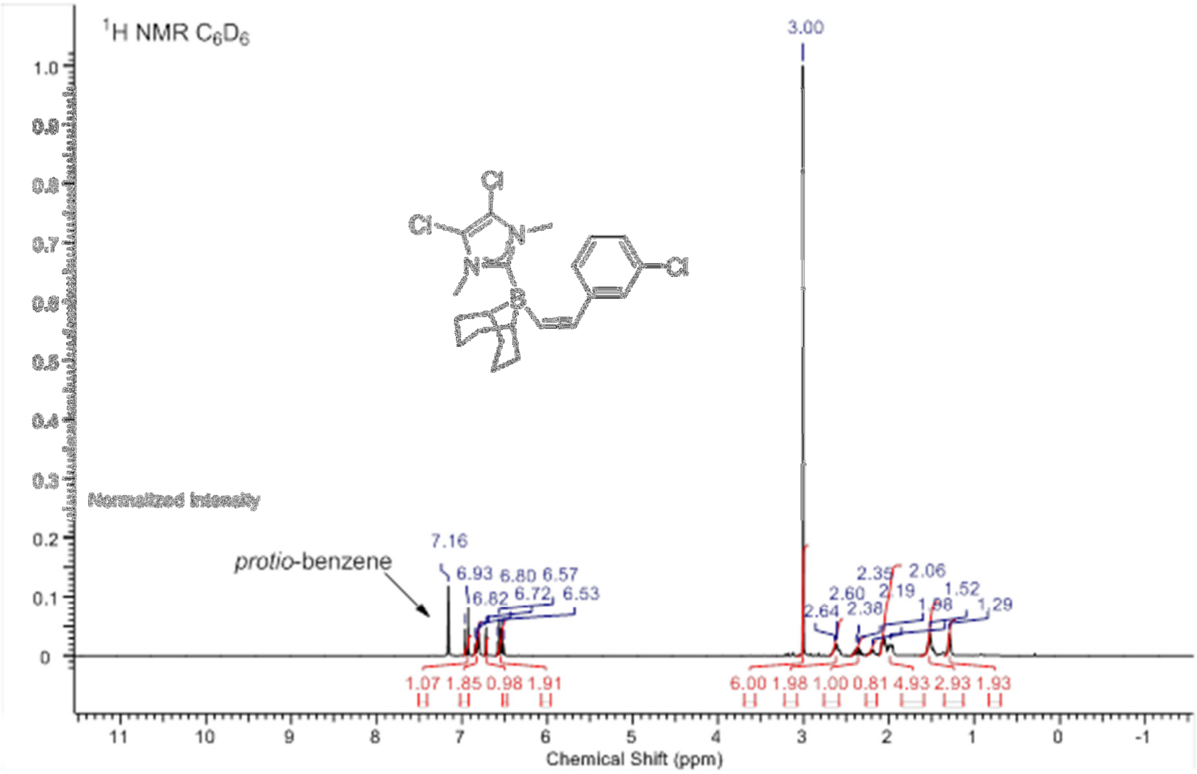

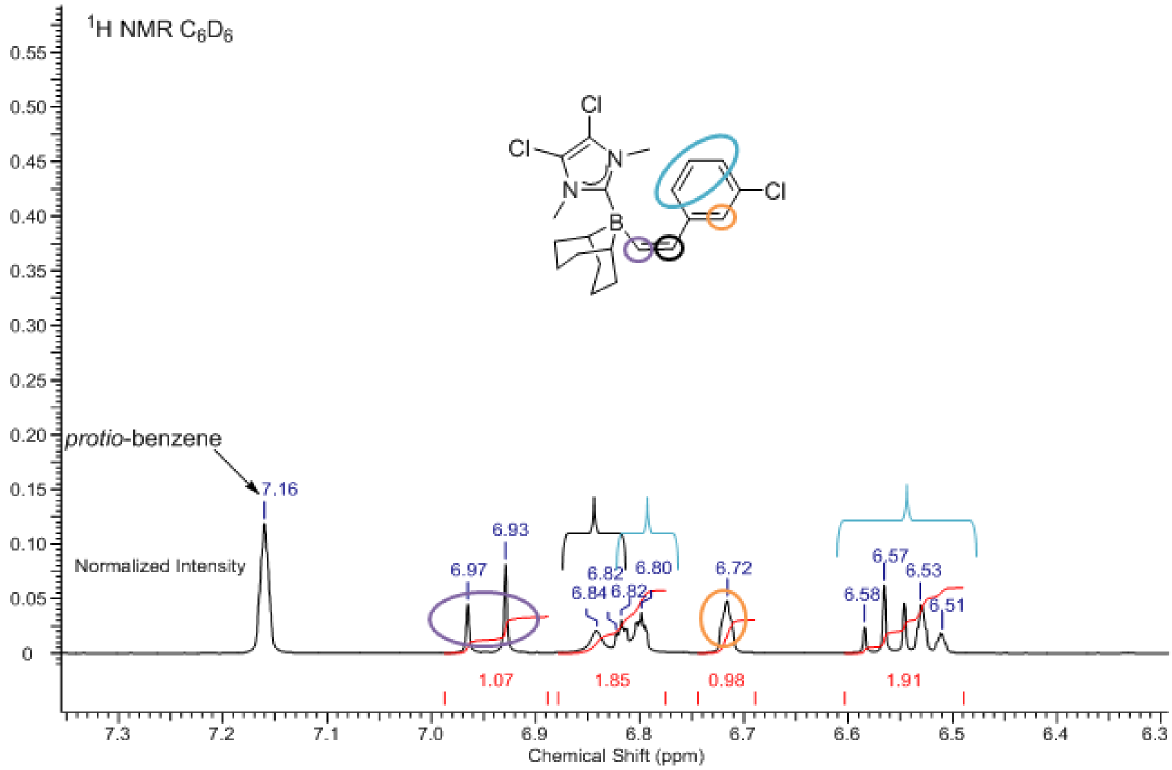

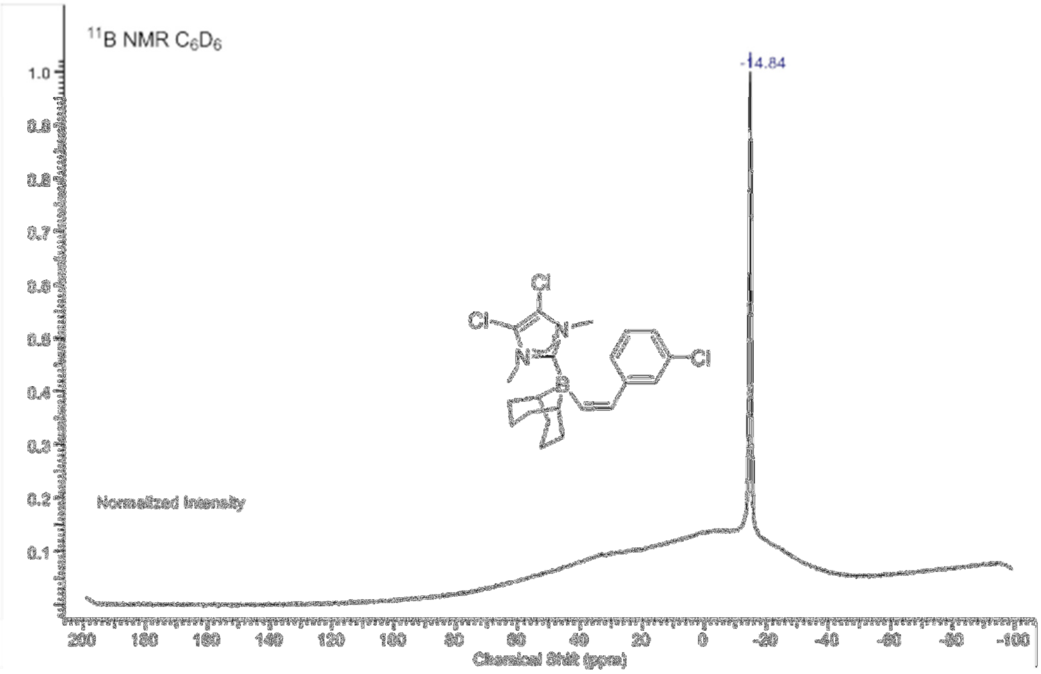
11B NMR C6D6
-14.84
Normalized Intensity
Chemical Shift (ppm)

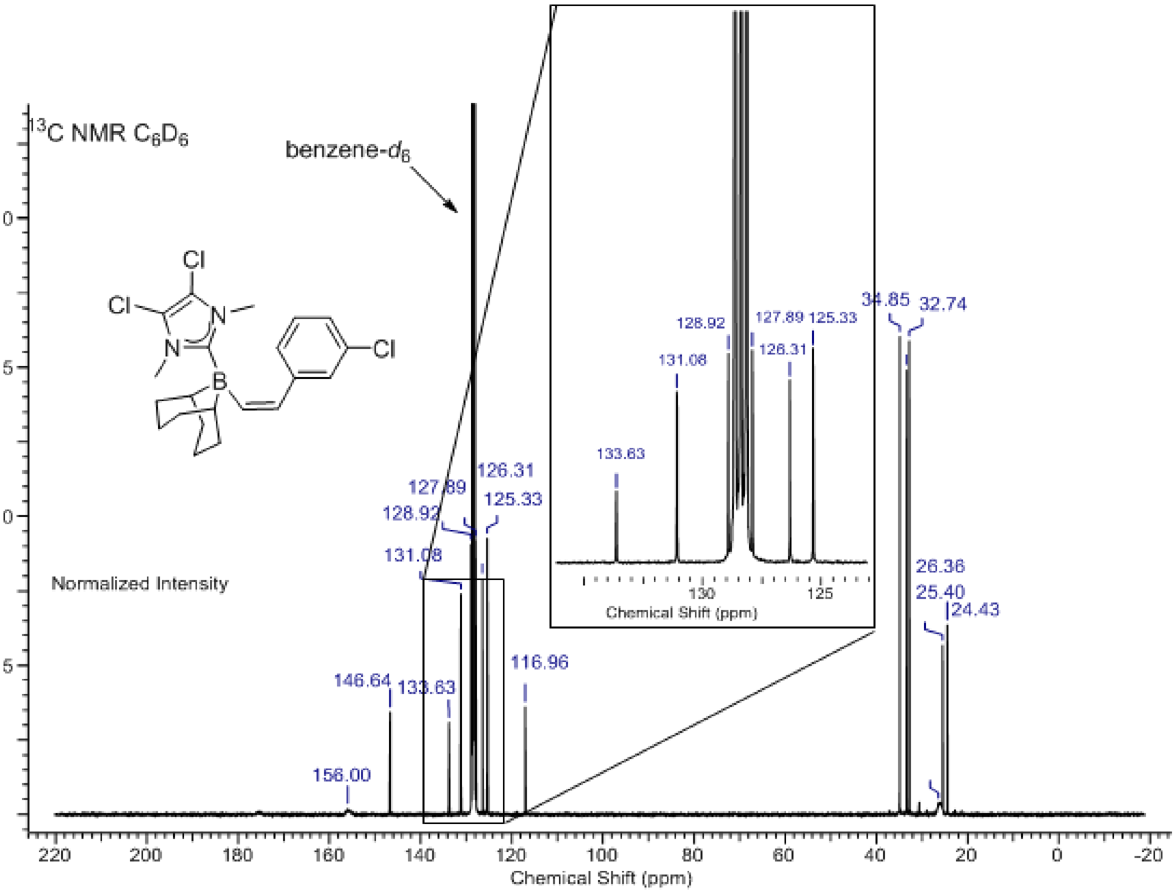
13C NMR C6D6
benzene-d6
Normalized Intensity
156.00
146.64
133.63
131.08
128.92
127.89
126.31
125.33
116.96
34.85
32.74
26.36
25.40
24.43
Chemical Shift (ppm)

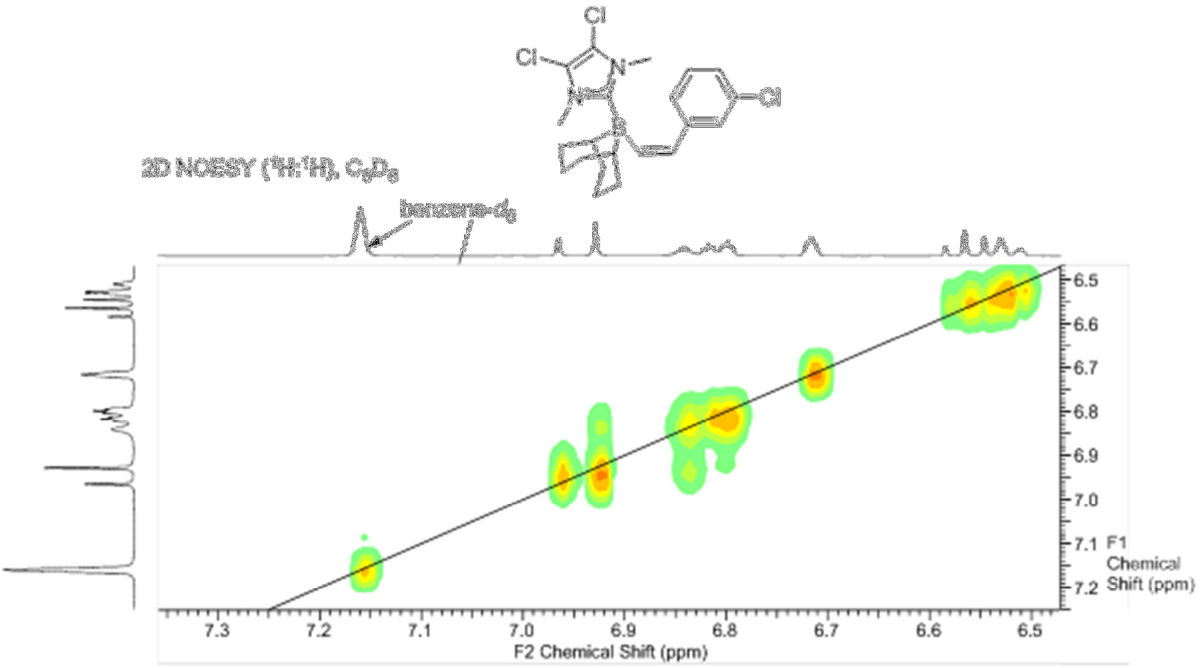
2D NOESY (1H:1H), C6D6
benzene-d6
F2 Chemical Shift (ppm)
F1 Chemical Shift (ppm)

9-BBN(I-DCDM)(2-(2,4,6-trimethylphenyl)-*Z*-ethene) (**10i**)

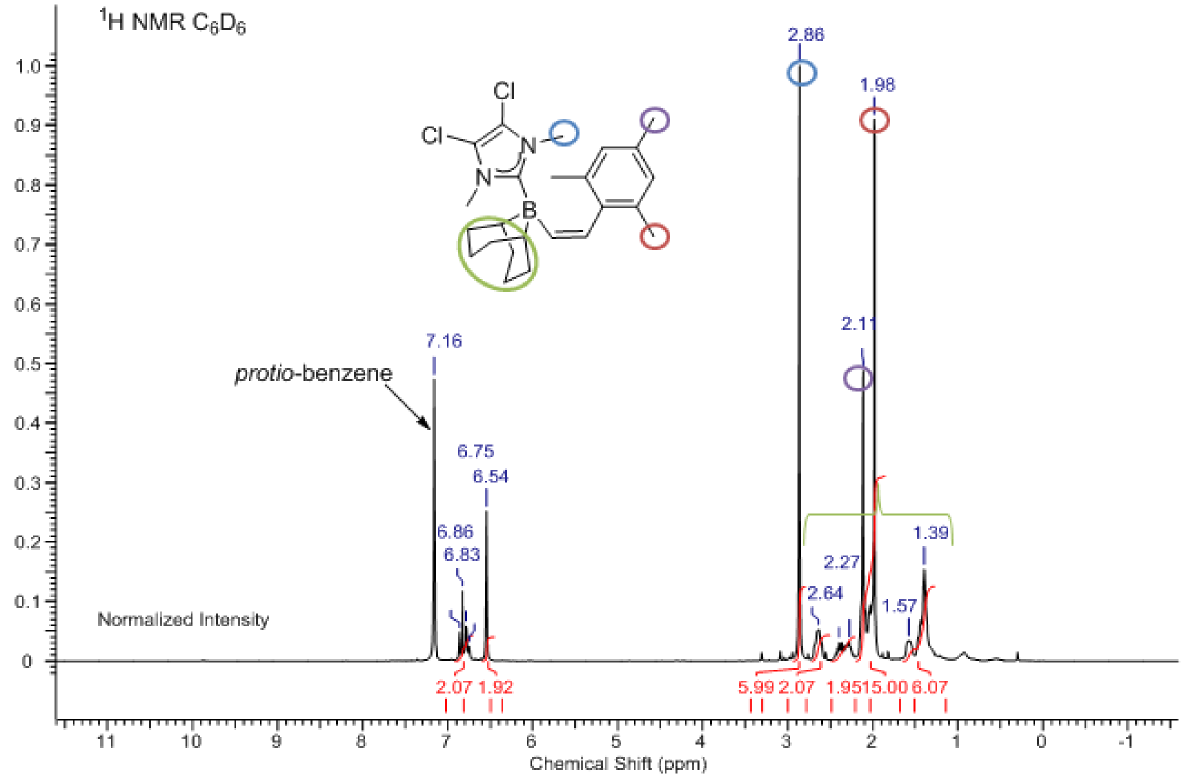

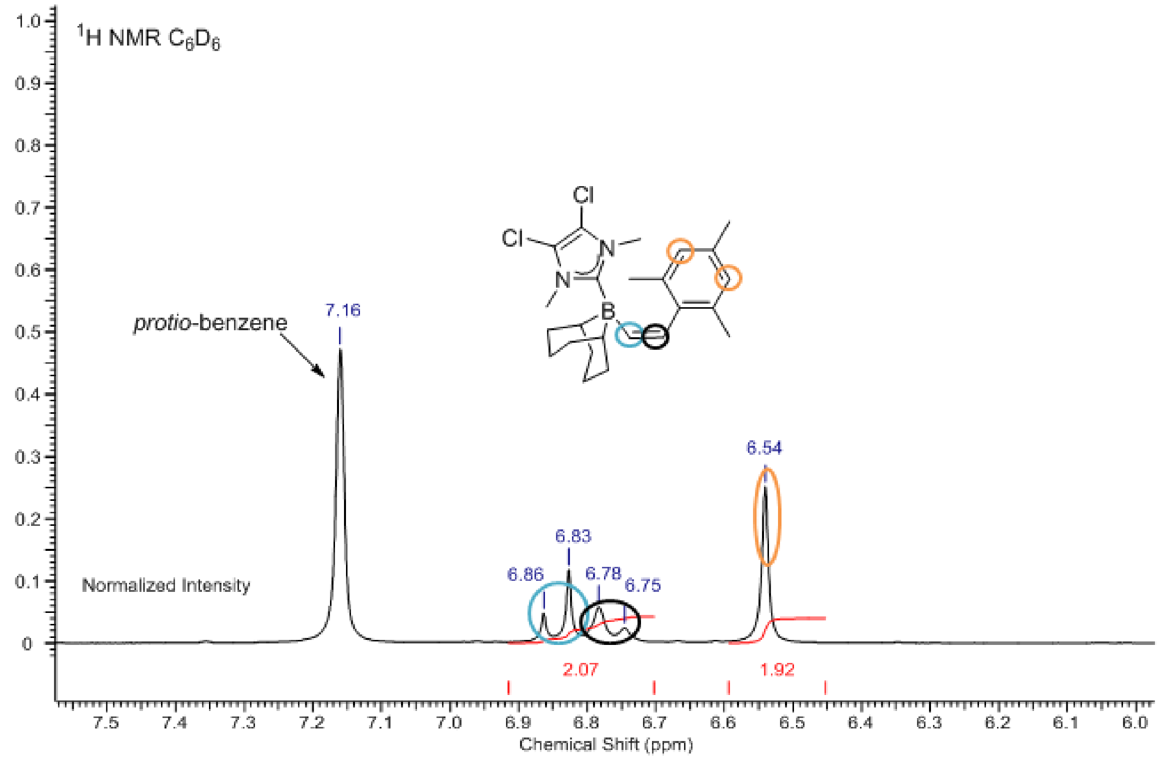

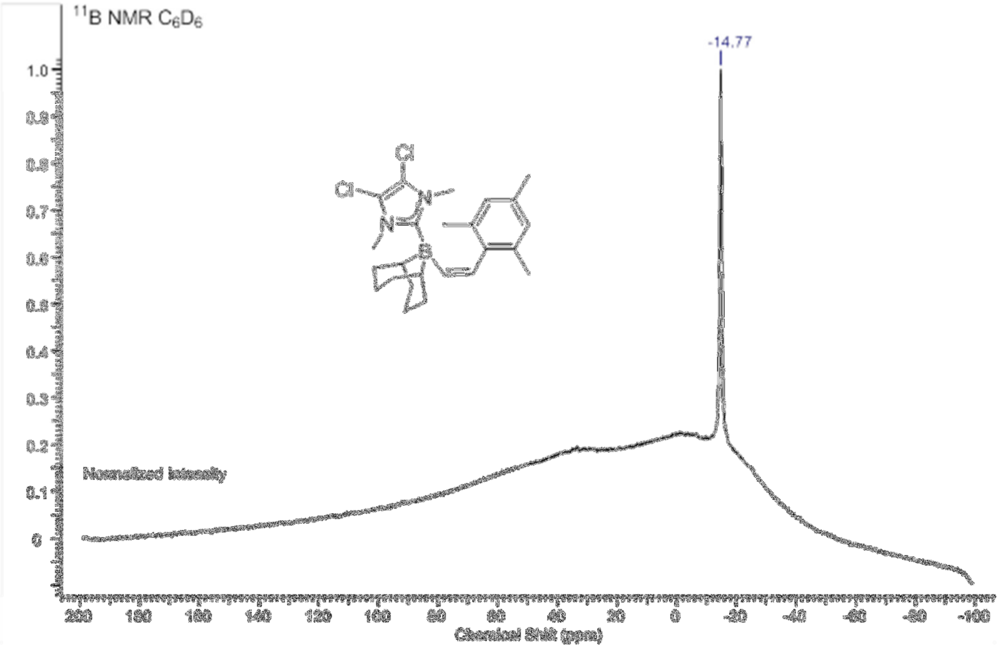

11B NMR C6D6
-14.77
Normalized Intensity
Chemical Shift (ppm)

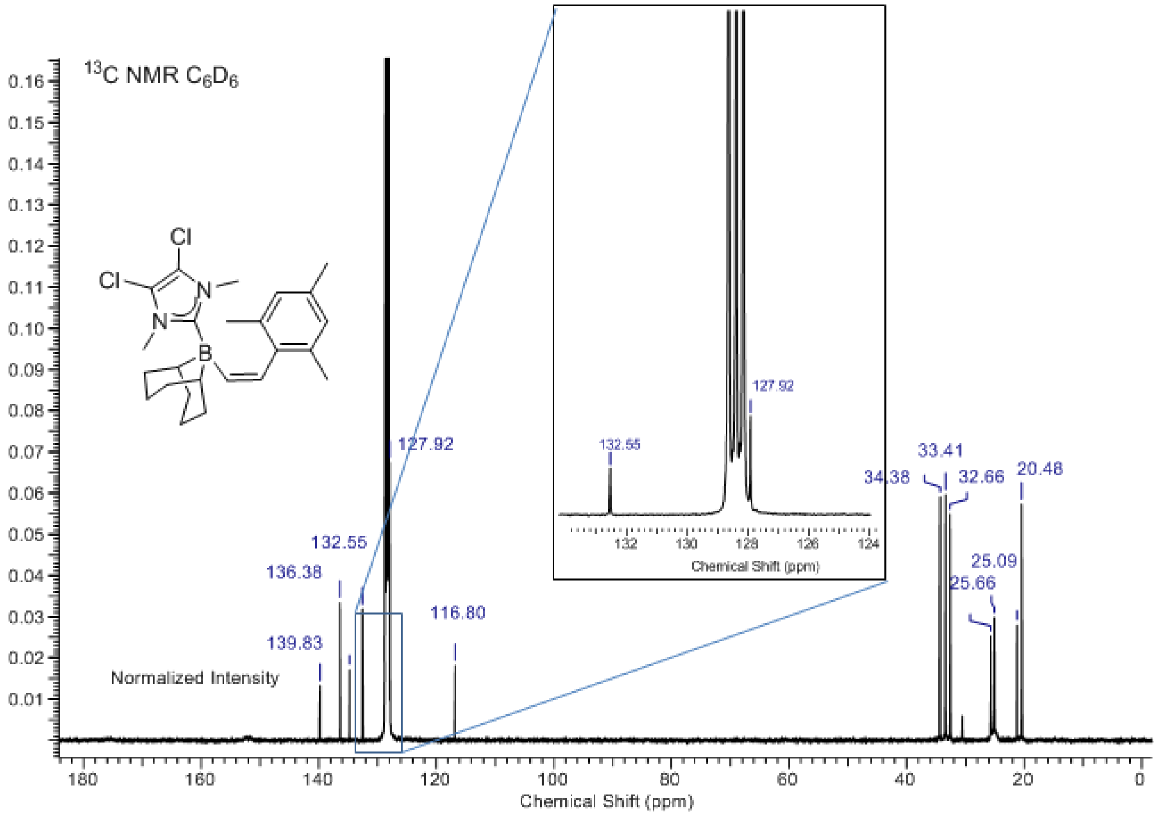

13C NMR C6D6
127.92
132.55
136.38
139.83
116.80
33.41
34.38
32.66
20.48
25.09
25.66
Normalized Intensity
Chemical Shift (ppm)

9-BBN(I-DCDM)(2-(2-thiophenyl)-*Z*-ethene) (**10j**)

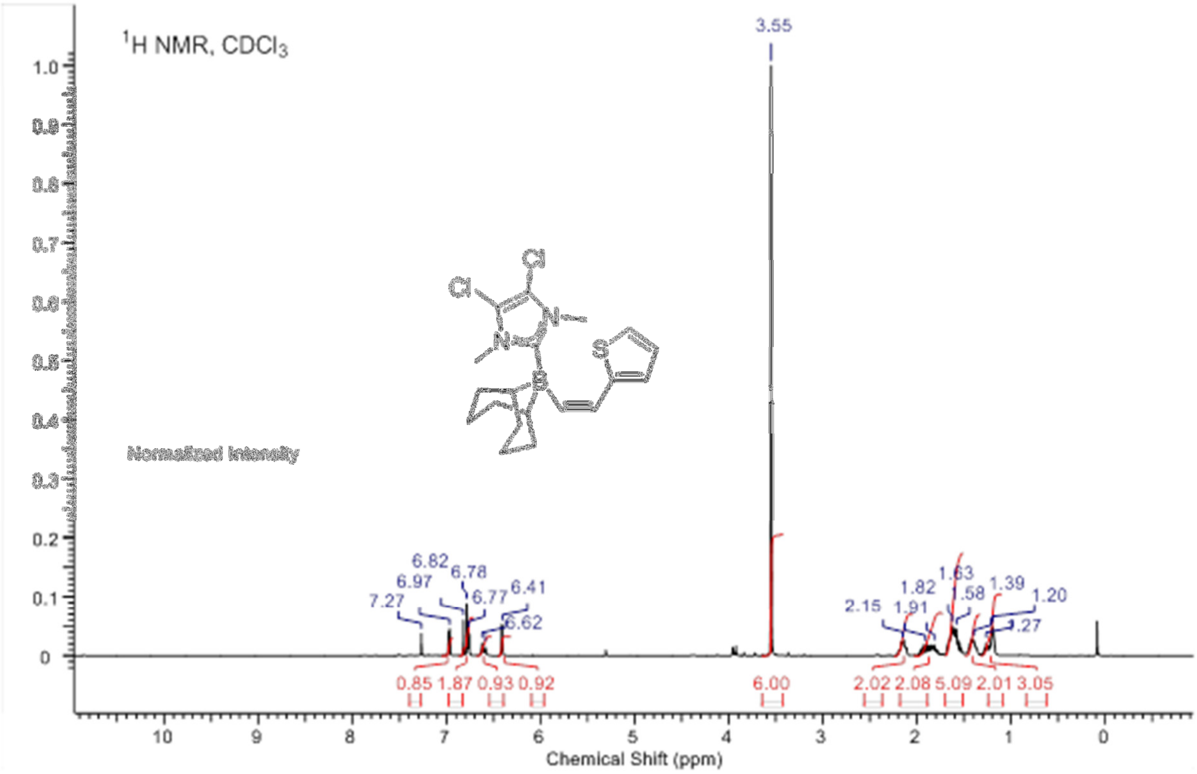

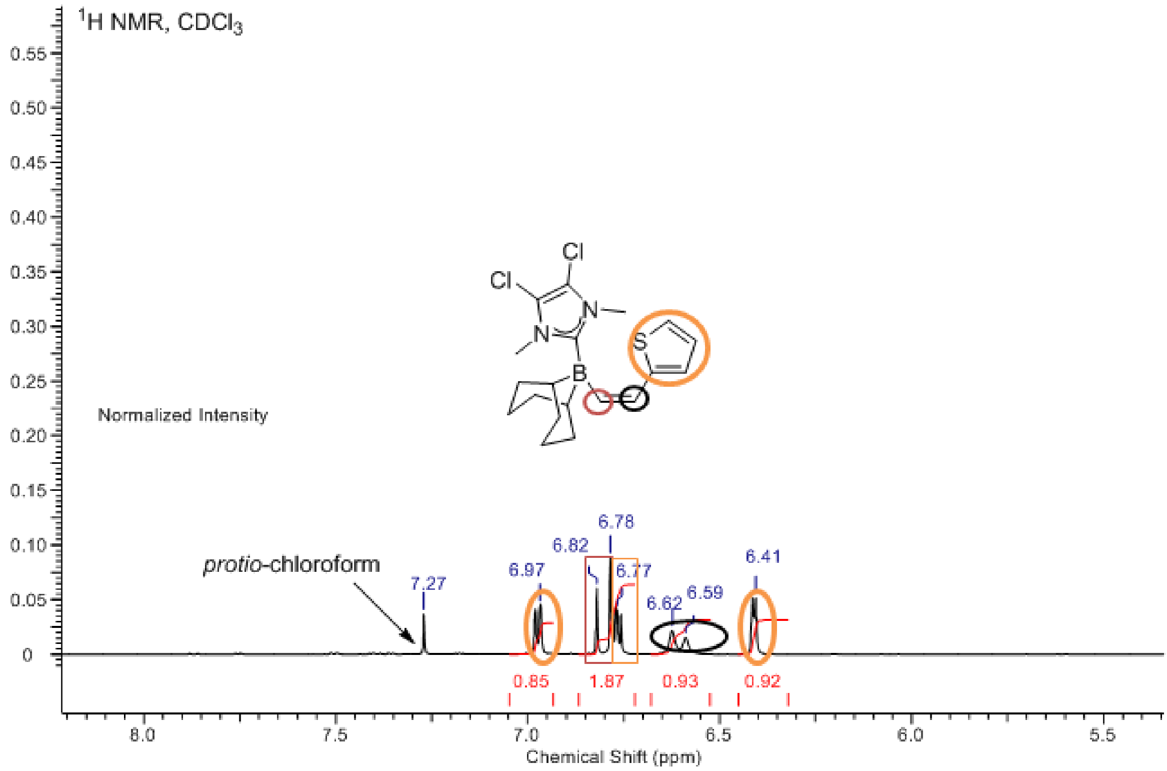

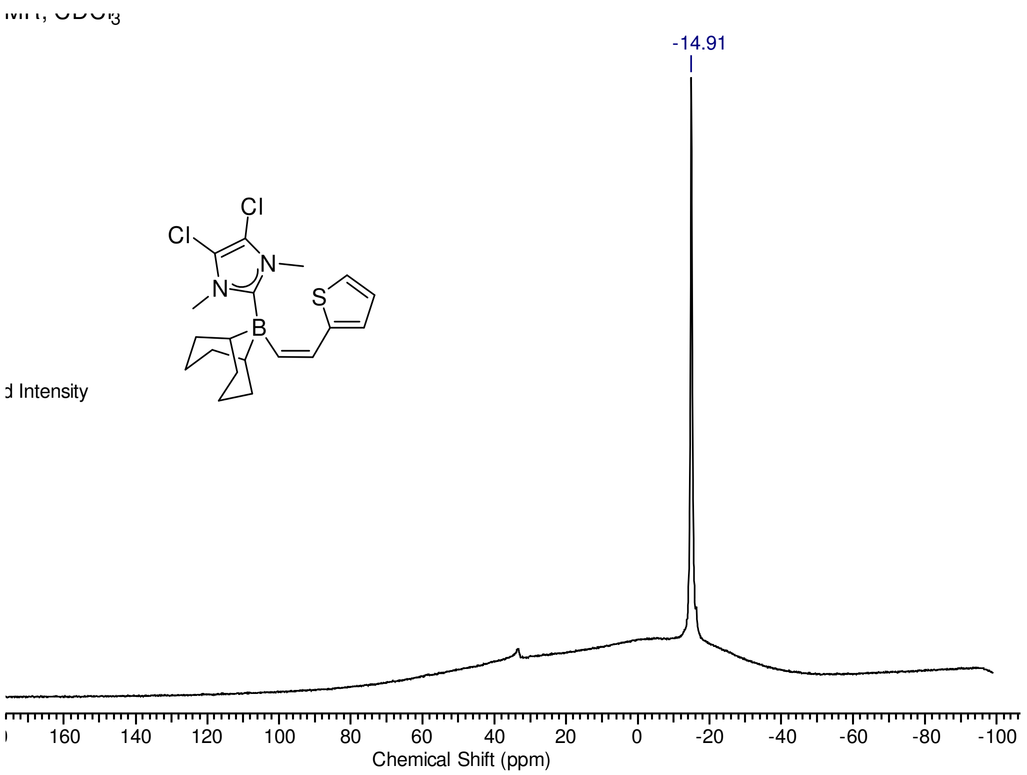

-14.91
Cl
Cl
N
N
S
B
d Intensity
160
140
120
100
80
60
40
20
0
-20
-40
-60
-80
-100
Chemical Shift (ppm)

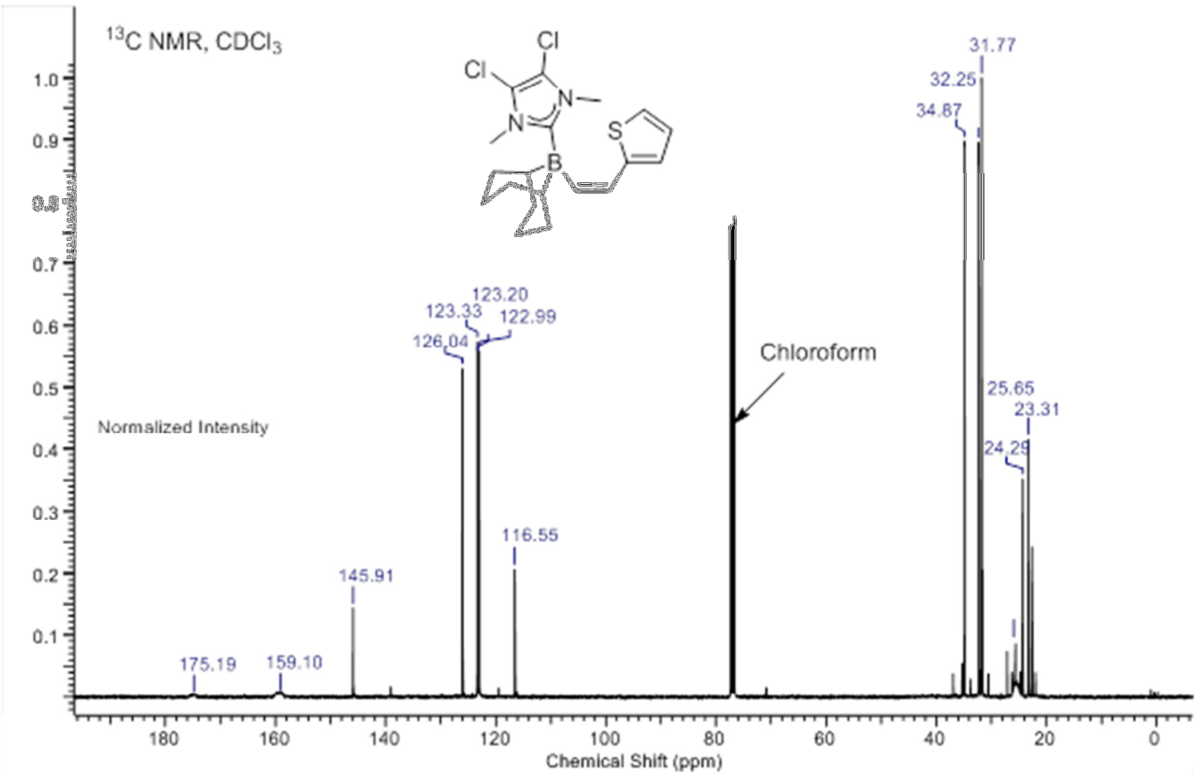

13C NMR, CDCl3
Cl
Cl
N
N
S
B
Normalized Intensity
Chloroform
175.19
159.10
145.91
126.04
123.33
123.20
122.99
116.55
34.87
32.25
31.77
25.65
24.29
23.31
Chemical Shift (ppm)

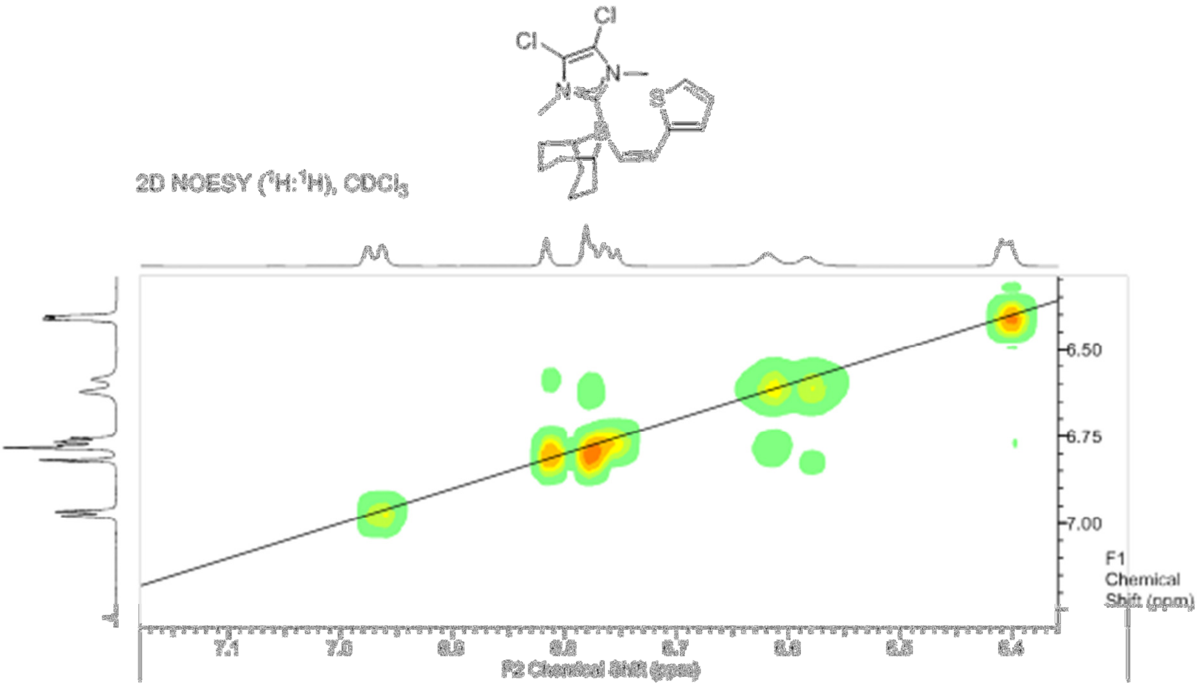

Cl
Cl
N
N
S
B
2D NOESY (¹H:¹H), CDCl₃
6.50
6.75
7.00
F1 Chemical Shift (ppm)
7.1
7.0
6.9
6.8
6.7
6.6
6.5
6.4
F2 Chemical Shift (ppm)

9-BBN(I-DCDM)(Z-ethynyl-cyclohex-1-ene) (**10k**)

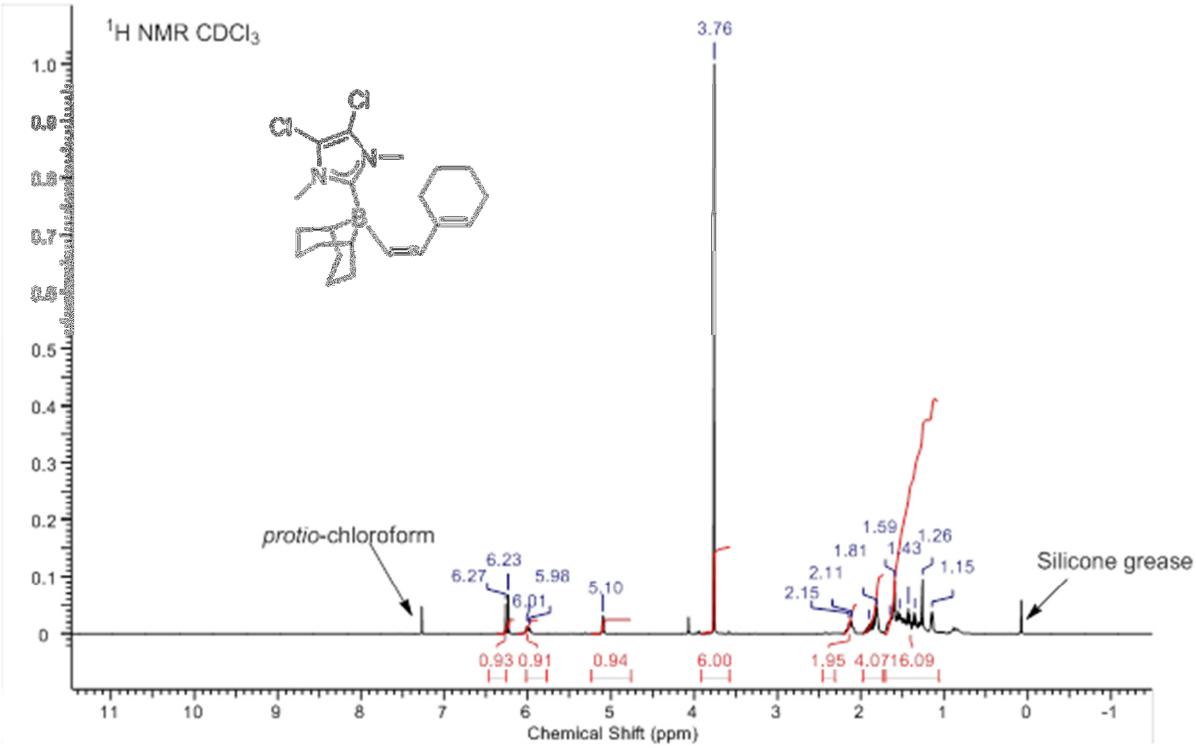

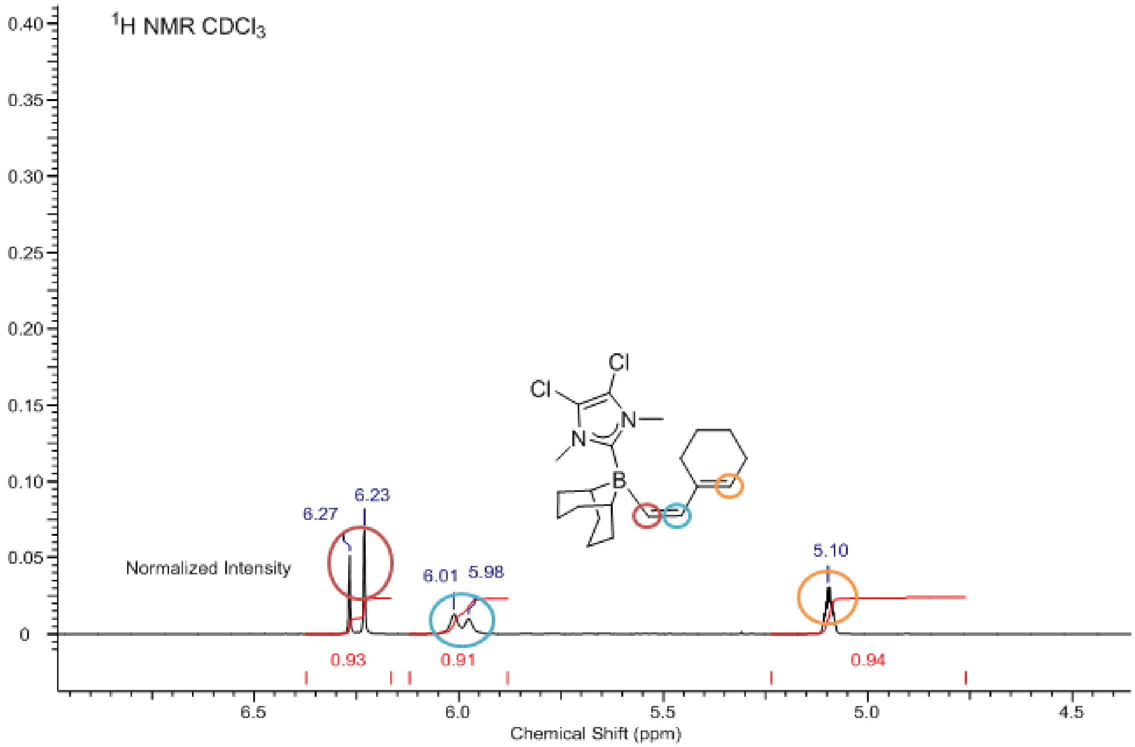

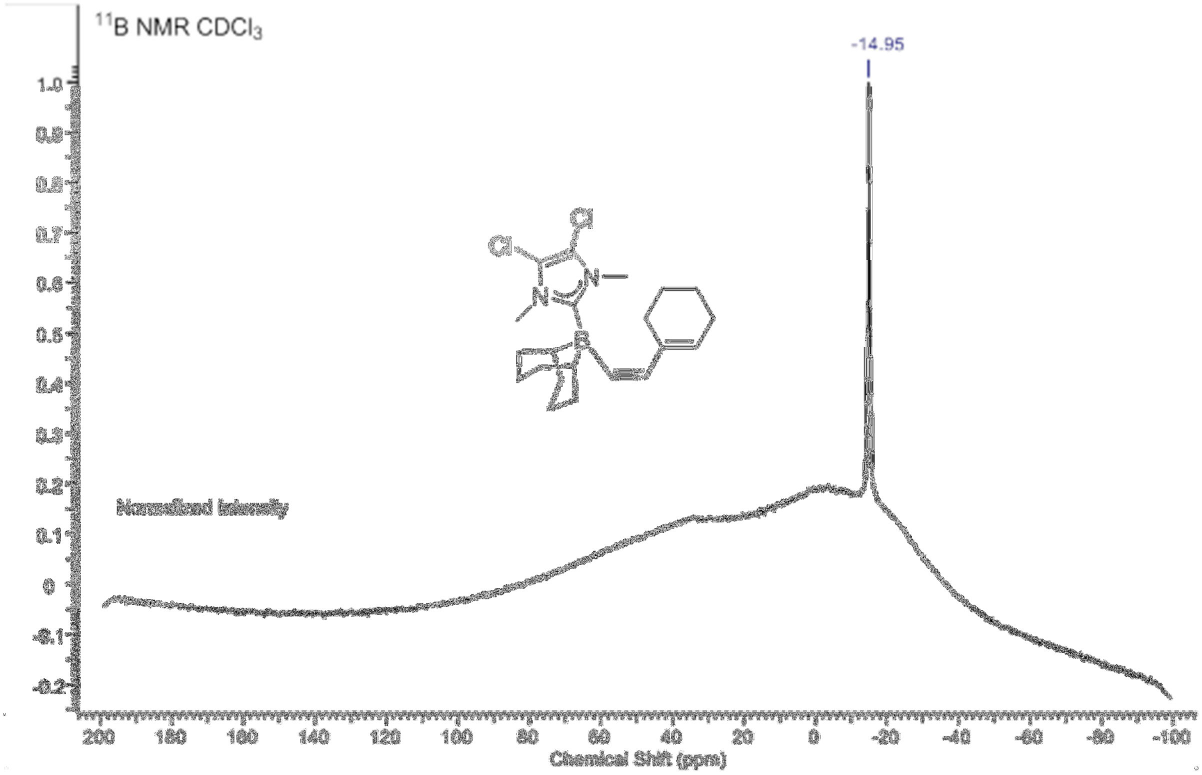

11B NMR CDCl3
-14.95
Cl
Cl
N
N
B
Normalized Intensity
Chemical Shift (ppm)

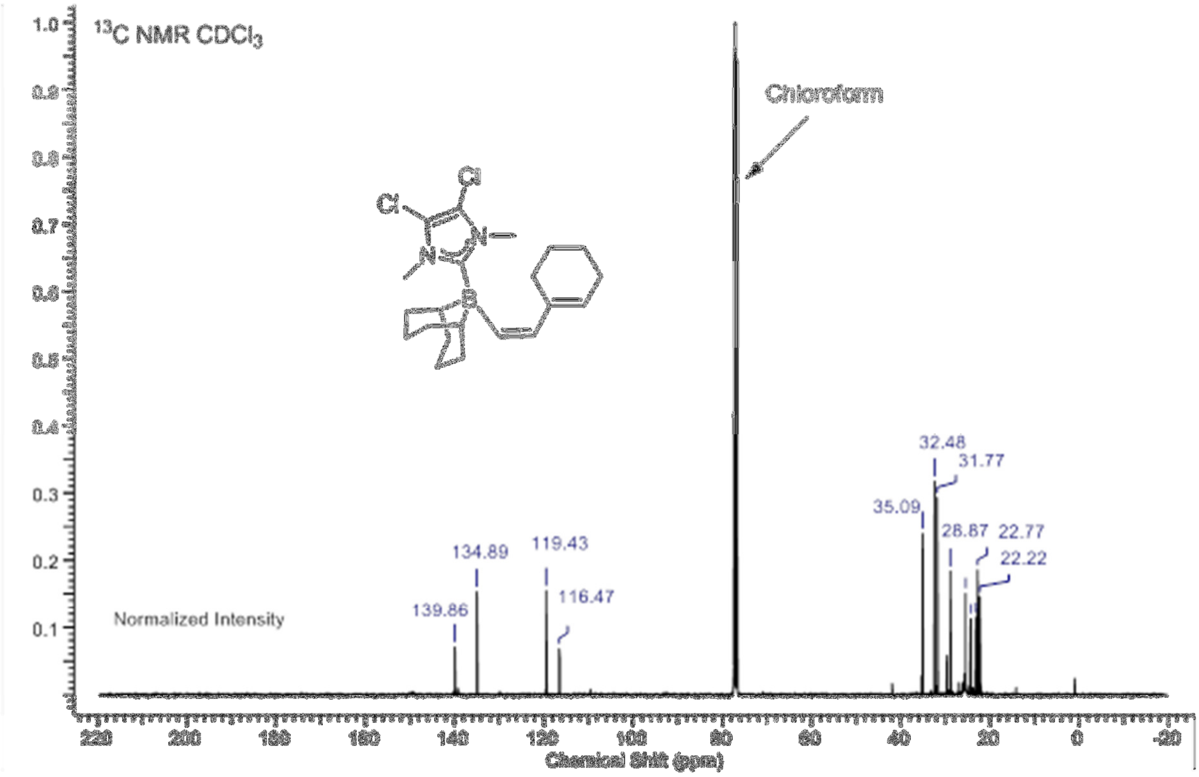

13C NMR CDCl3
Chloroform
Cl
Cl
N
N
B
32.48
31.77
35.09
28.87
22.77
22.22
134.89
119.43
116.47
139.86
Normalized Intensity
Chemical Shift (ppm)

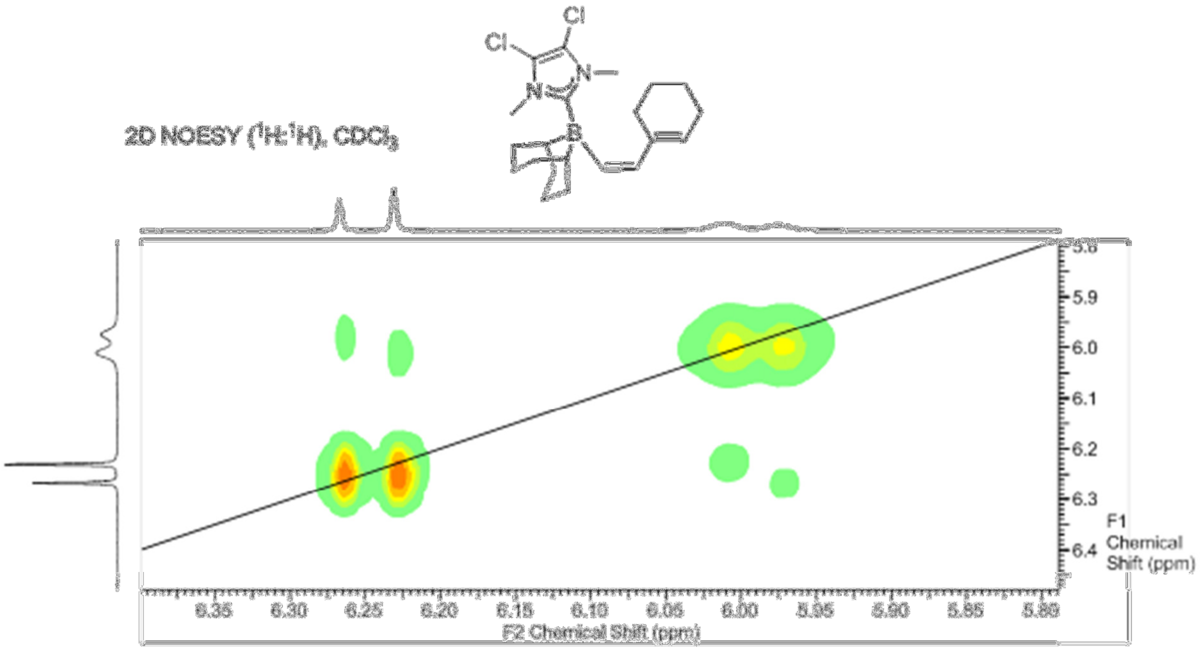

2D NOESY (¹H:¹H), CDCl₃
Cl
Cl
N
N
B
F1 Chemical Shift (ppm)
F2 Chemical Shift (ppm)
6.35
6.30
6.25
6.20
6.15
6.10
6.05
6.00
5.95
5.90
5.85
5.80
5.9
6.0
6.1
6.2
6.3
6.4

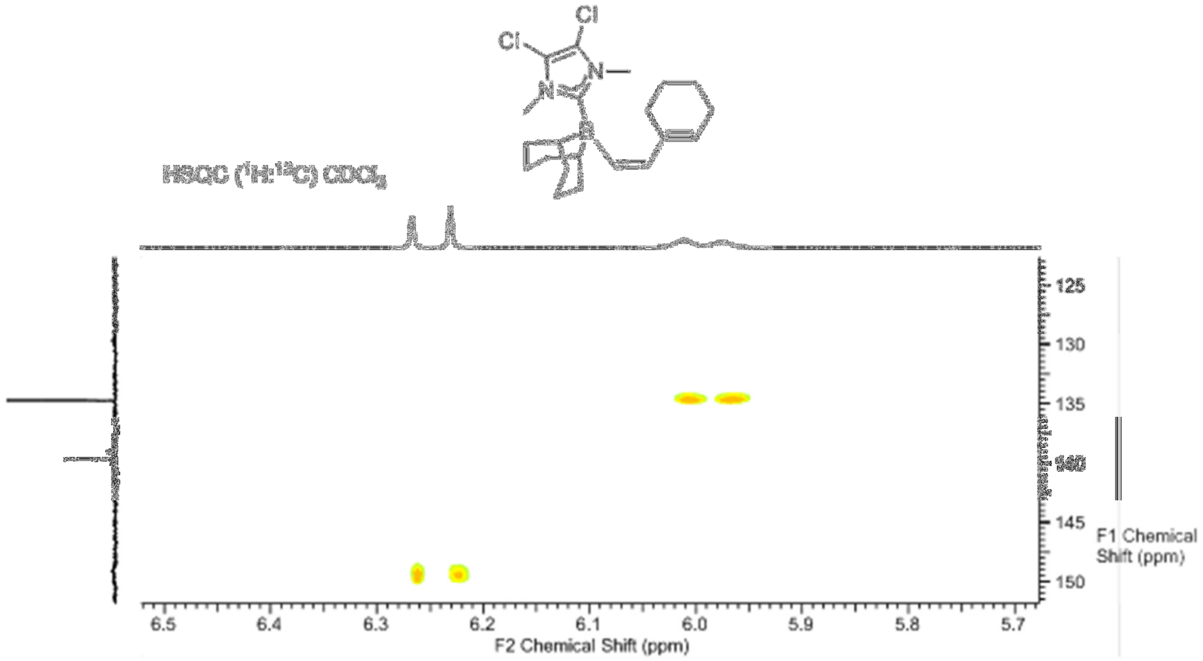

HSQC (¹H:¹³C) CDCl₃
Cl
Cl
N
N
B
F1 Chemical Shift (ppm)
F2 Chemical Shift (ppm)
6.5
6.4
6.3
6.2
6.1
6.0
5.9
5.8
5.7
125
130
135
145
150

In-situ NMR spectra (with mesitylene as internal standard) for 1-[9-BBN(I-DCDM)]-(*Z)*-4-methylbuta-1,3-diene (**10l**)

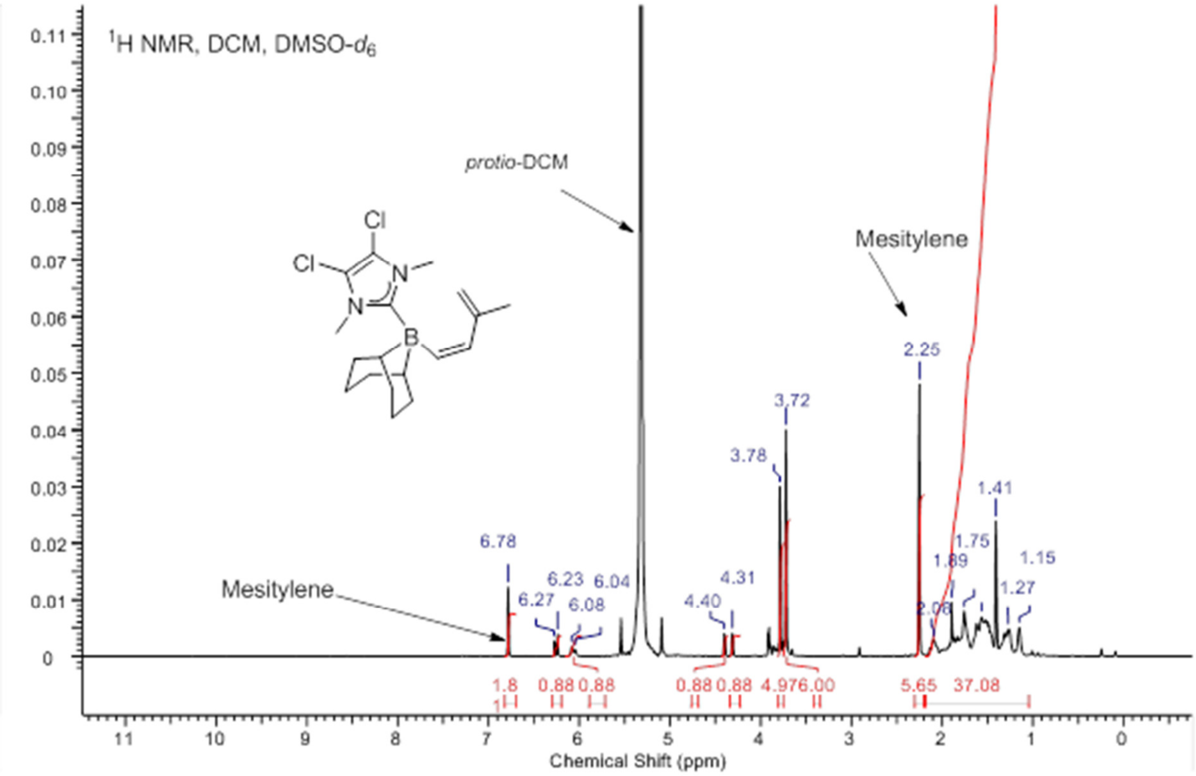

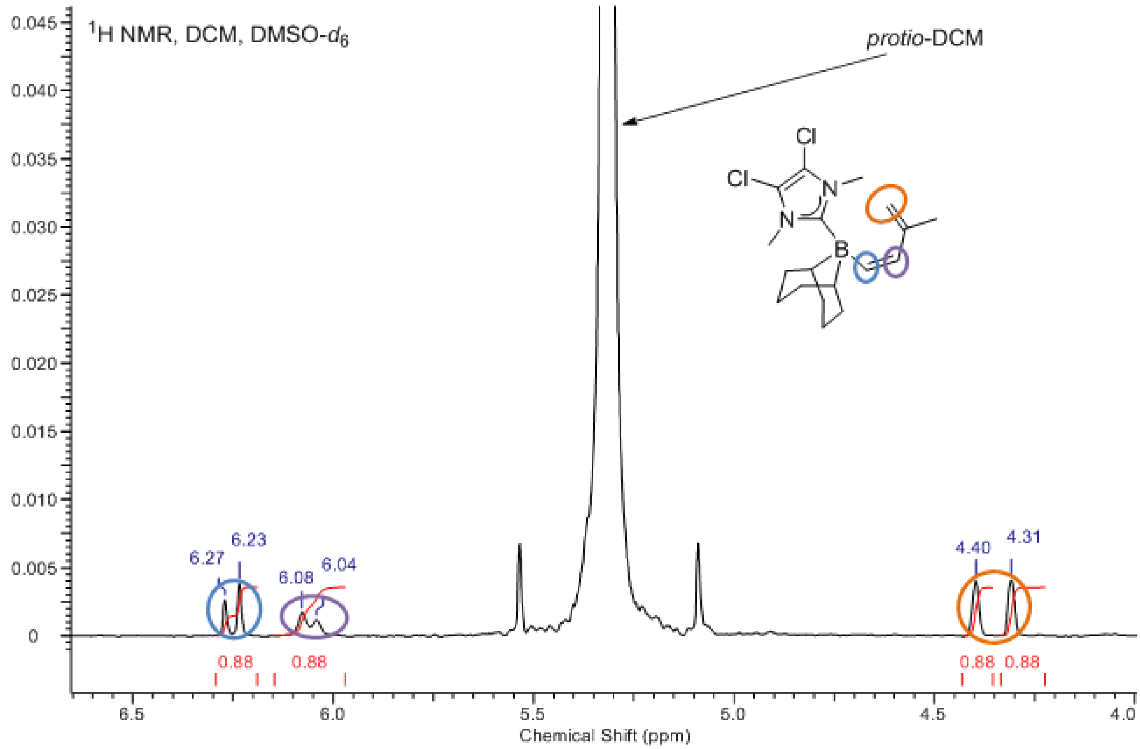

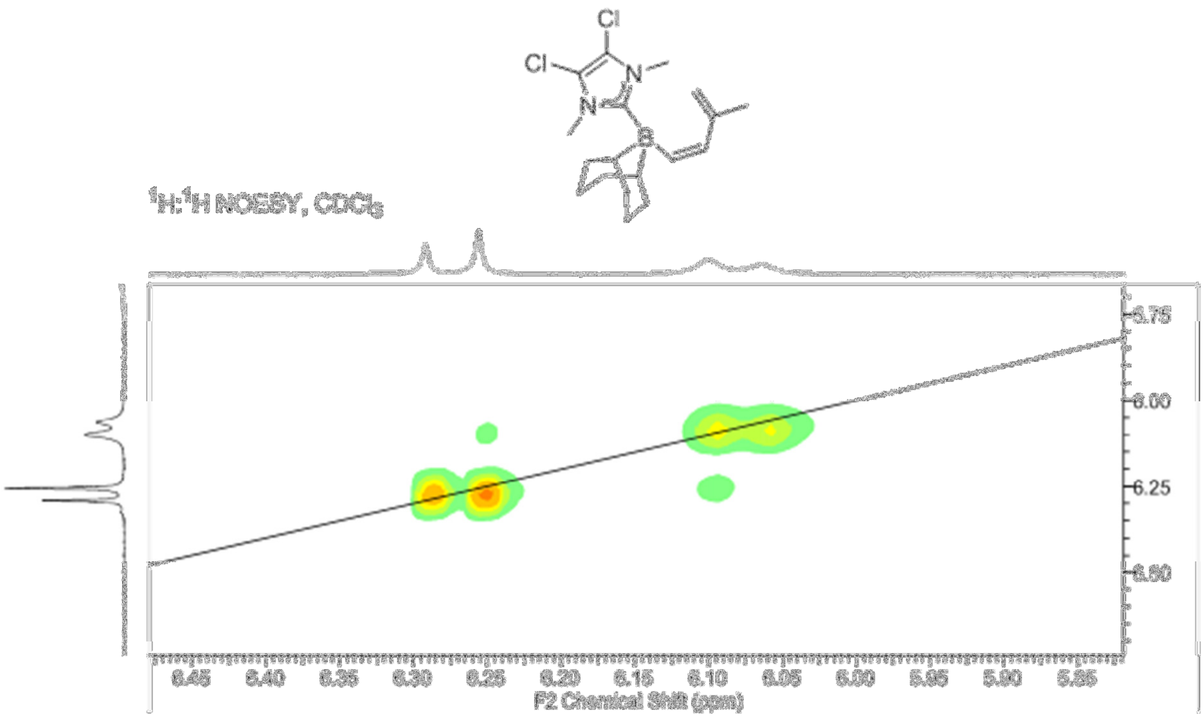

Cl
Cl
N
N
B
1H:1H NOESY, CDCl3
5.75
6.00
6.25
6.50
6.45
6.40
6.35
6.30
6.25
6.20
6.15
6.10
6.05
6.00
5.95
5.90
5.85
F2 Chemical Shift (ppm)

Crude mixture containing 9-BBN(I-DCDM)(1-(*Z*-pent-1-ene)) as the major product

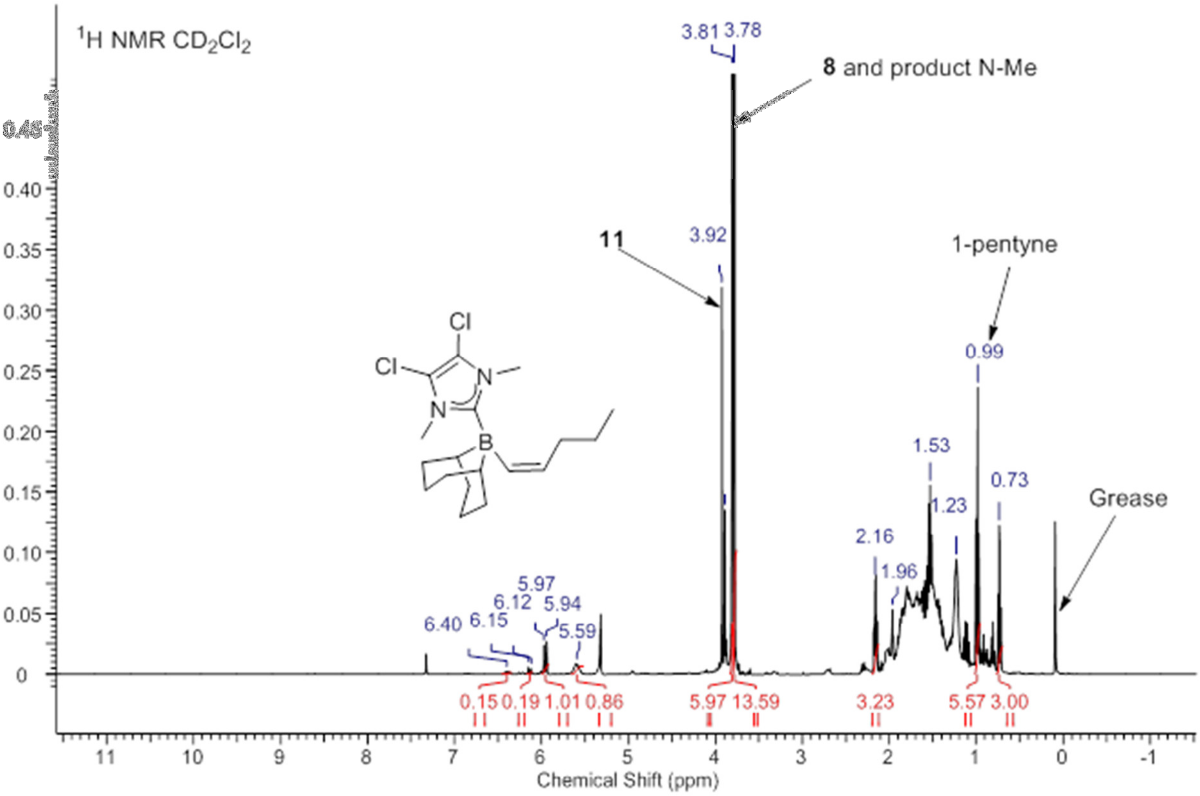

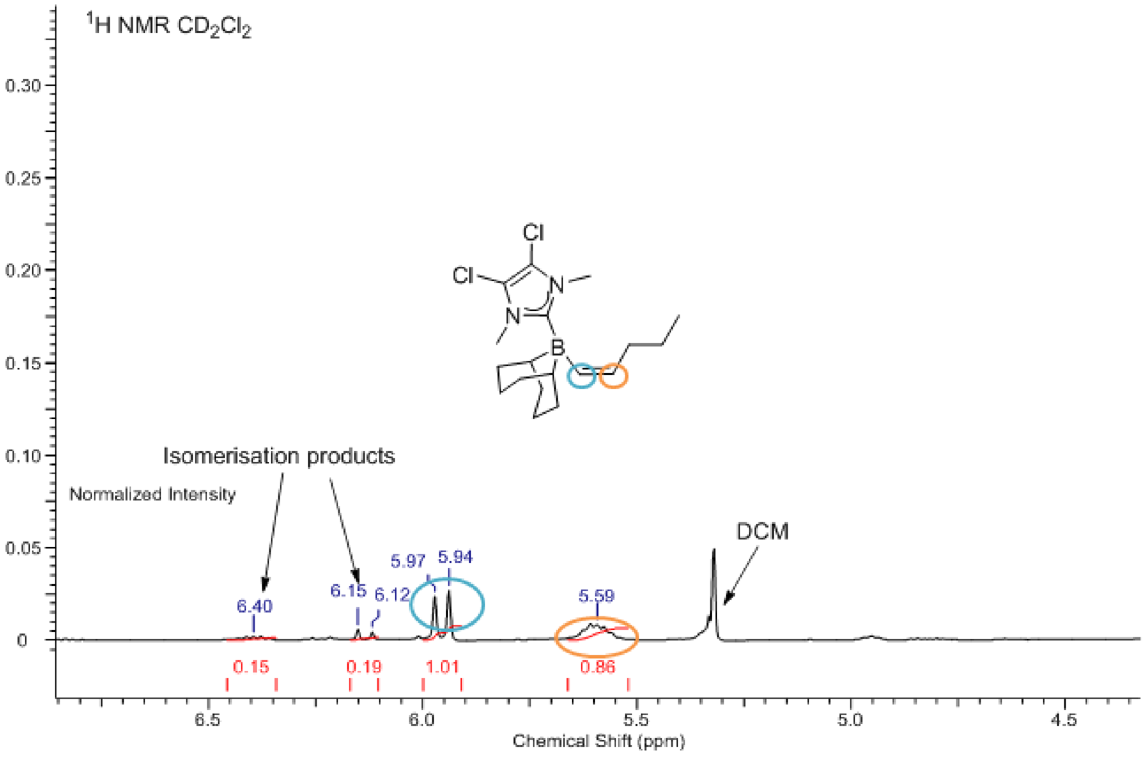

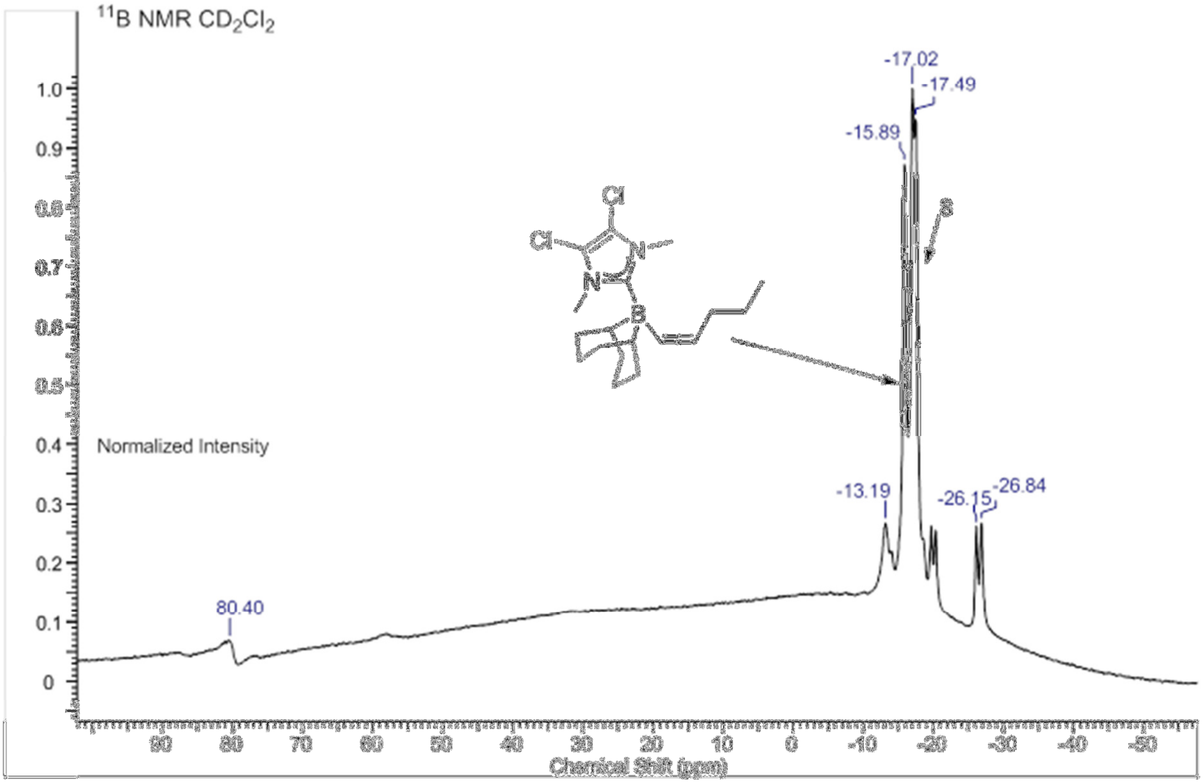

^{11}B NMR CD_2Cl_2
-17.02
-17.49
-15.89
S
Normalized Intensity
-13.19
-26.15
-26.84
80.40
Chemical Shift (ppm)

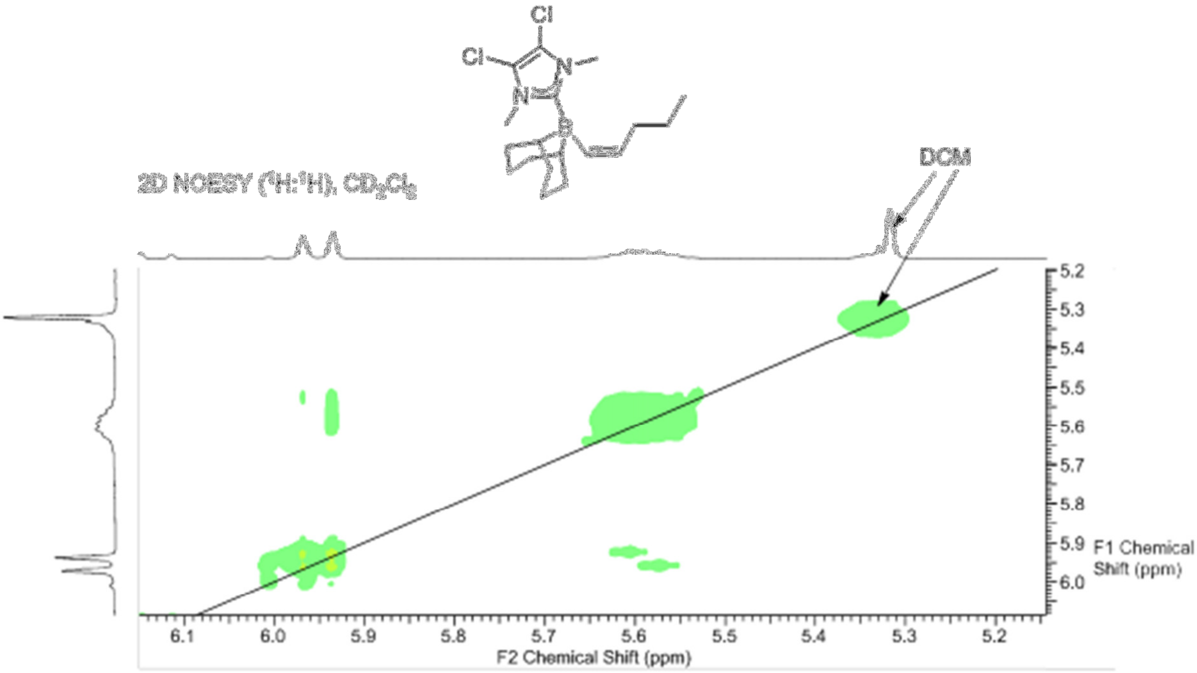

DCM
F1 Chemical Shift (ppm)
F2 Chemical Shift (ppm)

Crude mixture containing 9-BBN(I-DCDM)(1-(*Z*-oct-1-ene)) (**$10m_H$**) as the major new product

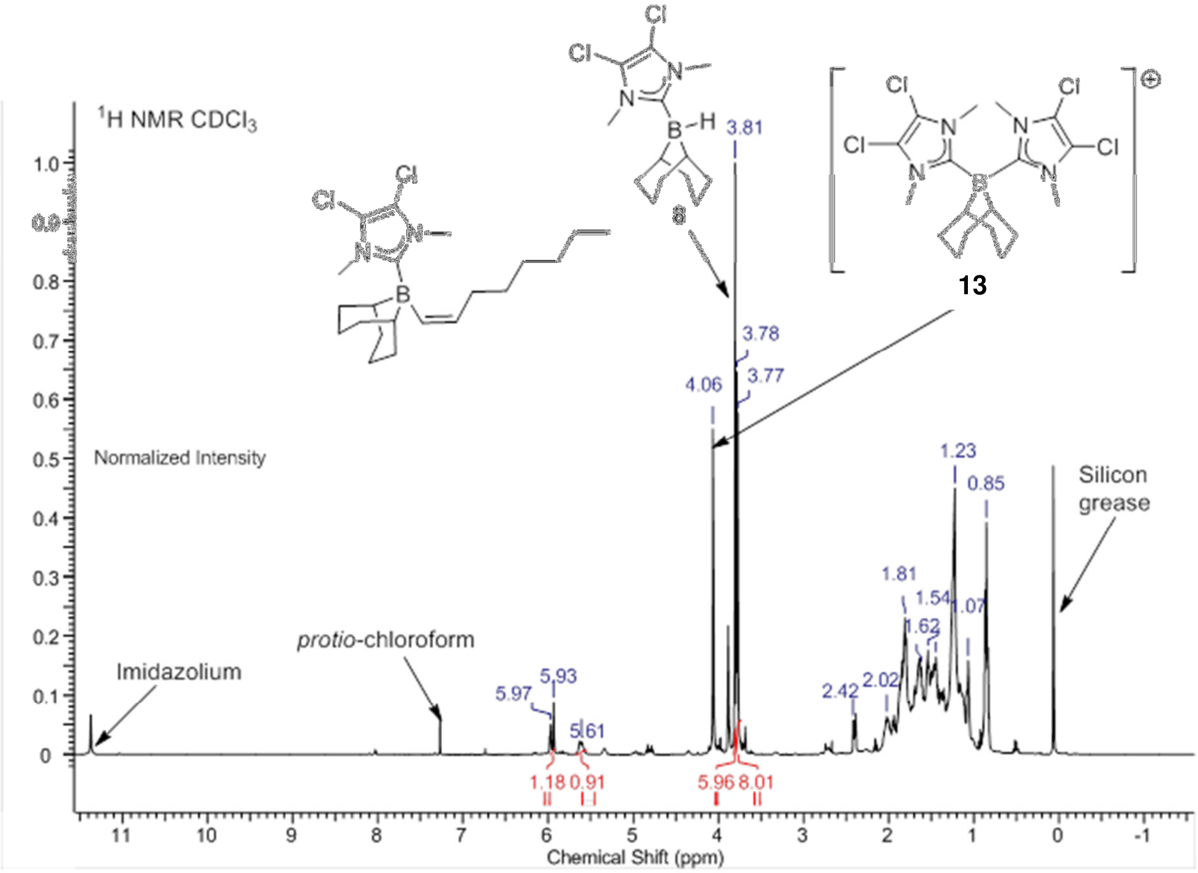

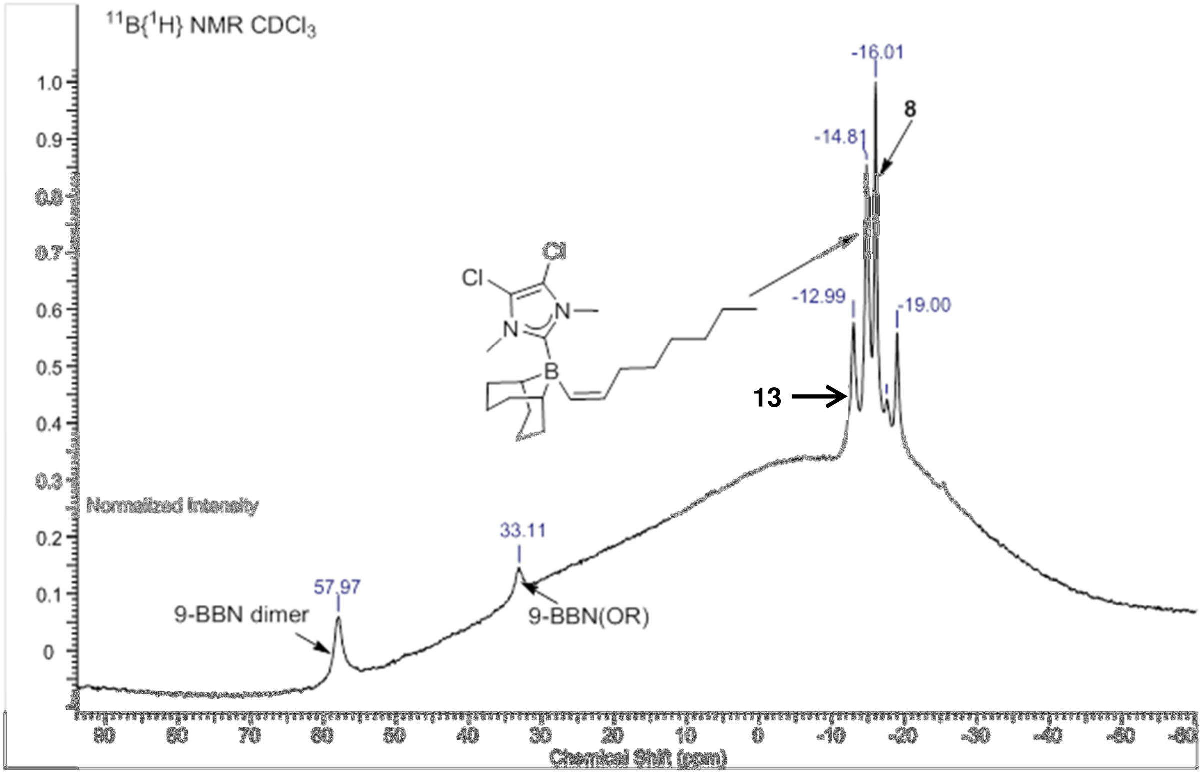

Crude mixture containing 9-BBN(I-DCDM)(1-(*Z*-oct-1-ene-d$_1$)) (**10m$_D$**) as the major new product

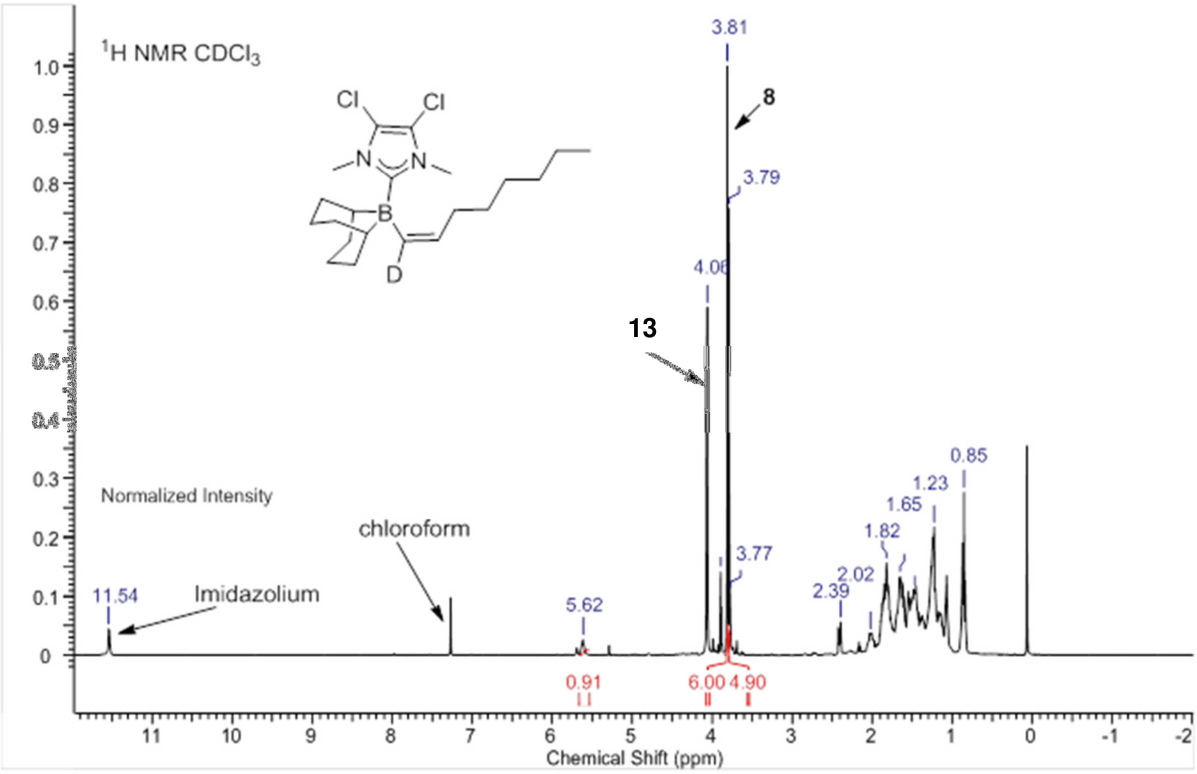

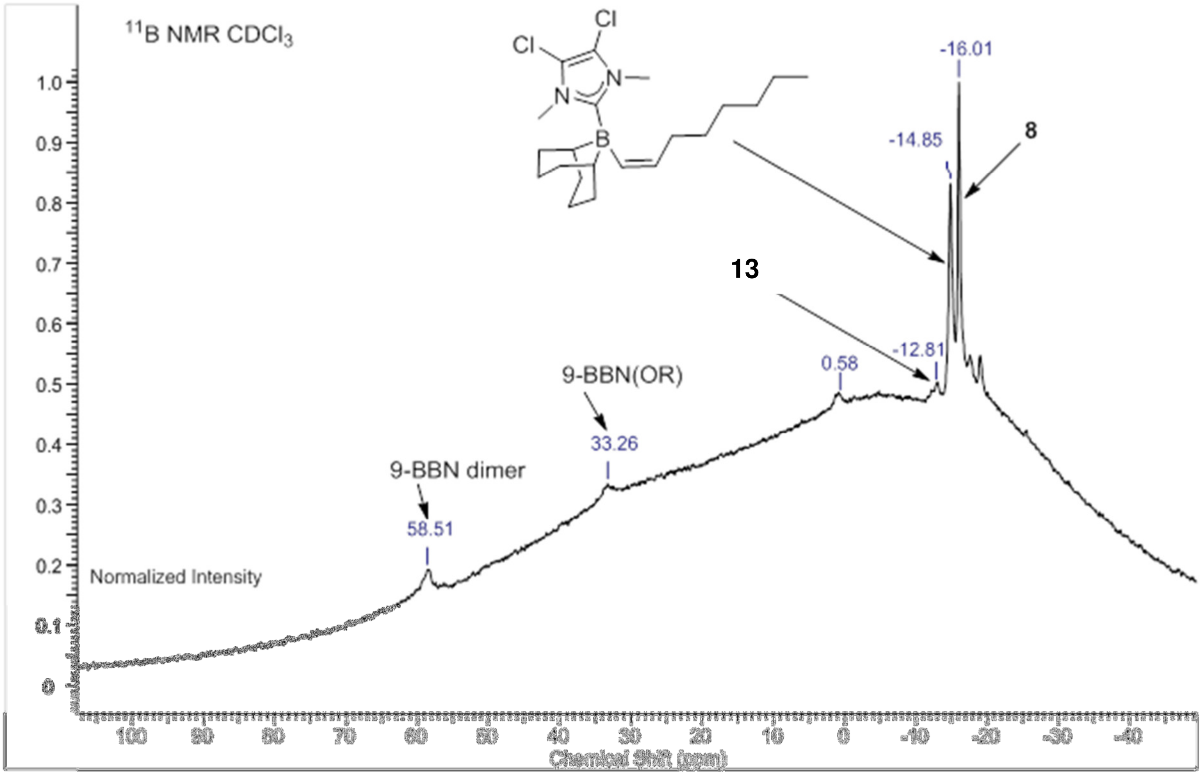

NMR spectra for 2-(9-BBN(I-DCDM))-3-(*para*-(*N,N*-dimethyl)aniline)-*E*-prop-2-ene) (**10n**)

Below are provided the in-situ NMR spectra in the presence of mesitylene used to determine the % conversion (the ^{1}H NMR spectra shows unreacted **8** and alkyne as the major other species present in solution along with **10n**). Also provided are the NMR spectra after attempted work up. This sample is sensitive to protodeboronation (significant amounts of **10n** is converted to the protodeboronated product on exposure to “wet solvent”) therefore the conversion is reported in-situ Vs. mesitylene. NMRs are provided in C_6D_6 on the “worked up” products for better separation of relevant resonances (facilitating analysis by NOESY).

In-situ NMR spectrum (with mesitylene)

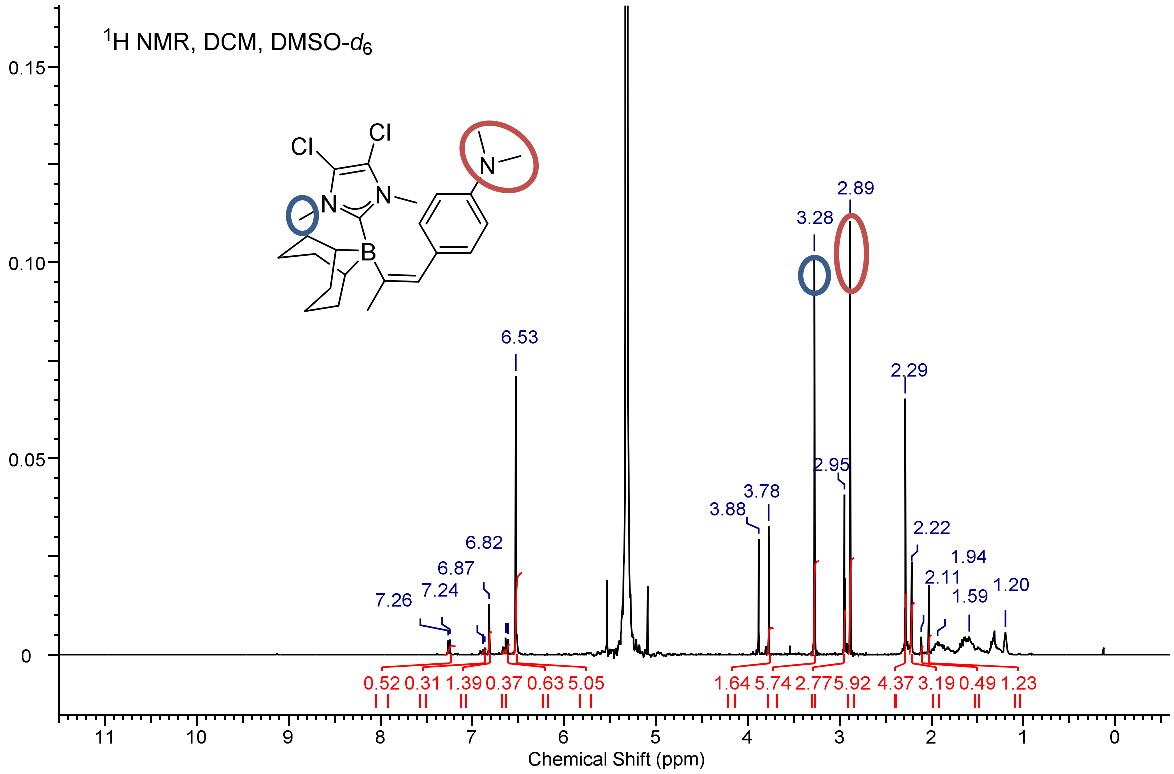

Spectra after attempted “work up”

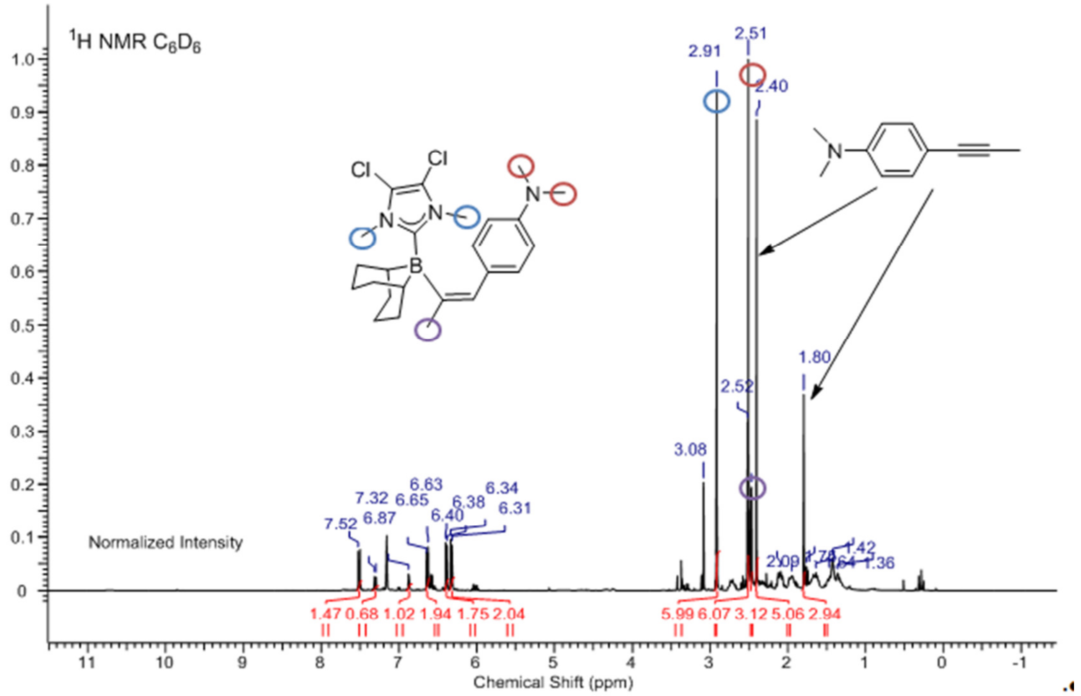

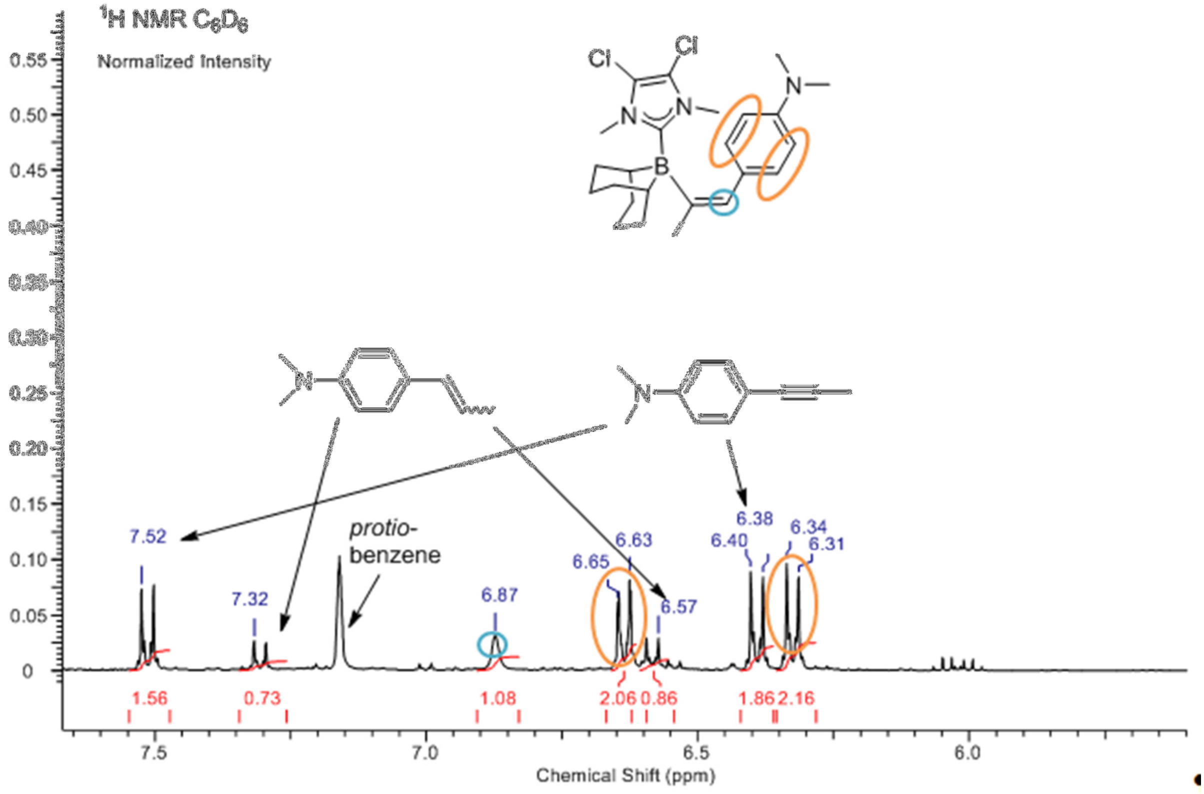

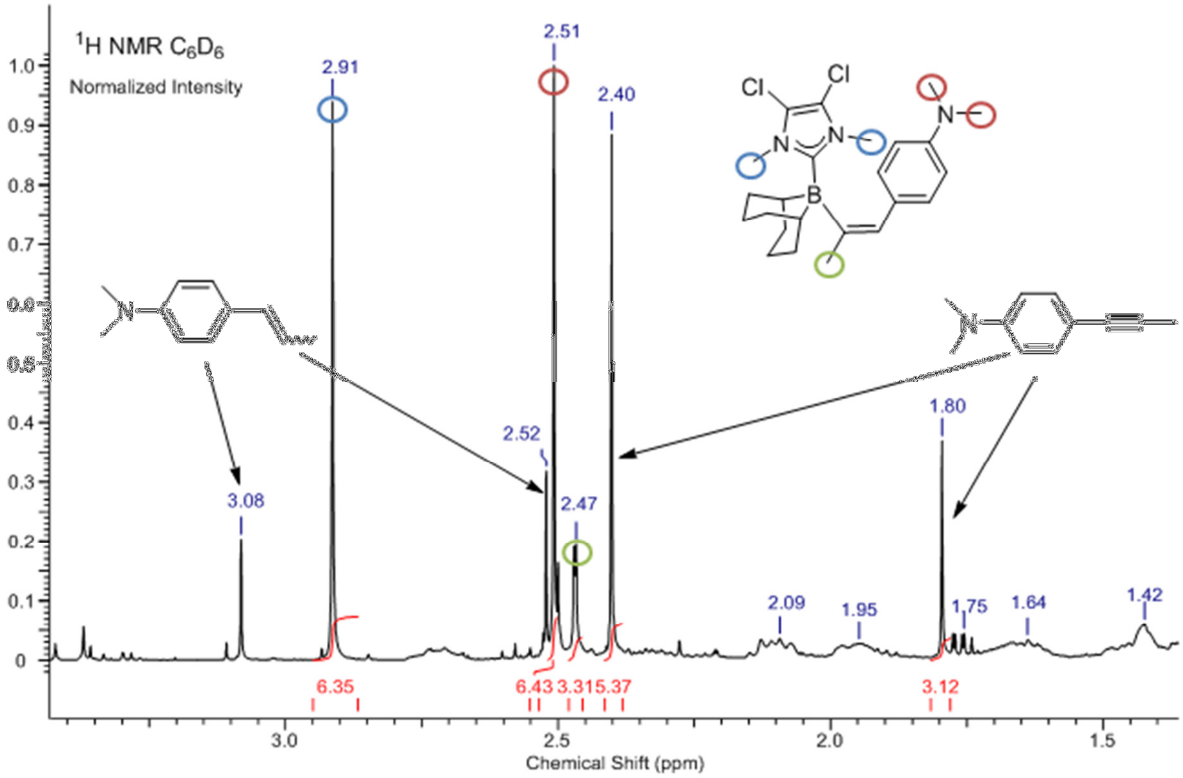
^{1}H NMR C_6D_6
Normalized Intensity
2.91
2.51
2.40
2.52
3.08
2.47
1.80
2.09
1.95
1.75
1.64
1.42
6.35
6.43
3.31
5.37
3.12
Cl
Cl
N
N
B
Chemical Shift (ppm)

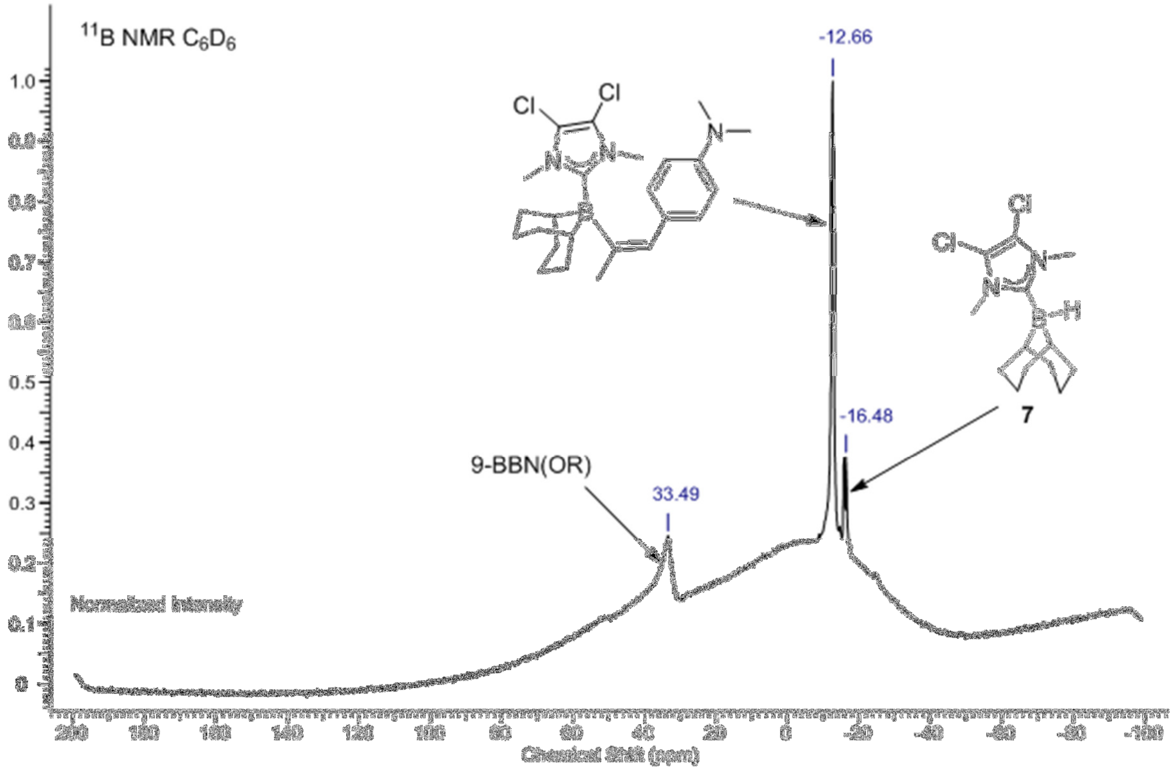
^{11}B NMR C_6D_6
-12.66
-16.48
33.49
9-BBN(OR)
7
Cl
Cl
N
N
B
H
Normalized Intensity
Chemical Shift (ppm)

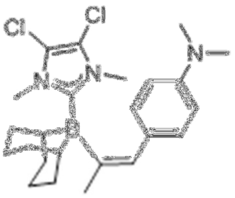

2D NOESY (^1H:^1H), C_6D_6

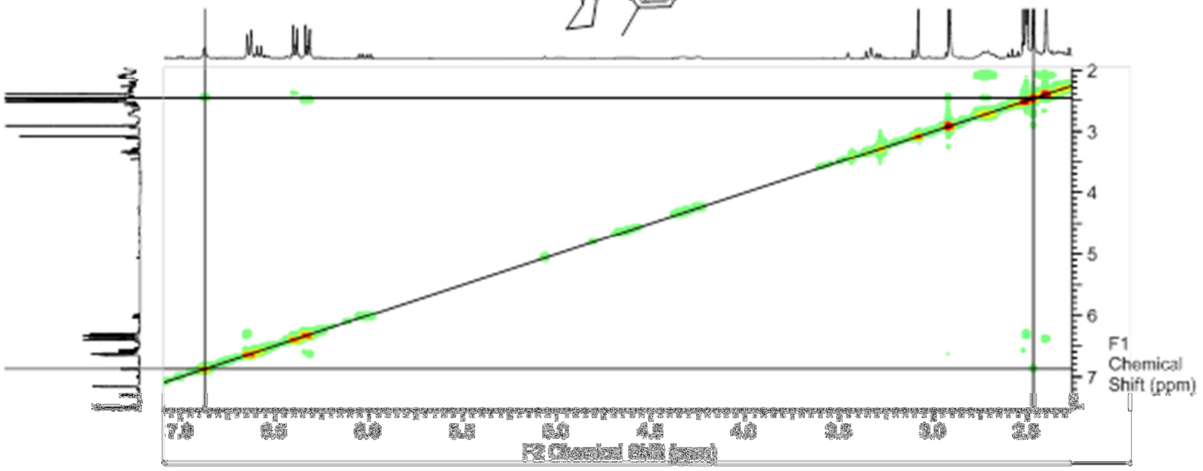

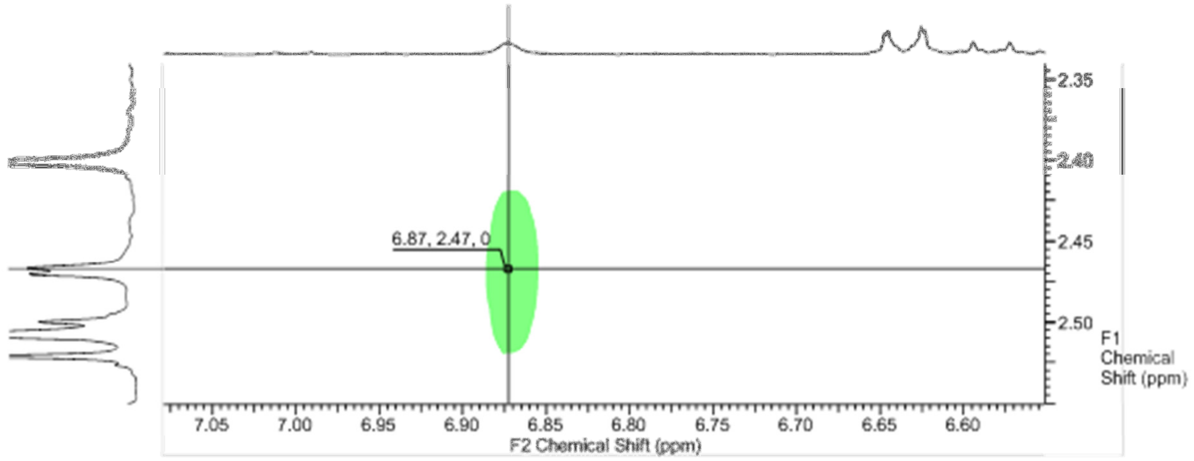

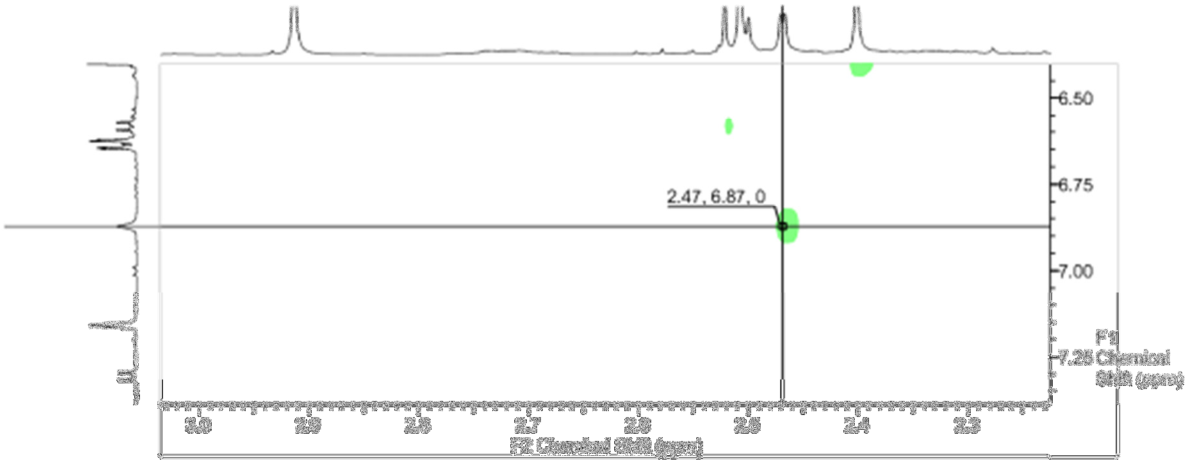

In-Situ ^{19}F and ^{11}B NMR spectra of the hydroboration of alkynes with **8** and 5 mol% $B(C_6F_5)_3$ to give **10x** and minor ionic by-products

Sets of C_6F_5 resonances can be seen for both the $[HB(C_6F_5)_3]^-$ anion, as well as $[(vinyl)B(C_6F_5)_3]^-$, and other minor unknown by products.

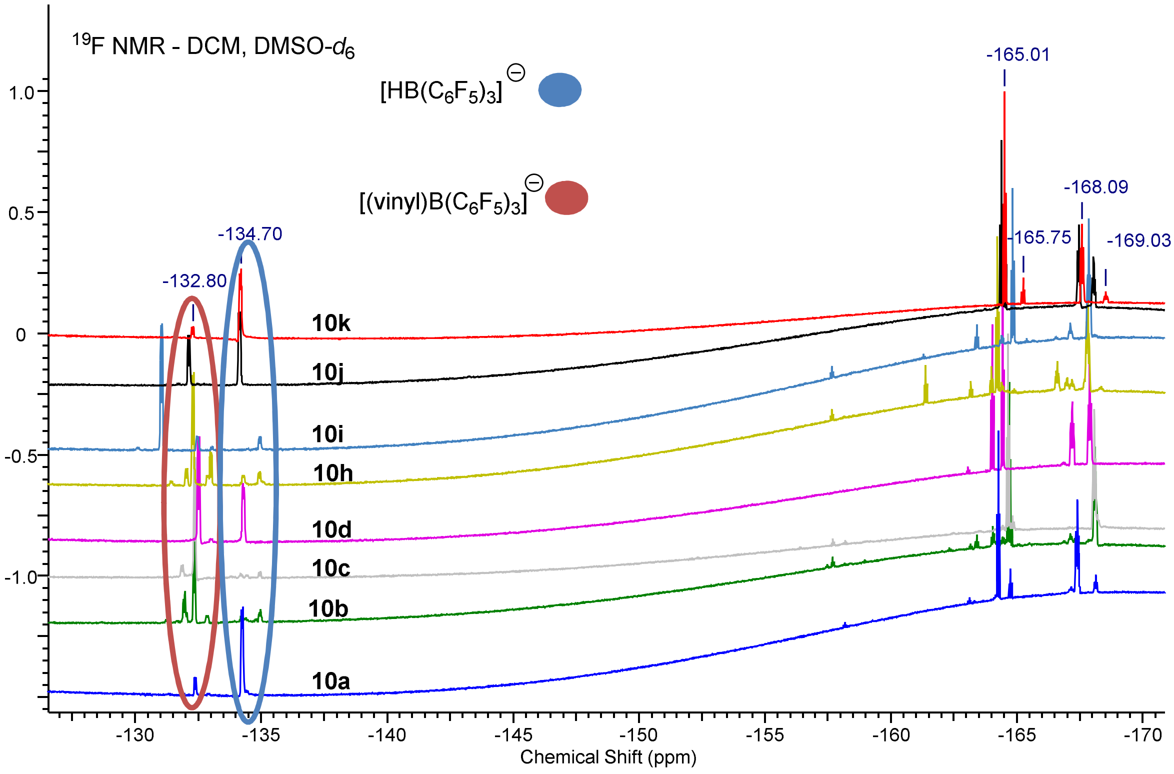

A representative crude ^{11}B NMR spectrum is shown below pre work up. With the minor products **13**, and $[vinylB(C_6F_5)_3]^-$ clearly visible.

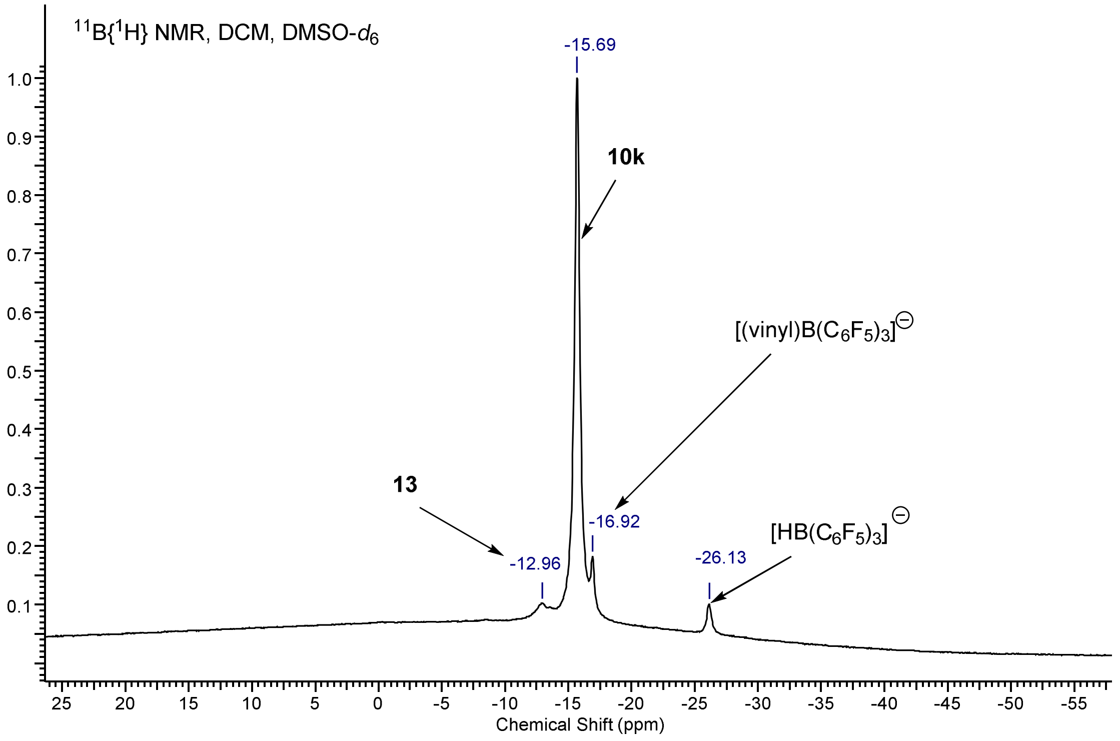

NMR spectra for the hydroboration of 4-ethynyltoluene with 1 eq **9**.

The reaction proceeds via trans-hydroboration, but NHC transfer is observed from the vinylBBN(NHC) to free $B(C_6F_5)_3$, forming vinylBBN.

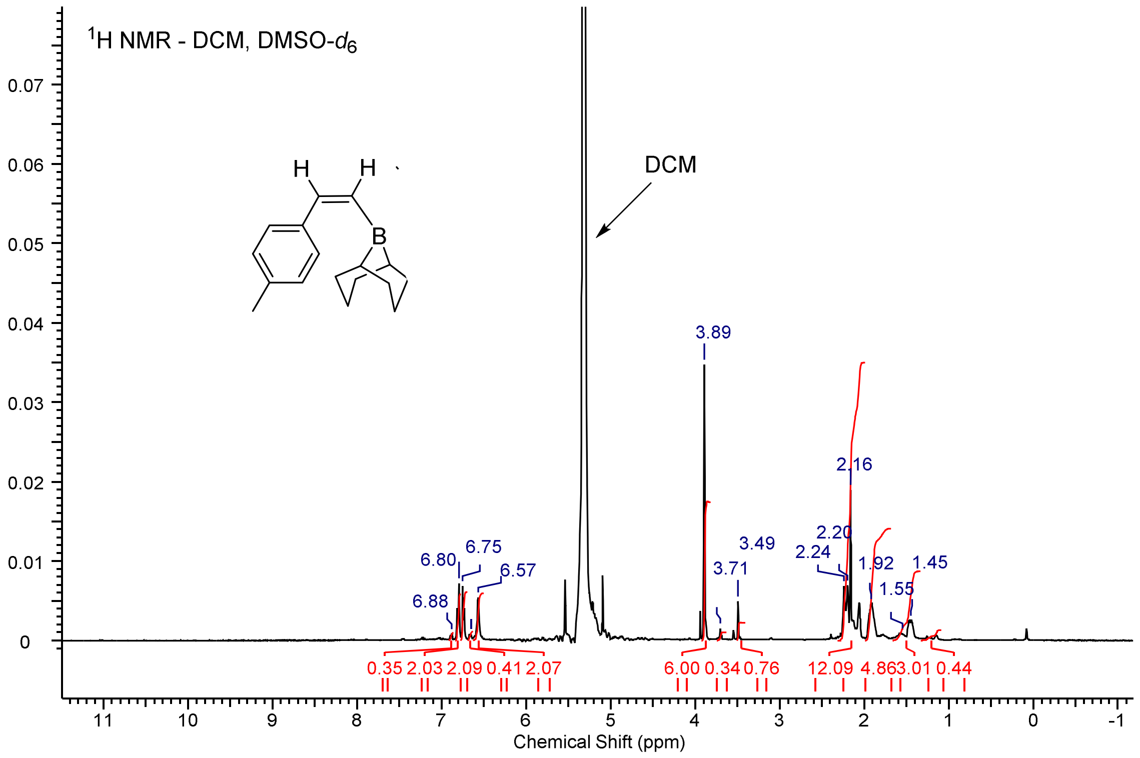

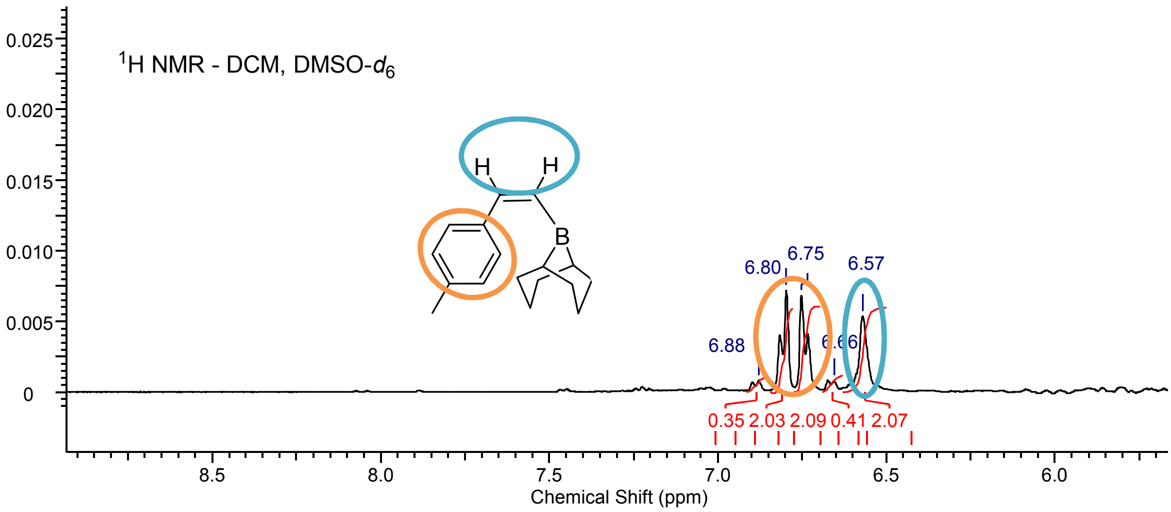

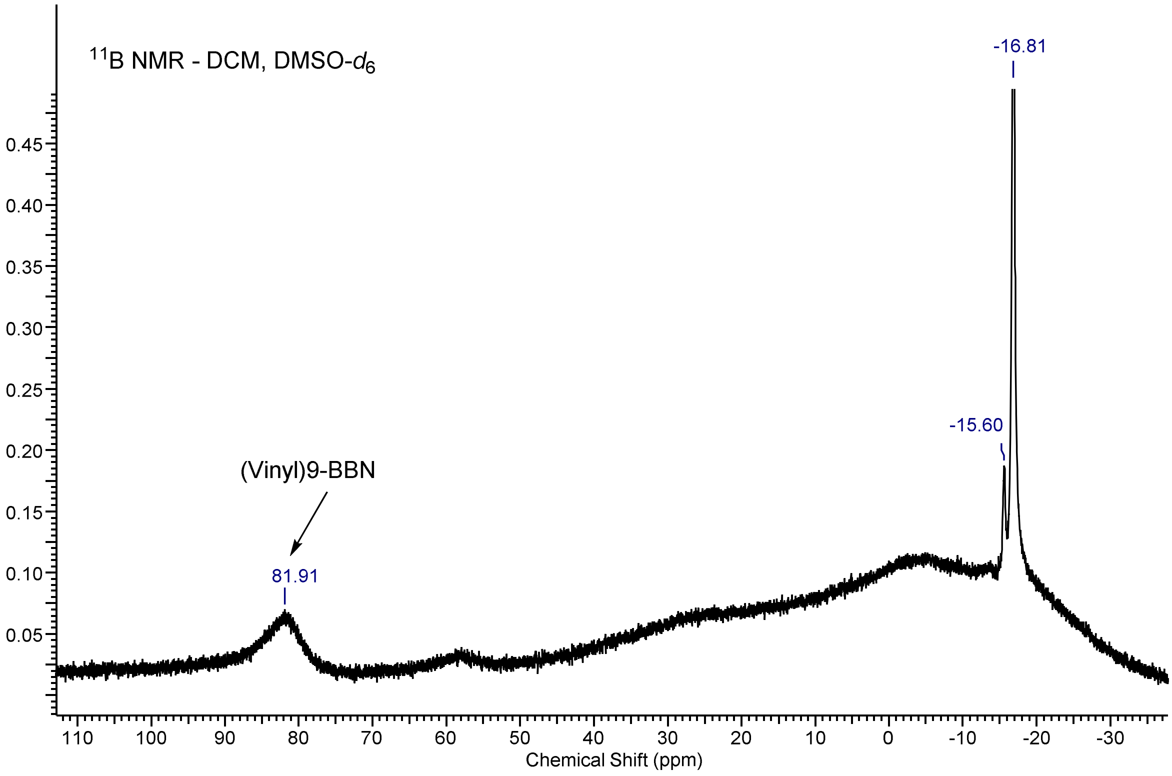

The by-products is tentatively assigned as NHC-B(C_6F_5)$_3$. For previous reports on NHC-B(C6F5)3 see: Chase, P. A.; Stephan,D.W. Angew. Chem., Int. Ed. 2008, 47, 7433.

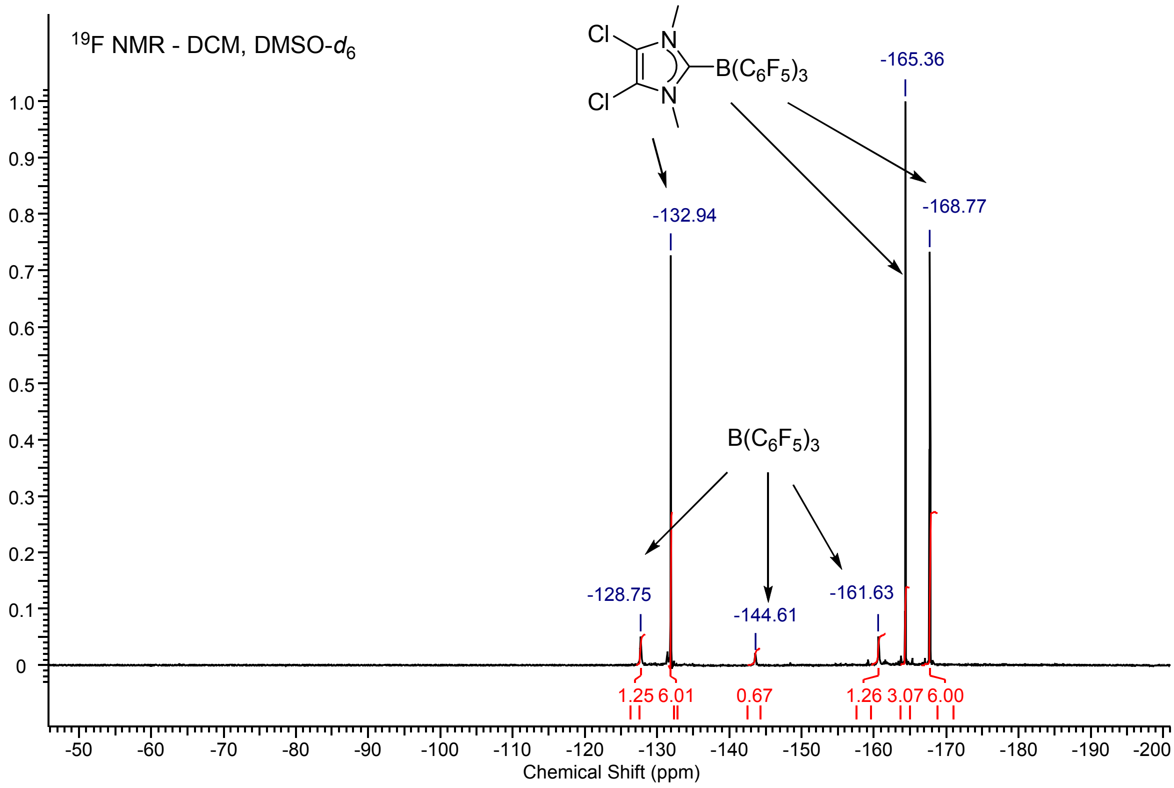

Sample dried and NMR spectra run in “wet” $CDCl_3$ to avoid coincidence of vinylic resonances.

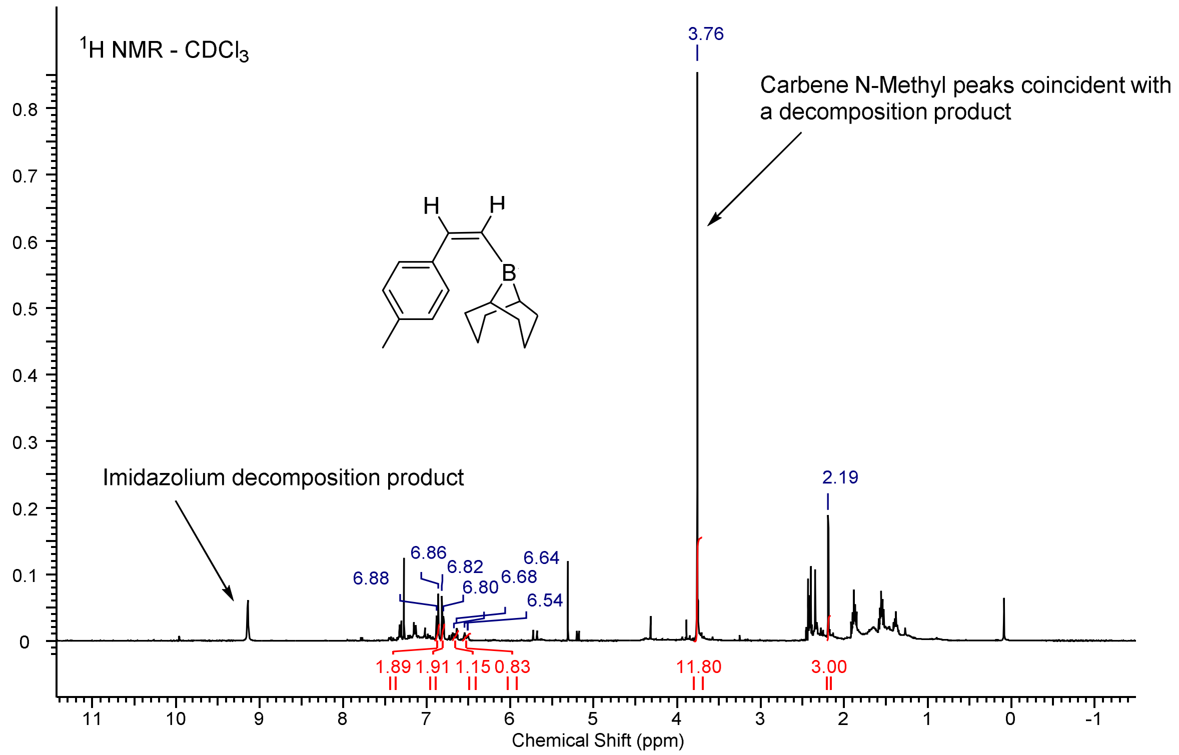

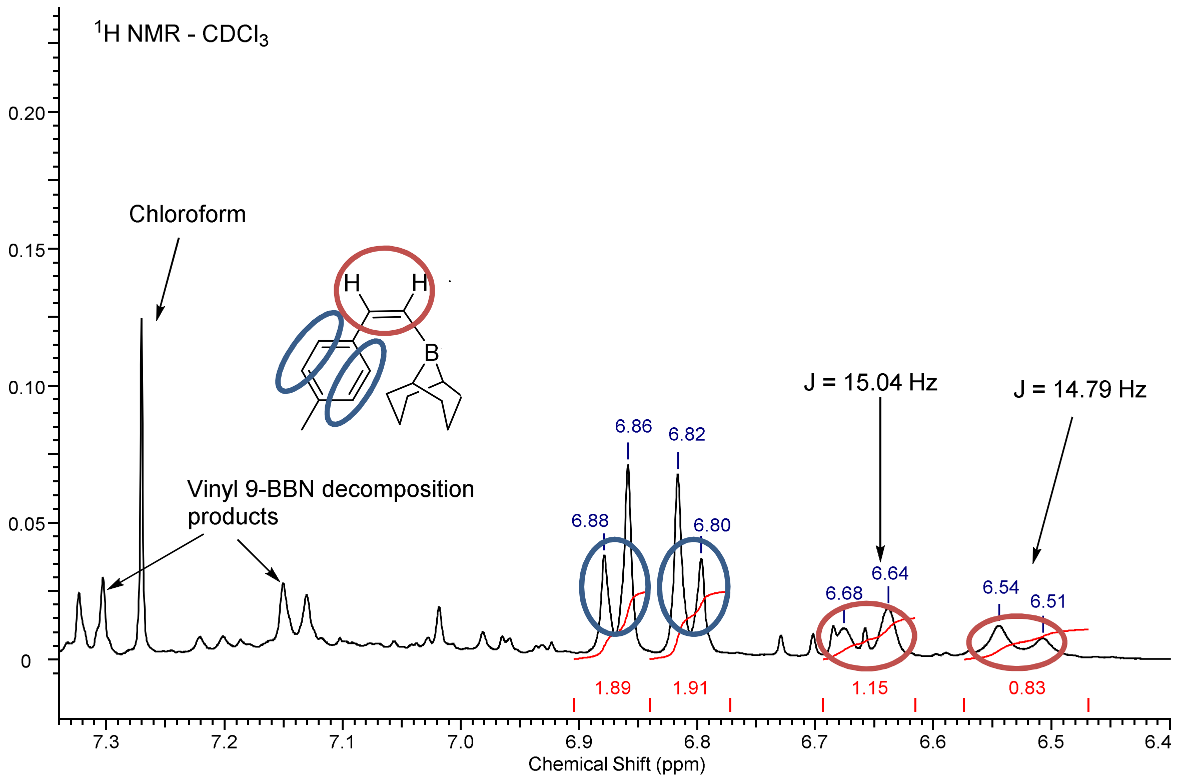

The 15 Hz coupling constant observed are fully constant with a Z-vinylBR_2 formulation.

In-situ NMR spectra for Carbene Removal from **10a** to form **11a** and BF_3(I-DCDM)

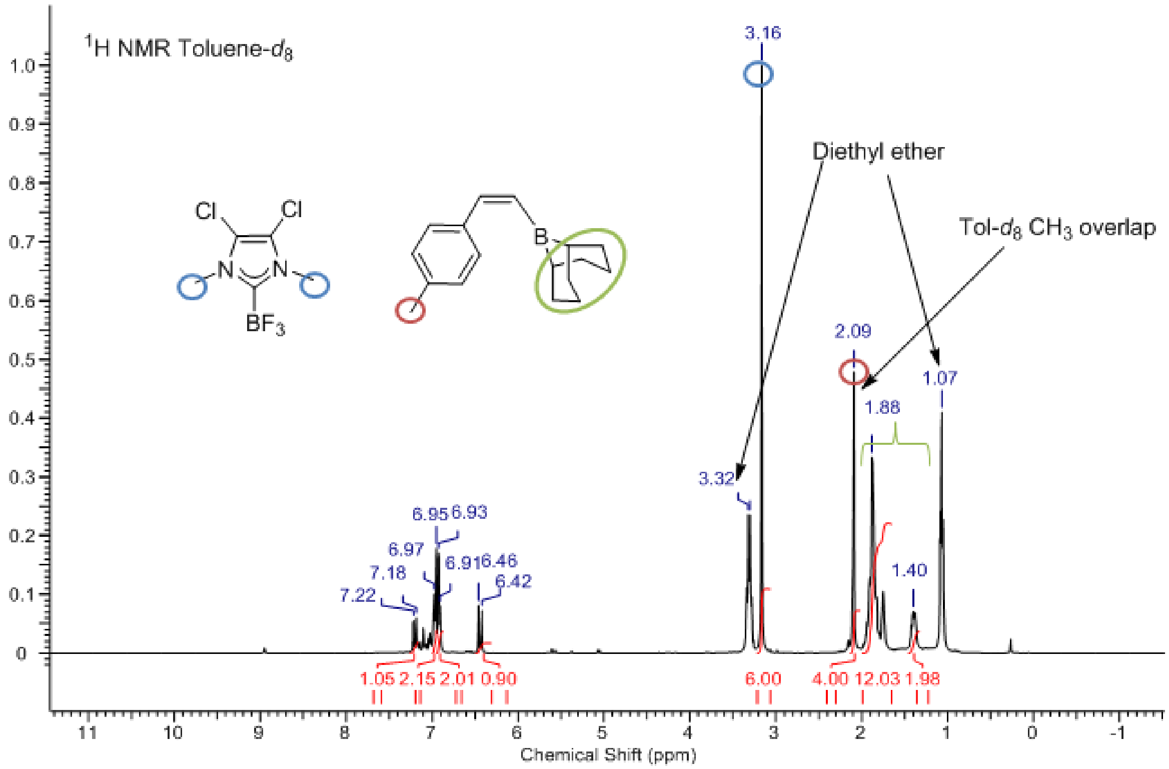

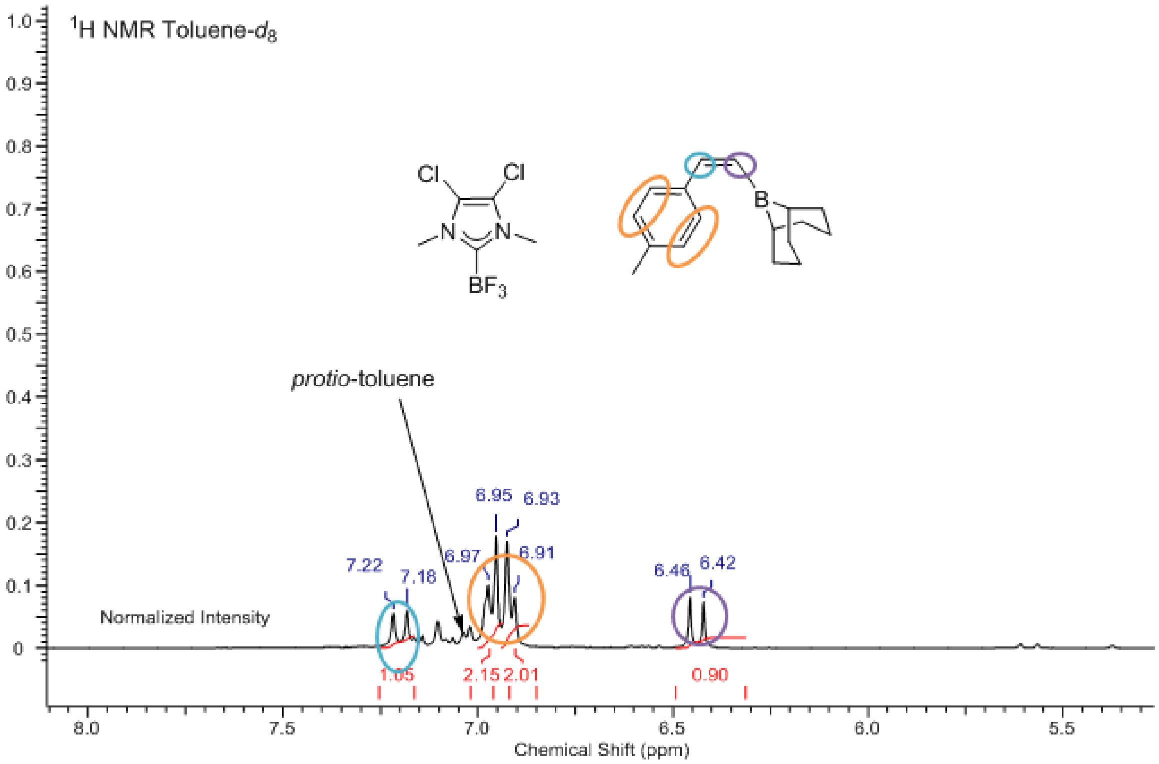

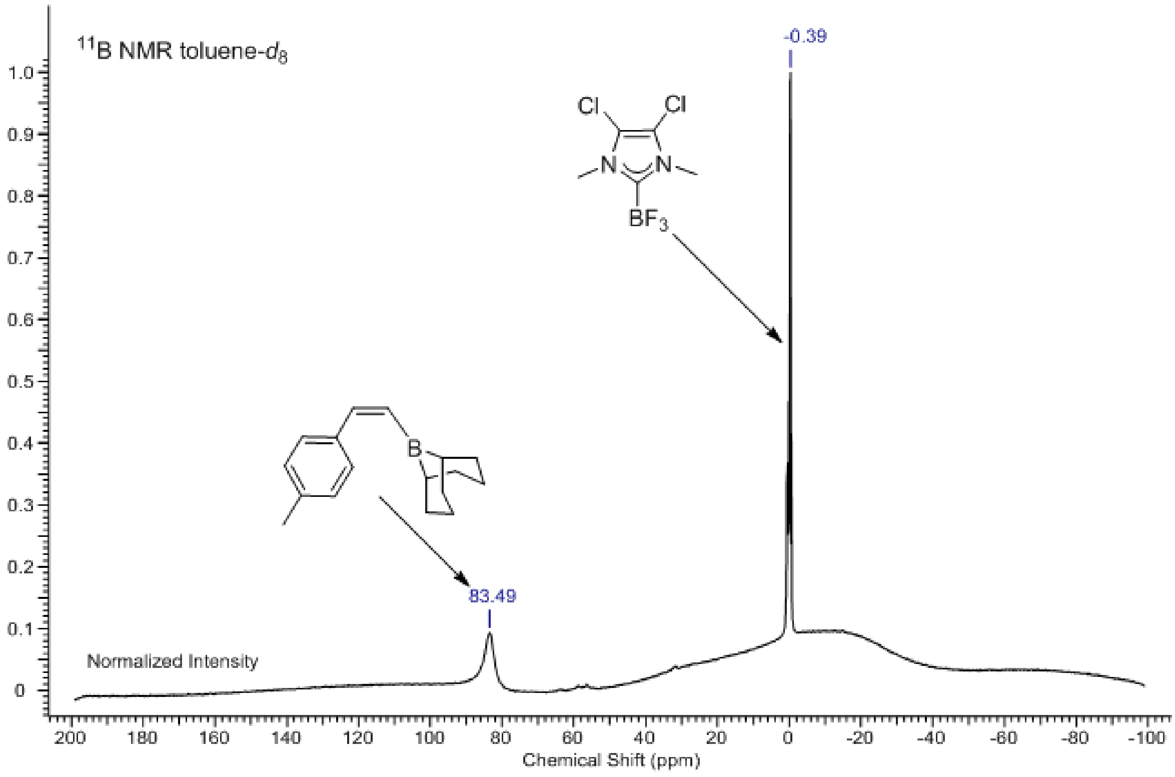
^{11}B NMR toluene-d_8
-0.39
83.49
Cl
Cl
N
N
BF3
B
Normalized Intensity
Chemical Shift (ppm)

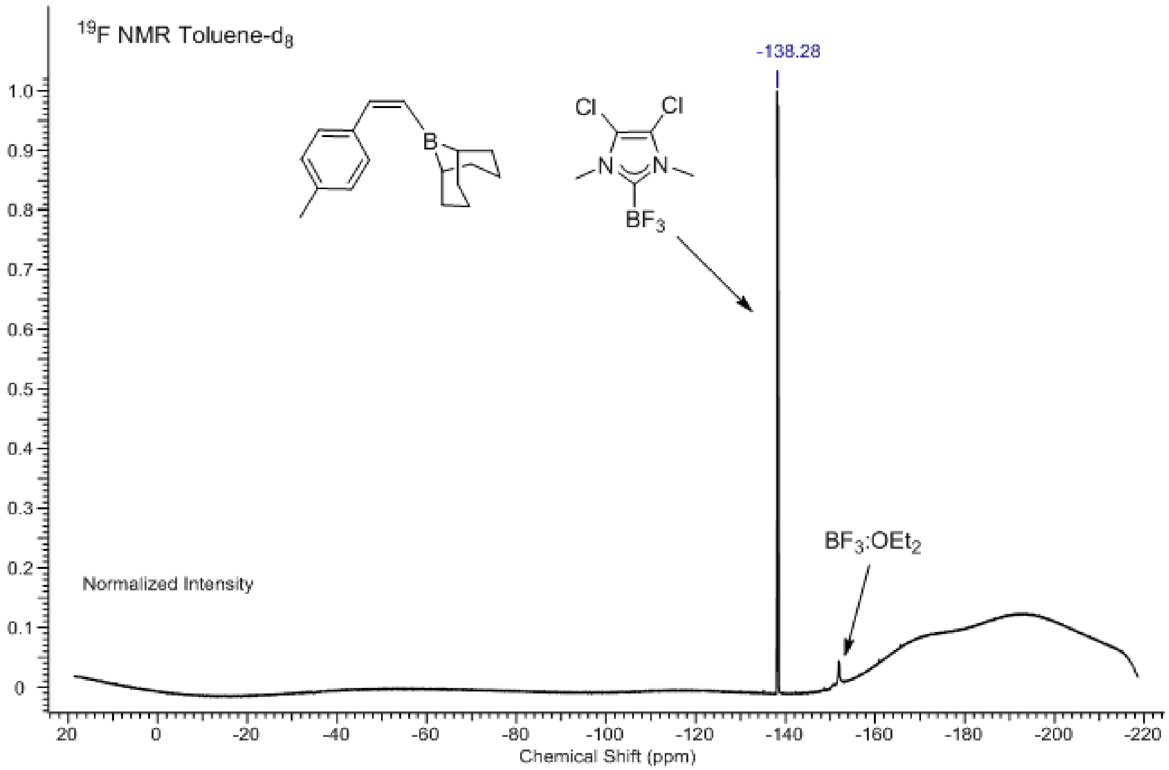
^{19}F NMR Toluene-d_8
-138.28
Cl
Cl
N
N
BF3
B
BF3:OEt2
Normalized Intensity
Chemical Shift (ppm)

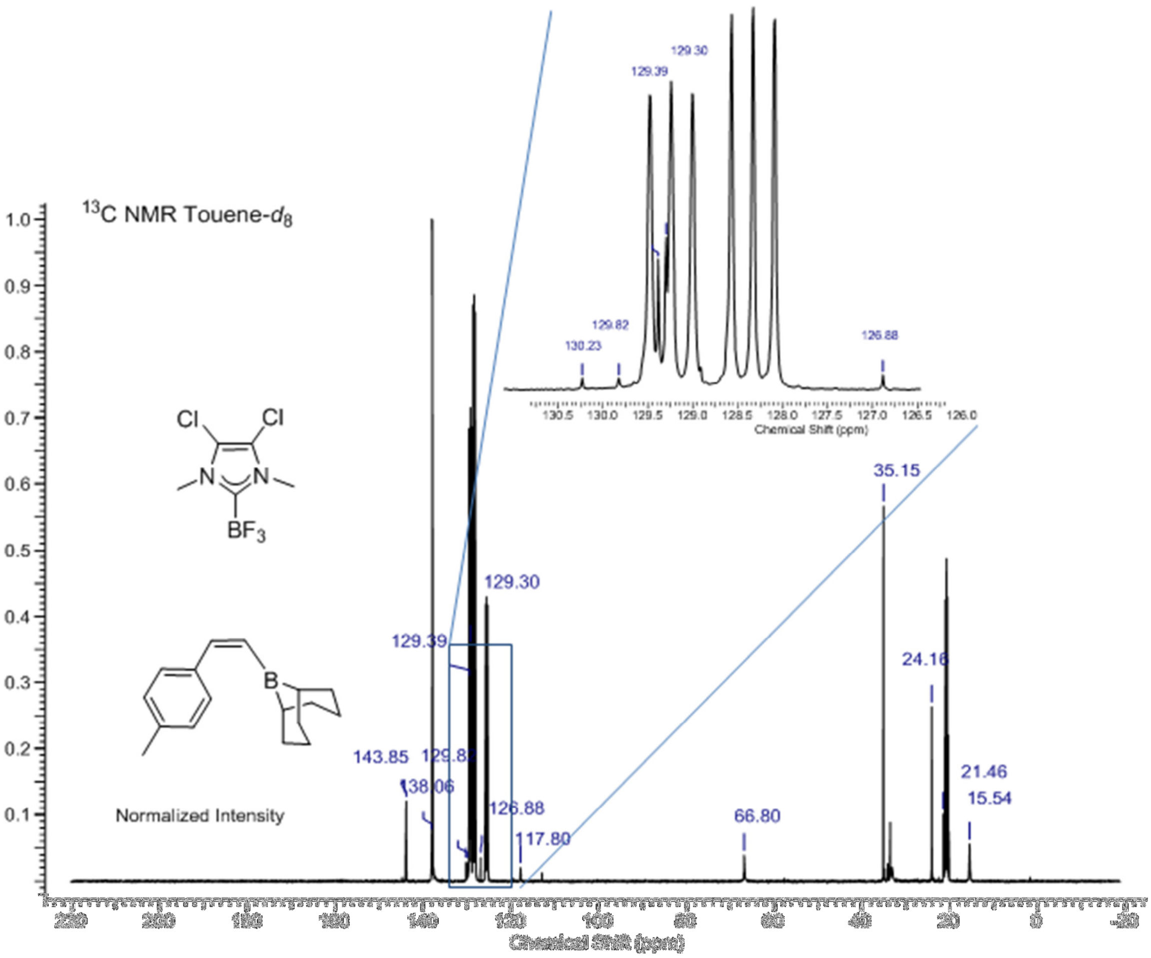

13C NMR Touene-d8
Normalized Intensity
Chemical Shift (ppm)
143.85
138.06
129.82
129.39
129.30
126.88
117.80
66.80
35.15
24.16
21.46
15.54
130.23
Cl
N
BF3
B

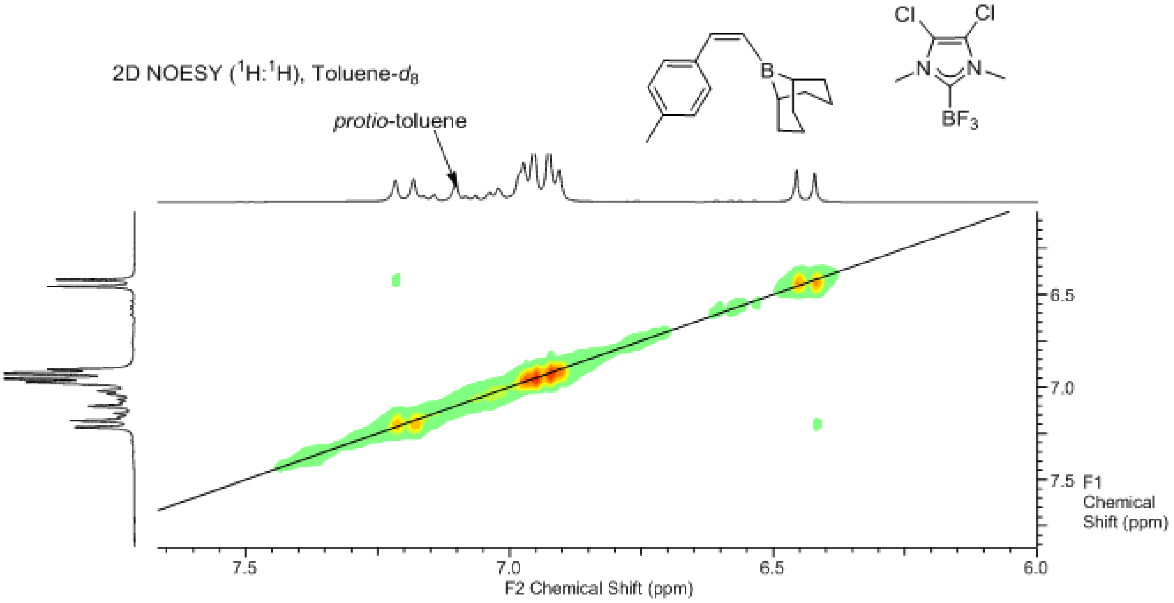

2D NOESY (1H:1H), Toluene-d8
protio-toluene
Cl
N
BF3
B
F2 Chemical Shift (ppm)
F1 Chemical Shift (ppm)
7.5
7.0
6.5
6.0

In-situ NMR spectra for Carbene Removal from **10c** to form **11c** and BF_3(I-DCDM)

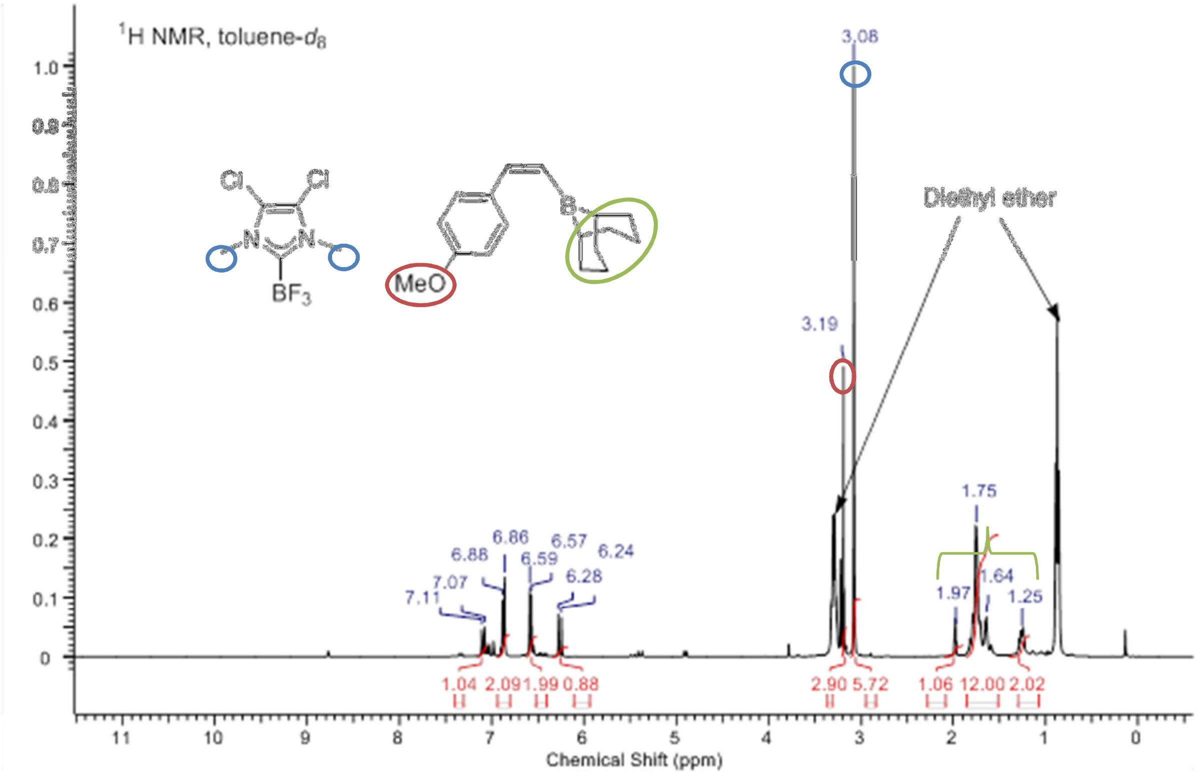

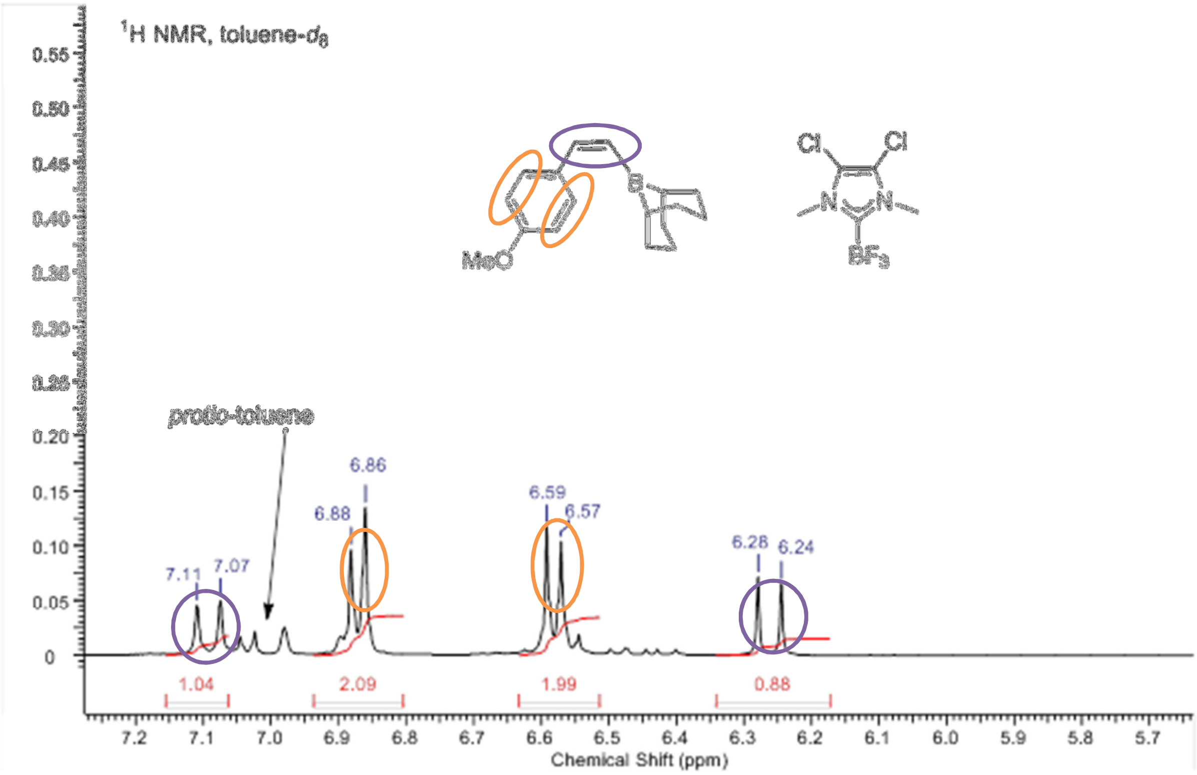

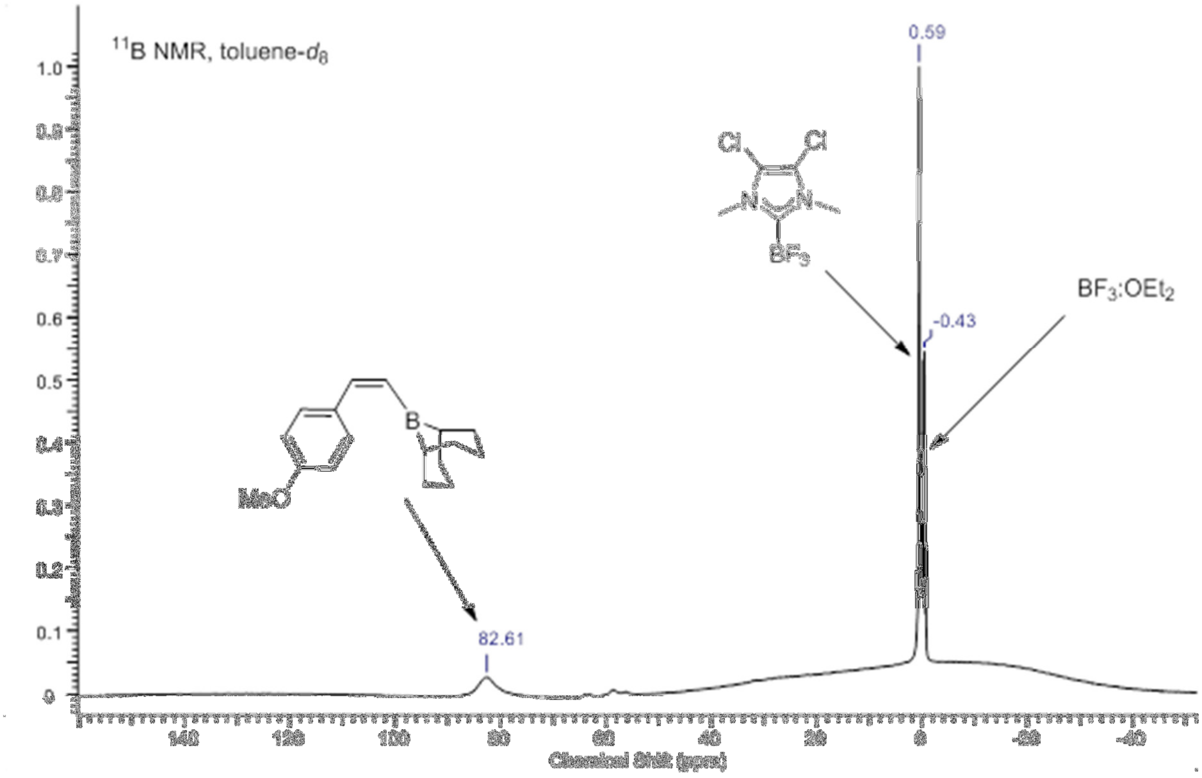

11B NMR, toluene-d8
0.59
-0.43
BF3:OEt2
82.61
MeO
Chemical Shift (ppm)

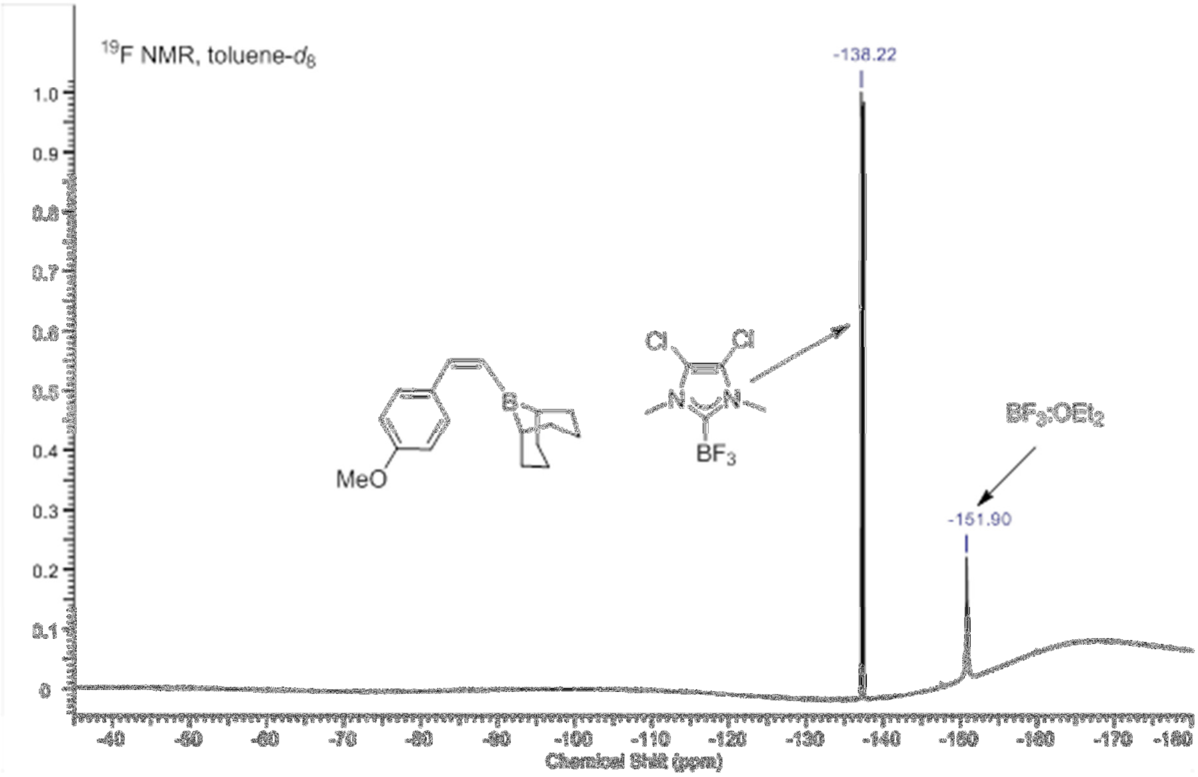

19F NMR, toluene-d8
-138.22
BF3:OEt2
-151.90
MeO
Chemical Shift (ppm)

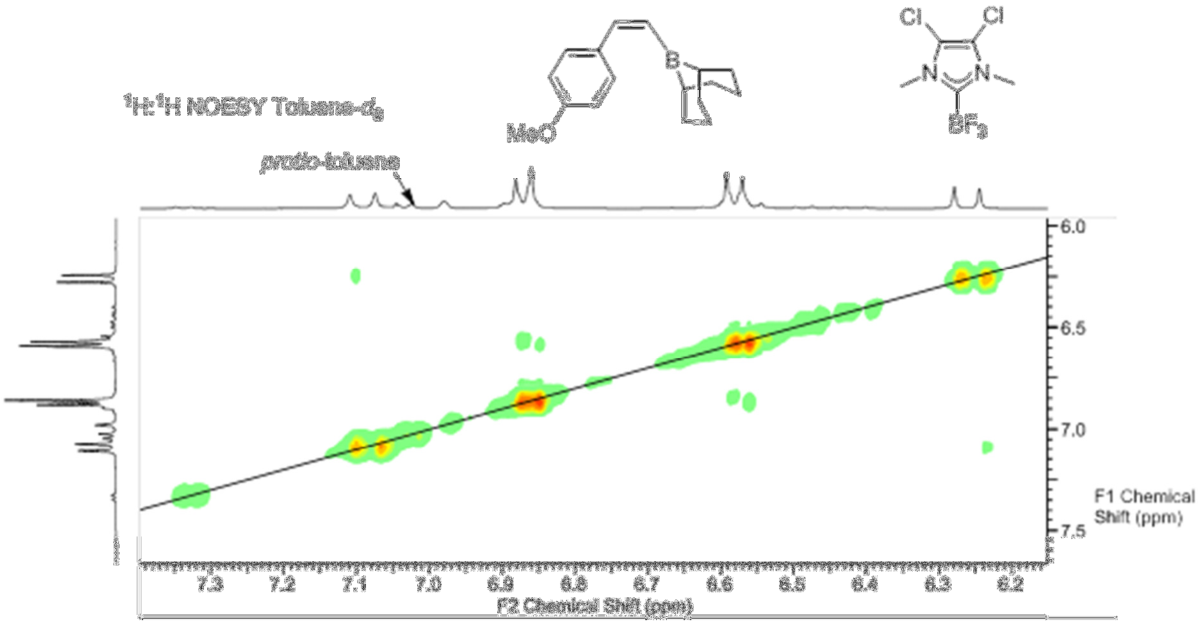

^1H:^1H NOESY Toluene-d_8
protio-toluene
MeO
B
Cl
Cl
N
N
BF$_3$
F1 Chemical Shift (ppm)
F2 Chemical Shift (ppm)

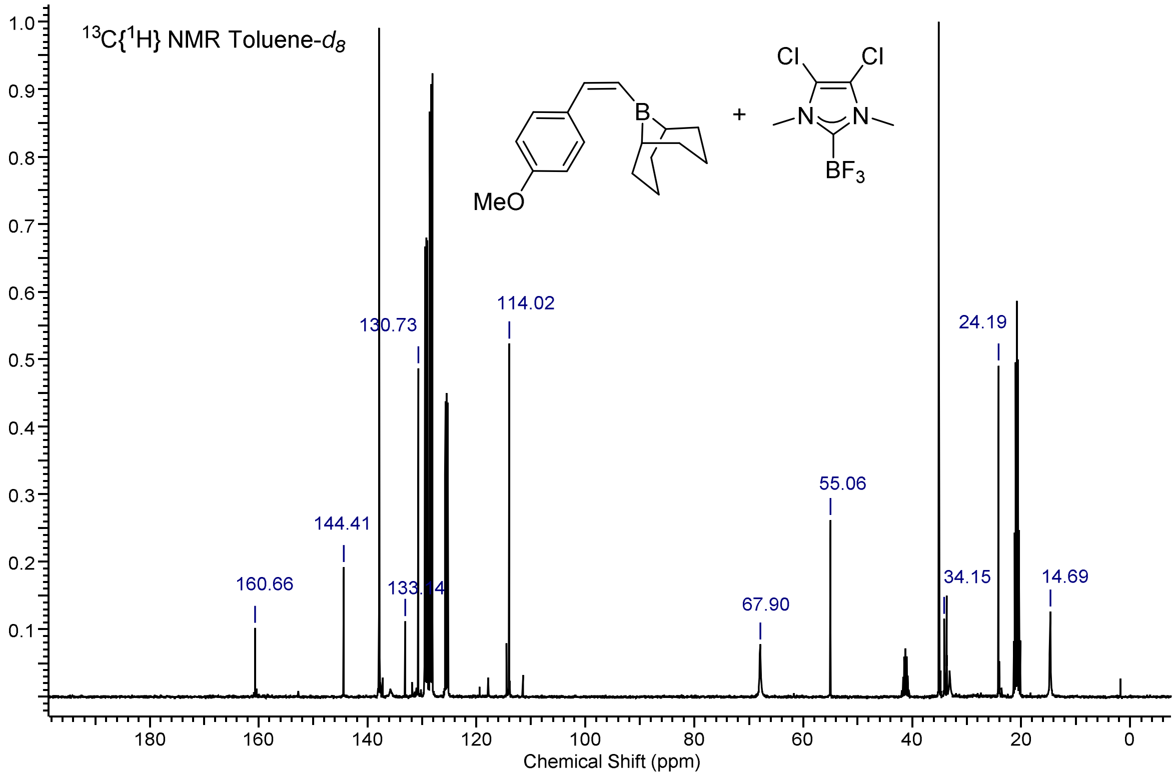

$^{13}C\{^1H\}$ NMR Toluene-d_8
MeO
B
+
Cl
Cl
N
N
BF$_3$
160.66
144.41
133.14
130.73
114.02
67.90
55.06
34.15
24.19
14.69
Chemical Shift (ppm)

4-Methyl-4′-Fluoro-*Z*-Stilbene (**12a**)

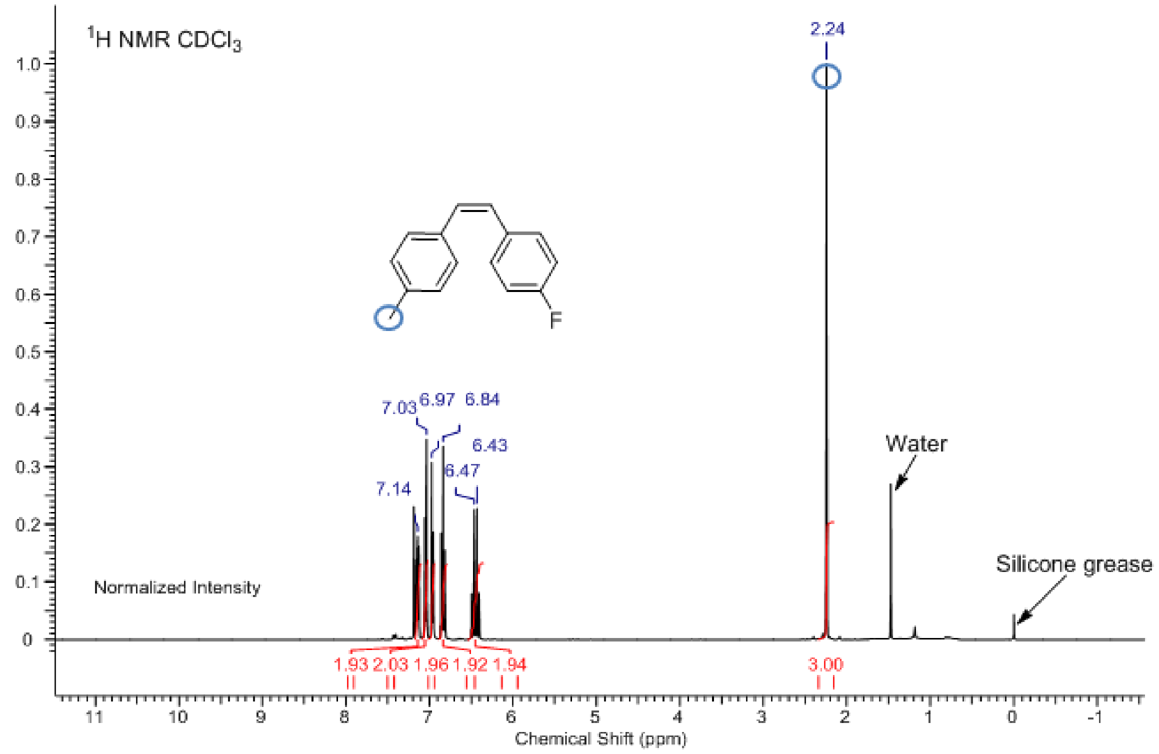

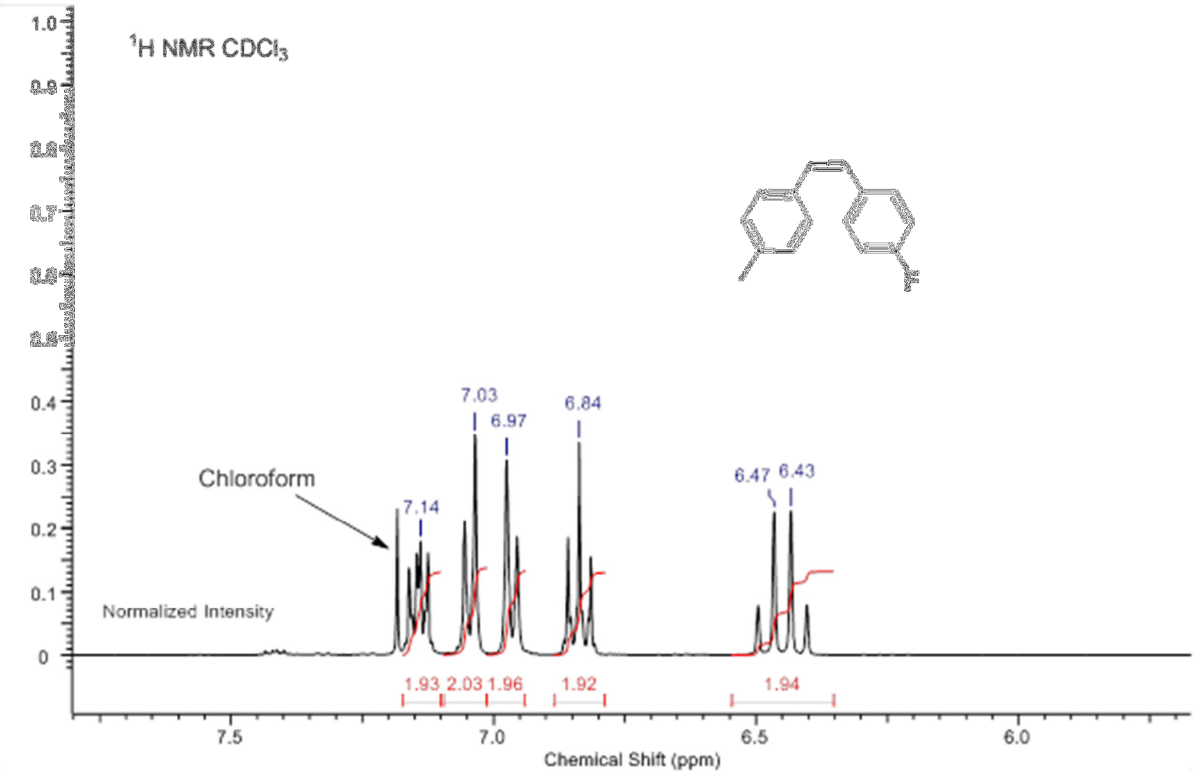

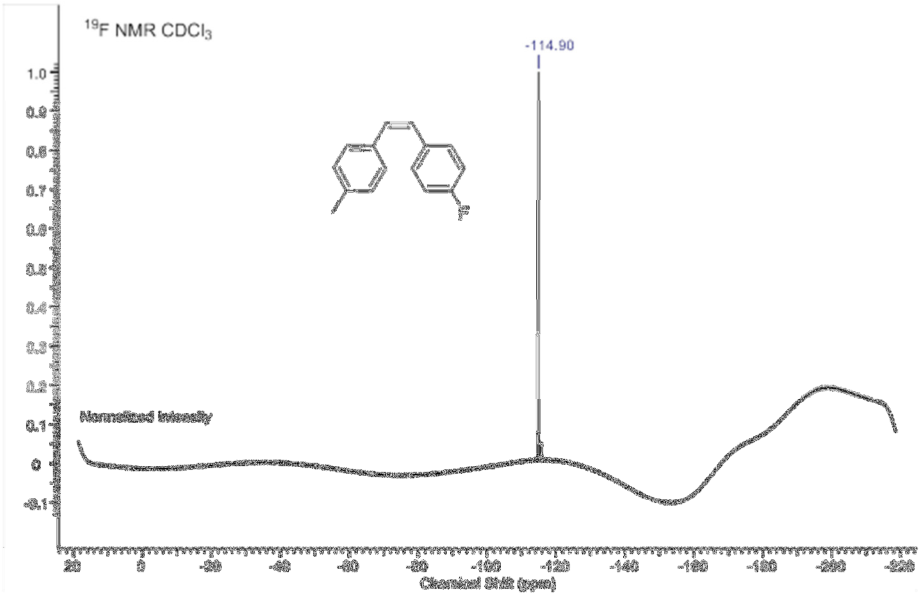

19F NMR CDCl3
-114.90
F
Normalized Intensity
Chemical Shift (ppm)

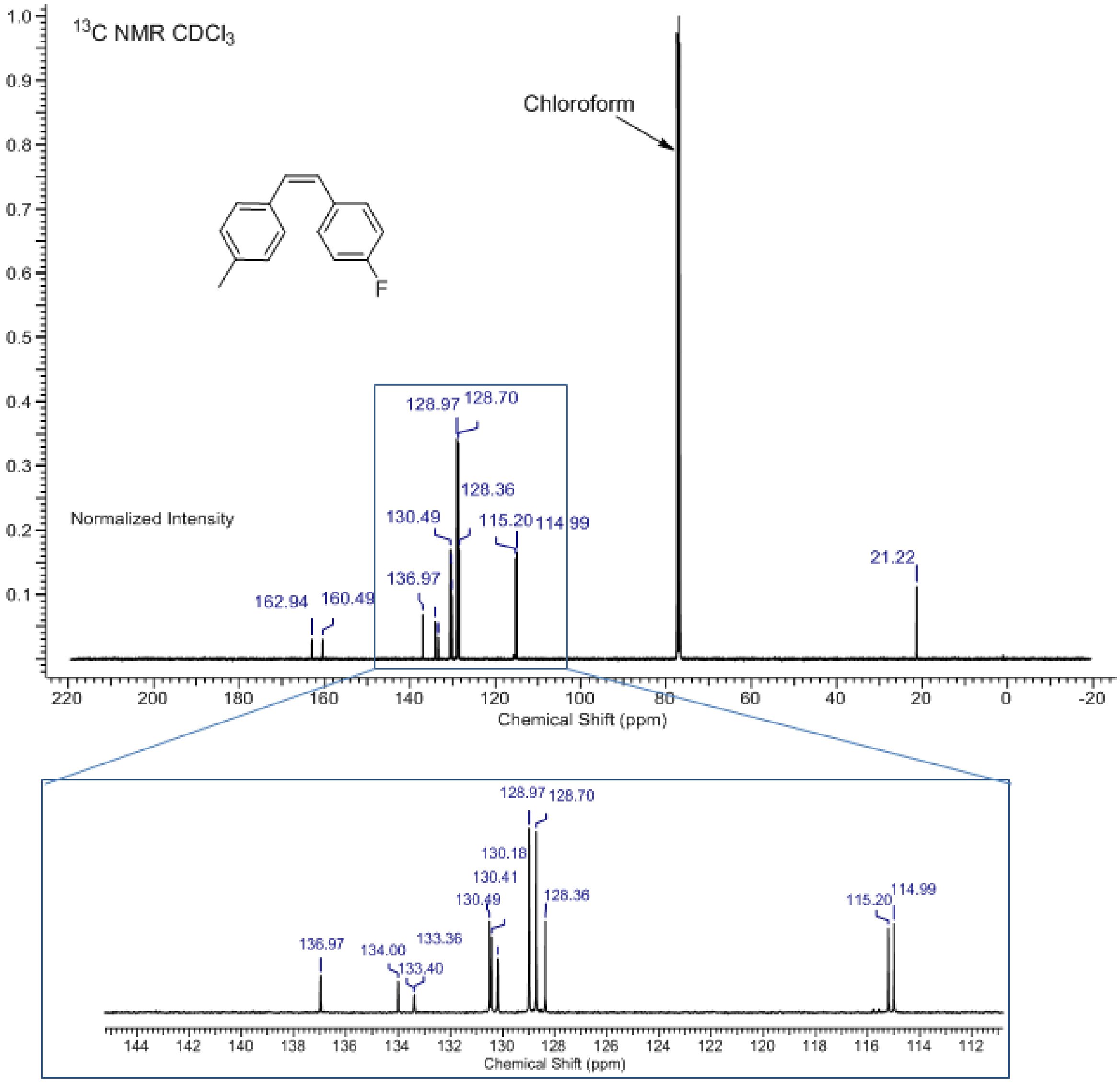

13C NMR CDCl3
Chloroform
F
Normalized Intensity
162.94
160.49
136.97
130.49
128.97
128.70
128.36
115.20
114.99
21.22
Chemical Shift (ppm)
136.97
134.00
133.40
133.36
130.49
130.41
130.18
128.97
128.70
128.36
115.20
114.99
Chemical Shift (ppm)

4-Methoxy-4'-Fluoro-*Z*-Stilbene (**12c**)

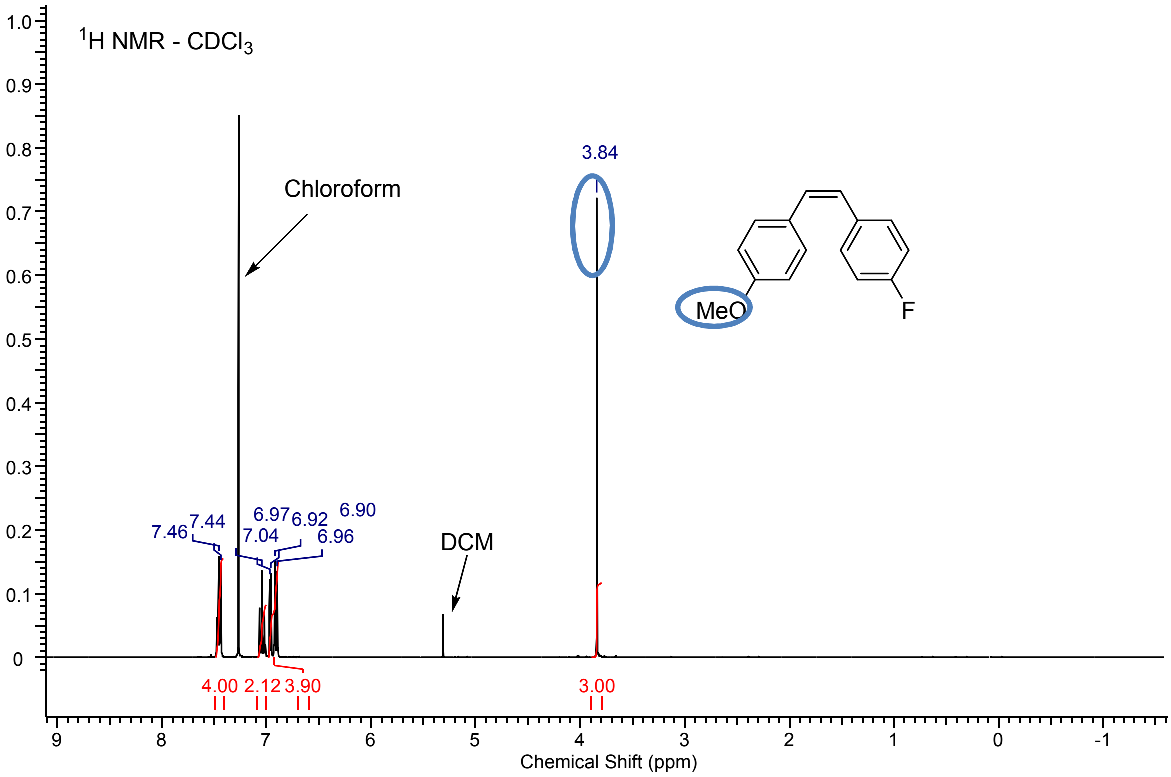

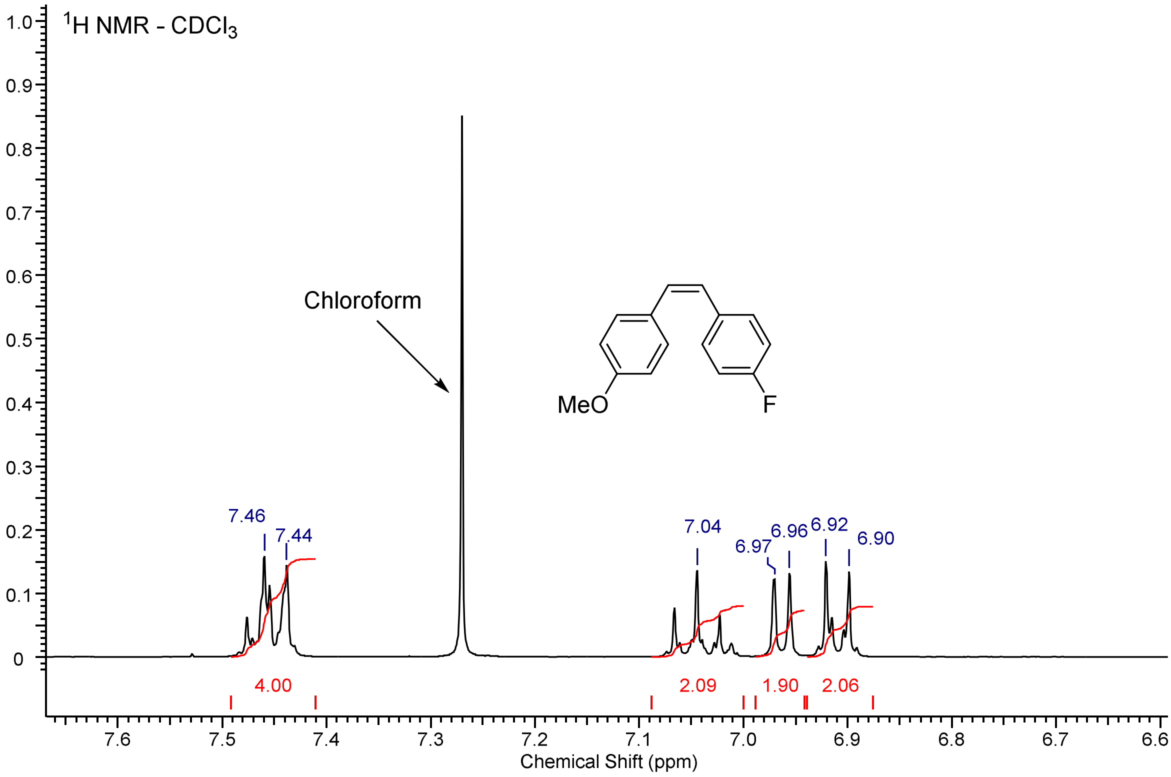

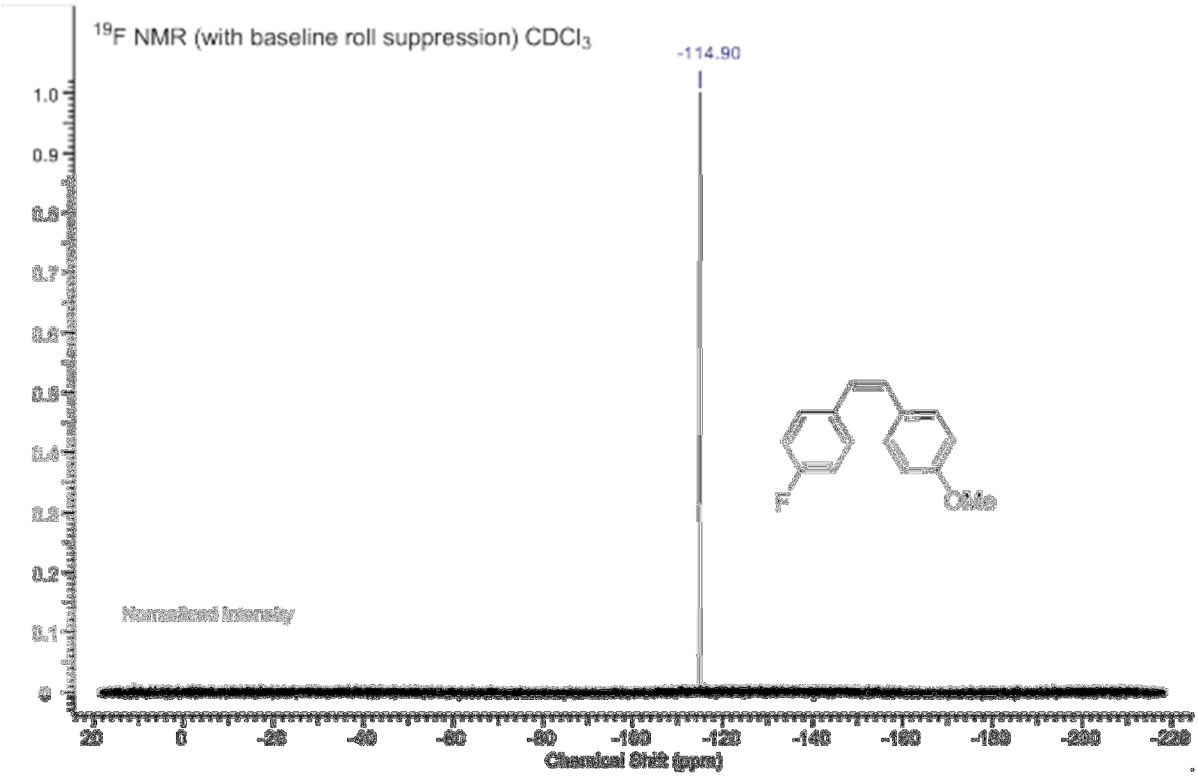

19F NMR (with baseline roll suppression) CDCl3
-114.90
F
OMe

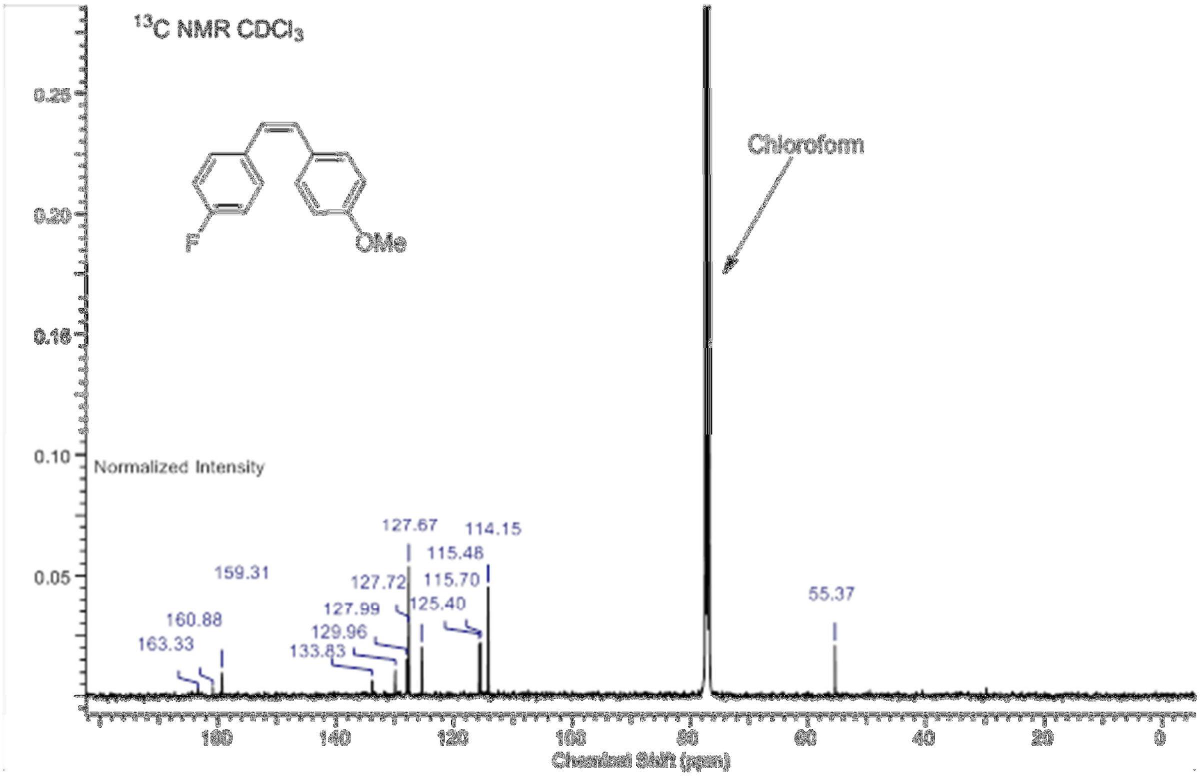

13C NMR CDCl3
F
OMe
Chloroform
Normalized Intensity
127.67
114.15
115.48
159.31
127.72
115.70
160.88
127.99
125.40
55.37
129.96
163.33
133.83

[9-BBN(I-DCDM)$_2$][HB(C$_6$F$_5$)$_3$] (**13**)

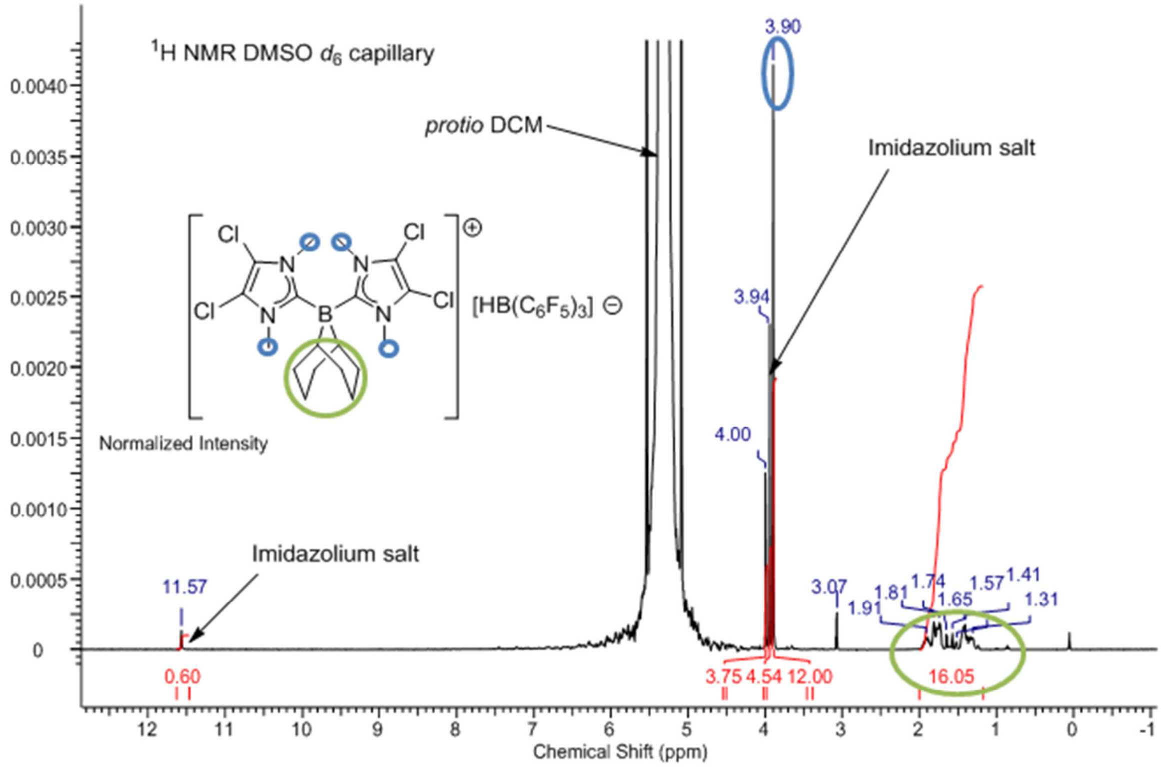

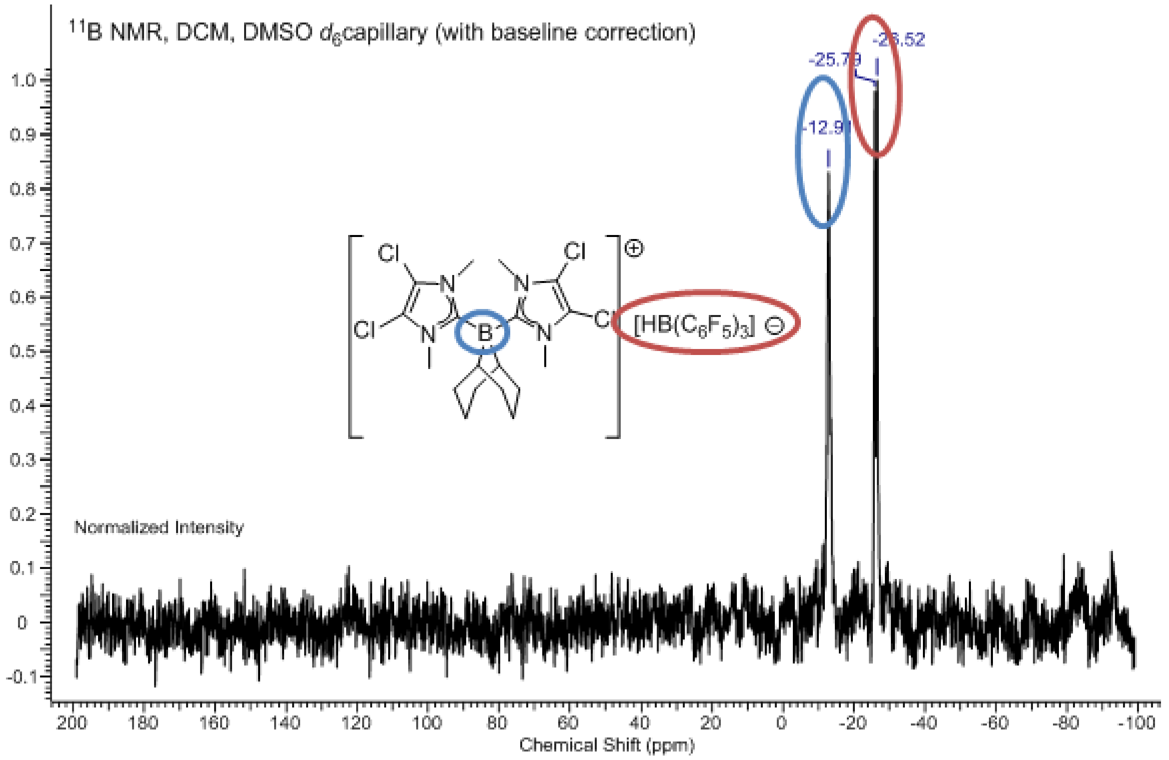

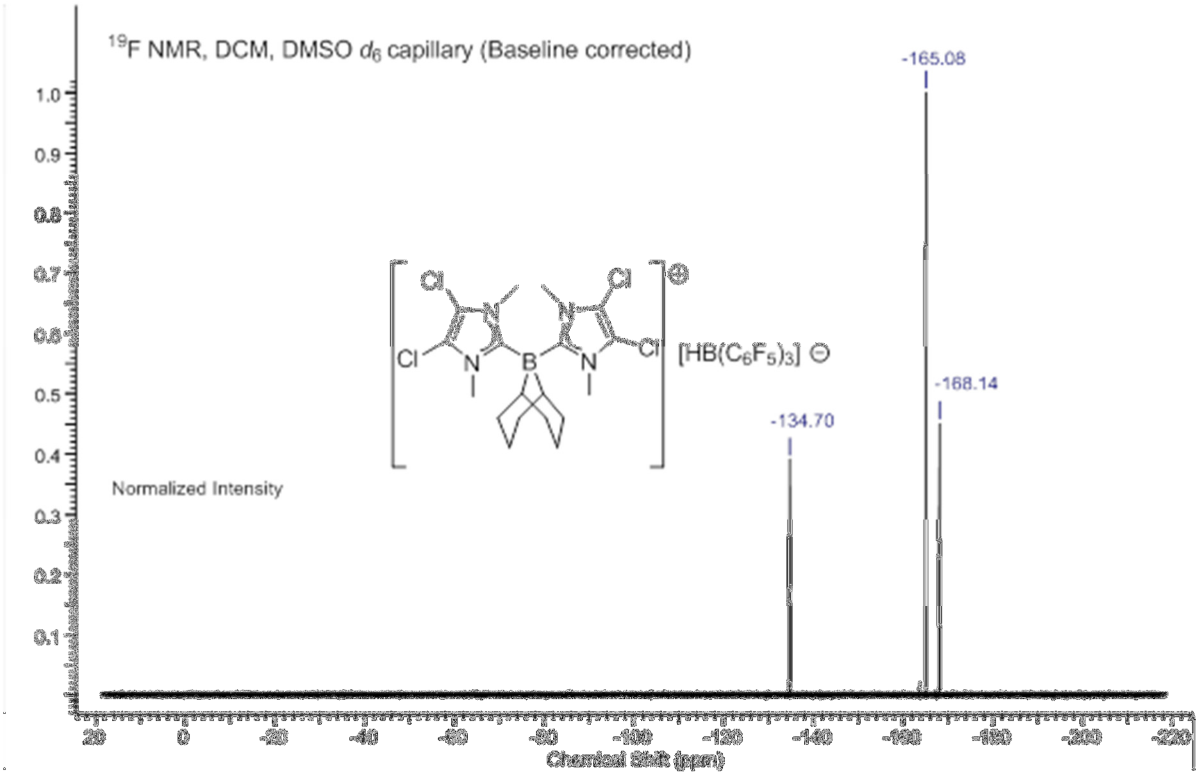

19F NMR, DCM, DMSO d6 capillary (Baseline corrected)
-165.08
-168.14
-134.70
[HB(C6F5)3]
Normalized Intensity
Chemical Shift (ppm)

[NBu_4][$HB(C_6F_5)_3$]

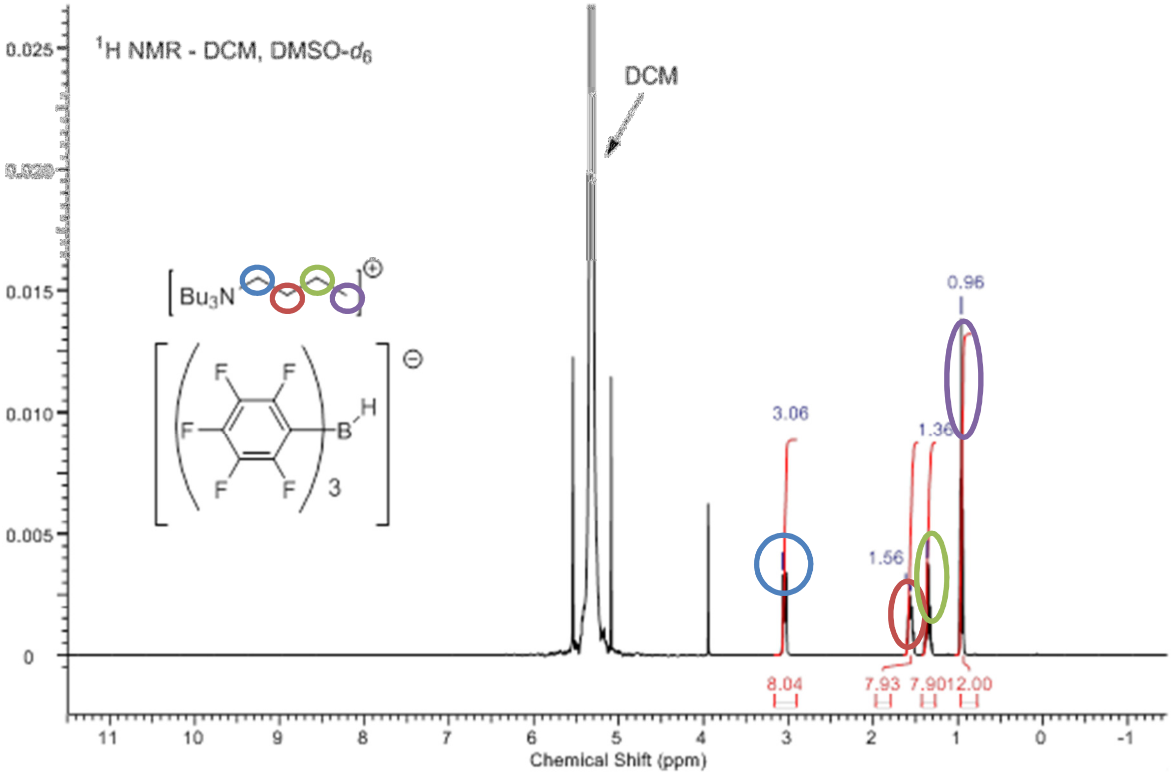

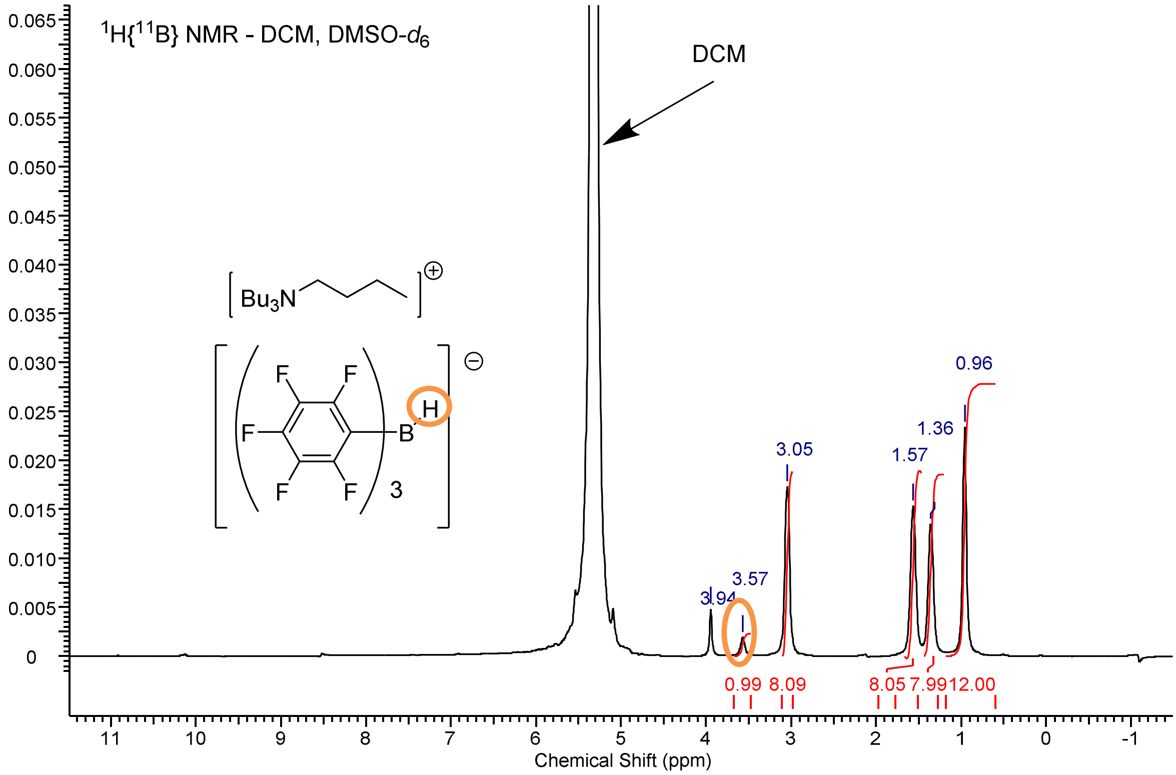

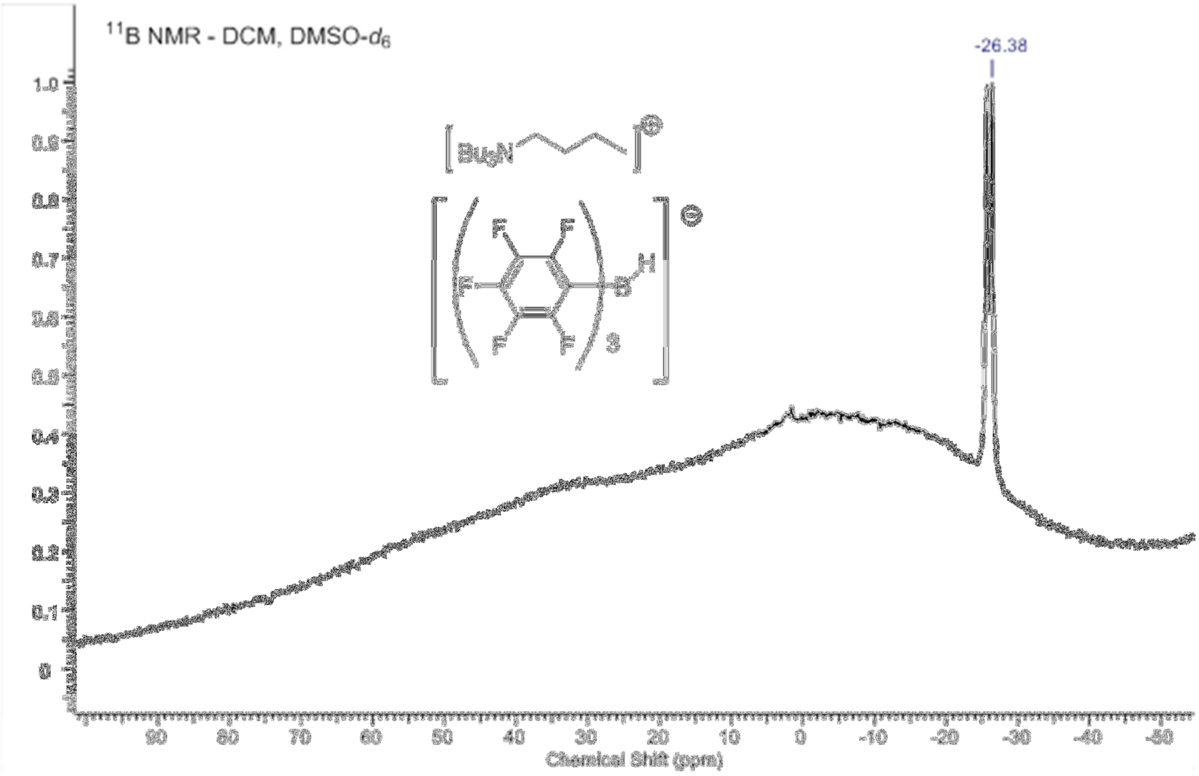

^{11}B NMR - DCM, DMSO-d_6
-26.38
Bu$_3$N
F
B
H
3
Chemical Shift (ppm)

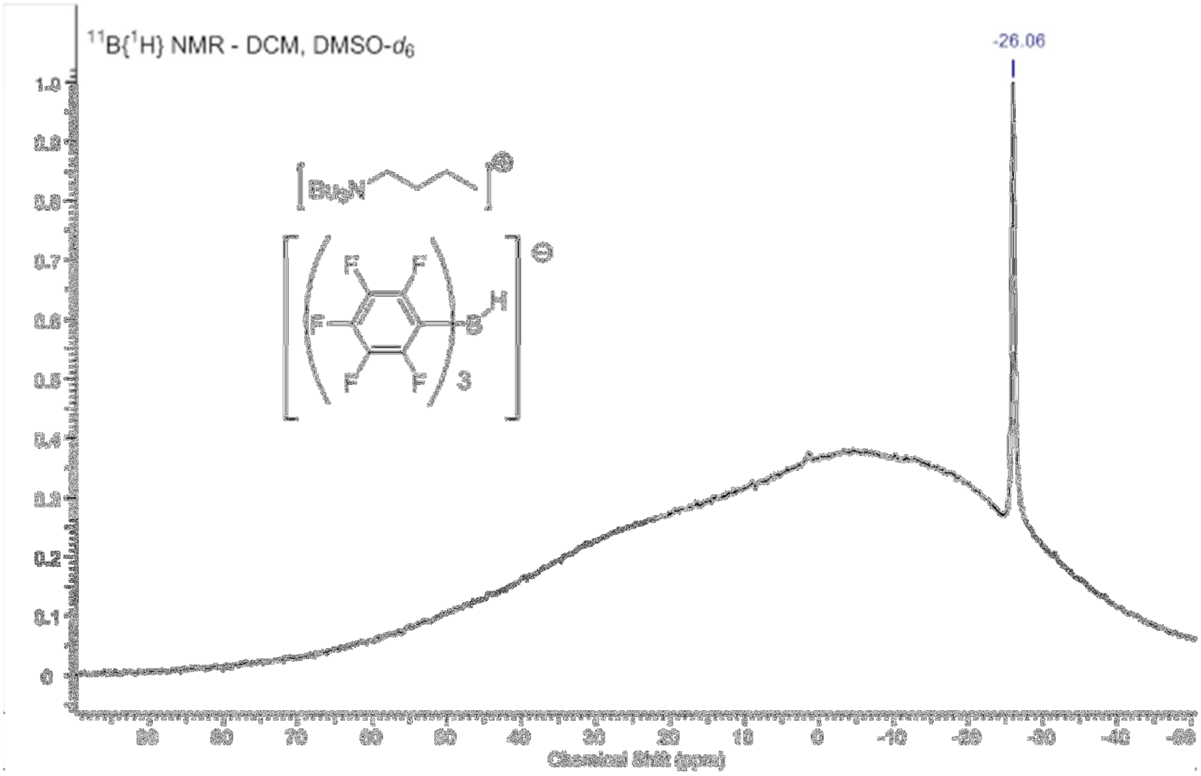

^{11}B{^{1}H} NMR - DCM, DMSO-d_6
-26.06
Bu$_3$N
F
B
H
3
Chemical Shift (ppm)

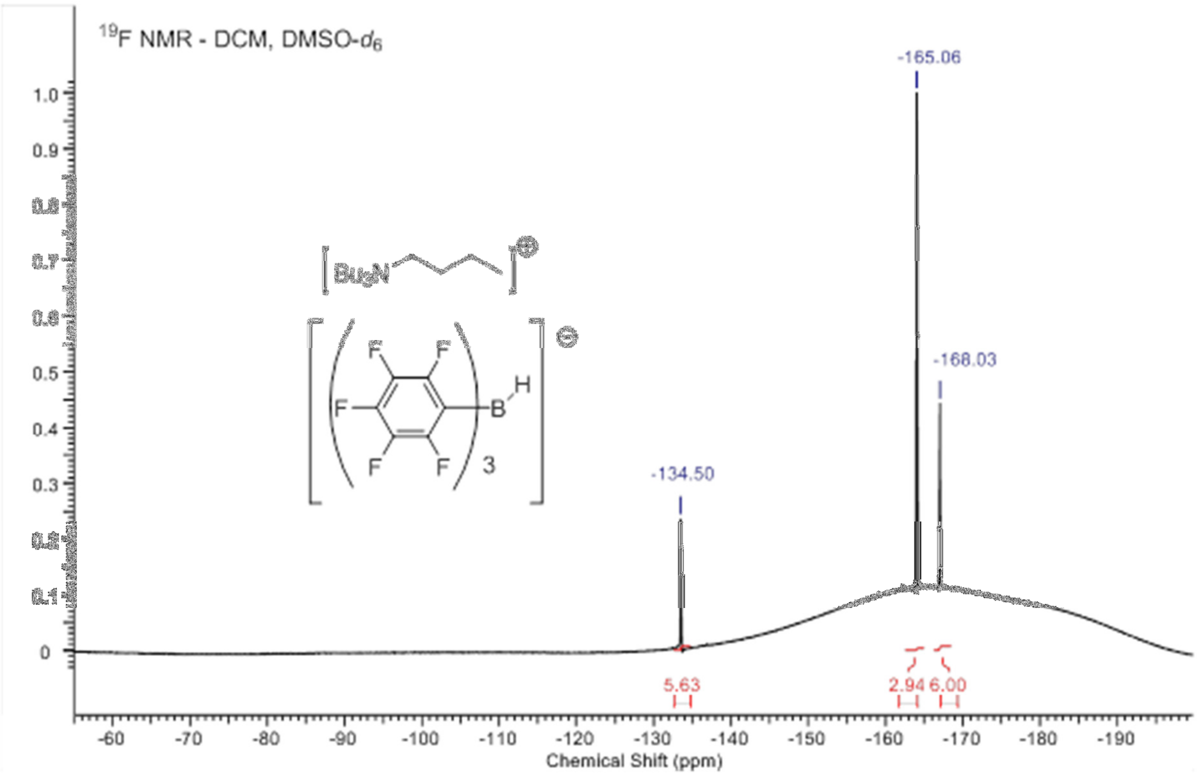

19F NMR - DCM, DMSO-d6
-165.06
-168.03
-134.50
5.63
2.94
6.00
Chemical Shift (ppm)

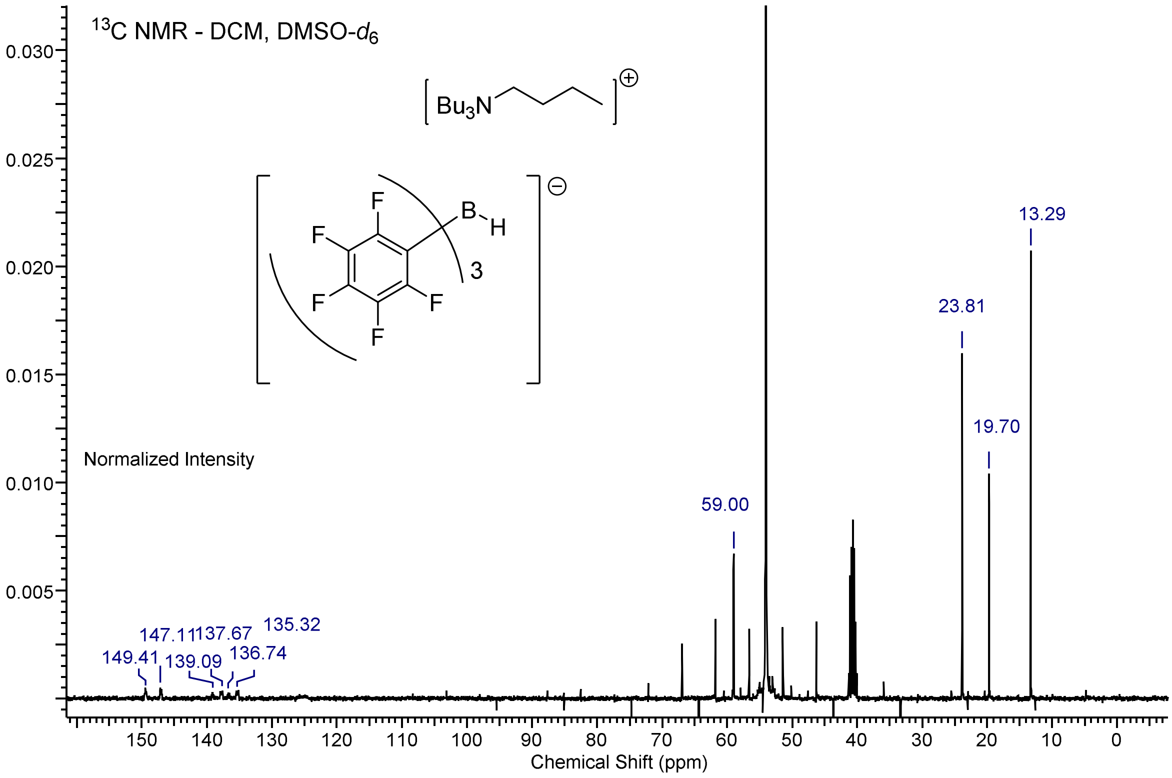

13C NMR - DCM, DMSO-d6
Normalized Intensity
13.29
23.81
19.70
59.00
149.41
147.11
137.67
139.09
135.32
136.74
Chemical Shift (ppm)

Tetrabutylammonium [(*Z*)-(2-*para*-tolyl)ethenyl]tris(pentafluorophenyl)borate (**14a**)

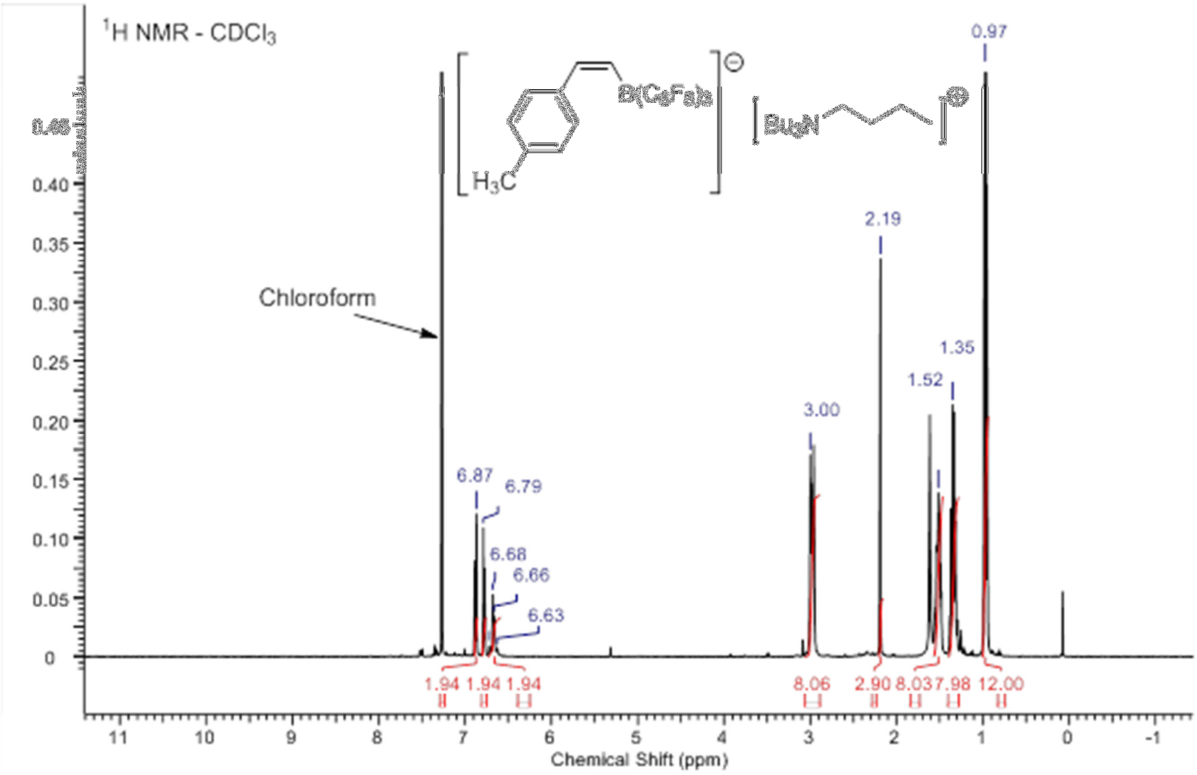

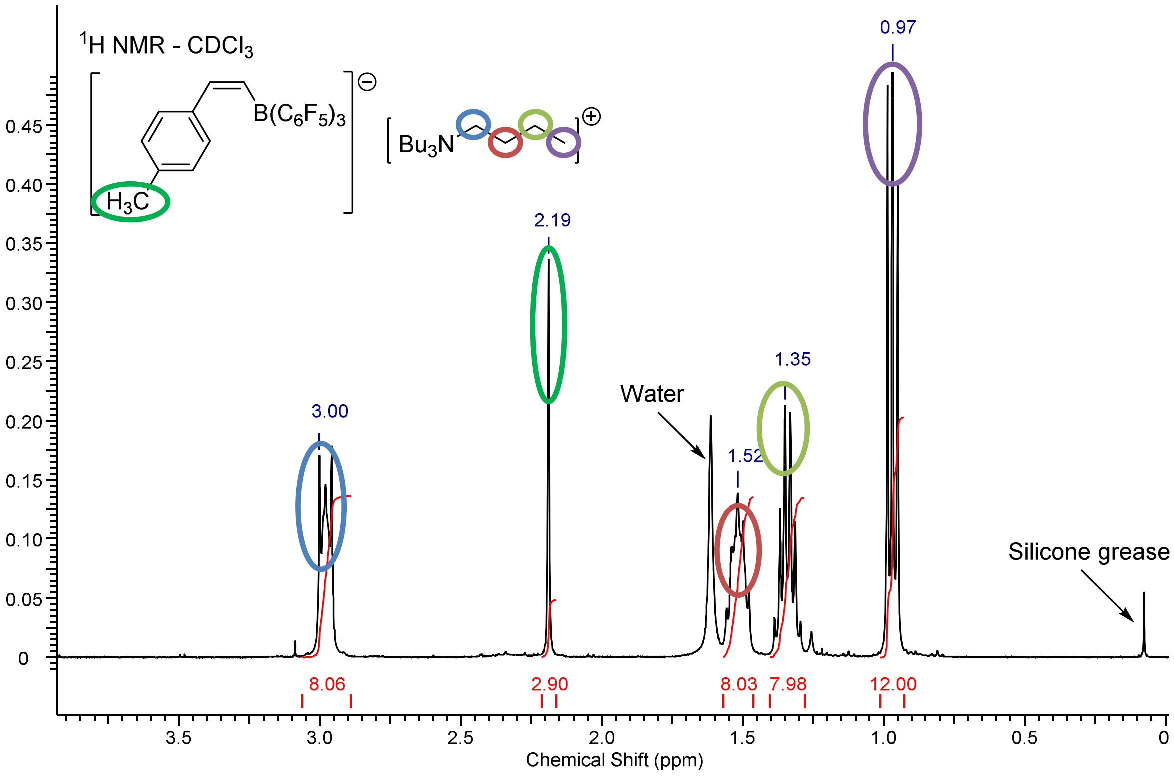

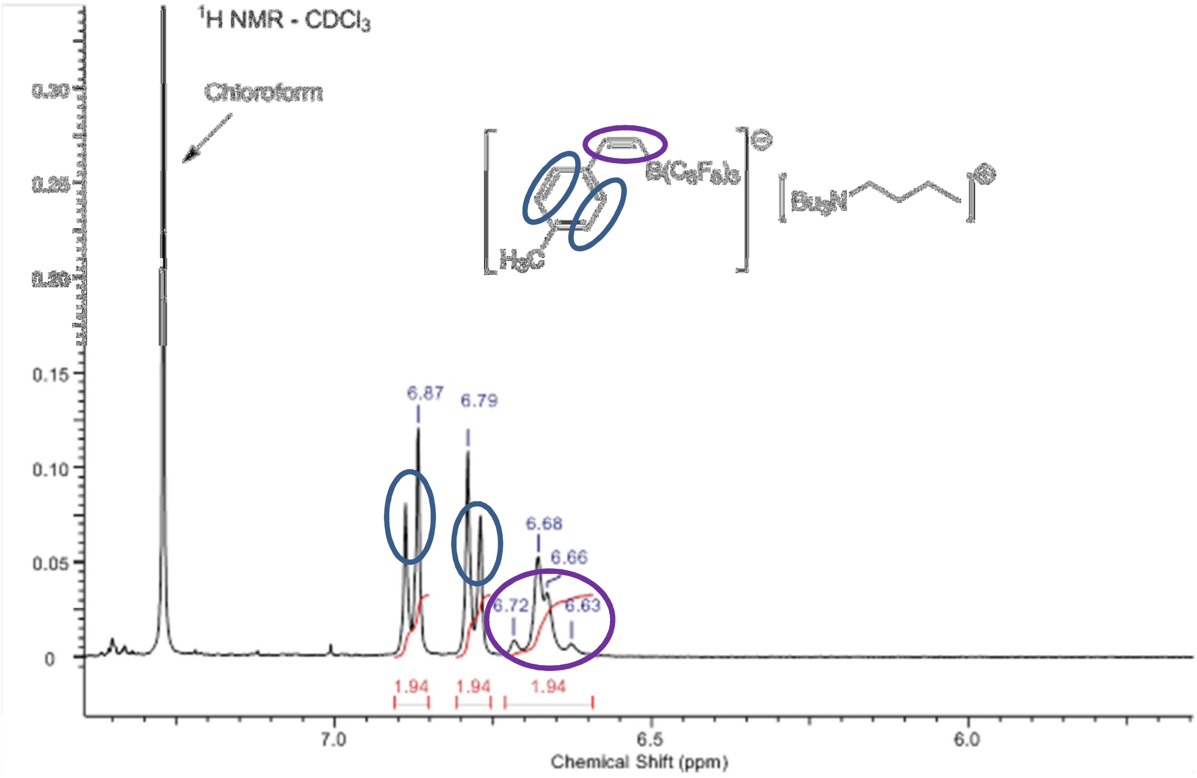

^{1}H NMR - CDCl$_3$
Chloroform
B(C$_6$F$_5$)$_3$
Bu$_3$N
H$_3$C
6.87
6.79
6.68
6.66
6.72
6.63
1.94
1.94
1.94
0.15
0.10
0.05
0
7.0
6.5
6.0
Chemical Shift (ppm)

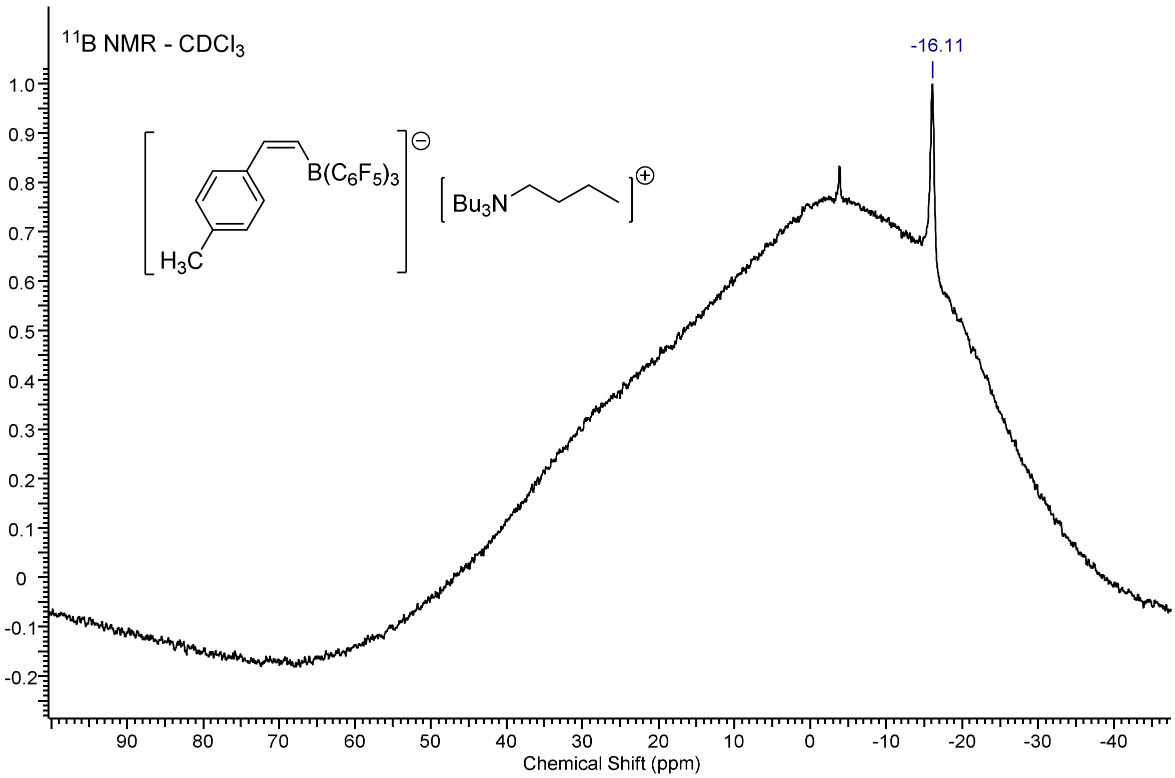

^{11}B NMR - CDCl$_3$
-16.11
B(C$_6$F$_5$)$_3$
Bu$_3$N
H$_3$C
1.0
0.9
0.8
0.7
0.6
0.5
0.4
0.3
0.2
0.1
0
-0.1
-0.2
90
80
70
60
50
40
30
20
10
0
-10
-20
-30
-40
Chemical Shift (ppm)

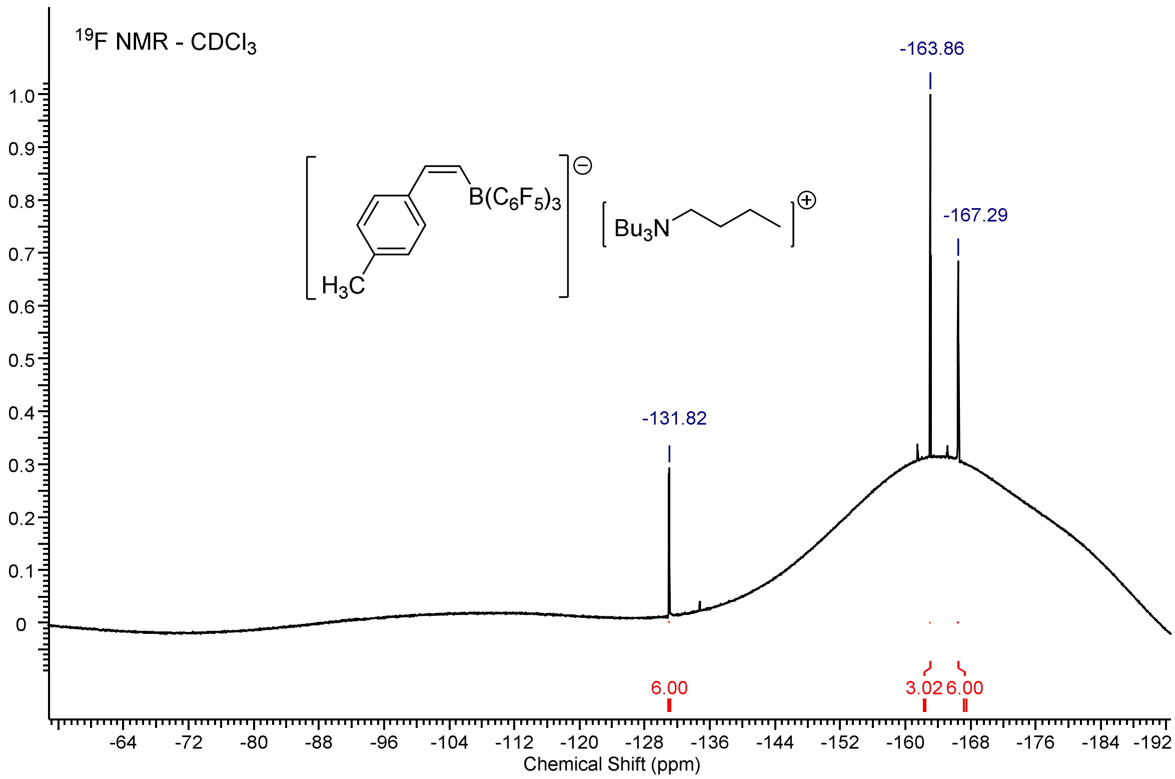

^{19}F NMR - CDCl$_3$
-163.86
-167.29
-131.82
B(C$_6$F$_5$)$_3$
Bu$_3$N
H$_3$C
6.00
3.02
6.00
Chemical Shift (ppm)

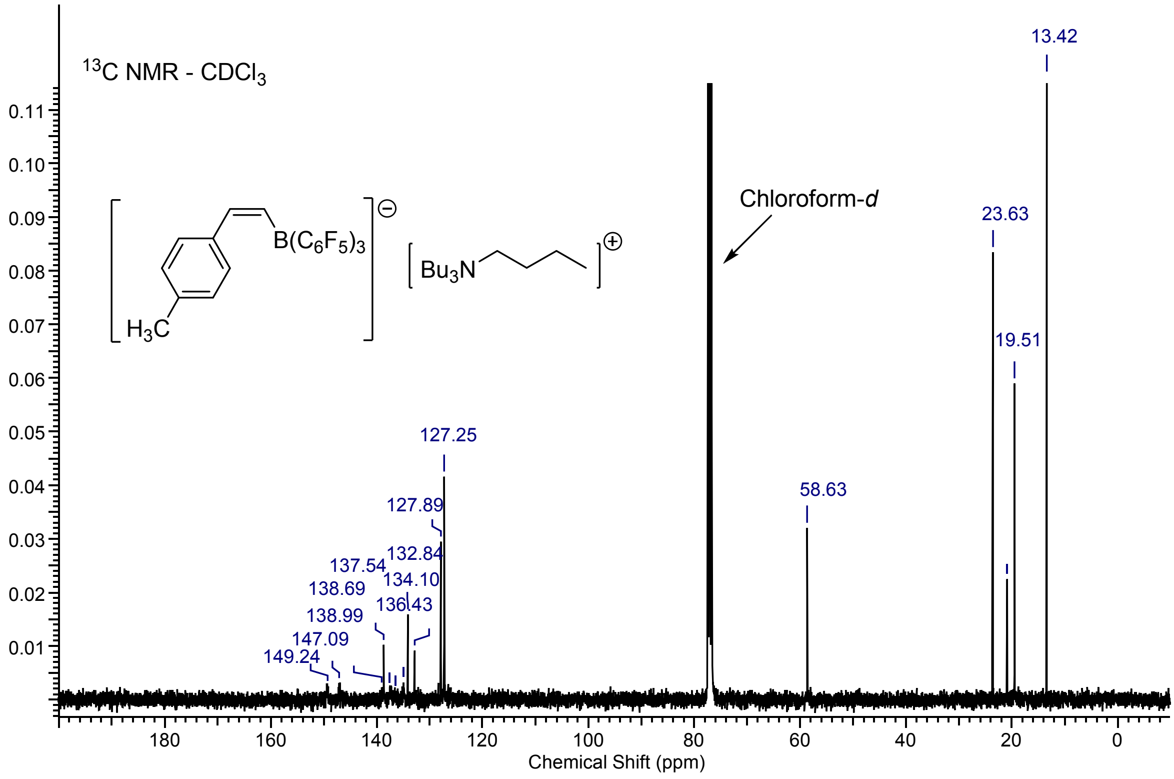

^{13}C NMR - CDCl$_3$
13.42
23.63
19.51
58.63
127.25
127.89
132.84
134.10
136.43
137.54
138.69
138.99
147.09
149.24
Chloroform-*d*
B(C$_6$F$_5$)$_3$
Bu$_3$N
H$_3$C
Chemical Shift (ppm)

Tetrabutylammonium [(*Z*)-2-phenylethenyl]tris(pentafluorophenyl)borate (**14b**)

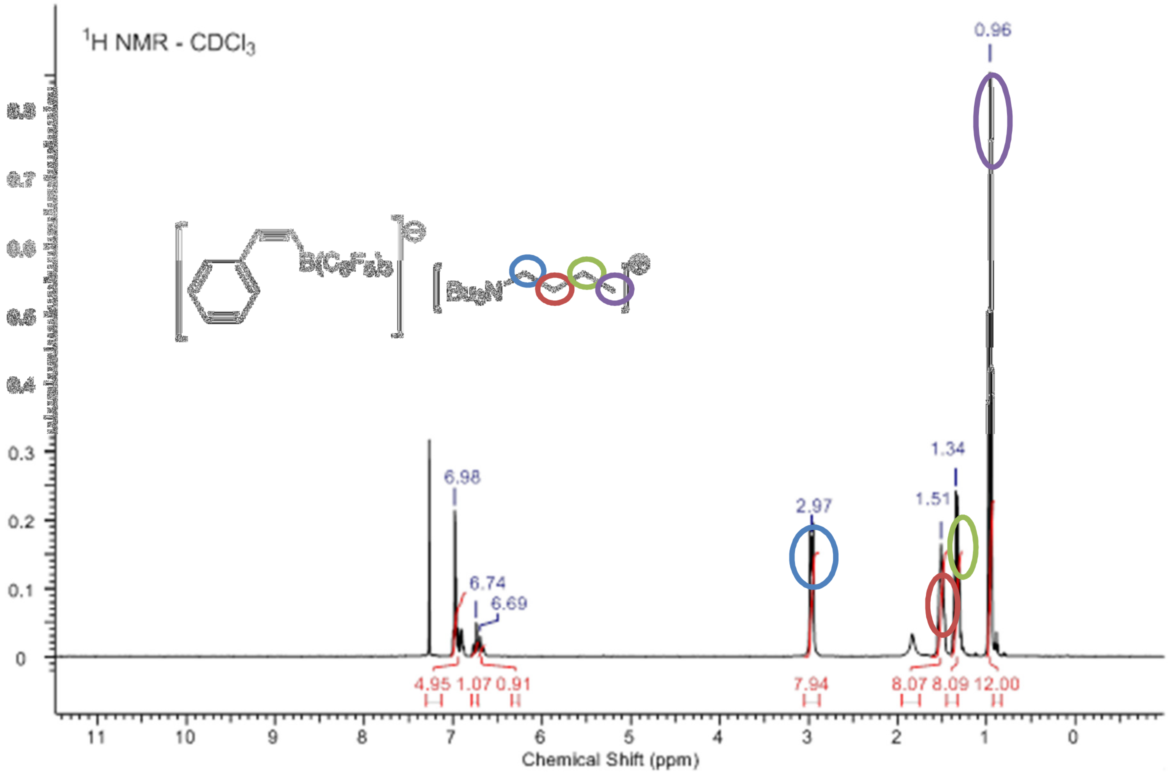

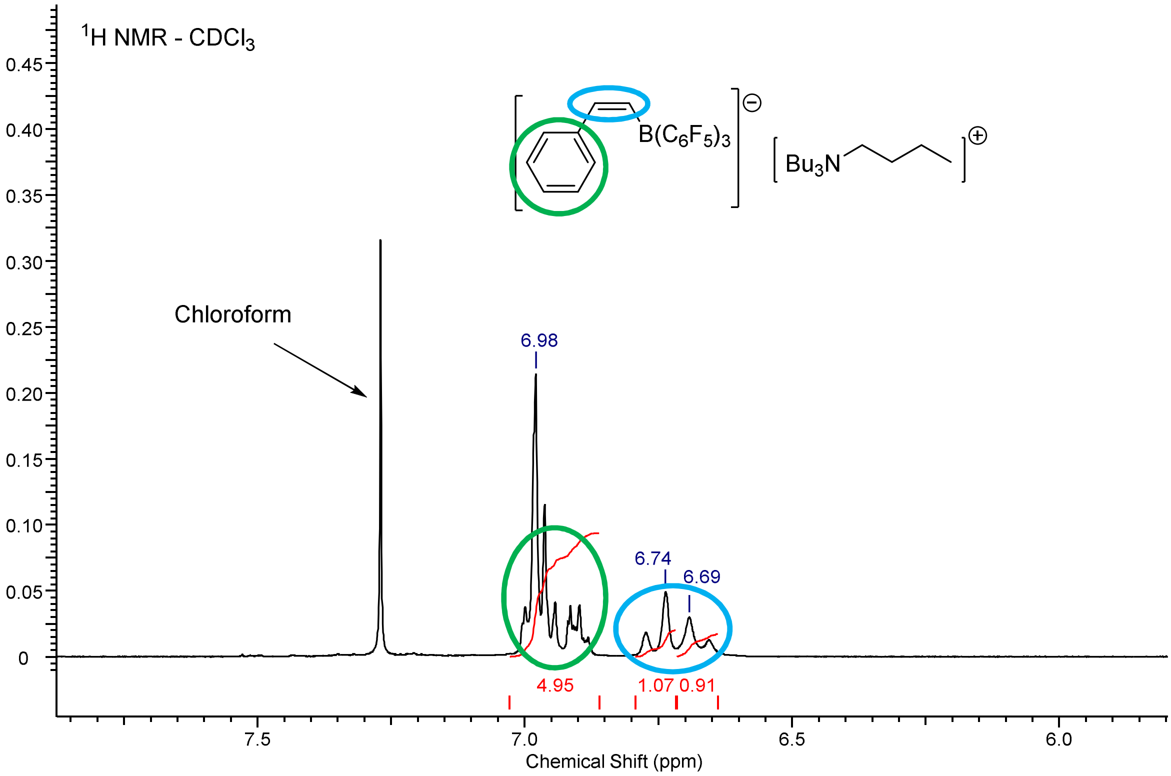

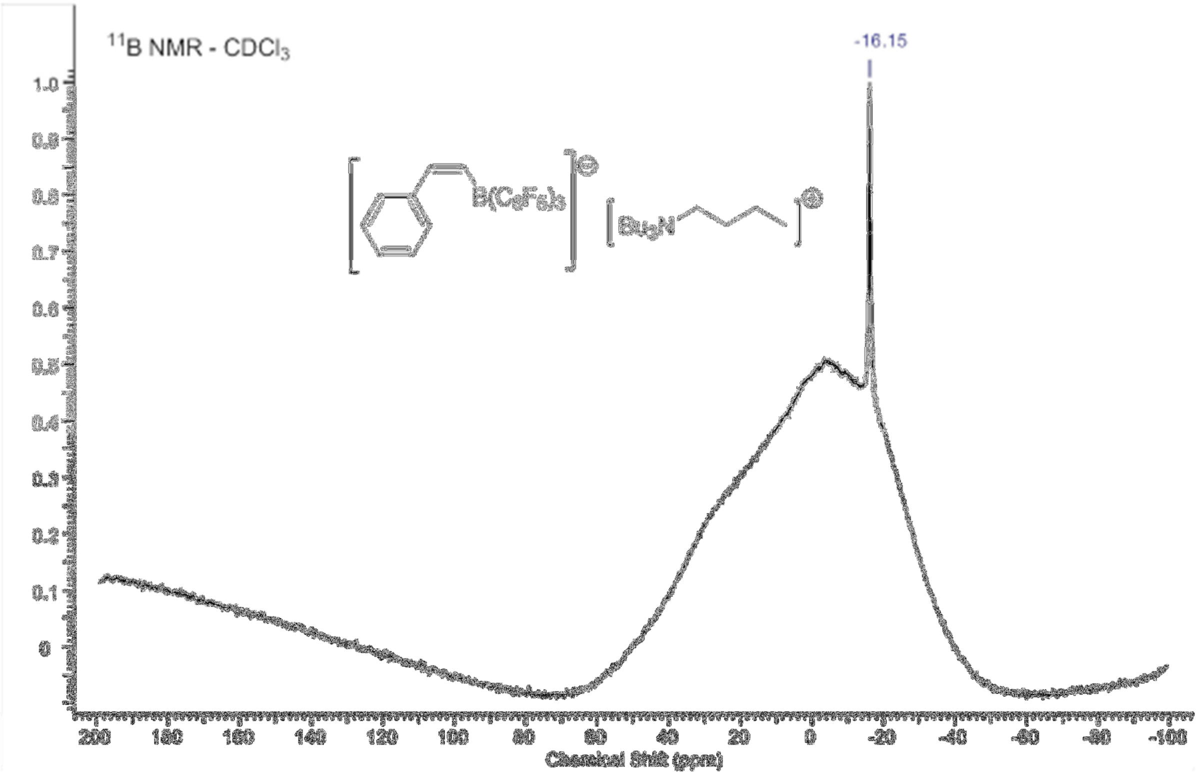
11B NMR - CDCl3
-16.15
Chemical Shift (ppm)

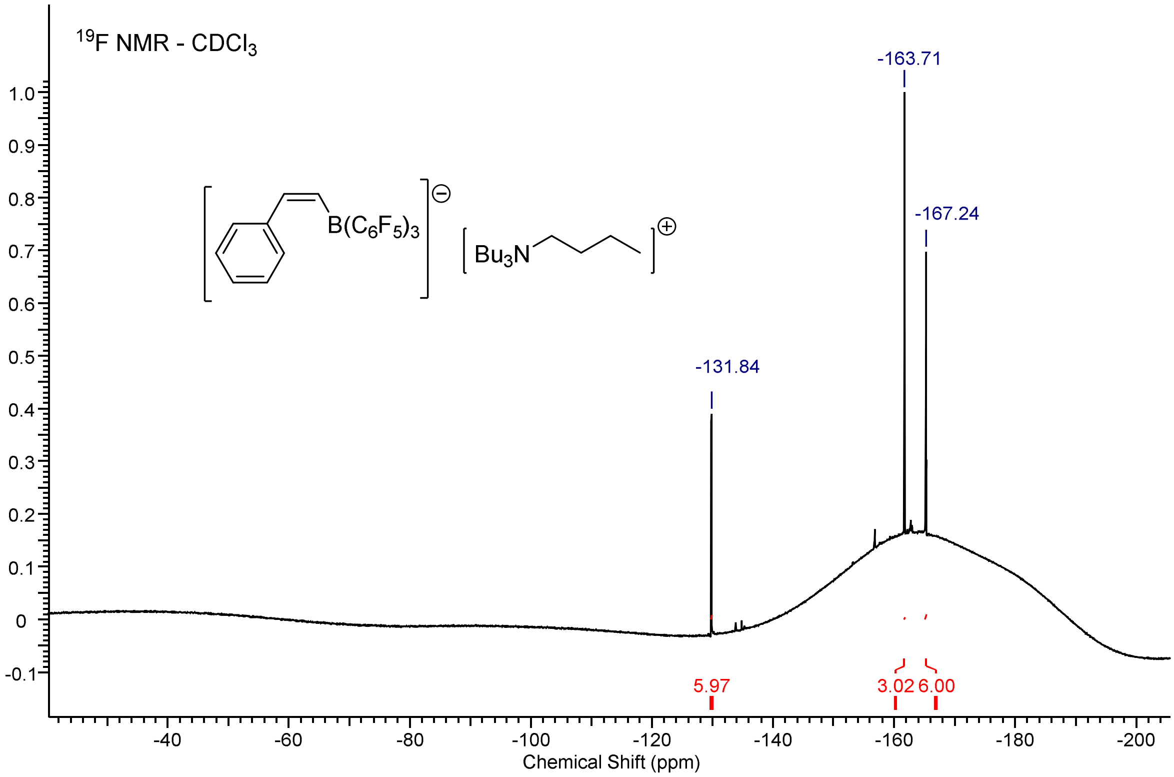
19F NMR - CDCl3
-163.71
-167.24
-131.84
B(C6F5)3
Bu3N
5.97
3.02
6.00
Chemical Shift (ppm)

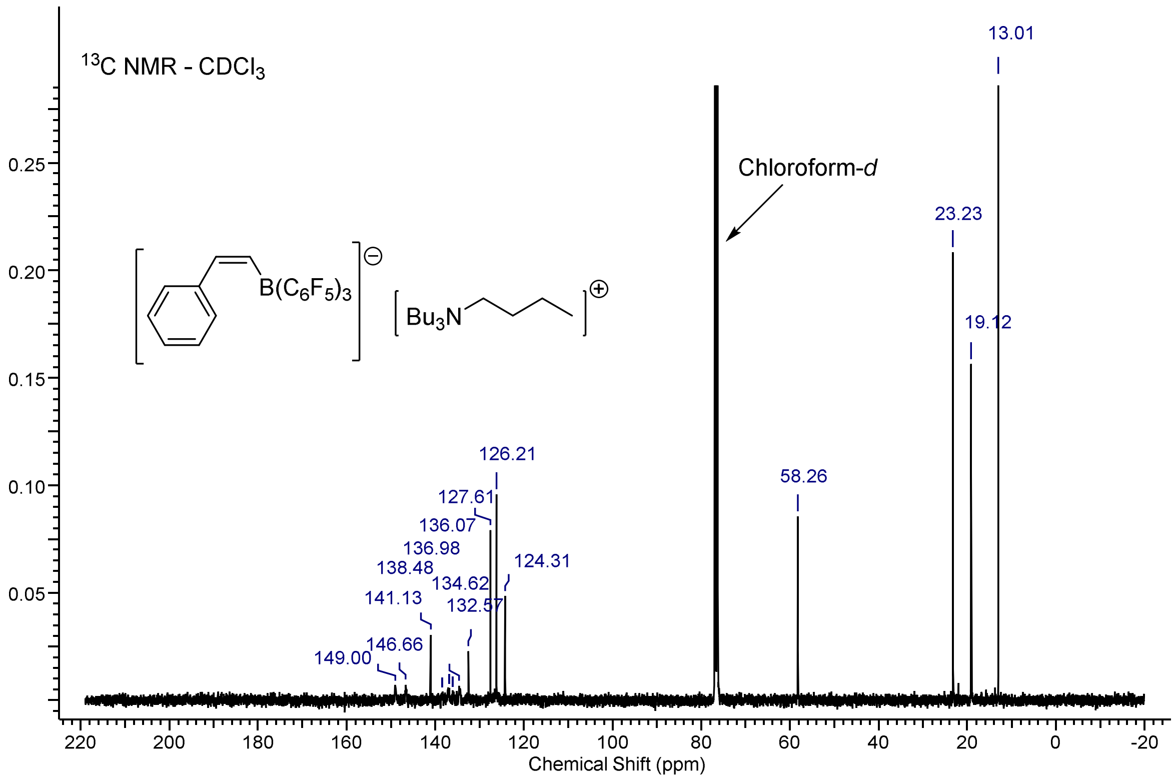

13C NMR - CDCl3
B(C6F5)3
Bu3N
Chloroform-d
13.01
23.23
19.12
58.26
126.21
127.61
136.07
136.98
138.48
124.31
134.62
141.13
132.57
146.66
149.00
Chemical Shift (ppm)

Tetrabutylammonium [(*Z*)-(2-*para*-methoxyphenyl)ethenyl]tris(pentafluorophenyl)borate (**14c**)

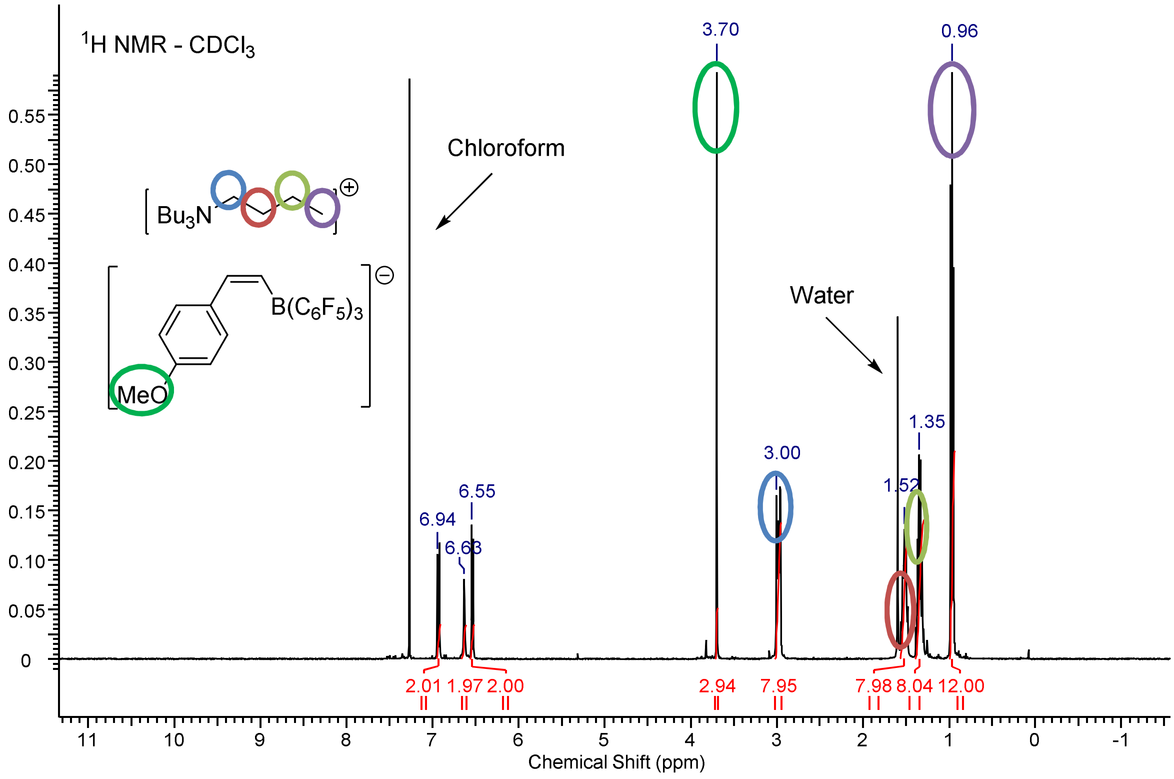

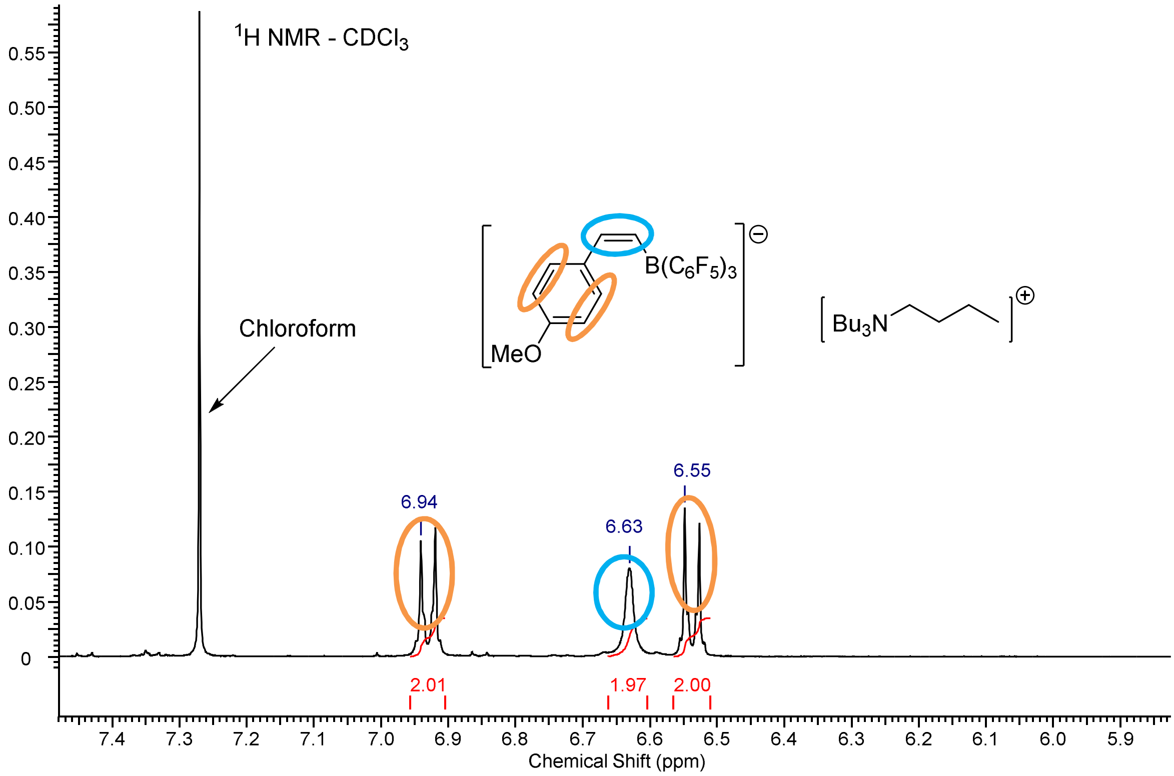

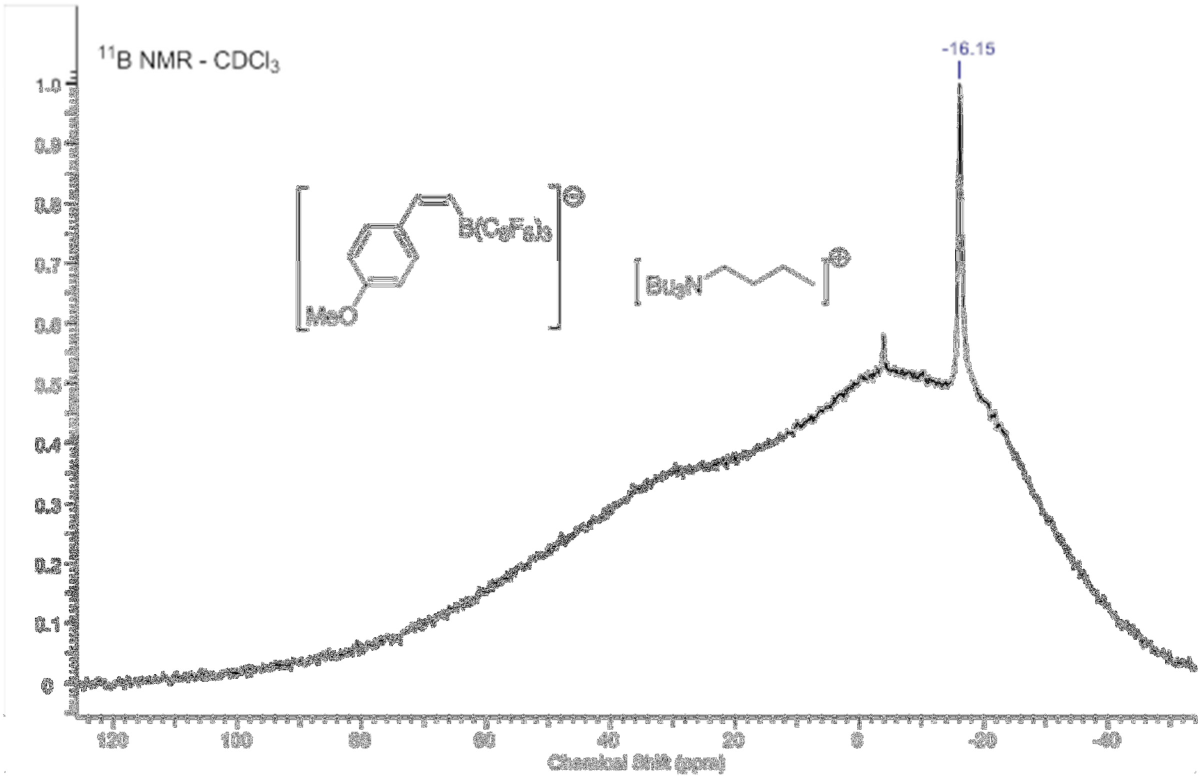

11B NMR - CDCl3
-16.15
B(C6F5)3
MeO
Bu3N
Chemical Shift (ppm)

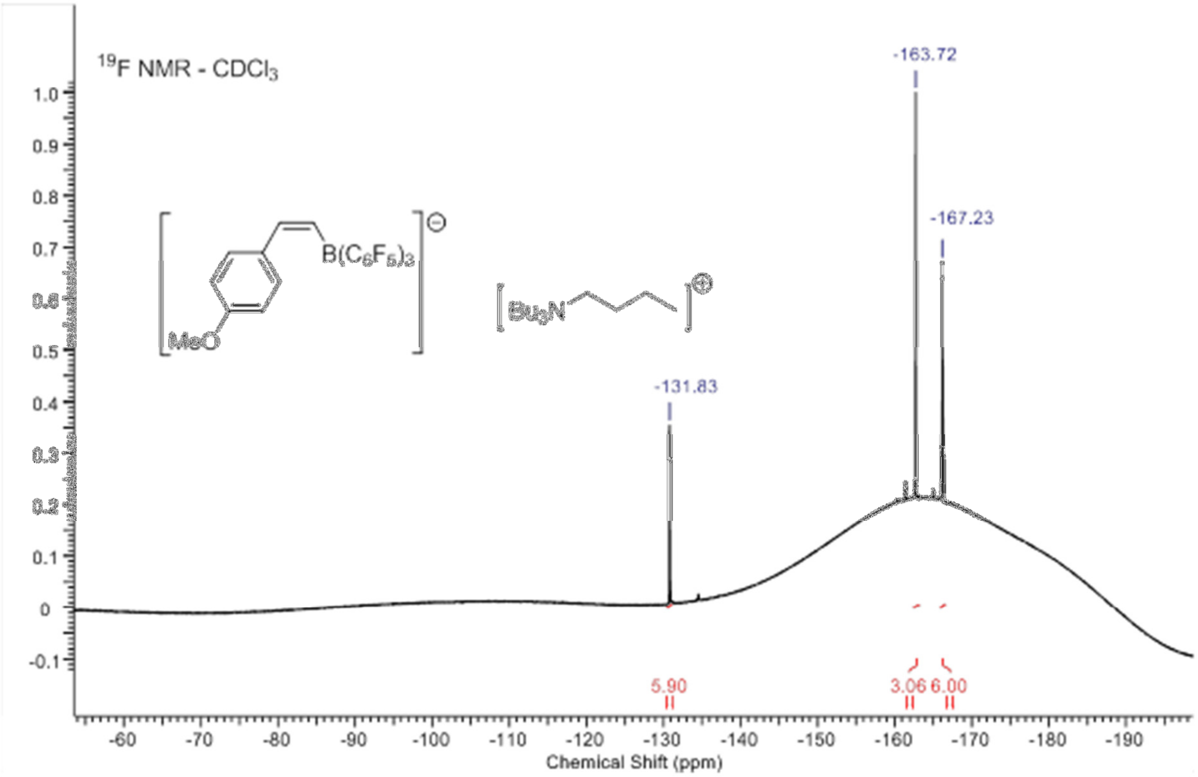

19F NMR - CDCl3
-163.72
-167.23
-131.83
B(C6F5)3
MeO
Bu3N
5.90
3.06
6.00
Chemical Shift (ppm)

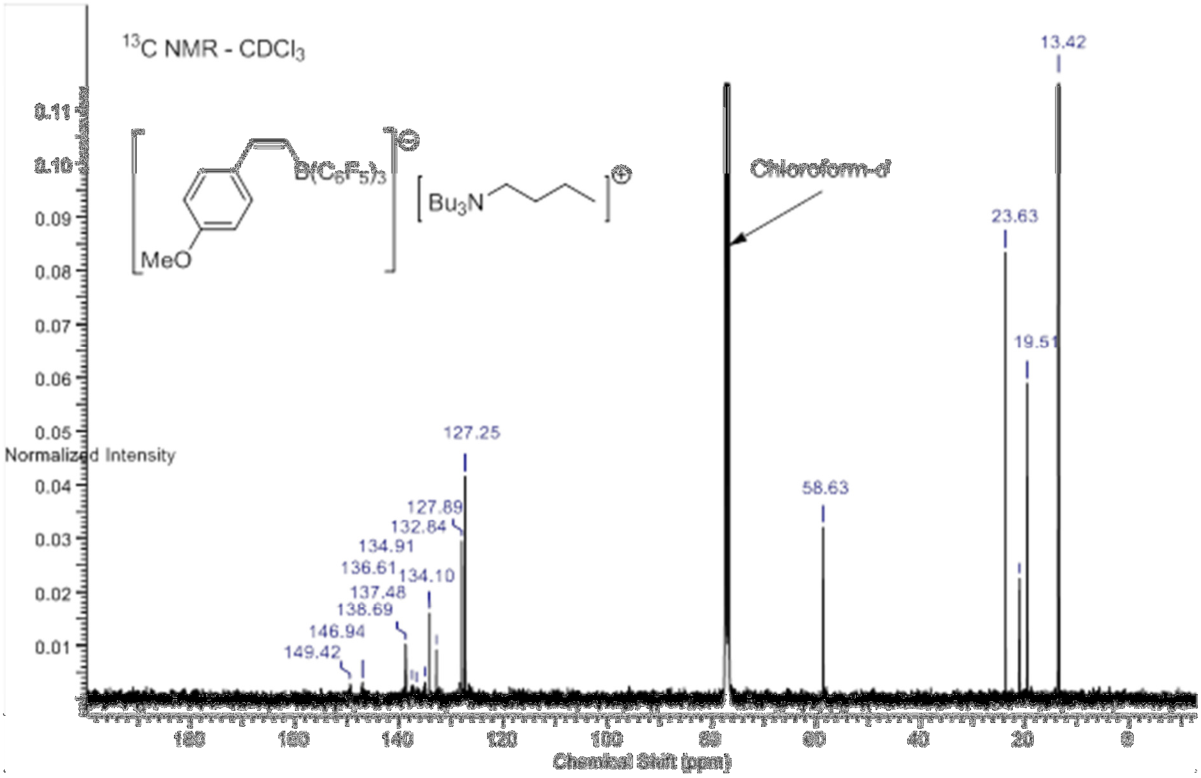

13C NMR - CDCl3
B(C6F5)3
MeO
Bu3N
Chloroform-d
13.42
23.63
19.51
58.63
127.25
127.89
132.84
134.91
136.61
134.10
137.48
138.69
146.94
149.42
Normalized Intensity
Chemical Shift (ppm)
